# The 2020 China report of the *Lancet* Countdown on health and climate change

**DOI:** 10.1016/S2468-2667(20)30256-5

**Published:** 2020-12-02

**Authors:** Wenjia Cai, Chi Zhang, Hoi Ping Suen, Siqi Ai, Yuqi Bai, Junzhe Bao, Bin Chen, Liangliang Cheng, Xueqin Cui, Hancheng Dai, Qian Di, Wenxuan Dong, Dejing Dou, Weicheng Fan, Xing Fan, Tong Gao, Yang Geng, Dabo Guan, Yafei Guo, Yixin Hu, Junyi Hua, Cunrui Huang, Hong Huang, Jianbin Huang, Tingting Jiang, Kedi Jiao, Gregor Kiesewetter, Zbigniew Klimont, Pete Lampard, Chuanxi Li, Qiwei Li, Ruiqi Li, Tiantian Li, Borong Lin, Hualiang Lin, Huan Liu, Qiyong Liu, Xiaobo Liu, Yufu Liu, Zhao Liu, Zhidong Liu, Zhu Liu, Shuhan Lou, Chenxi Lu, Yong Luo, Wei Ma, Alice McGushin, Yanlin Niu, Chao Ren, Zhehao Ren, Zengliang Ruan, Wolfgang Schöpp, Jing Su, Ying Tu, Jie Wang, Qiong Wang, Yaqi Wang, Yu Wang, Nick Watts, Congxi Xiao, Yang Xie, Hui Xiong, Mingfang Xu, Bing Xu, Lei Xu, Jun Yang, Lianping Yang, Le Yu, Yujuan Yue, Shaohui Zhang, Zhongchen Zhang, Jiyao Zhao, Liang Zhao, Mengzhen Zhao, Zhe Zhao, Jingbo Zhou, Peng Gong

**Affiliations:** a Department of Earth System Science, Tsinghua University, Beijing, China; b State Key Joint Laboratory of Environmental Simulation and Pollution Control, State Environmental Protection Key Laboratory of Sources and Control of Air Pollution Complex, School of Environment, Tsinghua University, Beijing, China; c Vanke School of Public Health, Tsinghua University, Beijing, China; d Institute of Public Safety Research, Tsinghua University, Beijing, China; e Department of Engineering Physics, Tsinghua University, Beijing, China; f School of Architecture, Tsinghua University, Beijing, China; g School of Humanities, Tsinghua University, Beijing, China; h People's Bank of China School of Finance, Tsinghua University, Beijing, China; i Research Center for Public Health, Tsinghua University, Beijing, China; j Institute of Population Research, Peking University, Beijing, China; k College of Environmental Sciences and Engineering, Peking University, Beijing, China; l School of Public Health, Sun Yat-sen University, Guangzhou, China; m College of Public Health, Zhengzhou University, Zhengzhou, China; n School of Environment, Beijing Normal University, Beijing, China; o Baidu Research, Baidu, Beijing, China; p Institute of Environment and Ecology, Shandong Normal University, Jinan, China; q School of Business, Shandong Normal University, Jinan, China; r The Bartlett School of Construction and Project Management, Institute for Global Health, University College London, London, UK; s State Key Laboratory of Infectious Disease Prevention and Control, National Institute for Communicable Disease Control and Prevention, Chinese Center for Disease Control and Prevention, Beijing, China; t Chinese Center for Disease Control and Prevention Key Laboratory of Environment and Population Health, National Institute of Environmental Health, Chinese Center for Disease Control and Prevention, Beijing, China; u Department of Statistics and Data Science, Southern University of Science and Technology, Shenzhen, China; v Faculty of Architecture, The University of Hong Kong, Hong Kong Special Administrative Region, China; w Department of Epidemiology, School of Public Health, Cheeloo College of Medicine, Shandong University, Jinan, China; x Shandong University Climate Change and Health Center, Shandong University, Jinan, China; y School of Economics and Management, Beihang University, Beijing, China; z Air Quality and Greenhouse Gases Programme, International Institute for Applied Systems Analysis, Laxenburg, Austria; aa Department of Health Sciences, University of York, York, UK; ab State Key Laboratory of Remote Sensing Science, Aerospace Information Research Institute, Chinese Academy of Sciences, Beijing, China; ac The State Key Laboratory of Numerical Modeling for Atmospheric Sciences and Geophysical Fluid Dynamics, Institute of Atmospheric Physics, Chinese Academy of Sciences, Beijing, China; ad School of Computer Science and Technology, University of Science and Technology of China, Hefei, China; ae Rutgers Business School, Rutgers, the State University of New Jersey, New Brunswick, NJ, USA; af Institute for Environmental and Climate Research, Jinan University, Guangzhou, China; ag Institute for Global Health, University College London, London, UK

## Executive summary

Left unmitigated, climate change poses a catastrophic risk to human health, requiring an urgent and concerted response from every country. As the home to one fifth of the world's population and the largest emitter of carbon dioxide globally, China's interventions in climate change are of pivotal importance, both to human health and to the planet. Similar to other countries, climate change mitigation and adaptation would bring immense health benefits for China's 1·4 billion people, and building these considerations into any COVID-19 recovery strategy and the detailed pathway to fulfil the 2060 carbon neutrality pledge will ensure it improves human wellbeing, both now and in the future. Decisions made over the coming months and years will establish the course of climate change policy for decades to come.

To meet this challenge, Tsinghua University (Beijing, China), partnering with University College London (London, UK) and 17 Chinese and international institutions, has produced the *Lancet* Countdown China report, focusing at the national level and building on the work of the global *Lancet* Countdown. Drawing on international methods and frameworks, this report aims to understand and track the links between public health and climate change at the national level. This paper is one part of the *Lancet* Countdown's broader efforts to develop regional expertise and understanding. Uniquely, the data and results in this report are presented at the provincial level where possible, to facilitate the targeted response strategies for local decision makers.

### The effect of climate change on health and the response in China

Taken as a whole, the findings of the 23 indicators convey two key messages.

The first message is that the health effects from climate change in China are accelerating, posing an unacceptably high amount of health risk if global temperatures continue to rise. Every province is affected, each with its unique health threats, and targeted response strategies should be made accordingly.

The effects of climate change, manifested in rising temperatures, more extreme weather events, and shifting vector ecology, are being felt in China. Heatwave-related mortality has risen by a factor of four from 1990 to 2019, reaching 26 800 deaths in 2019. The monetised cost of the high number of deaths is equivalent to the average annual income of 1·4 million people in China. Older people (>65 years old), who face a 10·4% higher risk of dying during a heatwave, endured an average of 13 more heatwave days in 2019 compared with the 1986–2005 baseline. For outdoor workers, their potential heat-related labour productivity loss reached 0·5% of total national work hours, costing 1% of China's gross domestic product (GDP), equivalent to its annual fiscal expenditure on science and technology. Driven in part by rising temperatures and a changing climate, the advent of more extreme wildfires and the spread of dengue fever will in turn lead to profound health effects.

Different regions have unique health threats, requiring a targeted response—19 provinces have had an at least 10% rise over the past two decades in three or more of the six health effect indicators reported. Importantly, many highly populated and economically advanced provinces, such as Henan, Shandong, and Zhejiang, are faced with health risks that are larger and more rapidly accelerating than others.

The second message is that impressive and concerted improvements have been made across several sectors in China; however, the gap in the country's response to the health effects of climate change is large.

In some sectors, China has taken large steps to address climate change. Solar power generation is growing at an unprecedented rate of 26·5% per year, rising to 26·8 gigawatts (GW) of newly installed capacity in 2019. Investments in low-carbon energy are now nine times greater than those in fossil fuels (rising from a 1:1 ratio in 2008); and, providing 4·1 million jobs in 2018, renewable energy now employs more people in China than fossil fuel extraction industries. As a result of strong policy measures, severe air pollution has also decreased, with a 28% reduction in annual average particulate matter of 2·5 μm or less (PM_2·5_) concentration in cities from 2015 to 2019, resulting in 90 000 fewer PM_2·5_-related premature deaths annually. These air pollution control policies also act to mitigate climate change and have resulted in a decline in China's coal share in total primary energy supply from 66% in 2014 to 59% in 2018. Showing leadership at the subnational level, three provinces already have a provincial health and climate change plan in place, with four more provinces underway.

However, although these changes have been rapid, more shifts of a greater size are necessary to enact a response that is of the scale required to fulfil China's carbon neutrality by 2060 pledge and to minimise the rising health burdens of climate change, both in China and around the world. Although renewable energy use is rising, coal stills holds a 59% share of the total primary energy supply in China. Fossil fuel subsidies were US$41·9 billion in 2018, without considering the contribution of fossil fuels to the estimated $10·7 billion economic losses because of premature mortality from PM_2·5_ air pollution. Although there have been substantial reductions in air pollution, 42% of China's population still live in areas that do not meet the interim air quality guidelines from WHO, and almost all cities have PM_2·5_ concentrations more than the recommended annual average of 10 μg/m^3^. The health effects of climate change are not adequately recognised or addressed, as climate change is not referenced in the Healthy China Action Plan (2019–30), and China is yet to introduce a standalone national adaptation plan for health. Taking a broader perspective, media coverage and individual engagement in health and climate change are low, with little spread of knowledge and engagement. China will need to scale up progress in all sectors to counteract the rising curve of the health risks from climate change.

### Policy recommendations from the 2020 *Lancet* Countdown China report

Five recommendations are proposed to key stakeholders in health and climate change in China:

(1) Enhance interdepartmental cooperation. Climate change is a challenge that requires an integrated response from all sectors. Although China commits to integrate health into all policies, substantial interdepartmental cooperation among health, environment, energy, economic, financial, and education authorities is urgently needed.
(2) Strengthen health emergency preparedness. Although the amount of health emergency preparedness in China would be greatly enhanced after COVID-19, knowledge and findings on current and future climate-related health threats still do not have enough attention and should be fully integrated into the emergency preparedness and response system, so that future health service, medical supplies, and infrastructure needs could be planned ahead.
(3) Support research and raise awareness. Additional financial support should be allocated to health and climate change research in China, to enhance the knowledge of health system adaptation, mitigation measures, and their resulting health benefits. At the same time, media and academia should be fully motivated to raise awareness on this topic for the public and for politicians. Additionally, the Government of China should update the Healthy China Action Plan (2019–30) to address the health risks of climate change as soon as possible.
(4) Increase climate change mitigation. China's new pledges towards carbon neutrality by 2060 is a major step forward. Speeding up the coal phase-out process is therefore necessary to be consistent with the carbon neutrality pledges and continue China's progress on air pollution reduction. Fossil fuel subsidies should also be phased out to reflect the true cost of ongoing fossil fuel use and to avoid undermining the effect of China's emissions trading scheme, scheduled to take effect in 2021.
(5) Ensure the country's recovery from the COVID-19 pandemic protects health both now and in the future. Decisions made as part of China's efforts to recover from COVID-19 will shape the public's health for years to come. The longer-term prospects for lives, livelihoods, and a sustainable economy will be put in jeopardy if these interventions do not prioritise climate change.

## Introduction

Climate change threatens the health and wellbeing of populations in every country.^1^ China is particularly susceptible to these health effects, with large proportions of the population exposed to rising sea levels, climate-sensitive infectious diseases such as dengue fever, yellow fever, and chikungunya, and substantial increases in both wildfires and heatwaves.^2^

As the world's second largest economy, and the country with the largest population and total carbon dioxide (CO_2_) emissions, China is a key global stakeholder in the response to the health effects of climate change, with progress benefiting not only the health of the 1·4 billion people in China, but also the health of populations around the world. However, the interlinkage of public health and climate change has yet to receive full attention from the Chinese Government. For example, the recently adopted Healthy China Action Plan (2019–30), a public health agenda for the building of a comprehensive health system in China, contains no mention of climate change.^3^ With the Paris Agreement due for implementation and the deadline for the achieving the Sustainable Development Goals (SDGs) just a decade away, interventions taken in 2020 will be pivotal.^4^, ^5^, ^6^ At the same time, the world has been disrupted by a global pandemic, from which the effects will be felt for years to come. Moving forward, the development of national and international COVID-19 stimulus packages should align with the goals and principles of both the Paris Agreement and the SDGs, to ensure a sustainable recovery.

Tracking the progress on health and climate change at the national level in China will not only enhance understanding of these interlinkages, but also evaluate the adequacy of its response and highlight the benefit of aligning environmental and social policy. To deliver this, Tsinghua University has developed the inaugural *Lancet* Countdown report for China, in collaboration with University College London and 17 Chinese and international institutions. This paper serves as the first endeavour to track China's progress across all of the dimensions of health and climate change, mirroring the definition and grouping of indicators, and approaches and methods used by the global *Lancet* Countdown report. This work will be developed over time, with an iterative and adaptive approach that sees continual improvement in the indicators and methods considered. In its first year, the report presents 23 indicators across five domains: climate change effects, exposures, and vulnerability; adaptation planning and resilience for health; mitigation actions and health co-benefits; economics and finance; and public and political engagement (panel).

Panel **The China Lancet Countdown indicators**

**Climate change impacts, exposures, and vulnerability**

1.1: health and heat
  1.1.1: exposure of susceptible populations to heatwaves
  1.1.2: heatwave-related mortality
  1.1.3: change in labour capacity
1.2: health and extreme weather events
  1.2.1: wildfires
  1.2.2: cyclones
1.3: climate-sensitive infectious diseases

**Adaptation, planning, and resilience for health**

2.1: adaptation planning and assessment
2.2: adaptation delivery and implementation
  2.2.1: detection, preparedness, and response to health emergencies
  2.2.2: air-conditioning–benefits and harms

**Mitigation actions and health co-benefits**

3.1: energy system and health
3.2: clean household energy
3.3: air pollution, transport, and energy
3.4: sustainable and healthy transport

**Economics and finance**

4.1: health and economic costs of climate change and benefits from its mitigation
  4.1.1: costs of heat-related mortality
  4.1.2: economics cost of heat-related labour productivity loss
  4.1.3: economic costs of air pollution-related premature deaths
4.2: the economics of the transition to zero-carbon economies
  4.2.1: healthy energy investments
  4.2.2: employment in low-carbon and high-carbon industries
  4.2.3: fossil fuel subsidies
  4.2.4: coverage and strength of carbon pricing

**Public and political engagement**

5.1: media coverage of health and climate change
5.2: individual engagement in health and climate change
5.3: coverage of health and climate change in scientific journals

Where possible, the data sources and methods have been improved, to provide higher spatial resolution (including down to the provincial level) or information more relevant and appropriate within the context of China. This report presents the results for each of the 23 indicators, with a complete description of the methods, data, limitations, and future improvements provided for each indicator in the appendix 2.

## Section 1: climate change impacts, exposures, and vulnerability

Climate change interacts with each of the social and environmental determinants of good health, affecting lives and livelihoods through a myriad of different pathways.^1^ This section attempts to understand the interactions between climate change and health, tracking the ways in which climate change has influenced the health of Chinese people, through heat and heatwave (indicators 1.1.1–1.1.3), extreme weather events (indicators 1.2.1 and 1.2.2), and the climate-sensitive infectious diseases (indicator 1.3). Given China's unique vulnerabilities and long coastline in the tropics, an additional indicator tracking exposure to cyclones (indicator 1.2.2) has been included in this report.

### Indicator 1.1: health and heat

#### Exposure of susceptible populations to heatwaves (indicator 1·1.1): In 2019 there were an additional 2·20 billion person-days of heatwave exposure affecting people older than 65 years, the equivalent of every person in this age group experiencing 13 additional days of heatwave in a single year

Heat and heatwave exposure can often be fatal for older populations, owing to a range of factors, including a higher prevalence of chronic disease and medication use, and an impaired physiological and behavioural response.^7^ Against a baseline of warm seasons in 1986–2005, this indicator tracks the number of days that people aged older than 65 were exposed to a heatwave from 2000 to 2019, with the use of gridded temperature and population data.^8^, ^9^ For this indicator, a heatwave is defined as a period of 3 or more consecutive days where the daily maximum temperature was greater than the 92·5th percentile of the grid's distribution of the baseline daily maximum temperature in summer, a definition that best captures the health effects of heatwave events in China.^10^ A full description of the methods and data can be found in the appendix 2 (pp 3–5).

**Figure 1:**
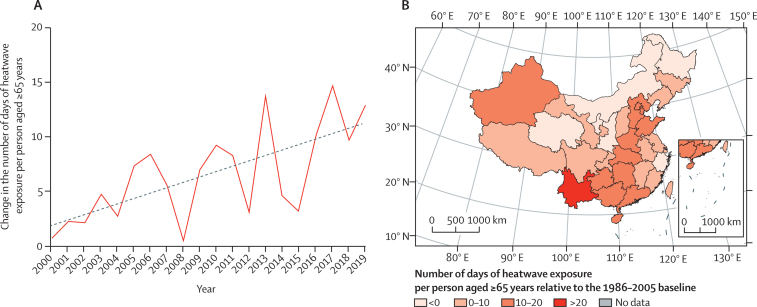
Change in the number of days of heatwave exposure for each person aged 65 years or older in China, relative to the 1986–2005 average. (A) Country-level trend. (B) Provincial-level results in 2019.

Nationally, heatwave exposure has been rising steadily, from an increase of 71·8 million person-days from the baseline in 2000 to a record high of 2·20 billion person-days in 2019, which is second only to 2·24 billion in 2017. The increase is equivalent to a person aged over 65 years enduring 13 more days of heatwave in 2019 than in 2000 (figure 1). The total amount of heatwave exposure is affected by both climate change and population ageing, whereas the change of heatwave exposure per person is only affected by climate change. Looking closer at 2019, each older person in Yunnan had 39 more heatwave days, the highest among all provinces, followed by Hong Kong (22 days), and Hainan (18 days).

#### Heatwave-related mortality (indicator 1·1.2): heatwaves caused an estimated 26 800 deaths in China in 2019, with the mortality rate rising by an additional 1000 deaths every 1·2 years over the past decade and with the greatest burden being in east and south-central China

In their most extreme form, heatwaves result in excess mortality, usually seen in the exacerbation of cardiovascular and respiratory disease.^11^ This indicator evaluates heatwave-related mortality across all age groups, with the use of gridded population and temperature data, the heatwave definition as described in indicator 1.1.1, and location-specific exposure–response curves, with methods described in the appendix 2 (pp 5–6).^8^, ^9^, ^10^, ^12^

**Figure 2:**
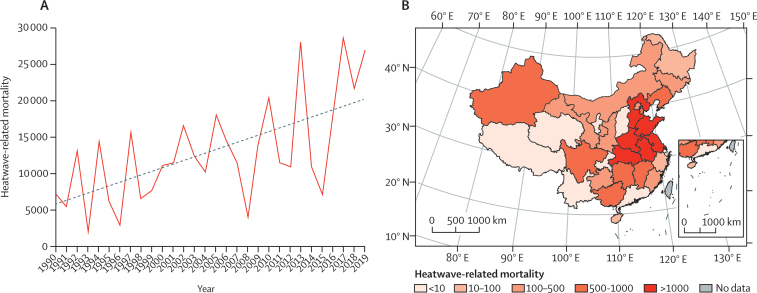
Heatwave-related mortality in China (A) Trend of heatwave-related mortality in 1990–2019. (B) Heatwave-related mortality in different provinces in 2019.

There were around 26 800 heatwave-related deaths in China in 2019, with the rising trend becoming increas-ingly apparent over recent years (figure 2). Taking a 5-year moving average, it took 3·8 years for every in-crease of 1000 annual heatwave-related deaths from 1990 to 2009, but just 1·2 years for the same increase to occur from 2010 to 2019. Among the provinces, heatwave-related mortality was highest in Shandong, followed by Henan and Anhui, all of which are located in east and south-central China.

Considering broader heat-related mortality in the future, there would be 27 900 additional heat-related deaths in Chinese cities per year over 2060–99 in a 2·0°C increase in average annual temperature scenario compared with a 1·5°C increase scenario,^13^ and in the 2080s, heat-related cardiovascular mortality in Beijing could increase by 69·0% in the Representative Concentration Pathway 4·5 scenario and 134·0% in the Representative Concentration Pathway 8·5 scenario.^14^

#### Change in labour capacity (indicator 1.1.3): Chinese workers had potential heat-related productivity losses of an estimated 9·9 billion h in 2019, 0·5% of the total national work hours for that year. Almost a quarter of these losses occurred in Guandong province

There is a clear consensus from biometeorological studies that increased heat stress from climate change will reduce labour productivity, leading to an increased socioeconomic burden.^2^, ^15^, ^16^ Indicator 1.1.3 focuses on this effect, calculating the work hour losses by the use of Wet Bulb Globe Temperature in primary industries (agriculture, forestry, animal husbandry, and fishery), secondary industries (manufacturing, construction, and mining), and tertiary industries (catering, finance, and other services), as described in the global *Lancet* Countdown report and in the appendix 2 (pp 6–9).^17^

In 2019, the potential total work hours lost in China were over 9·9 billion, 4·8% higher than in 2000 and representing 0·5% of the total national work hours. Labour capacity loss per worker in the primary industry worsened by 6·2% annually from 2000, reaching 36 hours (or 4·5 working days) in 2019. Nearly a quarter (2·4 billion h) of these potential losses occurred in Guangdong, the most populous and economically developed province, which accounts for 11% of China's gross domestic product (GDP). Each primary industry worker in Guandong potentially lost an average of 14·3 days and secondary industry workers potentially lost an average of 5·2 days, resulting in substantial losses to wages, productivity, and livelihoods.

### Indicator 1.2: health and extreme weather events

#### Wildfires (indicator 1.2.1): in 2016–19, 24 Chinese provinces saw an increase in the number of annual days of population exposure to wildfires compared with 2001–05. These increases were greatest in northern and northeastern China

Wildfire causes direct thermal injuries and death, as well as excess morbidity and mortality from smoke-related exacerbations of acute and chronic respiratory symptoms.^2^ In this report, change in the population exposure to wildfire is estimated by overlaying satellite data with population data and counting the number of days in which wildfires occur per grid cell, with densely populated urban areas excluded.^9^, ^18^ The annual mean days of exposure were calculated for four time periods: 2001–05, 2006–10, 2011–15, and 2016–19. Nationally, total exposure increased in the first three periods, and decreased in the last period, probably owing to increased urbanisation. However, at the provincial level, 24 of 34 provinces had an increase in annual person-days exposed to wildfire in 2016–19 compared with 2001–05. The largest increase was observed in northern and northeastern provinces, including Heilongjiang, Jilin, and Tianjin provinces, implying a need for strengthened wildfire monitoring and control.

#### Cyclones (indicator 1.2.2): China has had a substantial increase in the occurrence of severe typhoons from 2000 to 2019, compared with a stable baseline

China's extended eastern coastline is affected by tropical cyclones, which can cause injury and death, infectious diseases, and negative mental health effects.^19^, ^20^, ^21^, ^22^, ^23^, ^24^ Unique to the Chinese *Lancet* Countdown report, this indicator tracks cyclone exposure and damage, with the use of national data.^25^, ^26^ The tropical cyclones are described in terms of frequency, intensity, and spatial–temporal distribution at the provincial level. The trend in the occurrence of tropical cyclones for each intensity grading is calculated for 2000–19 compared with an extended 1980–99 baseline. Compared with the baseline, a statistically significant increase has been detected in the occurrence of severe and super typhoons from 2000 to 2019, whereas the occurrence of tropical depressions and tropical storms have decreased. Because of better adaptation interventions, the damages caused by tropical cyclones to hotspot provinces, such as Fujian and Zhejiang in east China, have significantly decreased over this period (appendix 2 pp 20–25).

### Indicator 1.3: climate-sensitive infectious diseases

#### Vectorial capacity for the transmission of dengue virus through Aedes aegypti has increased by 37% and through Aedes albopictus has increased by 14% since the 1960s

Dengue virus is a notable climate-sensitive infectious disease that is vector borne, with climate suitability for the transmission of this virus rising in every world region. This indicator focuses on the change in vectorial capacity of the *A aegypti* and *A albopictus* mosquitos to transmit dengue virus, which is expressed as the average number of daily cases resulting from one infected patient, and is influenced by daily temperature. The method for calculating vectorial capacity is the same as that described in the 2019 *Lancet* Countdown report and by Rocklöv and Tozan.^27^ Compared with 1961–65, the climate suitability for the transmission of dengue virus in 2014–18 has risen by 37% for *A aegypti* and 14% for *A albopictus*. In turn, there has been considerable and continuous national growth in both the incidence and disability-adjusted life-years lost in China. In 2017, the all-age incidence rate of dengue fever increased by 5·7 times and the disability-adjusted life-years rate of dengue fever increased by 4·7 times, compared with that of 1990, reaching 183·8 per 100 000 for the all-age incidence rate and 1·8 per 100 000 for the disability-adjusted life-years rate.^28^

Several other infectious diseases in China are climate sensitive, placing further risk to Chinese populations now and in the future. For example, the transmission potential of malaria will increase by 39–140% in south China, with an air temperature increase of 1–2°C.^29^

### Conclusion

**Figure 3:**
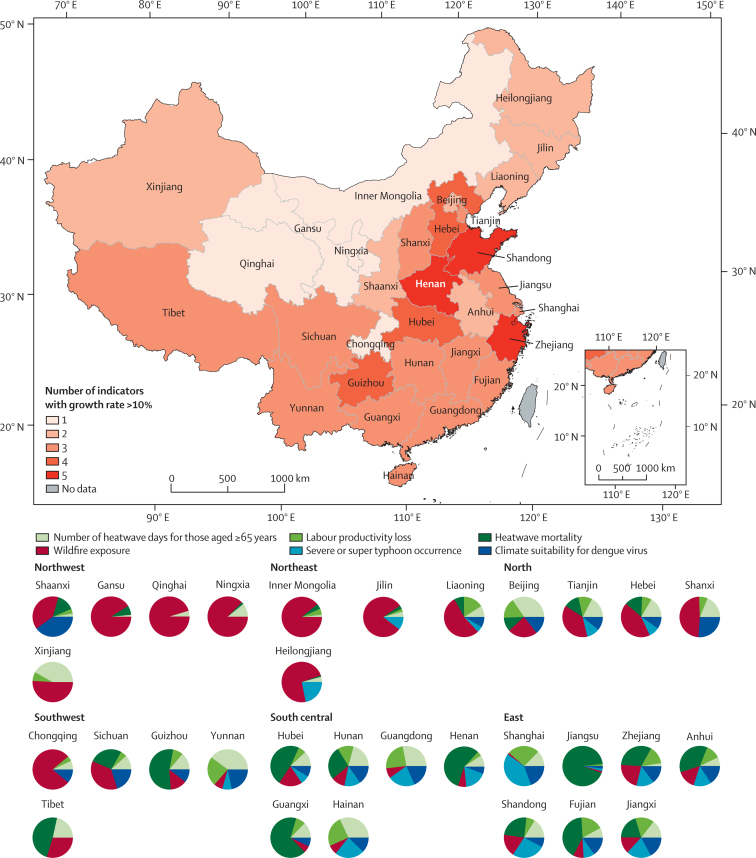
The key rising health risks from climate change in each province in China Each province in the map is coloured according to the number of indicators with a growth rate larger than 10% between 2000 and 2019. Each colour in the pie chart represents one of the indicators tracked. With each indicator weighted equally, the size of the slice is proportional to the size of the growth rate of each indicator.

Overall, this section provides clear evidence that the health effects of climate change are rising rapidly and affect different parts of China in unique ways. Different regions have their unique health threats and need a targeted response, with figure 3 providing a composite assessment of this diversity across the country. In three provinces, Henan, Shandong, and Zhejiang, five of six indicators reported here have worsened by at least 10% between 2000 and 2019. These three provinces alone account for around 20% of China's population and national GDP.^30^ Most of the highly populated and economically developed provinces in eastern and northern China have more than three indicators that have risen by at least 10%, implying that a large proportion of Chinese people and the economy are at risk. These findings provide a strong justification for more ambitious adaptation and mitigation inter-ventions to protect health, for which the indicators are tracked in later sections in this Health Policy paper.

The aforementioned findings are by no means an exhaustive list of the effects of climate change on human health in China—a greater resolution will be required across all provinces, and a number of core indicators will need to be further developed, including those focused on sea level rise, other extreme weather events, as well as climate-sensitive diseases, allergies, and mental health.^31^

## Section 2: adaptation, planning, and resilience for health

The health effects of climate change highlighted in section 1 require a concerted response from all sectors of society, which aims to reduce greenhouse gas emissions and adapt to the effects that are already present. The central government of China issued a National Plan in Response to Climate Change^32^ in 2007, which begun to recognise the health effects of climate change. At provincial and municipal levels, China is also increasingly developing climate change adaptation strategies for health. These measures mainly focus on the health effects of extreme weather events such as heatwaves, floods, and droughts; however, without the guidance of a national adaptation plan for health, China's ability to cope with climate change varies greatly from region to region. The second section draws on a selection of indicators from the global report across two domains: adaptation planning and assessment (indicators 2.1), and adaptation delivery and implementation (indicators 2.2.1 and 2.2.2). A third indicator domain, climate information services for health, is under development and is considered in the appendix 2 (pp 46–47).

### Indicator 2.1: adaptation planning and assessment

#### China has no standalone national adaptation plan for health; however, three provinces have a provincial plan in place in 2020, with a further four provinces under development. Six provinces have completed a comprehensive assessment of health and climate change effects and vulnerabilities

WHO identifies Health National Adaptation Plans,^33^ including the assessment of specific climate change effects and vulnerabilities, as being important first steps in managing country-specific health risks, with approximately 50 countries across the world having completed these two steps.^2^ Although China has a broad range of adaptation strategies in which health is referenced to some extent, there is no integrated assessment for health adaptation. For the purposes of this study, and to track China's efforts in adaptation planning and assessment at the provincial level, a Health and Climate Change Survey for China, which adapts the design of WHO's Health and Climate Change Survey,^34^ was done in May, 2020, led by Sun Yat-sen University (Guangzhou, China) and the Chinese Centre for Disease Control and Prevention (Beijing, China). The full details of this survey, as well as further data, analysis, and caveats are presented in the appendix 2 (pp 32–38).

Of the 17 provinces that completed the survey, three provinces (Guangdong, Shanghai, and Sichuan) declared that there was a provincial health and climate change plan in place, and a further four provinces indicated that plans were under development. Four provinces also stated their provincial health departments and meteorological departments were in close collaboration on health and climate change planning and strategy. The absence of a mechanism for multisectoral cooperation (all respondents), government funding (82%), and national surveillance systems (82%), were identified as the main constraints to developing climate change adaptation plans for health.

Turning from health adaptation planning to vulnerability assessments, six of the 17 provinces reported that a comprehensive assessment of climate change and health had been completed. Within these assessments, the effects of heatwave were among the most readily analysed health risk. Notably, human health has been included in the following: Climate and Environmental Evolution in China 2012;^35^ National Assessment of Climate Change in 2015;^36^ and China's annual reports on Actions to Address Climate Change (the Green Book Series) in 2014 and 2019.^37^, ^38^

Despite these provincial and national assessment findings, little progress has been made in influencing health policy making, and in allocating human and financial resources. As a matter of priority, China needs to strengthen its leadership and establish longer-term funding to ensure a comprehensive national adaptation plan that protects health against climate change.^39^

### Indicator 2.2: adaptation delivery and implementation

#### Detection, preparedness, and response to health emergencies—headline finding: clear regional differences were found in each province's ability to manage health emergencies. East China reported a higher ability index than other regions in China, and Jiangsu scored the highest, with an index score of 69·7 of 100·0 (indicator 2.2.1)

**Figure 4:**
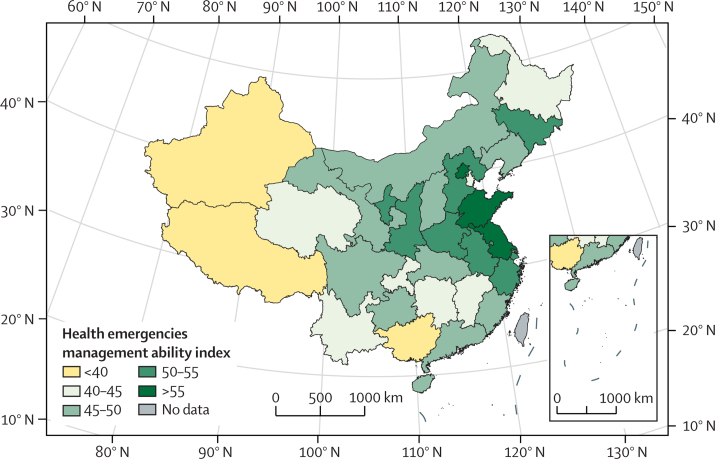
Comprehensive index measuring health emergencies management ability in different provinces in China

Climate change affects human health by disease transmission and climate-related extreme events, such as heatwaves, floods, cyclones, and wildfires. The ability to detect and rapidly respond to a health emergency is essential for minimising the effect of outbreaks of infectious diseases, as well as climate-related extreme events.^40^ Although similarities are seen in each of the health emergency management systems across China's provinces, there is great variation between them, with differing capacities to respond to the health effects of climate change. For this indicator, a comprehensive index system derived from Check-up for China's Cities^41^ was created, designed to be used by provincial governments and consisting of indicators of risk exposure and preparedness, detection, and response; and resource support and social participation. The index components include urban population density, completeness of emergency planning for public health emergencies, constructing of an infectious disease reporting system, and the number of health-care institutions and health practitioners per 1000 population. All indicator components, data sources and weightings are described in full in the appendix 2 (pp 38–44). Drawing primarily on the most recent data available from 2018, the average index score for health emergencies management across all provinces was 48·1 (of a possible 100·0). Results revealed that the indexes were generally higher in east China than in other regions, with Jiangsu (69·7), Shandong (68·9), and Beijing (60·9) scoring the highest in their ability to manage health emergencies (figure 4).

#### Air-conditioning, benefits and harms—headline finding: the use of air-conditioning provides protective benefits against heat-related mortality, and simultaneously substantially increasing energy consumption and CO_2_ emissions, with CO_2_ emissions rising by almost 1000% from 6·2 to 58·5 million tons per year from 2000 to 2016 (indicator 2.2.2)

Access to air-conditioning can protect people from heat-related morbidity and mortality,^42^ however, it also confers harm through its contribution to CO_2_ and particulate matter of 2·5 μm or less (PM_2·5_) emissions if its electricity source is from fossil fuels. Air-conditioning also emits waste heat, contributing to the urban heat island effect and can leak hydrofluorocarbons, which are powerful greenhouse gases.^43^ Therefore, other measures, focused on improving access to urban greenspace and building designs that improve energy efficiency and passive cooling, are also necessary. With the use of data from the International Energy Agency (IEA) and the relative risk described in the 2019 global *Lancet* Countdown report,^2^ this indicator calculates the prevented proportion of heatwave-related deaths caused by household air-conditioning use, with full methods and caveats presented in the appendix 2 (pp 45).

**Figure 5:**
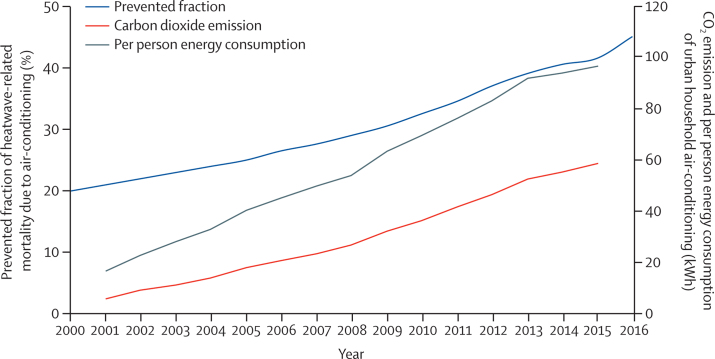
Prevented fraction of heatwave-related mortality caused by household air-conditioning use and energy consumption of urban household air-conditioning in China

As a result of increased household air-conditioning use, the prevented fraction of heatwave-related mortality caused by air conditioning in China doubled between 2000 and 2016, to 45% in 2016 (figure 5). However, the increasing use of air-conditioning also led to a concerning rise in energy consumption, CO_2_ emissions, and air pollution. Between 2001 and 2015, the per person energy consumption of Chinese urban household air-conditioning increased from 16·4 kWh to a remarkable 96·6 kWh, and air-conditioning-related CO_2_ emissions increased by almost a factor of ten, from 6·2 to 58·5 million tons per year (figure 5). These trends are deeply concerning and show the risks posed by the rapid adoption of technologies with substantial lock-in potential, thus worsening greenhouse gas emissions over the long term; and alternative heat adaptation measures, including improved energy efficiency in buildings, passive ventilation, and increased urban green space, should also be introduced to reduce the negative effect of air-conditioning use.^44^, ^45^

### Conclusion

There is a clear need for adaptation to the effects of climate change today, and those expected in the future. This section has considered both the broad and crosscutting importance of planning and assessment for all the health risks of climate change, as well as one specific adaptation intervention focused on the use of air-conditioning, with more to follow in subsequent years. These indicators suggest that China's current efforts are still in their initial stages, with varying degrees of progress seen across the provinces. Given the variation in geographical and socioeconomic context, as well as the climate change-related health risks faced, there is a need for more localised planning, as well as an integrated national assessment to reduce public health risks from climate change.

## Section 3: mitigation actions and health co-benefits

Robust and accelerated climate change mitigation will not only restrict the effects of temperature rise (section 1), but also lead to direct positive effects on human health. These improvements are found through a range of sectors of the economy, resulting in cleaner air, healthier diets, and physical activity. For example, measures such as the phasing out of small and outdated factories, and the promotion of clean fuels in the residential sector from 2013–17, was associated with 210 000 avoided premature deaths caused by air pollution in China in 2017.^46^ Further research increasingly supports these findings, suggesting that further reductions in fossil fuel combustion will further reduce air pollution, seeing substantial benefits for human health, and supporting China's transition towards sustainable development.^47^, ^48^, ^49^

Under the Paris Agreement, the Chinese Government has pledged to peak its emissions by 2030.^50^ However, global emissions have continued to rise in recent years, reaching 55·3 Gt of CO_2_ equivalent (CO_2_e) in 2018. Limiting the temperature rise well below 2°C will require further ambition both globally and within China.^51^ China has had rapid economic development over recent decades, whereas greenhouse gas emissions have increased at a slower rate, leading to a reduction in the carbon intensity of its economic system of 36% from 2000 to 2019.^52^, ^53^ In absolute terms, after a 5-year period of being nearly flat, China's greenhouse gas emissions have dropped from 11·6 Gt CO_2_e in 2017 to 10·3 Gt CO_2_e in 2018. After surging with an average annual growth rate of 10% since 2000, China's CO_2_ emissions peaked in 2013 (10·1 Gt CO_2_), decreased during 2014–16,^54^ rebounded onwards from 2017 with 9·7 Gt CO_2_, and increased to 9·9 Gt CO_2_ in 2018.^55^ COVID-19 has seen CO_2_ emissions in China drop substantially in the first quarter of 2020, with a 6·9% reduction when comparing January–April, 2019 with 2020,^55^ which led to a reduction in population-weighted PM_2·5_ of 14·5 μg/m^−3^ across China.^56^ Recovery measures that are consistent with the Paris Agreement, such as those that adjust the energy structure, increase the proportion of renewable energy, and improve energy efficiency, will ensure continued progress to reduce greenhouse gas emissions and improve health. This section tracks Chinese efforts to reduce greenhouse gas emissions, and the associated co-benefits by sector. For this inaugural report, four indicators are presented in total, tracking energy and health (indicator 3.1), clean household energy (indicator 3.2), air pollution (indicator 3.3), and sustainable and healthy transport (indicator 3.4). These will be further expanded on in subsequent years, with every effort made to include the additional sectors of the economy and more directly capture the attribution of these indicators to climate change and to health outcomes.

### Indicator 3.1: the energy system and health

#### The downward trend of coal consumption in China was reversed after 2016, with the total primary energy supply from coal rising to 80·2 EJ in 2018. Wind and solar power generation also continues to rise rapidly, and renewable energy represented 13·4% of China's total power generation in 2019

The energy system emits more CO_2_ than any other sector in China, and causes a substantial proportion of the burden of disease from air pollution. This indicator reports on key areas required to reduce the carbon intensity of China's energy system—the phasing out of coal and zero-carbon emission electricity—with the use of data from the Energy Statistical Yearbook of China.^52^

Driven by effective air pollution control,^57^, ^58^ the total primary energy supply from coal in China decreased from 2013 to 2016. However, this downward trend has been reversed in each subsequent year, in large part because of the launch of economy stimulus policies, with the total primary energy supply of coal increasing to 80·2 EJ in 2018.^30^ Given the early importance of a rapid phasing out of coal-fired power, and readily available technologies that are healthier and more cost-effective, this trend is particularly concerning.

More positively, the national share of low-carbon electricity continues to grow, rising from 17% in 2000 to 31% in 2019.^52^ Promisingly, in 2019, renewable energy (solar and wind power) accounted for 13·4% of total electricity generation, and within this, solar generation continues to grow at an unprecedented rate of 26·5% per year. China's newly installed capacity of solar and wind energy in 2019 reached 26·8 gigawatts (GW) for solar energy and 25·7 GW for wind energy, which is equivalent to adding one modern wind farm and one modern solar farm of 70 megawatts every day to its grids. To fulfil its National Determined Contribution to the Paris Agreement, China needs to have integrated stimulus packages in considering total primary energy supply and low-carbon electricity.

### Indicator 3.2: clean household energy

#### Further work is required to increase the use of clean and healthy energy within households. Fossil fuels accounted for over 74% of energy for cooking in urban households in 2018, whereas biomass still contributed 61·4% of total household energy in rural areas in 2013

The access and use of clean energy in China have rapidly increased since 2000, which is essential for social development and health and wellbeing.^59^ This indicator reports on household energy consumption with the use of data compiled by the National Bureau of Statistics, as well as fuel used for household hot water and cooking, with data collected by the Tsinghua University Building Energy Conservation Research Centre (Beijing, China). Per person household energy consumption has increased substantially, by 215% from 3·9 GJ in 2000 to 12·2 GJ in 2017, with electricity use as a share of total household energy consumption rising from 10% in 2000 to 20% in 2018. Meanwhile, household fossil fuel consumption made modest progress, but is high and accounts for over 74% of urban cooking energy in China. In rural households, biomass is the primary source of energy, accounting for 61·4% of household energy in 2013, increasing the health burden from household air pollution.^60^ Replacing gas-fired, coal-fired, and biomass cookers with electric cookers (and connecting to a grid powered by renewable energy) presents a notable opportunity for changing the household energy structure in China, reducing both household greenhouse gas emissions and air pollution.^61^

### Indicator 3.3: air pollution, transport, and energy

*Ambient PM_2·5_ pollution in urban China has decreased by nearly 28% from 2015 to 2019, leading to a reduction of 90* *000 premature deaths attributable to air pollution over this time period. However, 42% of the population is exposed to annual average PM_2·5_ econcentrations of more than 35 μg/m^3^*

As air pollution is the most important global environmental risk factor for premature mortality,^2^ China has adopted an ambitious response to deliver cleaner air. Here, PM_2·5_ concentrations in cities are presented, taking daily measured PM_2·5_ data from 367 cities as reported on by the Data Centre of the Ministry of Ecology and Environment of China.^62^ Premature mortality attributable to ambient PM_2·5_ by sector and region is also estimated, integrating data from the IEA and the China Energy Statistical Yearbook into the greenhouse gas–air pollution interactions and synergies (GAINS) model (as described in the global *Lancet* Countdown report) to estimate PM_2·5_ exposure and then mortality on the basis of integrated exposure–response functions.^63^ A full description of the datasets, methods, and projections is presented (appendix 2 pp 58–63).

**Figure 6:**
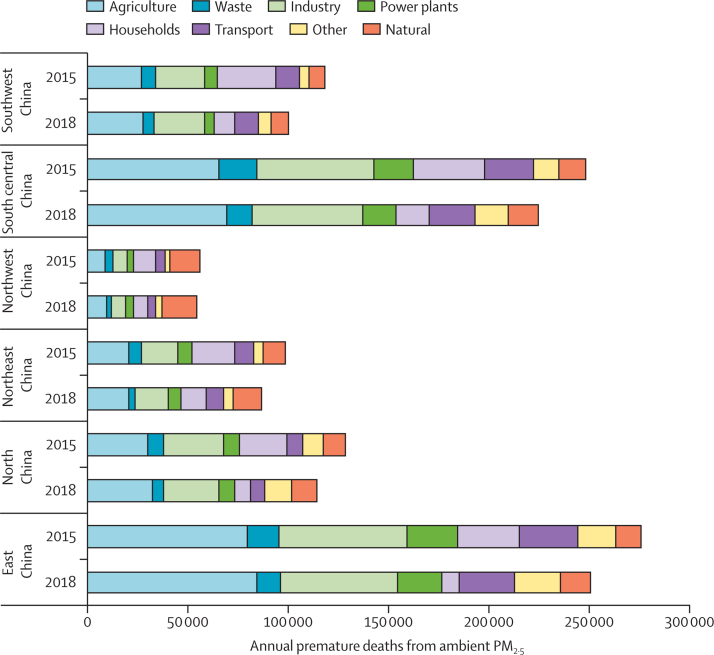
Premature deaths attributable to exposure to PM_2·5_ in 2015 and 2018, by key sources of pollution in China PM_2·5_=particulate matter with an aerodynamic diameter of 2·5 μm or less.

The implementation of China's clean air policy (eg, the Air Pollution Prevention and Control Action Plan) has seen 367 cities reduce air pollution by almost 28% on average from 2015 to 2019.^57^, ^58^, ^64^ Correspondingly, the deaths attributable to ambient PM_2·5_ pollution had declined by 10% from 2015 to 2018, with 830 000 deaths in 2018. However, approximately 42% of the Chinese total population were exposed to air pollution concentrations above WHO's first interim air quality target (35 μg/m^3^ of annual mean PM_2·5_ concentration, the least ambitious of the three interim targets presented) in 2018.^65^, ^66^ Industrial and agricultural sectors caused 53% of total ambient premature deaths in 2018, followed by transport sectors (10%), and residential sectors (8%; figure 6). At the regional level, east China has the highest premature deaths, where agriculture and industry sectors have a dominant contribution to PM_2·5_, followed by south-central China. With the exception of a few cities in Tibet,^62^ all Chinese cities continued to have PM_2·5_ concentrations above the WHO recommended annual average of 10 μg/m^3^. China has an opportunity to continue to reduce its population exposure to air pollution over the coming years, by aligning its economic recovery from COVID-19 with its air pollution policies, and the priorities of the Paris Agreement and SDGs. However, if the recovery measures implicitly increase the use of fossil fuels in China, given the exacerbating effects of future climate change to pollutant accumulation, these short-term improvements made to reduce air pollution in China will probably be reversed.^67^, ^68^, ^69^

### Indicator 3.4: sustainable and healthy transport

*The emissions intensity of road transport in China, represented by average emissions per vehicle, has dropped from 2000 to 2018 by over 90% for four major air pollutants*

**Figure 7:**
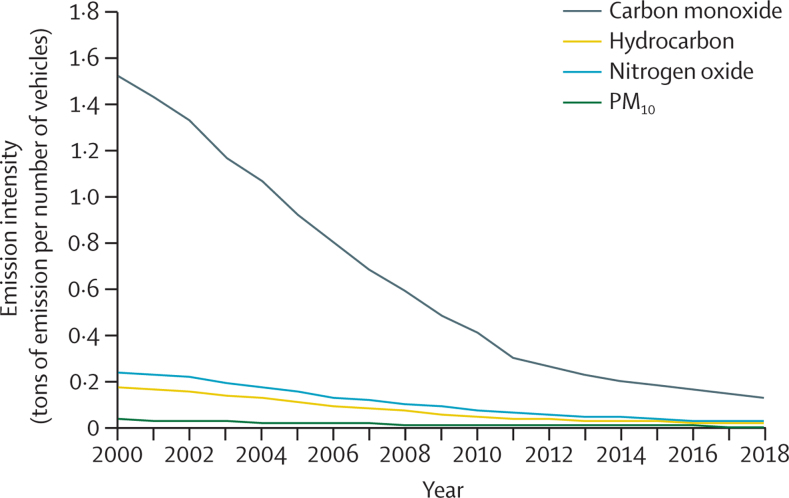
Air pollution emissions intensity of road transport in China from 2000 to 2018 for carbon monoxide, hydrocarbons, nitrogen oxide, and PM_10_ PM_10_=particulate matter with an aerodynamic diameter of 10 μm or less.

As well as emitting large amounts of CO_2_, fossil fuel combustion for road transport emits other harmful pollutants, including hydrocarbon, carbon monoxide, nitrogen oxide, and PM_10_, threatening public health, particularly in densely populated areas of China. In this indicator, the emission intensity of road transport, calculated by the ratio of emission and vehicle ownership, establishes the average emission for the whole fleet, including electric, hybrid, and natural gas vehicles. Data are taken from the China Vehicle Environmental Management Annual Reports and the National Bureau of Statistics of China.^30^, ^64^ The emission intensity for carbon monoxide has decreased by 92%; for hydrocarbon, by 91%; for nitrogen oxide, by 91%; and for PM_10_, by 94%, from 2000 to 2018 in China, reflecting the effective emissions control of road transport (figure 7). Emission per vehicle in 2000–18 has reduced from 0·24 to 0·02 tons per vehicle for nitrogen oxide and from 0·030 to 0·002 tons per vehicle for PM_10_. Between 2010 and 2018, the emission intensity in Beijing decreased by 42%; in Shanghai, it decreased by 44%; and Guangdong it decreased by 71%. The upgrade of emissions standards has played a notable part in this decrease, alongside ongoing modal shift.^70^ The number of electric vehicles reached 3·1 million in China in 2019, growing on average by 600 000 annually from 2014. This amount of growth is impressive, compared with the average annual growth of 260 000 between 2014 and 2019 in the USA (the next biggest market for electric vehicles).^71^ Again, in recent months, the response to COVID-19 has resulted in substantial reductions in transport activities and improved air quality for many cities.^56^, ^72^, ^73^ However, this response has also led to several unexpected haze events in northeast China because of the enhanced atmospheric oxidising capacity, caused by the imbalanced emission abatement of nitrogen oxide and volatile organic compounds.^74^, ^75^

### Conclusion

The indicators in this section highlight both the impressive progress China has made in mitigating climate change, as well as the health benefits it has had as a result, with substantial reductions in air pollution seen in recent years. However, these indicators also make clear that there is little room for complacency, with further effort required to be consistent with the Paris Agreement's long-term target. Coal is the predominant source of fuel for power generation, as well as a major contributor to air pollution and ill health, and is a clear target for early and rapid phase-out.

## Section 4: economics and finance

This section tracks the economics of climate change, and the financial and economic implications of addressing climate change in China. Mounting evidence suggests that the health cost of inaction on climate change is high,^76^ and that the health benefits of climate change mitigation alone could far exceed the mitigation cost in many other sectors and in many regions in China.^77^, ^78^ The financial case is clear, with cost-effective interventions available to allow increased ambition to address climate change for health, in China. The seven indicators in this section are divided into two domains: first the economic effect of climate change and its mitigation (indicator 4.1); and second the economics of the transition to zero-carbon economies, including investments in a low-carbon economy, as well as pricing greenhouse gas emissions from fossil fuels (indicator 4.2).

### Indicator 4.1: health and economic costs of climate change and benefits from mitigation

#### Costs of heat-related mortality—headline finding: the economic cost of heat-related mortality in China reached to US$13·6 billion in 2019 (indicator 4.1.1)

**Figure 8:**
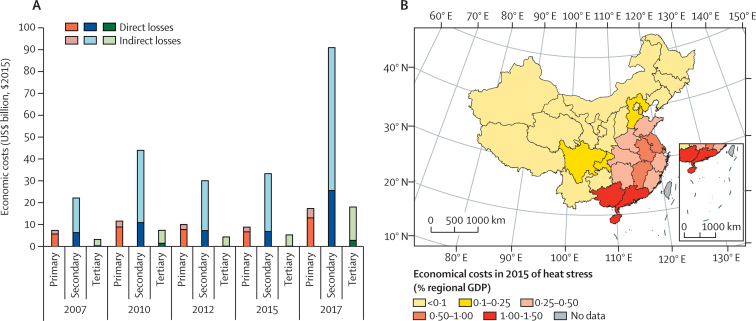
Monetised value of heat-related mortality, by region in China $2015=based on the value of the US$ in 2015.

This indicator tracks the monetised value of heatwave-related mortality by province in China, with the use of a value of a statistical life for China of $3·2 million (the value of the US dollar in 2015), based on the mean value of a statistical life of previous studies.^79^ At a country level, the annual cost of heat-related mortality increased from $1·0 billion to $13·6 billion from 1993 to 2019 (figure 8). This value is equivalent to the income of more than 1·3 million people in China in 2019. These costs have been the greatest in east China, reaching $6·1 billion in 2018 and 0·11% of the regional GDP (led by Shandong at $5·0 billion and 0·38% of regional GDP).

#### Economic costs of heat-related labour productivity loss—headline finding: the economic costs of heat-related labour capacity loss reached $126 billion (1·14% of China's GDP) in 2017, with the highest losses as a proportion of provincial GDP in Guangdong, Hainan, and Guangxi (indicator 4.1.2)

**Figure 9:**
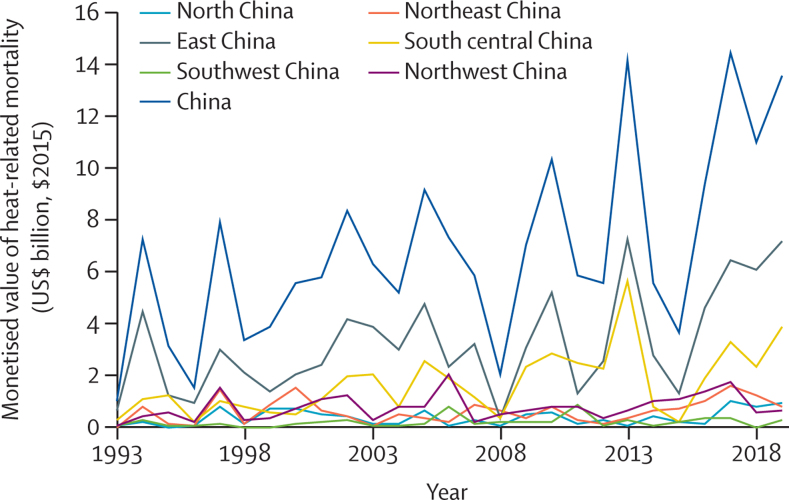
Economic costs of heat-related labour productivity loss. (A) National-level results, by year and industry, in $US billions of $2015. (B) Provincial-level results in 2015, relative to GDP. $2015=based on the value of the US$ in 2015. GDP=gross domestic product.

This indicator measures the total annual economic costs of heat-related labour capacity losses (indicator 1.1.3) and estimates direct losses (resulting from first-order losses of labour capacity in a particular industry) and indirect losses (higher-order losses in other industries that have dependencies on industries that have had direct losses) under an input–output analytical framework.^80^, ^81^, ^82^ Absolute economic costs of labour productivity loss in 2017 were $126 billion (1·14% of GDP), nearly four times the costs in 2007 and equivalent to the scale of national fiscal expenditure on science and technology or on environmental protection (figure 9A). In 2015, nearly 70% of the total costs were indirect costs, which were concentrated largely in the secondary industry (manufacturing, construction, utilities, and mining). The regional distribution of economic costs (in terms of shares in regional GDP) is consistent with China's geographical climate patterns, with south-central China suffering higher costs than other regions (figure 9B). The top three provinces with the greatest costs in 2015 were Guangdong (1·65% of GDP), Hainan (1·41%), and Guangxi (1·22%), each of which are southern provinces with warm and humid climates.

#### Economic costs of air pollution-related premature deaths—headline finding: the economic costs of premature deaths caused by ambient PM_2·5_ pollution have fallen over time in China, however still make up the equivalent of 0·09% of China's GDP in 2018 (indicator 4.1.3)

As reported in indicator 3.3, ambient air pollution (PM_2·5_) continues to be a substantial contributor to mortality in China. Diverging from the global *Lancet* Countdown report, this indicator estimates the economic costs of this premature mortality by considering the annual labour productivity loss that it results in, with the use of an input–output model as described in indicator 4.1.2 and in the appendix 2 (pp 72–75). This model does not fully or adequately capture the economic costs of air pollution-related mortality, which will need to be developed into future forms of this indicator.

**Figure 10:**
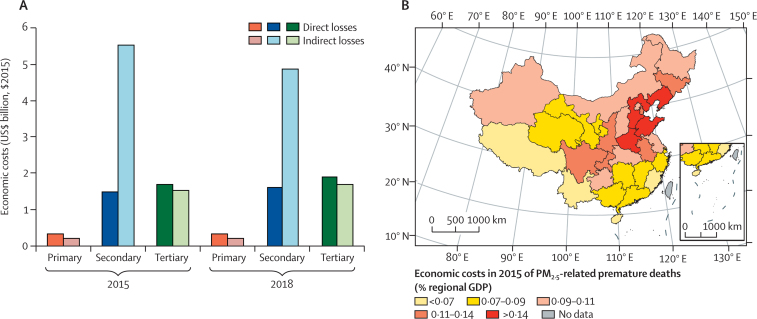
Economic costs of premature deaths from ambient PM_2·5_ pollution. (A) National-level result, by year and industry, in $US billions of $2015. (B) Provincial-level result in 2015, relative to GDP. $2015=based on the value of the US$ in 2015. GDP=gross domestic product. PM_2·5_=particulate matter with an aerodynamic diameter of 2·5 μm or less.

Figure 10 makes these changes over time clear, reflecting the air pollution (PM_2·5_-related) mortality seen above. The annual economic costs that resulted from premature mortality caused by ambient PM_2·5_ pollution decreased by 1·1% from $10·8 billion (2015) to $10·7 billion (2018). In 2018, these costs represented approximately 0·09% of China's GDP, a value that potentially rises to 1% of GDP when morbidity is also considered.^81^ In 2018, the indirect costs resulting from interindustrial dependencies were 64% of the total costs, mostly in the secondary industry. Provinces surrounding Beijing suffered the greatest costs (in terms of shares in regional GDP) from PM_2·5_-related premature deaths, as a consequence of the rapid development of energy-intensive and high-pollution industries in these provinces.

### Indicator 4.2: the economics of the transition to zero-carbon economies

#### Healthy energy investments—headline finding: China's investment in new coal-fired electricity capacity continued to decline again in 2019, continuing the downward trend observed since 2015. Low-carbon investments are now nine times higher than those of coal, with renewable investments reaching $86·4 billion in 2019 (indicator 4.2.1)

In parallel to indicator 3.1, this indicator tracks the financial aspects of mitigation in the energy system, considering investments in coal, low-carbon energy, and energy efficiency, taking data from the National Energy Administration.^83^ The investment in new coal-fired power generation in China declined from $31·7 billion in 2008 to $9·3 billion in 2019.^83^ Correspondingly, the ratio between investment in low-carbon energy (including hydropower and nuclear power) and new coal power has risen sharply, from 1:1 in 2008 to 9:1 in 2019. Investment in renewable energy reached $86·4 billion in 2019, largely because of investments in solar photovoltaics, which peaked at $76·1 billion in 2017 and then declined in the following 2 years.^84^ Investment in wind also increased to $17·3 billion in 2019.^85^ To support expanded renewable energy development, investment in the overall power grid itself also continues to be high: $81·5 billion in 2018 and $71·6 billion in 2019.

#### Employment in low-carbon and high-carbon industries—headline finding: for the second consecutive year, the renewable energy sector has employed more people in China than fossil fuel extraction industries, providing 4·1 million jobs in 2018 (indicator 4.2.2)

China leads employment in renewable energy worldwide, with 4·1 million jobs in 2018, and has seen a steady rise since 2012 (with employment falling slightly, by 2·7% from 2017 to 2018). For the second year running, this number has been higher than employment in fossil fuel extraction industries, which decreased by 6% from 2017 to 3·88 million in 2018. In 2018, the solar energy industry provided 2·9 million jobs in China, contributing to 70·1% of total jobs in the renewable energy sector. Although China represents 18·5% of the world's population, it now provides 37% of the world's renewable energy jobs. The data for this indicator are taken from the REN21 Renewables 2019 Global Status Report, Computer and Enterprise Investigations Conference Data, and the Chinese National Bureau of Statistics.^30^, ^86^, ^87^

#### Fossil fuel subsidies—headline finding: downward trends in fossil fuel consumption subsidies have reversed in recent years and were $41·9 billion in 2018, more than 10% higher than 2017 subsidies, and more than 100% higher than the 2015 amount (indicator 4.2.3)

Fossil fuel consumption subsidies distort the prices in the energy market. These subsidies wrongly provide fossil fuels with a competitive advantage over clean energy and neglect the negative externalities and costs to the environment, climate, and human health. This indicator tracks the absolute value of fossil fuel consumption subsidies in China, as well as China's share of total global subsidies, with the use of data from the IEA.^88^ Fossil fuel subsidies declined between 2011 and 2015, but rebounded to $41·9 billion in 2018—a 10% rise from 2017 and more than 100% higher than 2015 amount. This reversal is the result of substantial increases in subsidies for coal power plants, whose profitability has deteriorated severely over the past decade. Of all the countries with fossil fuel subsidies reported by IEA, China ranked third in 2018, behind only Iran and Saudi Arabia, two of the world's major oil producers.

#### Coverage and strength of carbon pricing—headline finding: pilot carbon pricing instruments already cover 11% of China's emissions in 2019; however prices are much less than what is consistent with the Paris Agreement (indicator 4.2.4)

An effective price on carbon is an important policy tool to incentivise and guide the transition to a low-carbon economy, and meet the goals of the Paris Agreement.^89^ This indicator tracks the coverage and strength of carbon pricing in China, with the data and methods described in the appendix 2 (pp 82–84).^90^, ^91^ The carbon prices in eight pilot carbon emission trading markets in China varied from $0·24 per total (t)CO_2_ to $13·0 per tCO_2_ over the last 6 years. The real-time carbon prices depend on the supply and demand of the carbon quota in each pilot market.^91^ In 2019, the annual average carbon prices ranged from $11·4 per tCO_2_ in Beijing to $0·56 per tCO_2_ in Chongqing and Shenzhen. However, these prices continue to be much lower than the price of $40–80 per tCO_2_ by 2020 (the price expected to be at by 2020 by the Paris Agreement), which is required to be consistent with the well below 2°C goal.^92^ The carbon emissions covered by eight pilot markets are 1330 metric tons of CO_2_e in 2019, representing 11% of China's total emissions and 53% of emissions in these provinces. With the introduction of China's national emissions trading scheme in 2021, the total coverage of carbon pricing would increase to 33% of China's emissions. All the pilot markets in China are still in their early stage, and do not generate regular revenues.

### Conclusion

In line with air pollution reduction efforts, the economic costs associated with its lost labour productivity have declined slightly over recent years. However, elsewhere, the economic costs of the increasingly worsening heat and heatwaves are rising, both in the form of increased mortality and decreased labour capacity. Although China has already enacted a range of policies to transition to a low-carbon economy, a more decisive intervention is required to phase out fossil fuel subsidies and enhance the carbon price signals. Without this intervention, the risk of short-term investment decisions that result in a commitment to the use of fossil fuel energy systems for the long term is high.

## Section 5: public and political engagement

Although the health effects of climate change are already being felt in China, responses have been insufficient at both the national and provincial level. In some instances, increased public engagement in health and climate change by a range of stakeholders has exerted pressure on governments, driving enhanced mitigation and adaptation efforts.^5^, ^93^ This section tracks engagement with health and climate change from the media (indicator 5.1), individuals (indicator 5.2), and academia (indicator 5.3). The engagement in health and climate change of the Chinese Government will be explored in next year's report.

### Indicator 5.1: media coverage of health and climate change

#### Media engagement in health and climate change is low in China, with 67 posts across five key media accounts in 2019

This indicator covers social media accounts exclusively and adopts a novel approach to assessing the engagement of social media in China with health and climate change. As of March, 2020, the number of internet users in China reached 904 million.^94^ The active monthly users of the social media platform Weibo reached 516 million and daily active users reached 222 million at the end of 2019.^95^ Five accounts (People's Daily, The Beijing News, Caixin, Health News, and China Science Daily) were selected and analysed on the basis of their size, influence, and variety (aiming to procure a range of official, commercial, and professional media). Data were drawn from posts published from these accounts to Weibo and analysed with the use of keywords as described in the global *Lancet* Countdown report, and a full description of the methods, data, and search terms are included in the appendix 2 (pp 84–86).

**Figure 11:**
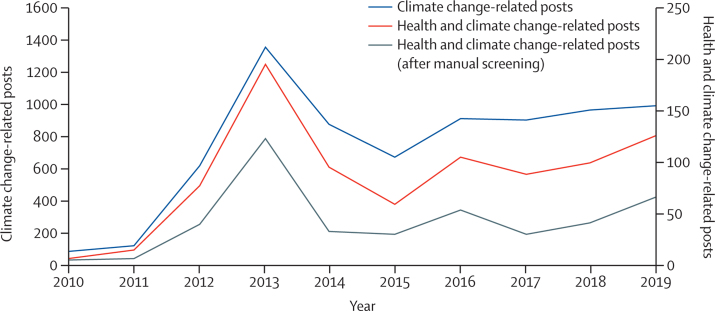
Annual coverage of climate change and health and climate change on Weibo between 2010 and 2019

From 2010 to 2019, there were 7526 posts in total discussing climate change across these five media accounts on Weibo. The annual coverage increased by 11 times, from 87 posts in 2010 to 997 posts in 2019 (figure 11), equivalent to 2·7 posts per day and 2% of the total number of daily posts by these five media accounts across all topics. Within these, the proportion that then referred to public health was low, at 5·7% in 2010, rising slightly to 6·7% in 2019. A spike seen in 2013 was related to increased awareness of air pollution and its links with health and climate change, corresponding with the initiation of the Air Pollution Prevention and Control Action Plan.^96^, ^97^ With only 67 health and climate change posts across all five accounts in 2019, and zero posts from Health News since 2014, it is clear that far more needs to be done to make the links between public health and climate change in social media.

### Indicator 5.2: individual engagement in health and climate change

*More work is required to engage individuals on the links between these two issues, with few people making the connection between public health and climate change on Baidu*

Individuals' search preferences provide insight into the degree of public engagement on a topic.^98^ This indicator provides an analysis of search queries for health and climate change over the past 3 years, identified by keyword matching on Baidu search engine.^99^ Baidu has taken up more than 66% of the of market share of search engines in China over the past decade.^100^ This site also has more than 1·1 billion monthly active users, covering most of China's population.^101^ Because of the widespread use of Baidu, the query data from Baidu can well reflect the individual engagement of people in China. Compared with other methods, such as surveys, query data from Baidu are easy to collect and have a broad coverage of people. All data are anonymised and no queries can be associated with an individual. A full description of the keywords used, and a full explanation of the methods and analysis are shown in the appendix 2 (pp 86–91).

A clear increase is seen in search queries for climate change from 2017 to 2019; however queries relating to health and climate change were seldom co-searched by users. In 2019, one in 500 of all climate change queries had some relation to health. Conversely, only one in 200 000 of all health queries had any relation to climate change. Considering the Chinese city tier system based on population and economic development, further analysis suggests that co-searching for health and climate change increased somewhat for tier 1 and 2 cities compared with the overall Chinese population.

### Indicator 5.3: coverage of health and climate change in scientific journals

#### A total of 15 articles in Chinese and 30 articles in English were published by Chinese authors in 2019 compared with 12 articles in Chinese and three articles in English in 2008

This indicator tracks engagement in health and climate change by Chinese researchers in scientific journals. The inclusion of climate-related keywords and their co-occurrence with health-related keywords in scientific publications was tracked with the use of the advanced search function in the China National Knowledge Infrastructure site for Chinese articles and in both Ovid MEDLINE and Ovid Embase databases for articles in English published by Chinese authors. Full details of these search strategies are provided in the appendix 2 (pp 92–101). In the China National Knowledge Infrastructure site, a total of 26 849 climate change-related academic journal articles between 2008 and 2019 were collected, of which 0·83% (222) were related to health. From 2010 to 2019, articles focused on health and climate change decreased slightly, from a high point of 29 in 2018 to 15 in 2019.

A different picture is seen when searching articles in English with Chinese authors in Ovid Embase and Ovid MEDLINE database, with 932 climate-related publications recorded between 2008 and 2019, and 17·7% (165) focusing on health and climate change. Between 2008 and 2019, the number of health and climate change articles written in Chinese that these databases recorded increased from three articles published in 2008 to 30 in 2019, although this still represents a small proportion of the 734 health and climate change articles published globally in 2019. Despite slow progress, awareness in the local scientific community is rising and it is expected to grow over time.^102^

### Conclusion

Engagement in health and climate change by all sectors is an essential component of initial and sustained mitigation and adaptation efforts. In China, the quantity of health and climate change engagement from the media, individuals, and academia has remained stubbornly flat, with small increases seen in certain sectors. These trends are at odds with those seen internationally and the commitments of the Paris Agreement which requires an expanded understanding of the links between social and environmental systems, in the media, in academia, and the general public.

## Conclusions of the 2020 China report of the *Lancet* Countdown on health and climate change

This inaugural China report of the *Lancet* Countdown tracks 23 indicators of health and climate change, at the national, regional, and provincial level. The findings represent the work of 77 experts from 19 institutions, and the report is the first complete picture of the health effects of climate change, and the responses undertaken in China. The indicators here will be continually improved on, with new concepts and datasets built in on an annual basis. To this end, the collaboration is committed to an iterative approach, and is open to input from technical experts and academic institutions across China.

The health risks from climate change tracked here (including heat, extreme weather events, and climate-sensitive diseases) are increasing rapidly in China. Although everyone is affected, susceptible populations, including those living in poverty, with social disadvantages, older people (≥65 years old), and those working outdoors, are particularly at risk. In 2019, every older person endured the equivalent of an average of 13 more days of heatwave, implying substantial health risks. In the same year, across all age groups, heatwave-related mortality reached 26 800, with the resulting economic loss estimated to be $13·6 billion.

Progress in adaptation and mitigation has been mixed. On the one hand, there is no standalone National Health Adaptation Plan and few provincial-level assessments of climate change and health risks have been done. Similarly, previous reductions in coal use and fossil fuel subsidies have been reversed. Slow progress across all sectors is in part because of the limited engagement in health and climate change within the media, academia, the public, and the government.

On the other hand, China has made remarkable progress in renewable energy development and air pollution control. 90% of investment in new power generation now goes to non-fossil fuel energy. Employment in renewable energy industries in China is higher than in any other country and accounts for a third of the global total. Air pollution continues its downward trend in China, leading to a 10% decrease in PM_2·5_-related mortality and $400 million saved. A national emissions trading scheme that covers 33% of China's emissions is being set up and is expected to enter into force in 2021.

**Figure 12:**
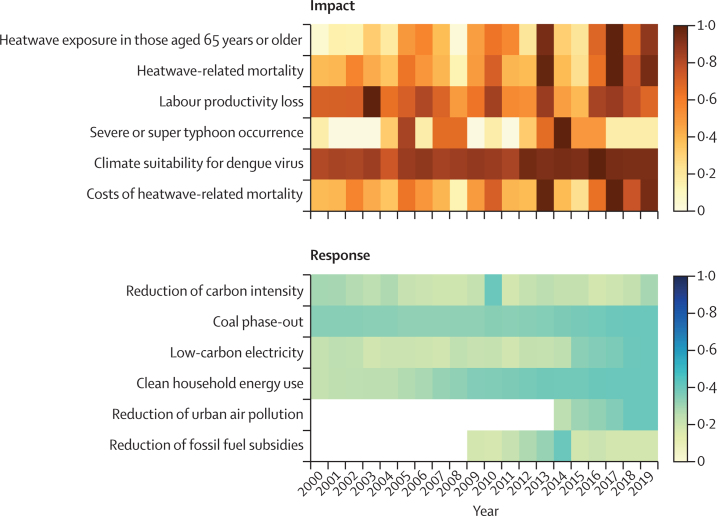
An overview of indicator trends For each indicator, an index is created, ranging from 0 to 1, with the colour in each block representing its score. Darker colours indicate a more concerning effect, and a more ambitious response. For example, index score 1 represents the worst case in the past 20 years for impact factors and the best possible case for response factors.

Figure 12 provides an overview of these risks and responses over time, exploring the health effects of climate change (upper panel) and China's responses to climate change for health (lower panel) with the use of a selection of the indicators described in this report, for which there is the most temporal data and the most clearly defined scale. For the response indicators presented, such as coal phase-out, clean household energy use, and the reduction of fossil fuel subsidies, an index score of 1 means the best possible response, for example, a 100% coal phase-out. Most of these indicators are far from 1, highlighting the space for substantial improvement. A comprehensive explanation for each indicator displayed in the figure can be found in the appendix 2 (pp 102–103) and this figure will be updated with each subsequent report, including additional indicators with scales defined on the basis of the best available evidence.

Immediate and ambitious responses to climate change will save lives in China. Conversely, delay and hesitation will impede the realisation of the Healthy China goals,^103^ affecting the health and wellbeing of 1·4 billion Chinese people. Although the COVID-19 pandemic has led to a range of reflections, reforms, and changes to social and health policy, these discussions have yet to include a serious consideration of the links between climate change and public health. Without such consideration, there is a risk that the recovery plans of one health crisis—that of COVID-19—will exacerbate the long-term risks of another health crisis—that of climate change.

For more on **China's emissions trading scheme** see https://www.iea.org/reports/chinas-emissions-trading-scheme

**This online publication has been corrected. The corrected version first appeared at thelancet.com/public-health on January 8, 2021**

## Supplementary Materials

Chinese translation of the full text
