# Correction to *Lancet Public Health* 2021; 6: e64–81

**DOI:** 10.1016/S2468-2667(21)00003-7

**Published:** 2021-01-08

*Cai W, Zhang C, Suen HP, et al. The 2020 China report of the Lancet Countdown on health and climate change.* Lancet Public Health *2021;* 6: *e64–8*—In figure 6 of this Health Policy paper, the years ‘2015' and ‘2018' on the y-axis were the incorrect way around. This correction has been made as of Jan 8, 2021.

