# Supplementary material for: The 2020 China report of the Lancet Countdown on health and climate change
Source: Lancet Public Health. 2020 Dec 2;6(1):e64–81. doi: 10.1016/S2468-2667(20)30256-5 (PMC7966675; doi:10.1016/S2468-2667(20)30256-5)
Supplement: Chinese translation of the full text [file mmc1.pdf]

THE LANCET
Public Health

Supplementary appendix 1

This translation in Chinese was submitted by the authors and we reproduce it as supplied. It has not been peer reviewed. *The Lancet*'s editorial processes have only been applied to the original in English, which should serve as reference for this manuscript.

Supplement to: Cai W, Zhang C, Suen HP, et al. The 2020 China report of the *Lancet* Countdown on health and climate change. *Lancet Public Health* 2020; published online Dec 2. https://doi.org/10.1016/S2468-2667(20)30256-5.

此简体中文译文由作者提交，我方按照提供的版本刊登。此译文并未经过同行审阅。医学期刊《柳叶刀》的编辑流程仅适用于英文原稿，英文原稿应作为此手稿的参考。

中国版柳叶刀倒计时人群健康与气候变化报告（2020 年）

执行摘要

如果气候变化的趋势得不到控制，它将对全球的人群健康造成巨大的威胁，因此迫切需要各国密切协作，积极应对气候变化。中国拥有全球五分之一的人口，也是世界上最大的二氧化碳排放国，在中国开展气候行动对人群健康和全球变暖的趋势都至关重要。和其他国家一样，减缓和适应气候变化将极大地造福于中国 14 亿人的健康状况，而将这些考虑纳入新冠疫情下的经济复苏计划和 2060 年碳中和目标的具体实现路径之中，将确保中国人当今和未来的福祉得到改善。未来数月乃至数年做出的决策将决定后续数十年气候政策的进程。

为应对这一挑战，清华大学联合英国伦敦大学学院和另外 17 所国内和国际机构，在《柳叶刀倒计时：跟踪健康和气候变化进展》（*Lancet Countdown: Tracking Progress on Health and Climate Change*）全球报告工作的基础上，聚焦国内情况，共同发布了针对中国的柳叶刀倒计时报告。本报告借鉴了国际方法和框架，旨在更全面地了解和跟踪中国国内人群健康与气候变化之间的联系。本报告是柳叶刀倒计时为深入了解世界不同区域的健康与气候变化联系所做出的尝试。相比全球报告而言，本报告的特点在于尽可能地使用省级数据、展示省级结果，以便地方决策者制定有针对性的应对策略。

气候变化对健康的影响以及中国的应对措施

本报告通过 23 项指标的研究得出了两个关键结论：

1.在中国，气候变化对健康的影响正在加快，如果全球气温继续升高，会带来令人难以接受的高健康风险。每个省份都受到了影响，但都面临着独特的健康威胁，因此需要制定有针对性的应对策略。

中国已经感受到了气候变化的影响，表现为气温升高、极端天气事件增加和媒介生态的改变。在过去 20 年，热浪相关死亡人数上升了 4 倍，2019 年的死亡人数达到了 2.68 万人，其货币化成本相当于中国 140 万人的年均国民收入。与 1986-2005 年基准水平相比，2019 年的热浪天数平均增加了 13 天，而老年人在热浪天死亡的风险会上升 10.4%。户外工作者的高温相关潜在劳动生产力损失达到了全国总工时的 0.5%，占中国国内生产总值（GDP）的 1%，相当于中国每年在科技方面的财政支出。受气温上升和气候变化驱动，极端森林火灾事件的增加以及登革热的传播将进一步造成严重的健康影响。

不同地区面临着不同的健康威胁，需要采取有针对性的应对措施——近二十年来，在 19 个省份报告的六项健康影响指标中，有三项或以上都提高了至少 10%。重要的是，一些人口密集、经济发达的省份，比如河南、山东和浙江，都面临着比其他省份更高、进展更迅猛的健康风险。

2.中国在应对气候变化的多个方面都取得了巨大的进步，但针对气候变化健康影响的应对措施仍显不足。

中国在一些行业开展了大量的行动来应对气候变化。太阳能发电量的年增长率达到了前所未见的 26.5%，2019 年新增装机容量达到 26.8 GW。低碳能源投资是化石能源投资的九倍（2008 年为 1:1）；2018 年，可再生能源部门提供了 410 万个工作岗位，目前就业人数已超过化石燃料开采行业。此外，由于中国采取了强有力的政策措施，严重空气污染已有所缓解，2014-2019 年期间，城市中直径小于等于 2.5 微米（μm）的颗粒物（$PM_{2.5}$）的年均浓度下降了 28%，每年 $PM_{2.5}$ 相关过早死亡人数减少了 9 万人。这些大气污染控制政策也起到了减缓气候变化的效果，使得煤炭在中国一次能源供应总量中的比重从 2014 年的 66%降低至 2018 年的 59%。有三个省份已经制定了省级健康与气候变化计划，另有四个省份正在制定相关计划，展现了地方层面的领导力。

然而，尽管上述变化非常迅速，但为了确保实现中国的“2060 年碳中和”承诺、并最大限度地减少气候变化给中国带来的日益增长的健康负担，中国还需要做出更大的改变。尽管可再生能源的使用量不断增长，但煤炭在中国一次能源供应总量中的比重仍占到 59%。2018 年，中国的化石燃料补贴仍然高达 419 亿美元，而事实上，化石燃料使用导致的 $PM_{2.5}$ 空气污染所造成的早逝损失（即负外部性）已经高达 107 亿美元。虽然中国的大气污染水平已有显著改善，但仍有 42% 的人口居住在未能达到世界卫生组织（WHO）过渡期空气质量标准的地区，几乎所有城市的 $PM_{2.5}$ 浓度都超出了 10 微克/立方米（$\mu g/m^3$）的年均水平目标值。由于《健康中国行动计划（2019-2030 年）》中并未提到气候变化且中国尚未出台针对健康的独立的国家适应计划，因此气候变化对健康的影响在中国并没有得到充分的认识。从更广泛的角度来说，健康和气候变化方面的媒体报道率和个人参与度仍然很低，相关知识的传播和参与也都很有限。中国需要在所有部门推动这方面的进展，从而扭转气候变化健康风险曲线的走高势头。

《柳叶刀倒计时 2020 年中国报告》提出的政策建议

《柳叶刀倒计时 2020 年中国报告》基于相关数据和研究结果，为中国健康和气候变化领域的关键利益相关者提出了五项建议：

1. **强化部门间合作。**气候变化是一项需要各行各业携手应对的艰难挑战。尽管中国一直致力于将健康纳入各项政策，但仍迫切需要卫生、环境、能源、经济、金融和教育等各个部门之间展开实质性合作。

2. **增强突发卫生事件应急准备。**尽管新冠疫情过后，中国会大力加强针对突发卫生事件的防范力度，但对气候在健康卫生领域所造成的或即将造成的威胁的认知尚未得到应有的重视。这些问题应被完全纳入应急防范和响应系统之内，以便于为未来的健康服务、医疗供应及基础设施需求事先做好准备。

3. **强化科研支撑，提升认知水平。**中国需要加强对人群健康及气候变化领域的研究支持，这有利于增强卫生系统对适应和减缓措施及其健康效益的认识。同时，还应充分调动媒体和学术界的力量以提升公众以及政界对该话题的认知程度。此外，中国政府应及时更新《健康中国行动计划（2019-2030 年）》，从而尽快应对气候变化所带来的健康挑战。

4. **提升减缓气候变化的行动力度。**中国承诺到 2060 年实现碳中和，这无疑是中国向前迈出的重大一步。因此，加速煤炭淘汰进程不仅对实现该承诺非常必要，同时对中国继续推进大气污染防治也至关重要。此外，中国政府应当取消化石燃料补贴，从而凸显当前化石燃料消耗的真实成本，同时也可避免对其即将在 2021 年生效的排放权交易制度的效力造成影响。

5. **确保新冠疫情后的经济复苏政策是对当前及未来我国人群健康的保障。**中国为新冠疫情后经济复苏所作的各项决策将决定未来几年内公共卫生领域的整体局势。如果中国政府未能将气候变化作为首要事项纳入其干预措施，则其民众的生命、生计以及可持续经济的长期愿景必将岌岌可危。

引言

气候变化威胁着各国人民的健康和福祉。[1] 中国大量人口都面临海平面上升、登革热、黄热病、基孔肯亚热等气候敏感型传染病以及森林火灾和热浪显著增加的威胁，因此尤其容易受这些健康影响的侵害。[2]

作为世界上第二大经济体以及人口最多、二氧化碳排放总量最大的国家，中国在应对气候变化的健康影响方面将发挥关键作用，其相关进展不仅能够造福于中国 14 亿人的健康，也能改善全球人口的健康。然而，人群健康与气候变化之间的联系尚未引起中国政府应有的重视。比如，最近通过的《健康中国行动计划（2019-2030 年）》并未提到气候变化，而该文件是中国全面卫生系统建设的关键路线图。[3] 随着落实《巴黎协定》日期的临近，距离实现可持续发展目标（SDGs）的最后期限也只剩下 10 年，2020 年起采取的气候行动将起到关键作用。[4-6] 同时，新冠疫情给全球造成的影响可能长达数年。因此，在各国制定针对疫情冲击的经济刺激计划时，应保持与《巴黎协定》和 SDGs 目标规划的一致性，以确保经济恢复的可持续性。

跟踪中国在健康与气候变化方面的进展，不仅能增进对健康与气候变化关联的认识，还有利于评估中国的气候行动是否足够，同时还能凸显气候政策对环境和社会目标的协同促进作用。为此，清华大学联合英国伦敦大学学院和另外 17 所国内和国际机构，编写了首份中国柳叶刀倒计时报告。本报告首次尝试跟踪中国在人群健康和气候变化领域各个方面的进展，沿用了柳叶刀倒计时全球报告中使用的指标定义和分组以及计算方法。这项工作会逐年改进相关指标的计算方法。今年的报告提出了 23 项指标，涵盖以下五个领域：气候变化影响、暴露和脆弱性；针对健康的适应措施、规划和韧性；减缓气候变化及其健康协同效益；经济与投资分析；以及公众和政府参与（见专题 1）。

本报告尽可能改进了全球报告中的数据来源和方法，以提高结果的空间分辨率（包括提供省级层面的结果），并提供与中国国情更相关的信息。本报告介绍了 23 项指标的结果，并在附录中详细说明了各项指标的方法、数据、局限性和未来改进方法；附录是本报告的重要组成部分。

专题 1：柳叶刀倒计时中国指标

第 1 节：气候变化影响、暴露和脆弱性

- 指标1.1：热和健康
  - 指标1.1.1：脆弱人群的热浪暴露
  - 指标1.1.2：热浪相关的过早死亡人数
  - 指标1.1.3：劳动生产力的改变
- 指标1.2：健康和极端天气事件
  - 指标1.2.1：森林火灾
  - 指标1.2.2：热带气旋

指标 1.3：气候敏感型传染病

第 2 节：针对健康的适应措施、规划和韧性

- 指标2.1：适应措施的规划和评估
- 指标2.2：适应行动和执行
  - 指标2.2.1：监测、预备和应对突发公共卫生事件的能力
  - 指标2.2.2：空调使用——利与弊

第 3 节：减缓气候变化及其健康协同效益

- 指标3.1：能源系统与健康
- 指标3.2：清洁的居民用能
- 指标3.3：大气污染、交通和能源
- 指标3.4：可持续健康交通

第 4 节：经济与投资分析

- 指标4.1：气候变化的健康和经济成本以及减排收益
  - 指标4.1.1：热相关早逝的经济代价
  - 指标4.1.2：热相关劳动生产力损失的经济成本
  - 指标4.1.3：空气污染相关早逝的经济损失
- 指标4.2：向零碳经济转型的经济分析
  - 指标4.2.1：健康的能源投资
  - 指标4.2.2：低碳和高碳行业的就业
  - 指标4.2.3：化石燃料补贴
  - 指标4.2.4：碳定价的范围和力度

第 5 节：公众和政府参与

- 指标5.1：社交媒体对于健康和气候变化问题的关注度
- 指标5.2：公众对健康和气候变化议题的关注度
- 指标5.3：科学论文中对健康和气候变化议题的关注度

第 1 节：气候变化影响、暴露和脆弱性

气候变化与维持健康的所有社会和经济决定因素相互作用，通过各种途径影响着人类生活和生计。[1] 本节试图了解气候变化与健康之间的相互作用，跟踪气候变化通过高温和热浪（指标 1.1.1-1.1.3）、极端天气事件（指标 1.2.1 和 1.2.2）和气候敏感型传染病（指标 1.3）影响中国人口健康的方式。考虑到中国对气候变化韧性的特殊性以及热带地区的漫长海岸线，本报告额外研究了跟踪热带气旋暴露的指标（指标 1.2.2）。

指标 1.1：热和健康

指标 1.1.1：脆弱人群的热浪暴露

主要发现：2019 年，影响中国 65 岁以上人口的热浪天数共计增加了 22 亿天——相当于一年之内该年龄段人口人均多经历了 13 个热浪天。

在各种因素的作用下，高温和热浪对老年人来说往往具有致命性，包括慢性病患病率升高和药物使用以及生理和行为反应受损。[7] 该指标以 1986-2005 年暖季为基准，使用网格温度数据和人口数据，跟踪了 2000-2019 年期间 65 岁以上人口经历的热浪天数。[8,9] 该指标将热浪天数定义为各网格日最高气温大于夏季基准日最高温度分布的第 92.5 个百分位且持续三天及以上的天数，这一定义最能体现中国热浪事件对健康的影响。[10] 相关方法和数据的详尽说明，请参见附录第 3-5 页。

从全国来看，热浪暴露在稳步增加，2000 年热浪天数比基准水平增加了 7,180 万天，2019 年更是达到了创纪录的 22 亿天，仅次于 2017 年的 22.4 亿天。相比基准年而言，2019 年的增长水平相当于每个 65 岁以上的老年人人均多经历了 13 个热浪天（见**图 1**）。仔细研究 2019 年的数据的话就可以发现，2019 年云南省每位老年人经历的热浪天数增加了 39 天，居全国之首，其次为香港（22 天）、海南省（18 天）。

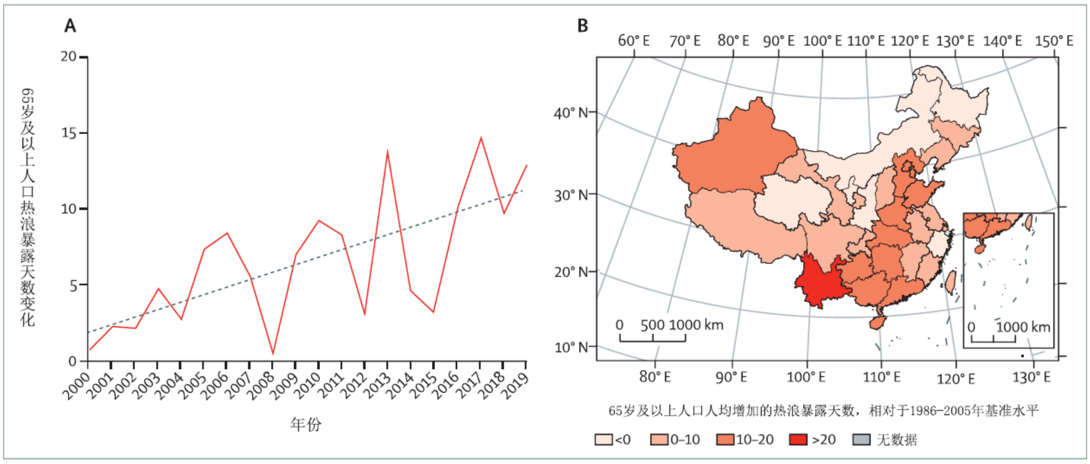

图 1：中国 65 岁及以上人口人均热浪暴露天数相对于 1986-2005 年平均水平的变化。（A）国家层面的结果；（B）省级层面 2019 年结果。

指标 1.1.2：热浪相关的过早死亡人数

主要发现：2019 年，中国热浪相关死亡人数达到 26,800 人，在过去的十年中，死亡人数每 1.2 年增加 1,000 人，其中华东和中南地区热浪相关死亡人数最多。

热浪事件会导致人群死亡率增加，通常为心血管疾病和呼吸道疾病加重引起的死亡。[11] 该指标利用网格人口数据和气温数据、指标 1.1.1 中的热浪定义和不同地区的暴露反应曲线，使用附录第 5-6 页所述的方法评估了各年龄段的热浪相关死亡人数。[8,10-12]

2019 年，在中国约有 26,800 人的死亡与热浪有关，且近年来的上涨趋势越来越明显（见*图 2*）。取五年移动均值， 1999 至 2009 年期间，年热浪相关死亡人数每增加 1,000 人平均用了 3.8 年时间，但 2010 至 2019 年期间，年热浪相关死亡人数每增加 1,000 人只用了 1.2 年。在所有省份中，山东省的热浪相关死亡人数最多，其次为河南省和安徽省，分别位于中南和华东地区。

除热浪会导致死亡率增加外，更广义的高温也会导致死亡率增加。有研究显示，2069 至 2099 年期间，2 ℃情景下中国每年与高温相关的死亡人数将比 1.5 ℃情景下增加 27,900 人[13]。到本世纪 80 年代，在 RCP 4.5 和 RCP 8.5 情景下，北京地区因高温引发的心血管病死亡率将分别提高 69.0%和 134%。[14]

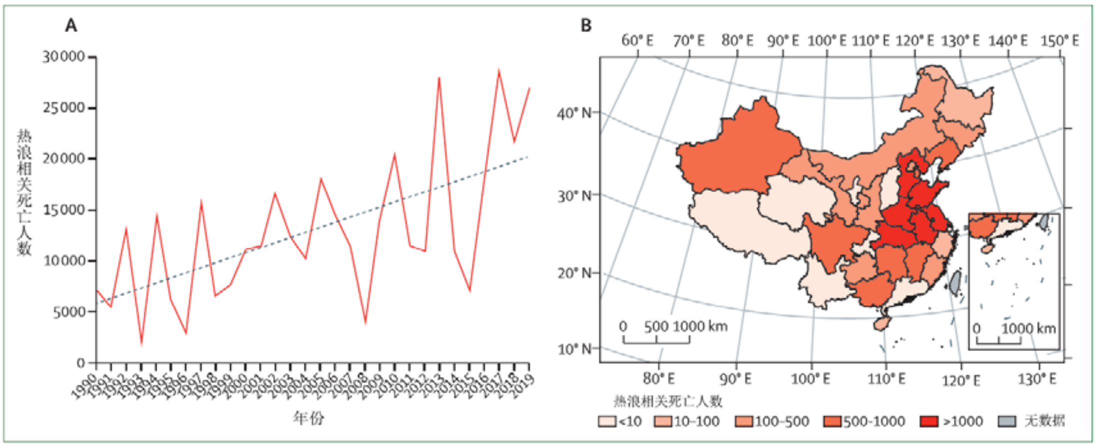

*图 2：*中国的热浪相关死亡人数。（**A**）**1990-2019** 年期间热浪相关死亡人数及变化趋势，（**B**）**2019** 年各省因热浪造成的死亡人数。

指标 1.1.3：劳动生产力的改变

主要发现：2019 年，中国因高温损失了 99 亿劳动小时数（占全国总劳动时间的 0.5%），其中广东省的劳动时间损失占全国总损失的近四分之一。

气候变化造成的热应力增加会降低劳动人群的工作效率及生产力，从而给社会经济发展带来负面影响。[2,15,16] 围绕这一现象，基于湿球黑球温度（WBGT）与劳动损失时间的关联函数，指标 1.1.3 评估了我国第一产业（农业、林业、畜牧业和渔业）、第二产业（制造业、建筑业和采矿业）和第三产业（金融业和其他服务行业）的劳动时间损失小时数。具体计算方法参见柳叶刀倒计时全球报告和附录第 6-9 页。[17]

2019 年，中国因高温造成总劳动时间的损失超过 99 亿小时（比 2000 年高出 4.8%），占全国总劳动时间的 0.5%。从 2000 年开始，第一产业的人均损失小时数每年增加 6.2%，2019 年达到了 36 小时（约 4.5 个工作日）。广东省作为中国人口密度最大、经济最发达的省份（对中国 GDP 的贡献率达 11%），其劳动损失时间占全国总损失时间的四分之一（约损失 24 亿劳动人时数）；该省第一产业和第二产业的人均劳动损失时间分别达到 14.3 天和 5.2 天，严重损害了劳动人群的生产力水平。

指标 1.2：健康和极端天气事件

指标 1.2.1：森林火灾

主要发现：2016-2019 年期间，中国 24 个省份每年的森林火灾人口暴露天数都在 2001-2005 年水平的基础上有所增加。其中，华北和东北地区增加的天数最多。

森林火灾不仅会对人造成直接的热伤害和死亡，其烟尘引发的急性和慢性呼吸系统症状恶化也会增加暴露人群的伤亡率。[2] 本报告通过同一网格中卫星火点数据与人口数据的叠置分析、清点结果网格中的森林火灾暴露人天数来估计人口的森林火灾暴露变化（去除了人口密集的城市地区）。[9,18] 报告计算了四个时间段的年均暴露天数：2001-2005 年、2006-2010 年、2011-2015 年和 2016-2019 年。从全国来看，年均总暴露在前三个时间段内均有所增加，最后一个时间段内有所降低，这可能是城市化水平的提高导致的。然而，从各省来看，在全国 34 个省份中，有 24 个省份在 2016-2019 年期间森林火灾暴露人天数要多于 2001-2005 年期间。北方和东北各省的情况最为严重，包括黑龙江、吉林和天津，这些地区亟需加强森林火灾的监测和控制。

指标 1.2.2：热带气旋

主要发现：2000-2019 年期间，与基准水平相比，中国发生强台风和"超级"台风的频率显著增加。

中国东部海岸线绵延，经常受到热带气旋的影响，造成人员伤亡、传染病的传播以及心理健康损害。[19-24] 该指标利用国内数据对气旋暴露水平和破坏程度进行跟踪，是柳叶刀倒计时中国报告的独有指标。[25,26] 本报告介绍了热带气旋的频率、强度和在省级行政单元水平的时空分布情况。以 1980-1999 年水平为基准，本报告计算了 2000-2019 年期间不同强度等级的热带气旋的出现趋势。与基准水平相比，2000-2019 年期间，发生强台风和超级台风的频率在统计学上显著增加，而发生热带低气压和热带风暴的频率明显减少。在此期间，由于采取了更好的适应措施，热带气旋对我国东部福建、浙江等热点登陆省份造成的破坏程度已明显减轻（见附录第 20-25 页）。

指标 1.3：气候敏感型传染病

主要发现：自 20 世纪 60 以来，通过埃及伊蚊和白纹伊蚊传播登革热的媒介能量分别提高了 37%和 14%。

登革热是一种典型的气候敏感型媒介传染病，登革热的气候适宜性在世界各地都有所上升。媒介能量主要衡量埃及伊蚊和白纹伊蚊传播登革热的媒介能量变化，表示为在考虑每日温度的影响下，1 例感染病例引发的日均病例数。计算媒介能量的方法与 2019 年柳叶刀倒计时报告和 Rocklöv 和 Tozan 等人采用的方法一致。[27] 与 1961-1965 年水平相比，通过埃及伊蚊和白纹伊蚊传播登革热的气候适宜性在 2014-2018 年期间分别提高了 37%和 14%。同时，中国登革热疾病负担在全国范围内持续而显著地增长，包括发病和伤残调整生命损失年（DALYs）等。2017 年，中国登革热全年龄段发病率和伤残调整生命损失率分别比 1990 年增加 5.7 倍和 4.7 倍，达到十万分之 183.8 和十万分之 1.8。[28]

中国还面临着其他几种气候敏感型传染病的影响，在当前和将来都会给中国人口带来威胁。比如，中国南方的气温每上升 1-2℃，疟疾的传播潜力将提高 39%-140%。[29]

小 结

总的来说，本节提出了确凿的证据表明，气候变化对中国居民的健康影响正迅速上升，且中国各省受到的影响各不相同。不同地区面临着独特的健康威胁，需要采取有针对性的应对措施，关于全国各省差异性的综合评估，见***图 3***。在河南、山东和浙江三省报告的六项指标中，有五项指标在 2000-2019 年期间的增速（或恶化速度）超过了 10%。仅这三个省份的人口和 GDP 就占到了全国的 20%左右。[30] 在大多数人口密集、经济发达的东部和北部省份中，有三项以上指标都提高了至少 10%，表明中国的大部分人口和经济部门都面临着风险。这些发现有力地证明，中国需要加强气候变化适应和减缓方面的干预措施，下文将对相关指标进行跟踪。

上述研究结果绝非气候变化对中国居民健康影响的详尽清单。因此，需要进一步研究各省内部不同地区的健康影响，同时需要进一步开发新指标，包括有关海平面上升、其他极端天气事件、其他气候敏感型疾病和心理健康的指标。[31]

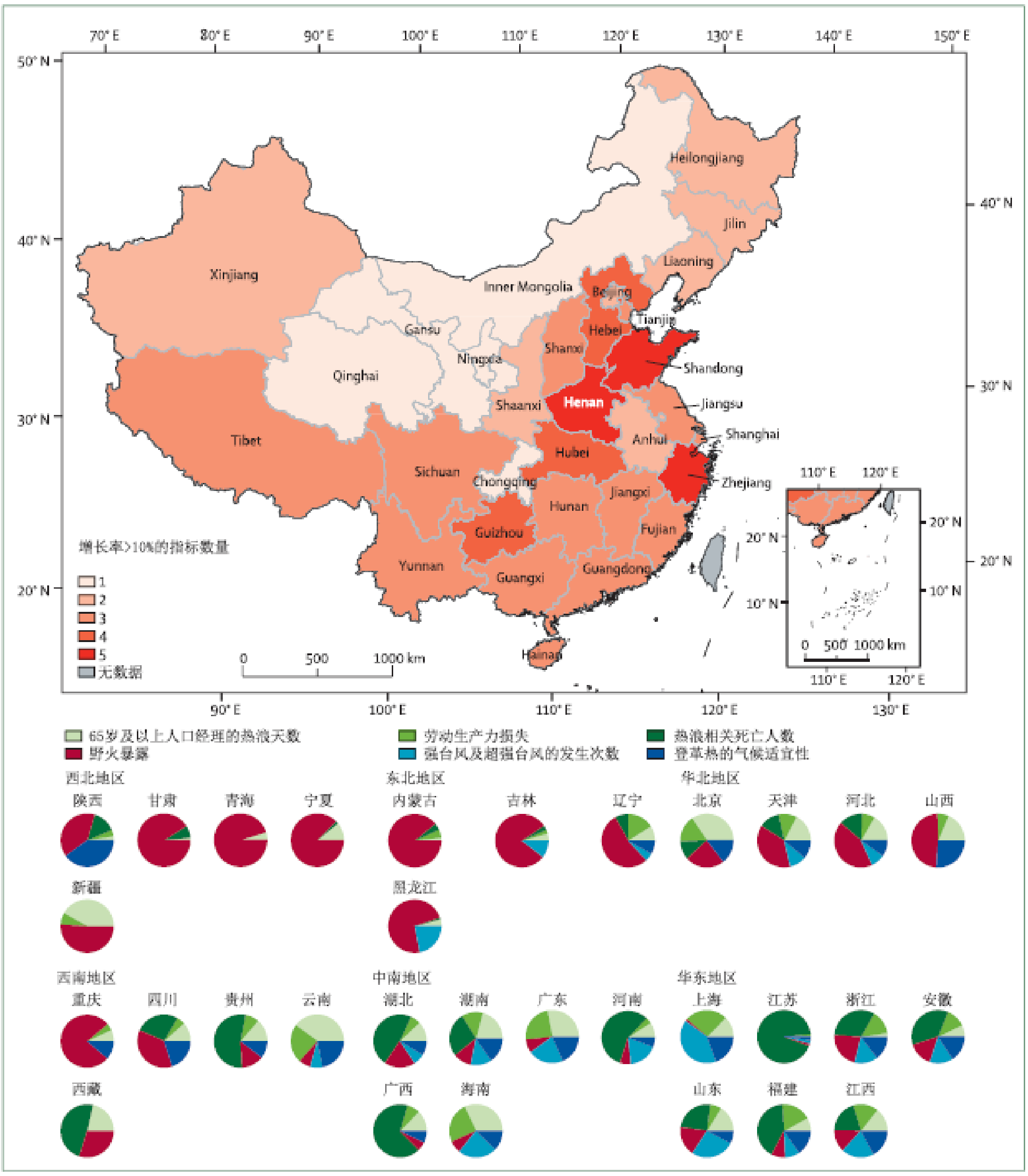

图 3：中国各省因气候变化而上升的主要健康风险

注：各省份按 2000-2019 年期间增长率大于 10%的指标数量标色，颜色越深，代表省份快速增长的指标的个数越多。饼状图中的每种颜色代表一种跟踪的指标。由于各指标的权重相同，因此扇区的大小与不同健康风险的变化率成正比。

第 2 节：针对健康的适应措施、规划和韧性

第 1 节中强调的气候变化对健康的影响需要社会各界的协同应对，其目标是减少温室气体（GHG）排放并适应已经造成的影响。2007 年，中国政府发布《中国应对气候变化国家方案》，[32] 开始认识到气候变化对健康的影响。中国各省市也越来越多地制定针对健康问题的气候变化适应战略。这些措施主要针对热浪、洪水、干旱等极端天气事件对健康的影响，但由于没有国家健康适应计划的指导，中国各地区应对气候变化的能力差异很大。第二节从全球报告中选取了两个领域的指标：适应性规划和评估（指标 2.1）以及适应措施的交付与实施（指标 2.2.1 和 2.2.2）。目前正在制定第三类指标，即健康方面的气候信息服务，见附录第 46-47 页。

指标 2.1：适应措施的规划和评估

主要发现：中国尚未出台专门针对气候变化健康问题的国家适应计划；但在 2020 年调查的 17 个省（直辖市）中，有三个省份出台了相关的省级层面适应计划，另有四个省份正在制定相关的计划。已有六个省份完成有关健康和气候变化影响、脆弱性的全面评估工作。

WHO 提出了针对健康问题的国家适应计划（H-NAP），[33] 包括对特定气候变化的影响和脆弱性评估，这是各国开展气候变化健康风险管理的重要举措，目前全球约有 50 个国家完成了这方面的工作。[2] 尽管中国已经制定较为广泛的气候变化适应策略，这些策略也在一定程度上涉及到健康方面的问题，但仍未针对健康领域适应气候变化进行全面的规划与评估。为了跟踪中国各省份在气候变化健康适应计划和评估方面所做出的努力，2020 年 5 月，由中山大学和中国疾病预防控制中心牵头，在参照 WHO 健康与气候变化全球调查的基础上，[34] 开展了基于中国的健康与气候变化适应专项调查工作。本次调查的实施细节以及相关数据、分析和说明，可参见附录第 32-38 页。

在完成调查的 17 个省份中，有三个省份（广东、上海和四川）已经制定了省级层面的健康和气候变化适应计划，另有四个省份正在制定相关的适应计划。有四个省份也表示，省级卫生健康部门和气象部门正在开展健康与气候变化适应计划和策略方面的密切合作。但同时也发现，缺乏多部门协作机制（所有被调查

的省份）、缺少政府财政支持（82%）和国家监测系统（82%）是当前阻碍地方层面制定针对健康领域的气候变化适应计划的主要挑战。

在脆弱性评估方面，17 个调查省份中有六个省份称其对气候变化与健康问题进行了综合评估，包括气候变化的健康影响、脆弱性和适应性评估。其中，评估关注最多的是热浪的健康影响。在国家层面的评估报告中，也已经将人群健康作为评估的重要内容之一，如《中国气候与环境演变》（2012）；[35]《气候变化国家评估报告》（2015）；[36] 以及《气候变化绿皮书——应对气候变化报告》（2014 和 2019）。[37,38]

尽管国家和省级层面都已开展气候变化与健康方面的评估，但评估结果对卫生健康决策和资源配置（如增加人员编制、财政经费支持等）方面的影响十分有限。中国未来需要加强气候变化领导力，建立长期的卫生资源投入机制，以制定更加全面的针对健康问题国家适应计划，从而减少气候变化带来的健康威胁。[39]

指标 2.2：适应行动和执行

指标 2.2.1：监测、预备和应对突发公共卫生事件的能力

主要发现：各省应对突发公共卫生事件的能力存在明显的地区差异。华东地区的能力指数高于中国其他地区，其中，江苏省的能力指数最高，达到了 69.7 分（满分 100 分）。

气候变化会加剧疾病传播并诱发热浪、洪水、热带气旋、森林火灾等相关极端气候事件，从而影响人群健康。监测并及时应对突发公共卫生事件的能力对于最大程度地减少传染病和气候相关极端事件的影响具有十分关键的作用。[40] 虽然中国各省的突发公共卫生事件管理体系存在相似之处，但仍存在很大差异，导致不同省份应对气候变化对健康的影响的能力也各不相同。针对这一指标，研究团队基于“城市体检”相关思路与成果 [41] 制定了一套综合指标体系，供省级政府使用，其中包括风险暴露与预备、监测与响应以及资源支持与社会参与等维度。指标组成包括城市人口密度、突发公共卫生事件应急预案的完备性、传染病报告系统建设情况、每千人口医疗机构数和医护人员数等。全部指标组成、数据来源和权重的详细信息可参见附录第 38-44 页。根据 2018 年最新数据，各省突发公共卫生事件应急管理的平均指数得分为 48.1 分（满分 100 分）。结果显示，华东地

区的突发公共卫生事件应急管理能力指数得分总体高于其他地区，其中江苏省（69.7 分）、山东省（68.9 分）和北京市（60.9 分）居前三（见图 4）。

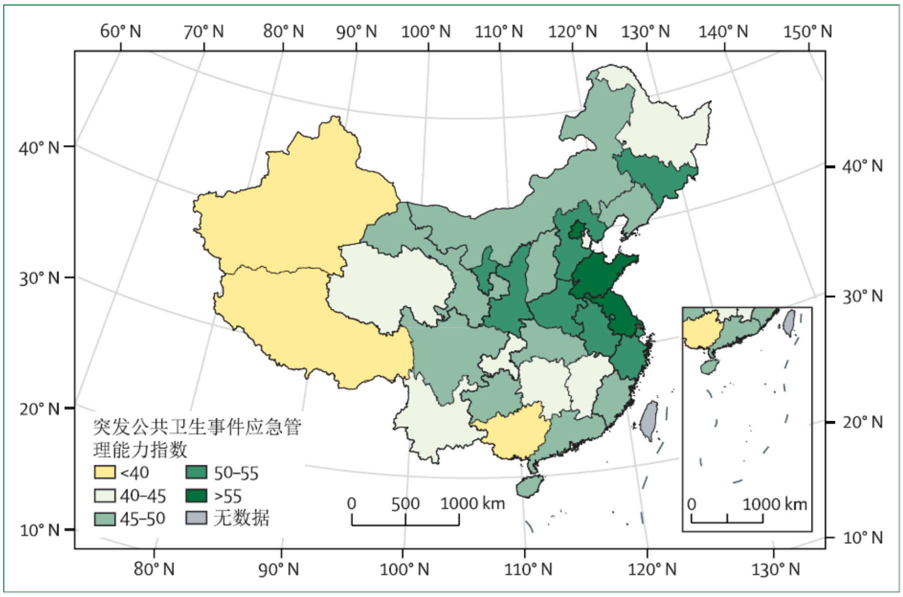

***图 4：*中国各省突发公共卫生事件应急管理能力指数**

指标 2.2.2：空调使用——利与弊

主要发现：空调的使用大大降低了由高温诱发的死亡率，但同时使得能源消耗和二氧化碳排放显著增加，在 2000-2016 年期间，其二氧化碳排放增加了近 1000%，从每年 620 万吨飙升至 5850 万吨。

使用空调能够降低高温相关疾病的发病率和死亡率，[42] 但如果空调的用电来源是化石能源，空调使用时就会带来二氧化碳和 $PM_{2.5}$ 排放，从而危害环境。空调使用还会产生废热，加剧城市热岛效应，还可能泄漏氢氟碳化物等破坏性极强的温室气体。[43] 因此，还需要采取其他措施来提高城市绿地可及性，改善建筑设计以提升运行能效，并实现建筑被动式降温。该指标利用国际能源署（IEA）数据和 2019 年柳叶刀倒计时全球报告中的相对风险（RR），计算了因使用家用空调而避免的热浪相关死亡人数，相关方法和说明可参见附录第 45 页。

中国在 2000-2016 年期间，由于家用空调使用的增加而避免的热浪相关死亡率翻了一番，在 2016 年达到 45%（见*图5*）。然而，空调使用的日益增多也导致能源消耗、二氧化碳排放和空气污染增加到令人担忧的水平。2001-2015 年期间，中国城市家用空调的人均能耗从 16.4 kWh 增加到 96.6 kWh 的惊人水平，空调相

关二氧化碳排放增加了近十倍，从 620 万吨/年增加到 5850 万吨/年（*图 5*）。这些趋势令人深感担忧，它表明快速采用空调技术存在风险，从长远来看会加剧温室气体排放，因此还需要采取其他热适应措施，包括提高建筑能效、被动通风和增加城市绿地，最大程度地削弱空调使用的消极影响。[44,45]

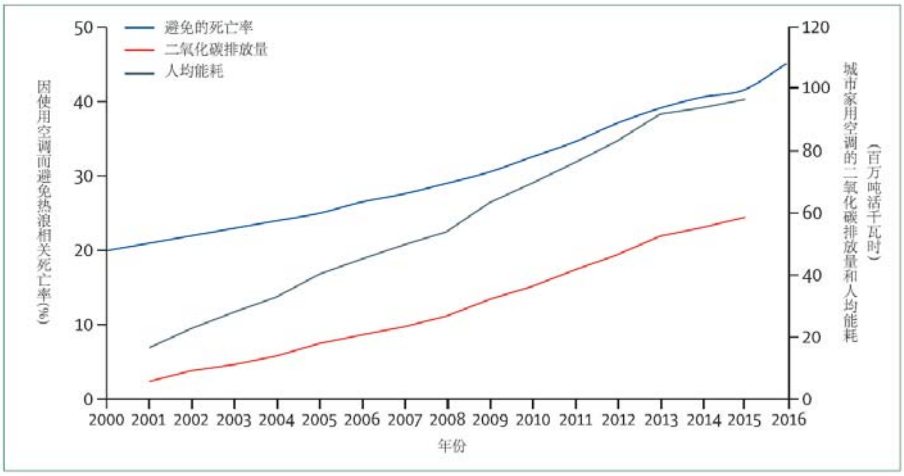

图 5：**中国因使用家用空调而避免的热浪相关死亡率和城市家用空调能耗**

小 结

适应气候变化带来的不良影响已成为各国的当务之急。本节讨论了对气候变化带来的多维健康风险进行系统规划与评估的重要性，还讨论了空调这一具体的适应措施的利与弊，未来几年本报告会追踪更多的适应行动进展。上述指标表明，中国当前的适应努力仍处于初级阶段，各省都取得了不同程度的进展。考虑到地理和社会经济条件的差异以及各省所面临的气候变化相关健康风险，中国需要加强地方规划并在全国范围内开展综合评估，尽可能地降低气候变化带来的公共卫生风险。

第 3 节：减缓气候变化及其健康协同效益

加快采取强有力的气候变化减缓措施不仅可以限制气温上升的影响（见第 1 节），还可以对人群健康产生直接的积极影响。这些改善体现在一系列经济部门，其结果是空气更加洁净、饮食更加健康、体育活动更加频繁。比如，2013-2017 年期间，中国通过逐步淘汰落后产能、在居民区推广清洁燃料等措施，在 2017 年避免了约 21 万人因空气污染而过早死亡。[46] 后续研究也越来越多地证实了这些

结论，这表明大规模减少化石燃料使用有助于进一步减少空气污染，对人群健康有显著的益处，助力中国向可持续发展转型。[47-49]

中国政府在《巴黎协定》下做出了在2030年前实现碳达峰的承诺。[50] 然而，近年来，全球碳排放量持续增长，2018年达到了553亿吨二氧化碳当量（CO_2e）。若想将温升控制在“2℃以内”，将需要国际社会和中国一道携手采取更有力的措施。[51] 近几十年来，中国经济快速发展，其温室气体排放量却没有同步增长，2000-2019年期间其经济体系的碳强度下降了36%。[52,53] 从绝对值来看，中国的温室气体排放量在经历了五年的平台期后，已从2017年的116亿吨 CO_2e 减少至2018年的103亿吨 CO_2e。中国的碳排放曾经在2000年后以年均10%的速度激增，2013年碳排放达到顶峰（101亿吨 CO_2），2014-2016年期间有所下降，[54] 从2017年（97亿吨 CO_2）开始反弹，2018年增加至99亿吨 CO_2。[55] 由于新冠疫情，2020年第一季度中国的二氧化碳排放量在2020年第一季度大幅下跌下降，比2019年1-4月减少了6.9%，[55] 使得全国人口加权 $PM_{2.5}$ 浓度因此下降了14.5 μg/ m^3。[56] 采取与《巴黎协定》相一致的经济恢复措施，如调整经济结构、提高可再生能源的比重和提高能效，将确保我们在减少温室气体排放量减排和改善健康改善两方面持续进步。本节跟踪了中国为减少温室气体排放所做的努力以及各行业的相关协同效益。在首部中国版柳叶刀倒计时报告中，共提出了四项指标，即能源与健康（指标3.1）、清洁的家用能源（指标3.2）、空气污染（指标3.3）以及可持续、健康的交通运输业（指标3.4）。今后几年将进一步增加相关指标，尽力涵盖其他经济部门，以便更直接地反映这些指标对气候变化和健康的影响。

指标3.1：能源系统与健康

主要发现：2016年后，中国煤炭消费量出现反弹；2018年，在一次能源供应总量中，煤炭供应量增加到了80.2 EJ。同时，风能和太阳能发电量继续快速增长；2019年，可再生能源在中国发电总量中占比达到13.4%。

在中国，能源系统排放的二氧化碳比其他任何部门都要多，是空气污染相关疾病负担的主因。该指标利用中国能源统计年鉴数据，报告了降低中国能源系统碳强度的关键措施——煤炭退出和零碳电力的进展情况。[52]

2013-2016 年期间，由于中国采取了有效的空气污染防治措施，[57, 58] 煤炭在一次能源供应总量（TPES）中的占比有所降低。但在随后的几年中，这种下行趋势发生了扭转，主要是因为中国出台了经济刺激政策，导致 2018 年 TPES 中的煤炭量增加到了 80.2 EJ。[30] 鉴于尽早淘汰煤电的重要性，以及现有技术在环保和经济性方面的优势，这一趋势尤其令人担心。

积极的一面是，国家低碳电力的比重持续上升，从 2000 年的 17%提高至 2019 年的 31%。[52] 令人感到欣慰的是，2019 年，可再生能源（太阳能、风能）在总发电量中占比 13.4%，其中，太阳能发电量继续以每年 26.5%的空前速度增长。2019 年，中国新增太阳能、风能装机容量分别达到 26.8 GW 和 25.7 GW，这个增长速度相当于中国每天建成了一座 70 MW 级的现代风电场和一座同样大小的现代太阳能电站。为实现《巴黎协定》中的国家自主贡献（NDC）目标，中国需要结合 TPES 和低碳电力的进展制定更加综合的经济刺激计划。

指标 3.2：清洁的居民用能

主要发现：家庭中清洁健康能源的使用需要进一步提高。2018 年，化石燃料在烹饪用能中的占比超过 74%；2013 年，生物质能在农村地区家庭总用能中的占比仍然达到了 61.4%。

自 2000 年以来，中国清洁能源的供应量和使用量迅速增加，这对社会发展、人群健康与福祉具有重要意义。[59] 该指标分别利用国家统计局汇编数据显示了家庭能源消费情况，并利用清华大学建筑节能研究中心收集的数据，分析了家庭生活热水和烹饪使用的燃料情况。2000-2017 年期间，家庭人均能耗大幅增长，从 2000 年的 3.9 GJ 增加到 2017 年的 12.2 GJ，涨幅达 215%；电能占家庭能源消费总量的比例从 2000 年的 10%上升至 2018 年的 20%。同时，家庭化石燃料消费小幅减少，但仍然处于高位，占中国城市烹饪用能的 74%以上。生物质能仍然是农村家庭用能的主要来源，2013 年占家庭用能的 61.4%，增加了室内空气污染的健康负担。[60] 使用电炉代替燃气、燃煤和生物质能灶具（并连接到由可再生能源供电的电网）可为改善中国家庭能源结构带来重大机遇，从而减少家庭温室气体排放和空气污染。[61]

指标 3.3：大气污染、交通和能源

主要发现：2015-2019 年间，中国城市的 $PM_{2.5}$ 污染下降了近 28%，这段时间内因空气污染导致的过早死亡人数减少了 9 万。但是，仍有 42%的中国人口暴露在 $PM_{2.5}$ 的年均浓度超过 35 μg/m³ 的环境中。

空气污染是导致过早死亡的最重要的环境风险因素，[2] 中国已经采取一系列强有力的应对措施来改善空气质量。该指标利用中国生态环境部数据中心呈报的 367 个城市的 $PM_{2.5}$ 每日监测数据，回顾了城市中 $PM_{2.5}$ 浓度的变化情况。[62] 该指标还估算了不同行业、地区因室外 $PM_{2.5}$ 导致的过早死亡人数：通过将 IEA 和中国统计年鉴数据整合到 GAINS 模型中（可参见柳叶刀倒计时全球报告），估算 $PM_{2.5}$ 暴露，并根据暴露-反应关系估算死亡人数。[63] 该指标对数据集和方法进行了详细的描述（见附录第 58-63 页）。

随着中国清洁空气政策（如《大气污染防治行动计划》（APPCAP））的实施，2015-2019 年期间，367 个城市整体的空气污染减少了近 28%。[57,58,64] 相应地，因室外 $PM_{2.5}$ 污染造成的死亡人数较 2015 年减少了 10%（2018 年为 83 万人）。但是，2018 年，仍有 42%的中国人口暴露在浓度超过 WHO 中期空气质量目标 1（年均 $PM_{2.5}$ 浓度为 35 μg/m³，这是三个中期目标中最不严格的一个）的环境当中。[65,66] 2018 年，工业和农业部门造成的可归因于空气污染的早死人数占总数的 53%，其次为交通运输部门（10%）、住宅部门（8%）（见***图 6***）。在区域层面，华东地区的过早死亡人数最多，其中农业和工业部门对 $PM_{2.5}$ 的贡献率最高，其次为中南地区。除西藏的几个城市外，[62] 中国所有城市的 $PM_{2.5}$ 浓度持续超过 WHO 年均浓度建议值 10 μg/m³。中国有机会在未来几年继续减少暴露于空气污染中的人口数量，关键是将新冠疫情的经济复苏计划与空气污染政策、巴黎协定和可持续发展目标相结合。但是，考虑到未来气候变化会加剧污染物的累积效应，如果经济复苏计划间接地增加了化石燃料的使用，就有可能会逆转近期中国空气质量的改进势头。[67-69]

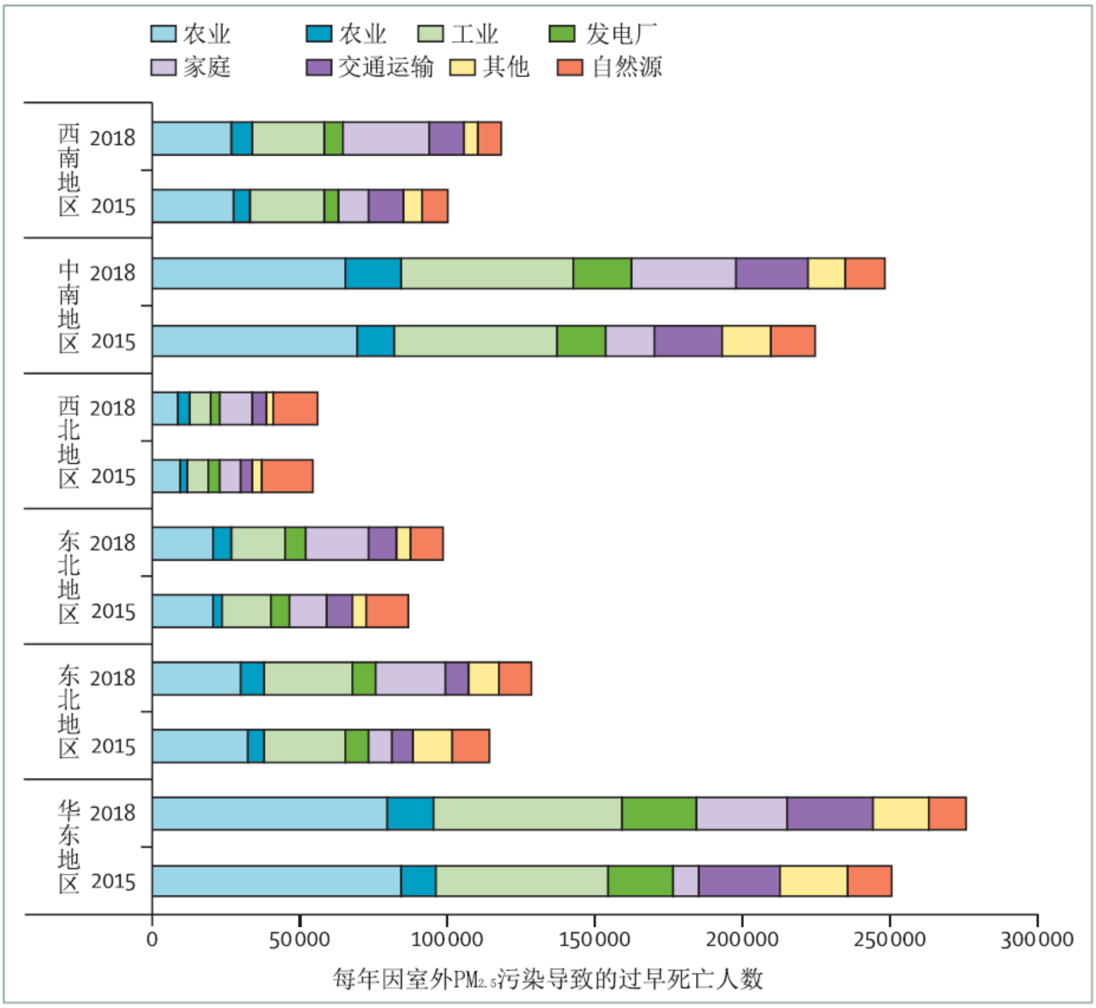

图 6：**2015、2018 年中国各污染源导致的可归因于细颗粒物（$PM_{2.5}$）暴露的早逝人数**

指标 3.4：可持续健康交通

主要发现：2000-2018 年期间，中国道路交通运输业四种主要空气污染物的排放强度，即每辆车的平均排放量，减少了 90%以上。

除了排放大量 CO_2 外，道路交通运输中化石燃料的燃烧还会释放其他有害污染物，包括碳氢化合物（HC）、一氧化碳（CO）、氮氧化物（NO_x）和 PM_{10}，威胁着中国的公众健康，尤其是在人口稠密地区。就该指标而言，道路交通运输排放强度（以排放量与汽车保有量的比值计算）决定着全部车辆的平均排放量，包括电动、混合动力和天然气汽车。数据来源于《中国机动车环境管理年报》和国家统计局。[30,64] 2000-2018 年期间，中国 CO、HC、NOx 和 PM_{10} 的排放强度分别下降了 92%、91%、91%和 94%，充分体现了道路交通运输排放控制的成效（见*图 7*）。同一时期，每辆车排放的 NOx 和 PM_{10} 分别从 0.24 吨和 0.03 吨下降到 0.02 吨和 0.002 吨。2010-2018 年期间，北

京、上海和广东的排放强度分别降低了 42%、44%和 71%。排放标准的提升以及正在进行的运输方式转变是前述排放强度下降的主要原因。[70] 2019 年，中国的电动汽车（EV）数量达到了 310 万辆，相比 2014 年，年增幅达到 60 万辆。相比于美国（第二大 EV 市场）2014-2019 年期间 26 万辆的年均增幅，这一增长水平着实惊人。[71] 此外，近几个月来，为控制疫情，许多城市的交通运输活动都显著减少，空气质量得到改善。[56,72,73] 然而，由于 NO_x 和挥发性有机化合物（VOCs）排放的减少不均衡导致大气氧化能力提高，中国东北地区因此意外地发生了几次雾霾事件。[74,75]

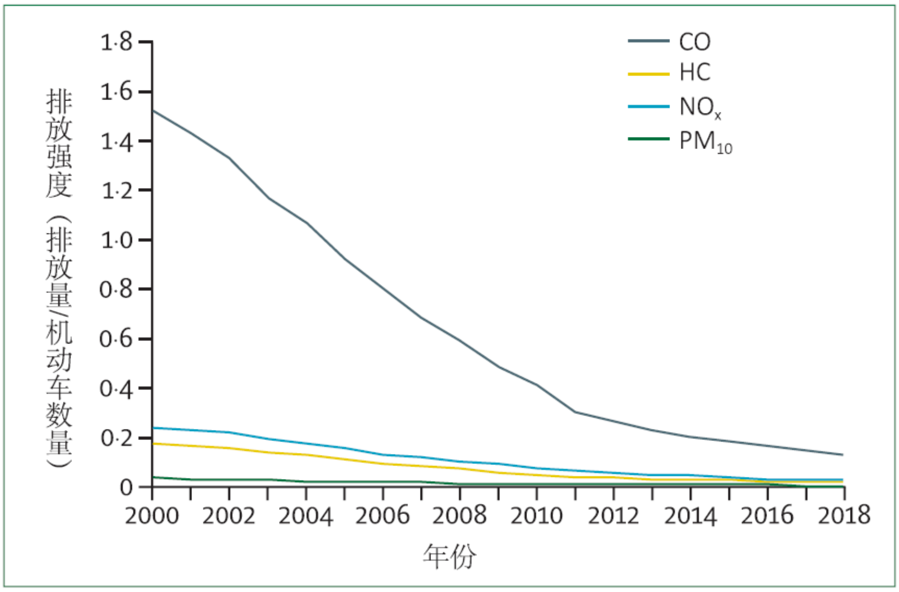

***图 7*：2000-2018 年期间中国道路交通运输业 CO、HC、NOx 和 PM_{10} 空气污染排放强度**

小 结

本节中的指标突出了中国在减缓气候变化方面取得的显著进展，以及由此带来的空气污染的明显减少和健康方面的巨大收益。然而，这些指标也清楚表明，要想实现《巴黎协定》的长期目标，必须进一步做出更大努力。煤炭依然是发电的主要燃料来源，也是空气污染和健康损害的核心原因，因此必须尽早、尽快淘汰。

第 4 节：经济与投资分析

本节关注中国的气候变化影响以及应对气候变化的经济和投资分析。越来越多的证据表明，如果在气候变化问题上无所作为，将付出高昂的健康成本，[76] 而对中国许多部门和许多地区来说，在减缓气候变化的众多效益中仅健康效益这一项便远远超过其成本。[77,78] 从成本效益的角度得到的结论也很清晰，中国有很多

气候行动是成本有效的，中国值得为改善人群健康进一步加强应对气候变化的措施力度。本节中的七项指标分为两大类：气候变化及其减缓的经济影响（指标 4.1）；向零碳经济转型的经济分析，包括低碳经济投资（指标 4.2）和化石燃料温室气体排放定价（指标 4.2.4）。

指标 4.1：气候变化的健康和经济成本以及减排收益

指标 4.1.1：热相关早逝的经济代价

主要发现：2019 年，中国热相关死亡的损失高达 136 亿美元，约为 1993 年损失的 13.6 倍，相当于 130 万人的国民收入。

该指标利用中国的平均统计生命价值（VSL），即 320 万美元（2015 年价），对中国各省热浪相关死亡进行了货币化。[79] 从全国来看，热浪相关死亡的损失从 1993 年的 10 亿美元增加到了 2019 年的 136 亿美元（见***图 8***），相当于 130 多万人的国民收入。华东地区损失最大，2018 年达到了 61 亿美元，约占地区 GDP 的 0.11%（山东省的损失高居首位，达 50 亿美元，占地区 GDP 的 0.38%）。

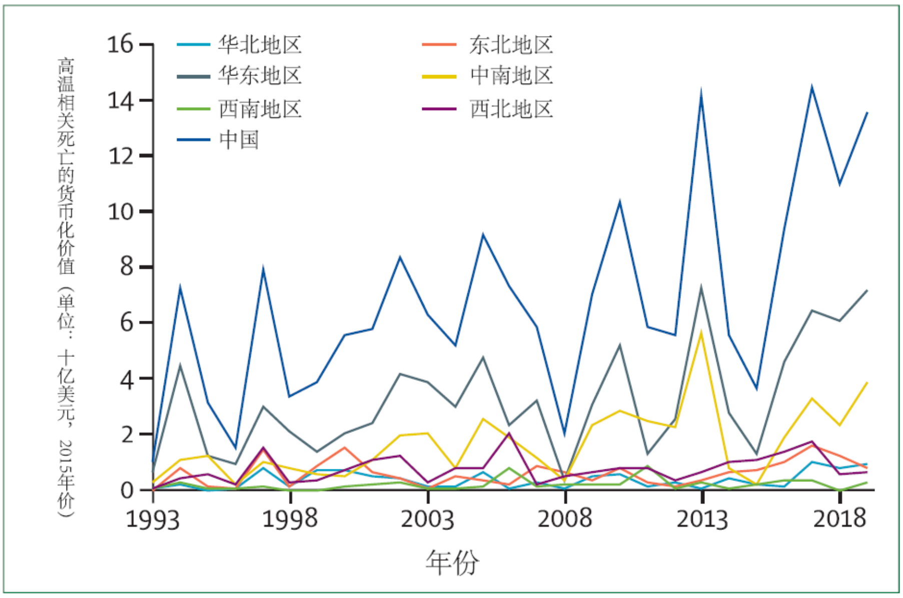

图 8：**中国各地区热相关死亡的货币化价值**

指标 4.1.2：热相关劳动生产力损失的经济成本

主要发现：2017 年，热相关劳动生产力损失的经济成本达 1260 亿美元（占中国当年 GDP 的 1.14%），而 2007 年仅为 330 亿美元（占当年 GDP 的 0.47%）。

从该损失的省级分布看，广东（1.65%）、海南（1.41%）和广西（1.22%）三省的损失最大。

该指标衡量了热相关劳动生产力损失（指标 1.1.3）的年度经济总成本。通过投入-产出分析框架，该指标分别估算了直接损失（直接受高温影响行业中劳动生产力的一阶损失）和间接损失（由于对遭受直接损失的行业的依赖而导致的其他行业的高阶损失）。[80-82] 2017 年，劳动生产力损失的经济成本数额为 1260 亿美元（占 GDP 的 1.14%），是 2007 年成本的近四倍，相当于国家在科技或环境保护领域的财政支出规模（见***图9 A***）。2015 年，总成本中近 70%为间接成本，主要发生在第二产业（制造业、建筑业、公用事业和采矿业）。就经济成本的地区分布（按该成本在地区 GDP 中的占比计算）而言，中南部地区的成本高于其他地区（见***图9 B***）。2015 年，成本排名前三的省份为广东（占该省 GDP 的 1.65%）、海南（1.41%）和广西（1.22%），均为气候温暖湿润的南部省份。

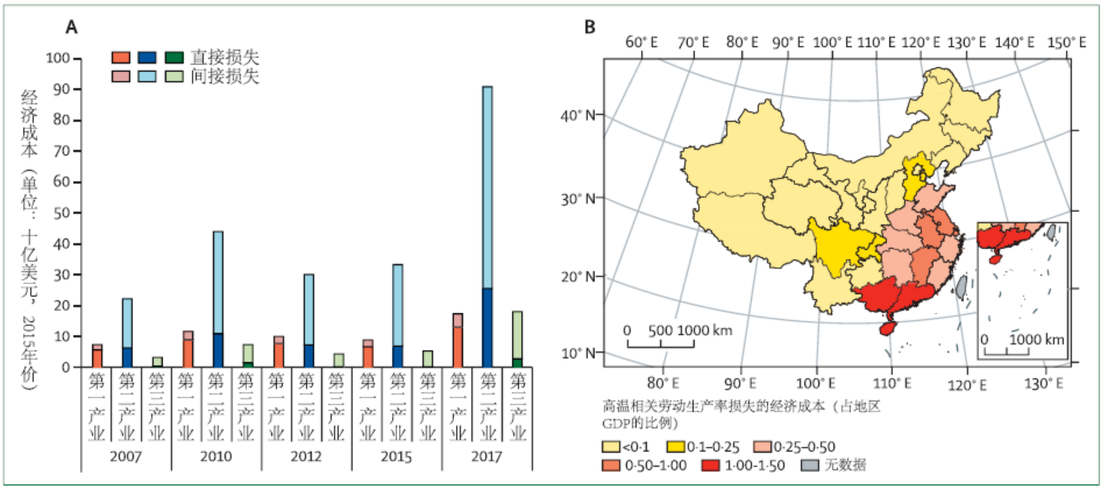

***图 9*：热相关劳动生产力损失的经济成本。（A）国家层面各年份分三次产业结果，单位：十亿美元（2015 年价）（B）2015 年各省份损失占地区 GDP 的比重**

指标 4.1.3：空气污染相关早逝的经济损失

主要发现：在中国，室外 $PM_{2.5}$ 污染所致过早死亡的损失逐年降低。从 GDP 占比来看，在 2015 年至 2018 年期间，该损失从 0.11%降低为 0.09%，下降了 21%。

如指标 3.3 所述，室外空气污染（$PM_{2.5}$）仍然是中国死亡率的一个主要原因。同柳叶刀倒计时全球报告不同，该指标利用指标 4.1.2 和附录第 72-75 页所述的

投入-产出模型，通过过早死亡所导致的年度劳动生产力损失，估算这种过早死亡造成的损失。当前方法学并没有完全反映空气污染相关死亡的全部损失，在未来将会进一步改进该指标的计算方法。

图 10 清楚展示了上述经济成本随着时间推移而发生的变化，与指标 3.3 中空气污染（$PM_{2.5}$）相关死亡率的变化趋势大体一致。室外 $PM_{2.5}$ 污染所致过早死亡引起的年度经济成本从 108 亿美元（2015 年）减少到 107 亿美元（2018 年），降幅为 1.1%。2018 年，该成本在中国 GDP 中占比约 0.09%，如果考虑患病人数所带来的劳动生产力损失，则该比例可能会上升到 1%。[81] 2018 年，由于产业间相互关联而引起的间接成本占总成本的 64%，主要发生在第二产业。北京周边省份，由于高能耗、高污染产业的快速发展，其 $PM_{2.5}$ 相关过早死亡的损失（从在地区 GDP 占比来看）也最高。

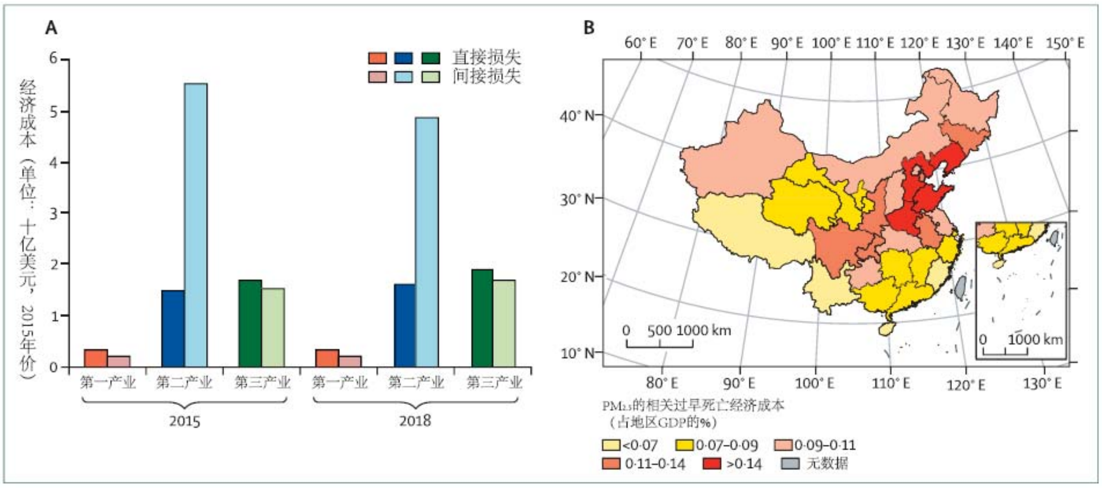

***图 10*：室外 PM2.5 污染致过早死亡造成的损失。（A）按年份和行业划分，国家层面的损失，单位：十亿美元（2015 年价）（B）2015 年各省的损失相对于 GDP 的占比**

指标 4.2：向零碳经济转型的经济分析

指标 4.2.1：健康的能源投资

主要发现：2019 年，中国对新增燃煤发电装机容量的投资继续下降，延续了 2015 年以来的下降趋势。目前，低碳能源投资已经达到煤炭投资的九倍；2019 年，可再生能源投资达到了 864 亿美元。

本指标与指标 3.1 相呼应，旨在利用国家能源局数据，追踪减缓气候变化相关的能源投资，包括煤电投资、低碳能源投资和能效投资。[83] 2008 至 2019 年之

间，中国新增燃煤发电装机容量的投资从 317 亿美元下降到了 93 亿美元。[83] 相应地，包括水电和核电在内的低碳能源投资与新建燃煤发电站投资之比从 2008 年的 1:1 快速增加至 2019 年的 9:1。2019 年，中国新能源投资达到了 864 亿美元，其中大部分为太阳能光伏投资，2017 年光伏投资峰值达到了 761 亿美元，并在接下来的两年里略有下降。[84] 2019 年，对风电的投资增加至 173 亿美元。[85] 为支撑可再生能源的扩张式发展，电网建设方面的总体投资也保持在较高水平，2018 年达到 815 亿美元，2019 年则为 716 亿美元。

指标 4.2.2：低碳和高碳行业的就业

主要发现：中国可再生能源部门的就业人数已连续两年超过化石燃料开采行业，2018 年达 410 万人。

中国在可再生能源领域的就业人数全球领先，2018 年达 410 万人，自 2012 年以来稳步上升（2017-2018 年期间，就业人数小幅下滑，下降了 2.7%）。该数字连续两年超过化石燃料开采行业的就业人数，后者在 2018 年为 388 万人，比 2017 年减少了 6%。2018 年，太阳能行业就业人数为 290 万人，占可再生能源部门就业总人数的 70.1%。中国人口占全球人口的 18.5%，而目前其可再生能源领域的就业人数占全球总量的 37%。该指标采用的数据包括 REN21 发布的《2019 年可再生能源全球状况报告（Renewables 2019 Global Status Report）》数据、Computer and Enterprise Investigations Conference 数据和中国国家统计局数据。[30,86,87]

指标 4.2.3：化石燃料补贴

主要发现：近年来，化石燃料消费补贴的下降趋势有所逆转，2018 年达到了 419 亿美元——比 2017 年增加了 10%以上，比 2015 年高出 100%以上。

化石燃料消费补贴扭曲了能源市场价格。这些补贴为化石燃料提供了不当的竞争优势，忽视了化石燃料对环境、气候和人群健康的负外部性和造成的损失。该指标利用 IEA 数据，跟踪了中国化石燃料消费补贴的绝对价值及其在全球化石燃料补贴总额中的比例。[88] 2011-2015 年期间，化石燃料补贴有所下降，但在 2018 年回升至 419 亿美元——比 2017 年增加增长了 10%，比 2015 年高出 100%以上。之所以会出现这样的逆转，是燃煤电厂补贴大幅增加的结果；近几十年来，

燃煤电厂的盈利能力严重恶化。在 IEA 报告的所有提供化石燃料补贴的国家中，2018 年，中国排名第三，仅次于全球两大石油生产国——伊朗和沙特阿拉伯。

指标 4.2.4：碳定价的范围和力度

主要发现：2019 年，碳定价机制试点项目已覆盖中国 11%的碳排放量，碳价从 0.56 美元/吨 CO_2 到 11.4 美元/吨 CO_2 不等，与实现《巴黎协定》目标尚存不小差距。

有效的碳定价是激励和引导低碳经济转型、实现《巴黎协定》目标的重要政策工具。[89] 本指标基于附录第 82-84 页所列数据和方法追踪了中国碳定价的覆盖范围和力度。[90,91] 近六年中国八个碳交易试点市场的碳价范围是 0.24-13.0 美元/吨 CO_2。实时碳价取决于各试点市场碳配额的供需情况。[91] 2019 年，北京碳市场的年均碳价最高，达 11.4 美元/吨 CO_2，而重庆和深圳年均碳价最低，仅 0.56 美元/吨 CO_2。目前中国碳交易市场的碳价仍然远低于与实现“2℃温升”控制目标相匹配的、到 2020 年 40-80 美元/吨 CO_2 的价格水平。[92] 2019 年，八个试点碳交易市场覆盖的碳排放量为 13.3 亿吨 CO_2 当量，占中国排放总量的 11%，占八个试点省份排放量的 53%。2021 年中国实行全国碳排放交易体系之后，碳定价将覆盖中国 33%的碳排放量。目前，中国所有碳排放交易试点市场仍处于发展的早期阶段，暂未产生稳定的收入。

小 结

近年来，由于中国采取大量空气污染治理的措施，因此因空气污染造成的劳动生产力损失及其经济成本均略有下降。然而，日益严重的高温和热浪所产生的经济成本不断上升，其表现形式包括死亡人数的增加和劳动生产力的降低。虽然中国已经颁布了一系列政策来促进低碳经济转型，但需要采取更果断的干预措施，如逐步取消化石燃料补贴，加强碳价格信号。否则，化石能源投资的锁定效应将对能源系统的长远发展产生负面影响。

第 5 节：公众和政府参与

尽管中国已经开始感受到气候变化对健康的影响，但国家层面或各省针对这些影响所采取的应对措施仍然不够。在某些情况下，不同的利益相关者促进了健

康和气候变化领域的公众参与，给政府施加了压力，并推动了其在减缓和适应的工作。[5,93] 本节跟踪了媒体（指标 5.1）、个人（指标 5.2）和学术界（指标 5.3）在健康和气候变化领域的参与情况。明年的报告将进一步报道中国政府在健康和气候变化领域的工作进展。

指标 5.1：社交媒体对于健康和气候变化问题的关注度

主要发现：在中国，社交媒体对健康和气候变化的关注度一直处于低位；2019 年，五大媒体的微博账号共计发布了 67 篇相关帖子。

该指标采用了一种新方法来评估媒体在健康和气候变化领域的参与情况。截至 2020 年 3 月，中国有 9.04 亿互联网用户。[94] 2019 年末，社交媒体平台新浪微博的月活跃用户数达 5.16 亿人，日活跃用户数达 2.22 亿人。[95] 根据规模、影响力和多样性（以涵盖一系列官方、商业和专业媒体），该指标选取了@人民日报、@新京报、@财新网、@健康报官方微博、@中国科学报这五个官方账号进行分析。数据来源于这些账号在新浪微博上发布的帖子，并使用柳叶刀倒计时全球报告中的关键词进行分析；关于相关方法、数据和搜索词的完整描述，可参见附录第 84-86 页。

从 2010 年到 2019 年，这五家媒体账号在新浪微博上共计发布了 7526 篇帖子探讨气候变化。年度报道量从 2010 年的 87 篇增加至 2019 年的 997 篇，增加了 11 倍（见***图 11***），相当于每天发布 2.7 篇帖子，占这五家媒体账号在所有话题上日发布总量的 2%。其中，提及公众健康的帖子占比较低，从 2010 年的 5.7% 小幅增加至 2019 年的 6.7%。2013 年，中国颁布《大气污染防治行动计划》，人们对空气污染及其与健康和气候变化的联系的认识有所提高，因此发帖量达到高峰。[96, 97] 2019 年，这五家媒体账号仅发布了 67 篇关于健康和气候变化的帖子，而健康报自 2014 年以来就不曾发布相关帖子；显然，中国需要采取更多措施，以确保媒体将公众健康与气候变化联系起来。

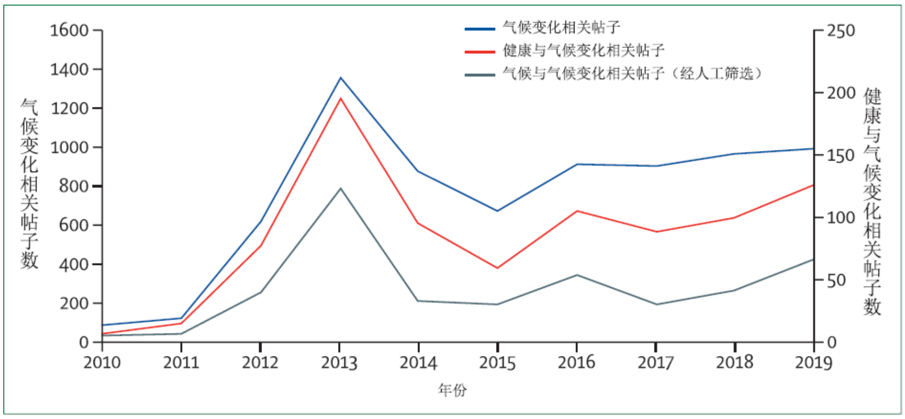

***图 11*：2008-2019 年期间新浪微博上关于气候变化以及健康和气候变化的年度报道量**

指标 5.2：公众对健康和气候变化议题的关注度

主要发现：在百度搜索中只有极少人会将公众健康与气候变化联系在一起，因此中国急需加强相关工作，以提升公众对此议题的关注度。

个人搜索偏好可表明公众对某一话题的参与程度。[98] 该指标利用关键字比对，分析了近三年来百度™上有关健康和气候变化的搜索情况。[99] 在过去十年中，百度在中国搜索引擎市场上占有超过 66%的市场份额。[100] 百度的月活跃用户数也超过了 11 亿，覆盖了中国的大部分人口。[101] 由于百度是使用最广泛的中文搜索引擎，因此百度上的搜索查询数据能够很好地反映中国的个人参与情况。与调研等其他方法相比，百度的查询数据易于收集、覆盖人群广。本研究中所有数据都是匿名的，且任何查询都不能关联到个人。关于所用关键字、相关方法和分析的完整描述和说明，可参见附录第 86-91 页。

2017-2019 年期间，气候变化搜索查询明显增加，但用户在查询时，很少共同搜索健康和气候变化这两个主题。2019 年，仅五百分之一的气候变化搜索中会关联搜索健康相关内容。而在所有健康查询中，仅有 1/200,000 的查询与气候变化有关。进一步分析表明，人口较多、经济发展水平较高的一、二线城市人口对健康和气候变化的共同搜索查询相比中国总体人口的平均水平要多一些。

指标 5.3：科学论文中对健康和气候变化议题的关注度

主要发现：2019 年，中国学者在健康和气候变化这个议题上共发表了 14 篇中文文章和 30 篇英文文章，而 2008 年分别为 12 篇和 3 篇。

该指标跟踪了中国学者通过发表科学期刊文章开展健康和气候变化问题研究的情况。该指标利用中国知网（CNKI）以及 Ovid Medline 和 Ovid Embase 数据库的高级搜索功能，检索了中国作者在科学出版物中分别发表的有气候相关关键字及其与健康相关关键字共同出现的中文文章和英文文章的情况。关于前述搜索策略的详细信息，可参见附录第 92-101 页。CNKI 共收录了 2008 年至 2019 年期间发表的 26,849 篇与气候变化相关的学术期刊文章，其中 0.83%（222 篇）与健康相关。从 2010 年到 2019 年，重点研究健康与气候变化的文章数量略有减少，2018 年时达到峰值 29 篇， 2019 年时减少到 15 篇。

而在 Ovid Embase 和 Ovid Medline 数据库中检索中国作者发表的英文文章则发现情况截然不同：2008-2019 年期间，这两个数据库共收录了 932 篇与气候相关的文章，其中 17.7%（165 篇）聚焦健康和气候变化。2008-2019 年期间，这两大数据库收录的与中国健康与气候变化相关的文章数量从 3 篇增加到了 30 篇，远不及 2019 年全球发表的 734 篇的本领域文章量。虽然进展缓慢，但中国学术界对健康和气候变化的认识正在不断提高，并且随着时间的推移还将继续提高。[102]

小 结

全社会共同关注健康和气候变化议题，是持续减缓和适应气候变化的关键。中国媒体、个人和学术界对健康和气候变化问题的参与度目前表现平平，仅某些行业的参与度出现小幅提升。这些趋势与国际趋势相左，要实现《巴黎协定》的承诺，需要媒体、学术界和公众对社会和环境系统之间的联系有更深入的了解。

结 语：《中国版柳叶刀倒计时年度报告（2020）》

首份《柳叶刀倒计时中国报告》对国家、地区和省级层面的 23 项健康与气候变化指标进行了跟踪。报告汇聚了 19 家国内外机构、77 位专家的工作成果，本报告首次全面介绍了气候变化对中国人群健康的影响以及中国所采取的应对措施。报告中的指标将不断完善，同时每年会增加新的指标和数据集。为此，本研究形成的合作平台是开放的，欢迎全国的专家和学术机构加入并开展合作研究。

显而易见，本报告跟踪的气候变化健康风险（包括高温、极端气候事件和气候敏感型疾病）在中国正迅速增加。每个人都无法逃脱气候变化对其健康的影响，而贫困人口、社会弱势群体、老年人、户外工作者等脆弱人群面临的风险尤其高。2019 年，每个老年人平均多经受了 13 个热浪天，这对他们来说可谓是巨大的健康风险。同年，全年龄段热浪相关死亡人数达到了 26,800 人，据估计，由此导致的经济损失达 136 亿美元。

中国在适应气候变化和减缓气候变化方面获得的进展喜忧参半。一方面，中国尚未出台单独的国家健康适应计划，且各省很少开展气候变化和健康风险评估。此外，煤炭使用量和化石燃料补贴的下降趋势已然逆转。各部门进展缓慢，部分是因为媒体、学术界、公众和政府参与健康和气候变化议题的程度较低。

另一方面，中国在可再生能源发展和空气污染治理方面取得了显著进展。在新增发电投资中，90%的资金都投向了非化石燃料能源。中国可再生能源行业的就业人数占全球总数的三分之一，高于其他任何国家。在中国，空气污染继续改善， $PM_{2.5}$ 相关死亡人数减少了 10%，由此产生的经济成本减少了 4 亿美元。中国正在建立覆盖全国 33%排放量的全国碳排放交易体系（ETS），预计将于 2021 年正式启动。

图 12 概述了 2000 年以来我国受到的气候变化健康影响的变化情况（上图）以及我国采取的应对措施（下图），这里选取的指标是本报告中时间序列最长、评分含义最明确的一组指标。每一项指标的分值都在 0-1 之间，每个色块中的颜色深浅对应着该指标在该年的得分情况。颜色越深，表示影响越令人担忧，或者应对措施的力度越大。比如，某年的指标得分为 1.0，代表气候变化对这个方面的健康影响在此年最为严重。对于图中的应对措施指标，如逐步淘汰煤炭、使用清洁家用能源、削减化石燃料补贴等，指数得分 1.0 则表示采取了最大力度的应对措施，比如，完全实现了煤炭的退出。大多数指标的得分远低于 1.0，突显中国在多个方面仍有较大提升空间。关于图中各指标的详细解释，可参见附录第 102-103 页，后续发布的每份报告都会更新该图，并可能在图中增加额外的指标。

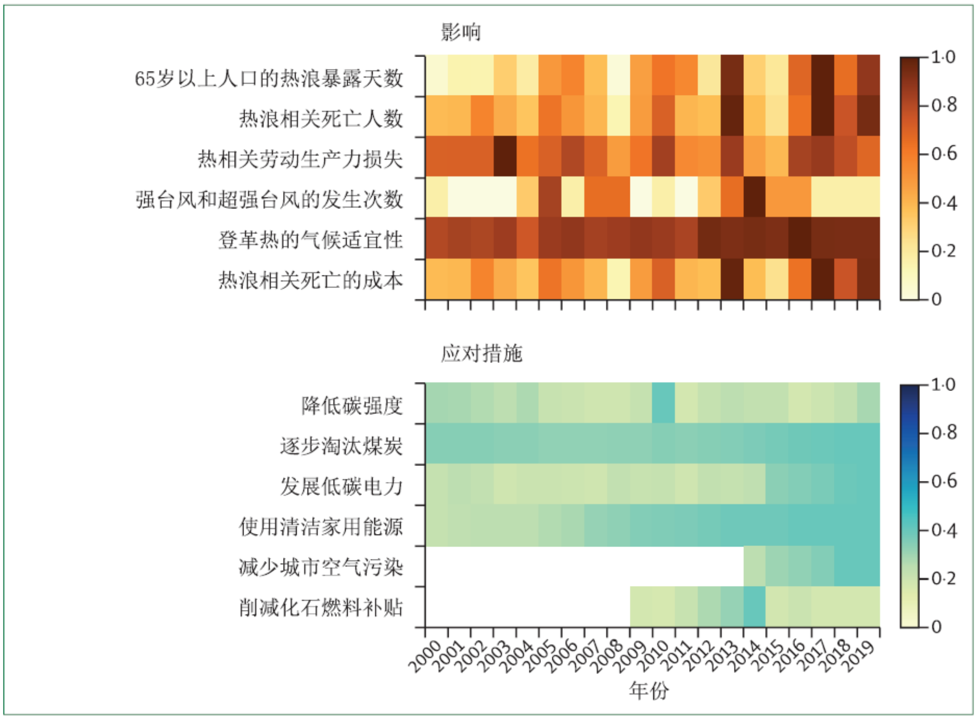

***图 12：*《柳叶刀倒计时 2020 年中国报告》指标趋势综述**

及时果断地采取措施应对气候变化将减少中国因气候变化而死亡的人数。相反，在气候行动中犹豫不决将阻碍健康中国 2030 目标[103]的实现，影响 14 亿中国人的健康与福祉。虽然新冠疫情的爆发已促使中国对社会和卫生政策进行了一系列反思、改革和调整，但仍未深入思考气候变化与健康之间的联系。如果不能做到这一点，那么新冠疫情这场健康危机的经济恢复计划可能会加剧气候变化这场健康危机的长期风险。

致 谢

我们对英国惠康基金会（Wellcome Trust）、得乐室中国（香港）有限公司（Delos China (HK) Limited）和唐仲英基金会（Cyrus Tang Foundation）对本研究提供的资金和战略支持表示感谢，没有他们的支持，我们将无法完成这项研究。以上单位并未参与本报告的设计、数据收集、分析和撰写工作。我们对陈冯富珍女士（清华大学）提出的宝贵意见和建议深表感谢。此外，感谢雷名雨、李瑞瑶和汪蕊（清华大学）对本研究提供的技术支持。

参考文献

1. Watts N, Adger WN, Agnolucci P, et al. Health and climate change: policy responses to protect public health. *Lancet* 2015; **386**(10006): 1861-914.
2. Watts N, Amann M, Arnell N, et al. The 2019 report of The *Lancet* Countdown on health and climate change: ensuring that the health of a child born today is not defined by a changing climate. *The Lancet* 2019; **394**(10211): 1836-78.
3. Promotion Committee for Healthy China Program. Healthy China Program (2019–2030). July 15, 2019. http://www.gov.cn/xinwen/2019-07/15/content_5409694.htm (accessed May 8, 2020)
4. UN Climate Change. The Paris Agreement. https://unfccc.int/process-and-meetings/the-paris-agreement/the-paris-agreement (accessed May 8, 2020).
5. The Intergovernmental Panel on Climate Change. Global warming of 1·5°C. An IPCC Special Report on the impacts of global warming of 1·5°C above pre-industrial levels and related global greenhouse gas emission pathways, in the context of strengthening the global response to the threat of climate change.2019. https://www.ipcc.ch/site/assets/uploads/sites/2/2019/06/SR15_Full_Report_High_Res.pdf (accessed April 20, 2020).
6. UN Sustainable Development Goals Knowledge Platform. Transforming our world: the 2030 agenda for sustainable development. 2015. https://sustainabledevelopment.un.org/post2015/transformingourworld (accessed April 20, 2020).
7. Mayrhuber EA-S, Dückers MLA, Wallner P, et al. Vulnerability to heatwaves and implications for public health interventions – A scoping review. *Environmental Research* 2018; **166**: 42-54.
8. Copernicus Climate Change Service. ERA5. https://www.ecmwf.int/en/forecasts/datasets/reanalysis-datasets/era5 (accessed April 23, 2020).
9. Chambers J. Hybrid gridded demographic data for the world, 1950–2020. April 27, 2020. https://zenodo.org/record/3768003#.X4spwZ5KiUk (accessed April 27, 2020).
10. Yang J, Yin P, Sun J, et al. Heatwave and mortality in 31 major Chinese cities: definition, vulnerability and implications. Sci Total Environ 2019; 649: 695–702.
11. Xu Z, FitzGerald G, Guo Y, Jalaludin B, Tong S. Impact of heatwave on mortality under different heatwave definitions: A systematic review and meta-analysis. *Environment International* 2016; **89-90**: 193-203.
12. Ma W, Chen R, Kan H. Temperature-related mortality in 17 large Chinese cities: how heat and cold affect mortality in China. *Environ Res* 2014; **134**: 127-33.
13. Wang Y, Wang A, Zhai J, et al. Tens of thousands additional deaths annually in cities of

China between 1.5 C and 2.0 C warming. *Nature communications* 2019; **10**: 3376
14. Li T, Ban J, Horton RM, et al. Heat-related mortality projections for cardiovascular and respiratory disease under the changing climate in Beijing, China. *Scientific reports* 2015; **5**: 11441.
15. Kjellstrom T, Freyberg C, Lemke B, Otto M, Briggs D. Estimating population heat exposure and impacts on working people in conjunction with climate change. *Int J Biometeorol* 2018; **62**(3): 291-306.
16. Dunne JP, Stouffer RJ, John JG. Reductions in labour capacity from heat stress under climate warming. *Nat Clim Chang* 2013; **3**(6): 563-6.
17. Watts N, Amann M, Arnell N, et al. The 2018 report of the Lancet Countdown on health and climate change: shaping the health of nations for centuries to come. *The Lancet* 2018; **392**(10163): 2479-514.
18. National Aeronautics and Space Administration. Active Fire Data. 2020. https://earthdata.nasa.gov/active-fire-data (accessed May 23, 2020).
19. Doocy S, Dick, A., Daniels, A., & Kirsch, T. D. . The human impact of tropical cyclones: a historical review of events 1980-2009 and systematic literature review. *Plos Currents* 2013; **5:5**.
20. Zheng J, Han W, Jiang B, Ma W, Zhang Y. Infectious Diseases and Tropical Cyclones in Southeast China. *International journal of environmental research and public health*; **14.5 (2017): 494.**
21. Jiao K, Hu W, Ren C, Xu Z, Ma W. Impacts of tropical cyclones and accompanying precipitation and wind velocity on childhood hand, foot and mouth disease in Guangdong Province, China. *Environmental Research* 2019; **173**(JUN.): 262-9.
22. Lowe SR, Joshi S, Pietrzak RH, Galea S, Cerdá M. Mental health and general wellness in the aftermath of Hurricane Ike. *Soc Sci Med* 2015; **124**: 162-70.
23. Scaramutti C, Salas-Wright CP, Vos SR, Schwartz SJ. The Mental Health Impact of Hurricane Maria on Puerto Ricans in Puerto Rico and Florida. *Disaster Med Public Health Prep* 2019; **13**(1): 24-27.
24. Taioli E, Tuminello S, Lieberman-Cribbin W, et al. Mental health challenges and experiences in displaced populations following Hurricane Sandy and Hurricane Harvey: the need for more comprehensive interventions in temporary shelters. *J Epidemiol Community Health* 2018; **72**(10): 867-70.
25. Ying M, Zhang W, Yu H, et al. An Overview of the China Meteorological Administration Tropical Cyclone Database. *Journal of Atmospheric & Oceanic Technology* 2014; **31**(2): 287-301.
26. China Meteorological Administration Tropical Cyclone Data Centre. Introduction to tropical cyclone data of China meteorological administration. 2020. http://tcdata.typhoon.org.cn/ (accessed May 23, 2020).

27. Rocklöv J, Tozan Y. Climate change and the rising infectiousness of dengue. *Emerging Topics in Life Sciences* 2019; **3**:133-42.
28. Institute for Health Metrics and Evaluation. Global burden of disease study 2017 (GBD 2017) data resources. http://ghdx.healthdata.org/gbd-2017 (April 23, 2020).
29. Yao-Dong D, Xian-Wei W, Xiao-Feng Y, Wen-Jun M, Hui A, Xiao-Xuan W. Impacts of climate change on human health and adaptation strategies in South China. *Advances in climate change research* 2013; **4**(4): 208-14.
30. National Bureau of Statistics of China. National and provincial vehicle ownership. 2020. http://data.stats.gov.cn/english/ (accessed May 5, 2020).
31. Chan EYY, Ho JY, Hung HHY, Liu SD, HCY L. Health impact of climate change in cities of middle-income countries: the case of China. *Br Med Bull* 2019; **130**(1): 5-24.
32. National Development and Reform Commission. National Climate Change Program. June 4, 2007. http://www.china.org.cn/english/environment/213624.htm (accessed May 5, 2020).
33. WHO. Health and climate Change Country Profiles 2015/2016 cycle. https://www.who.int/globalchange/resources/country-profiles/en/ (accessed May 8, 2020).
34. WHO. WHO Health and climate change survey report: tracking global progress. 2019. https://www.who.int/globalchange/publications/country-profiles-global-report-2019/en/ (accessed April 23, 2020).
35. Qin D. Climate and Environmental Evolution in China: 2012 (in Chinese). Beijing: China Meteorological Press, 2012.
36 Writing Committee of The Third National Assessment Report on Climate Change. The Third National Assessment Report on Climate Change (in Chinese). Beijing: Science Press, 2015.
37 Wang W, Zheng G, Pan J, Chao Q, Wang M, Hu G. Green book of climate change-annual report on actions to address climate change: 2014 (in Chinese). Beijing: Social Sciences Academic Press, 2014.
38 Xie F, Liu Y, Chao Q, Zhuang G, Hu G, Pan J. Green book of climate change-annual report on actions to address climate change: 2019 (in Chinese). Beijing: Social Sciences Academic Press, 2019.
39 Zhong S, Huang C. Climate change and human health: risks and responses (in Chinese). Chin Sci Bull 2019; 64: 2002–10.
40 WHO. Operational framework for building climate resilient health systems. 2015. https://www.who.int/globalchange/publications/building-climate-resilient-health-systems/en/ (accessed April 20, 2020).
41 Tang K, Gong P, Zhang WZ, Lin P. Report of check-up for China's cities (2019) (in Chinese). Beijing: China City Press, 2020.
42. Bouchama A, Dehbi M, Mohamed G, Matthies F, Shoukri M, Menne B. Prognostic factors in heat wave related deaths: a meta-analysis. *Archives of internal medicine* 2007; **167**(20): 2170-76.

43. Cheong KW, Riffat SB. Monitoring hydrofluorocarbon refrigerant leakage from air-conditioning systems in buildings. *Applied Energy* 1996; **53**(4): 341-7.
44. Vandentorren S, Bretin P, Zeghnoun A, et al. August 2003 heat wave in France: risk factors for death of elderly people living at home. *The European Journal of Public Health* 2006; **16**(6): 583-91.
45. Richard L, Kosatsky T, Renouf A. Correlates of hot day air-conditioning use among middle-aged and older adults with chronic heart and lung diseases: the role of health beliefs and cues to action. *Health education research* 2011; **26**(1): 77-88.
46. Zhang Q, Zheng YX, Tong D, et al. Drivers of improved PM2.5 air quality in China from 2013 to 2017. *Proceedings of the National Academy of Sciences of the United States of America* 2019; **116**(49): 24463-9.
47. Zhang S, Worrell E, Crijns-Graus W, et al. Modeling energy efficiency to improve air quality and health effects of China's cement industry. *Applied Energy* 2016; **184**: 574-93.
48. Markandya A, Sampedro J, Smith SJ, et al. Health co-benefits from air pollution and mitigation costs of the Paris Agreement: a modelling study. *Lancet Planetary Health* 2018; **2**(3): E126-E33.
49. Kan H, Chen R, Tong S. Ambient air pollution, climate change, and population health in China. *Environment International* 2012; **42**: 10-9.
50. Gallagher KS, Zhang F, Orvis R, Rissman J, Liu Q. Assessing the Policy gaps for achieving China's climate targets in the Paris Agreement. *Nat Commun* 2019; **10**(1): 1256.
51 UN Environment Programme. Emission Gap Report 2019. 2019. https://www.unenvironment.org/interactive/emissions-gap-report/2019/ (accessed April 23, 2020).
52 Department of Energy Statistics in National Bureau of Statistics. China Energy Statistical Yearbook 2001–2018. Beijing: China Statistics Press, 2002–19.
53 Statistical Bulletin of National Economic and Social Development 2018–2019. National Bureau of Statistics of China. 2019–2020. http://www.stats.gov.cn/tjsj/zxfb/202002/t20200228_1728913.html (accessed April 23, 2020).
54 Shan Y, Guan D, Zheng H, et al. China CO2 emission accounts 1997-2015. Sci Data 2018; 5: 170201.
55 Liu Z, Ciais P, Deng Z, et al. Near-real-time monitoring of global CO_2 emissions reveals the effects of the COVID-19 pandemic. Nat Commun 2020; 11: 5172.
56 Giani P, Castruccio S, Anav A, Howard D, Hu W, Crippa P. Short-term and long-term health impacts of air pollution reductions from COVID-19 lockdowns in China and Europe: a modelling study. Lancet Planet Health 2020; 4: e474–82.
57 State Council of China. Notice of the State Council on issuing the air pollution prevention and control action plan. 2013. http://www.gov.cn/zwgk/2013-09/12/content_2486773.htm

(accessed May 5, 2020).

58 State Council of China. State Council's issue of winning the blue sky defense war notice of three-year action plan. 2018. http://www.gov.cn/zhengce/content/2018-07/03/content_5303158.htm (accessed May 5, 2020).

59 Venkataraman C, Sagar AD, Habib G, Lam N, Smith KR. The Indian National Initiative for Advanced Biomass Cookstoves: the benefits of clean combustion. Energy Sustain Dev 2010; 14: 63–72.

60 Wu SM, Zheng XY, Wei C. Measurement of inequality using household energy consumption data in rural China. Nat Energy 2017; 2: 795–803

61 Building Energy Conservation Research Center of Tsinghua University. 2017 annual report on China building energy efficiency. Beijing: China Building Industry Press, 2017.

62 Data Center of Ministry of Ecology and Environment of China. Daily air quality of Chinese cities. https://datacenter.mee.gov.cn/websjzx/queryIndex.vm (April 20, 2020).

63 Amann M, Bertok I, Borken-Kleefeld J, et al. Cost-effective control of air quality and greenhouse gases in Europe: modeling and policy applications. Environ Model Softw 2011; 26: 1489–501.

64 Huang J, Pan X, Guo X, Li G. Health impact of China's air pollution prevention and control action plan: an analysis of national air quality monitoring and mortality data. Lancet Planet Health 2018; 2: e313–23.

65 Yang S, Fang D, Chen B. Human health impact and economic effect for PM2·5 exposure in typical cities. Appl Energy 2019; 249: 316–25.

66 Maji KJ, Ye WF, Arora M, Shiva Nagendra SM. PM2.5-related health and economic loss assessment for 338 Chinese cities. Environ Int 2018; 121: 392–403.

67 Yu S, Xia J, Yan Z, et al. Loss of work productivity in a warming world: differences between developed and developing countries. J Clean Prod 2019; 208: 1219–25.

68 Horton DE, Skinner CB, Singh D, Diffenbaugh NS. Occurrence and persistence of future atmospheric stagnation events. Nat Clim Chang 2014; 4: 698–703.

69 Chen K, Fiore AM, Chen R, et al. Future ozone-related acute excess mortality under climate and population change scenarios in China: a modeling study. PLoS Med 2018; 15: e1002598.

70 Ministry of Ecology and Environment of the People's Republic of China. Limits and measurement methods for emissions from light-duty vehicles (CHINA 5). 2016. http://english.mee.gov.cn/Resources/standards/Air_Environment/emission_mobile/201605/t20160511_337517.shtml (May 10, 2020).

71 The Ministry of Public Security of the People's Republic of China. National private vehicle ownership. 2020. https://www.mps.gov.cn/n2254314/n6409334/c6852472/content.html (accessed May 10, 2020).

72 He G, Pan Y, Tanaka T. The short-term impacts of COVID-19 lockdown on urban air pollution in China. Nat Sustain 2020; published online July 7. https://doi.org/10.1038/s41893-020-

0581-y.
73 Giani P, Anav A, De Marco A, Feng Z, Crippa P. Exploring sources of uncertainty in premature mortality estimates from fine particulate matter: the case of China. Environ Res Lett 2020; 15: 064027.
74 Lv Z, Wang X, Deng F, et al. Significant reduced traffic in Beijing failed to relieve haze pollution during the COVID-19 lockdown: implications for haze mitigation. arXiv 2020; published online June 12. https://doi.org/2006.07297v1 (preprint).
75 Huang X, Ding A, Gao J, et al. Enhanced secondary pollutionoffset reduction of primary emissions during COVID-19 lockdown in China. Natl Sci Rev 2020; published online June 18. https://doi.org/10.1093/nsr/nwaa137.
76 Xie Y, Dai H, Dong H, Hanaoka T, Masui T. Economic impacts from PM2·5 pollution-related health effects in China: a provincial-level analysis. Environ Sci Technol 2016; 50: 4836–43.
77 Tian X, Dai H, Geng Y, et al. Toward the 2-degree target: evaluating co-benefits of road transportation in China. J Transp Health 2019; 15: 100674.
78 Zhang X, Jin Y, Dai H, Xie Y, Zhang S. Health and economic benefits of cleaner residential heating in the Beijing–Tianjin–Hebei region in China. Energy Policy 2019; 127: 165–78.
79 Partridge I, Gamkhar S. A methodology for estimating health benefits of electricity generation using renewable technologies. Environ Int 2012; 39: 103–10.
80 Xia Y, Guan D, Jiang X, Peng L, Schroeder H, Zhang Q. Assessment of socioeconomic costs to China's air pollution. Atmos Environ 2016; 139: 147–56.
81 Xia Y, Guan D, Meng J, Li Y, Shan Y. Assessment of the pollution-health-economics nexus in China. Atmos Chem Phys 2018; 18: 14433–43.
82 Xia Y, Li Y, Guan D, et al. Assessment of the economic impacts of heat waves: a case study of Nanjing, China. J Clean Prod 2018; 171: 811–19.
83 National Energy Administration. The National Energy Administration releases 2019 national power industry statistics. Jan 20, 2020. http://www.nea.gov.cn/2020-01/20/c_138720881.htm (April 20, 2020).
84 National Bureau of Statistics of China. China Statistical Year Book 2019 (in Chinese). Beijing: China Statistics Press, 2020.
85 Energy Research Institute of Academy of Macroeconomic Research, NDRC China National Renewable Energy Centre. China renewable energy outlook 2019. 2020. https://www.dena.de/fileadmin/dena/Publikationen/PDFs/2019/CREO2019_-_Executive_Summary_2019.pdf (May 8, 2020).
86 Murdock HE, Gibb D, André T, et al. Renewables 2019 Global Status Report. 2019. https://www.ren21.net/wp-content/uploads/2019/05/gsr_2019_full_report_en.pdf (May 5, 2020).
87 Computer and Enterprise Investigations Conference. China employment in fossil fuel extraction CEIC global economic data, indicators, charts & forecasts. 2012–2019

https://www.ceicdata.com/zh-hans/china/no-of-employee-by-industry-monthly/no-of-employee-petroleum-coking--nuclear-fuel; https://www.ceicdata.com/zh-hans/china/no-of-employee-by-industry-monthly/no-of-employee-coal-mining--dressing (May 7, 2020).

88 International Energy Agency. Fossil-fuel consumption subsidies by country, 2018. 2020. https://www.iea.org/data-and-statistics/charts/fossil-fuel-consumption-subsidies-by-country-2018 (April 28, 2020).

89 Zapf M, Pengg H, Weindl C. How to Comply with the Paris agreement temperature goal: global carbon pricing according to carbon budgets. Energies 2019; 12: 2983.

90 European Union Joint Research Centre. CO2 time series 1990-2015 per region/country. 2016. https://edgar.jrc.ec.europa.eu/overview.php?v=CO2ts1990-2015 (April 26, 2020).

91 The World Bank. Carbon pricing dashboard. 2019. https://carbonpricingdashboard.worldbank.org/ (April 26, 2020).

92 Carbon Pricing Leadership Coalition. Report of the high-level commission on carbon prices. May 29, 2017. https://static1.squarespacecomstatic/54ff9c5ce4b0a53decccfb4c/t/59b7f2409f8dce5316811916/1505227332748/CarbonPricing_FullReport.pdf (April 26, 2020).

93 Wan H, Bian J, Zhang H, Li Y. Assessment of future climate change impacts on water-heat-salt migration in unsaturated frozen soil using CoupModel. Front Environ Sci Eng 2020; 15: 1–17.

94 China Internet Network Information Center. Statistical report on internet development in China. April, 2020. http://cnnic.com.cn/IDR/ReportDownloads/202008/P020200827549953874912.pdf (accessed May 25, 2020).

95 Sina Technology. Weibo monthly active users reach 516 million, and barriers to competition remain solid. Feb 26, 2020. https://tech.sina.com.cn/i/2020-02-26/doc-iimxxstf4598954.shtml (May 4, 2020).

96 Ojekunle Z, Oyebamji F, Olatunde A, et al. Global climate change:the empirical study of the sensitivity model in China's sustainable development, part 2. Energ Source Part A 2015; 37: 861–69.

97 Xu Z, Zhao C, Feng Z, et al. Estimating realized and potential carbon storage benefits from reforestation and afforestation under climate change: a case study of the Qinghai spruce forests in the Qilian Mountains, northwestern China. Mitig Adapt Strategies Glob Change 2013; 18: 1257–68.

98 Archibald CL, Butt N. Using Google search data to inform global climate change adaptation policy. Clim Change 2018; 150: 447–56.

99 Vaughan L, Chen Y. Data mining from web search queries: a comparison of google trends and baidu index. J Assoc Inf Sci Technol 2015; 66: 13–22.

100 StatCounter GlobalStats. Search engine market share China. 2020.

https://gs.statcounter.com/search-engine-market-share/all/china2020 (May 15, 2020).
101 Baidu. Baidu announces first quarter 2019 results. May 16, 2019. http://ir.baidu.com/news-releases/news-release-details/baidu-announces-first-quarter-2019-results (accessed May 9, 2020).
102 Bi P, Shi X-M, Liu, Q-Y. Climate change and population health research in China: knowledge gaps and further directions. Adv Clim Chang Res 2020; 11: 273–78.
103 Chinese Central Government. Health China Action (2019-2030). July 15, 2019. http://www.gov.cn/xinwen/2019-07/15/content_5409694.htm (accessed May 5, 2020).

.
