# Supplementary material for: The 2020 China report of the Lancet Countdown on health and climate change
Source: Lancet Public Health. 2020 Dec 2;6(1):e64–81. doi: 10.1016/S2468-2667(20)30256-5 (PMC7966675; doi:10.1016/S2468-2667(20)30256-5)
Supplement: Supplementary appendix 2 [file mmc2.pdf]

THE LANCET
Public Health

Supplementary appendix 2

This appendix formed part of the original submission and has been peer reviewed. We post it as supplied by the authors.

Supplement to: Cai W, Zhang C, Suen HP, et al. The 2020 China report of the *Lancet* Countdown on health and climate change. *Lancet Public Health* 2020; published online Dec 2. https://doi.org/10.1016/S2468-2667(20)30256-5.

The 2020 Chinese Report of The Lancet Countdown on Health and Climate Change

Appendix

Structure of indicators and the selection criteria

This Chinese report is the regional spin-off of the global Lancet Countdown report.[1] The definition of indicators and the selection criteria were described in the previous global reports. For the audiances who are not familiar with the global Lancet Countdown report, we reiterate the structure of indicators and the selection criteria here.

As denoted in the global Lancet Countdown reports, the global and Chinese Lancet Countdown report aims to "track progress on health and climate change and publish annual updates of the indicators" across five key domains: 1.The health impacts, exposures and vulnerabilities of climate change; 2.Adaptation, planning and resilience for health; 3.Mitigation actions and their health co-benefits; 4.Economic and financial aspects of the interaction between climate change and health; 5.Public and political engagement in climate change and health. Each following report has been an iteration of the previous, with new indicators introduced and improved methodologies, naming and categorizing of indicators in order to fill the indicator gaps of each domain.

The indicators are intended to be grouped by the problem they focus on and/or the solution they lead to. For the first domain, the indicators are grouped within different climate change-health pathways: heat, extreme weather events, infectious diseases (and in the global report also food security and migration). The second domain is structured around the WHO Operational Framework for Climate Resilient Health Systems. The global report also covers the areas of climate information services and health adaptation spending. Within the third domain, indicators are grouped by sector, with air pollution as a co-benefit and a separate indicator, as it spans across sectors. The global report also has indicators on agriculture and healthcare mitigation. Within the fourth domain, the indicators are grouped into two areas: the health and economic costs of climate change and benefits from mitigation, and the economics of the transition to zero-carbon economies. In the global report, the final domain is organized into five different areas of engagement: media, individual, scientific, government and corporate.

The selection criterias of the indicators include: 1.Track an aspect of the relationship between health and climate change, well evidenced in the literature and not adequately covered through other indicators in the report; 2.Utilise data from a reliable source, available at adequate temporal and spatial scales to enable globe/regional trends to be observed at a global/regional level; 3.Be updatable periodically, ideally annually. In this year's Chinese report, one new indicator that is unique to China's characteristics and fulfill the forementioned three criterias has been included (indicator 1.2.2-cyclones).

The current suite of indicators in the Chinese Lancet Countdown report is not fully comprehensive due to limited word count and limited time to develop this report. But they will be improved to cover a wider range of important impacts and interventions in future reports.

Important notes about the additional indicators in the appendix

The 2020 China Lancet Countdown report is the first annual report that documents indicators of progress on health and climate change. Although the findings of 23 indicators are reported in the main text, 34 indicators went through a process of development this year. Some indicators reflect the indicator structure of the global 2020 Lancet Countdown report, but were merged into single indicators in the main text. These include indicators 2.1.1 and 2.1.2, and 3.1.1 to 3.1.3, which are reported in the main text as 2.1 and 3.1 respectively. The methods and data of these lower-level indicators are displayed here in the appendix.

A further seven indicators (as shown in **Table 1**) reflect the indicators of the global 2020 Lancet Countdown report and a comprehensive assessment of health and climate change in China. Initial methods, data and findings of these indicators are reported here in the appendix and these indicators (along with the other 23

reported in the main text) will undergo further development and improvement, with their findings reported in full in 2021.

Table 1: Additional indicators under development and reported in the appendix

Indicator number	Name of indicator
1.1.4	Health and exposure to warming
1.1.5	Vulnerability to extremes of heat
1.2.3	Flood and drought
2.1.3	City-level climate change risk assessments
2.3	Climate information services for health
3.5	Food, agriculture, and health
4.1.4	Economic losses due to climate-related extreme events

Section 1: Climate change impacts, exposures, and vulnerability

Indicator 1.1: Health and heat

Indicator 1.1.1: Exposure of vulnerable populations to heatwaves

Methods

This indicator compared changes in heatwave exposure among the elderly population in China, using temperature data from the European Centre for Medium-Range Weather Forecasts, ERA5 reanalysis dataset,[2] and population count from a hybrid gridded demographic dataset, provided by Chambers (2020),[3] that combines the NASA Socioeconomic Data and Applications Center (SEDAC) Gridded Population of the World (GPWv4) and the Inter-Sectoral Impact Model Intercomparison Project (ISIMIP) Histsoc dataset. The methodology for this indicator is similar to methodology described in the 2020 global Lancet Countdown reports, but the definition of heatwave in the China report is different from the global report. The 92.5th percentile of daily maximum temperature over the warm season (May 1st to Sep 30th) between 1986 and 2005 was computed as reference, and a heatwave event was defined as a period of three or more days where the daily maximum temperature was higher than the reference at a given grid. The days of heatwave were defined as the number of days within the heatwave event.

Here we made some explaination about why we chose a definition that is different from the global report. Actually, defining heatwave remains a highly controversial topic.[4] An extremely strict heatwave definition (e.g., at least four consecutive days with daily maximum temperature ⩾ 99th percentile) may underestimate the heat-related deaths and could not protect the public health efficiently because moderate heatwave (e.g., at least two consecutive days with daily maximum temperature ⩾ 90th percentile) may have already caused considerable number of deaths, while a loose heatwave definition may activate the heatwave early warning too early and too frequently, and cause inconvenience to the public and waste health resources.

Previous studies in different regions have adopted different heatwave definitions. For instance, Tian et al. found that heatwave definition using 97.5th percentile of daily mean temperature and duration ⩾ 2 days performed best on estimating the effect of heatwave on mortality from coronary heart disease in Beijing;[5] Chen et al. reported that heatwave defined as ⩾4 consecutive days with daily mean temperature > 98th percentile was the most appropriate definition to assess the influence of heatwave added effect on mortality in Nanjing;[6] Daniel et al. found that heatwave defined as ⩾2 consecutive days with daily maximum apparent temperatures > 95th percentile was the most appropriate definition in Rome and Stockholm.[7]

Generally, these studies were carried out only in a single or limited number of cities, and it is unsuitable to

generalize these identified heatwave definitions directly to other cities. To determine which heatwave definition could best capture the health impact of heatwave in China, Yang et al. compared the goodness of model fits among 15 heatwave definitions using the Akaike Information Criterion for quasi-Poisson (Q-AIC).[8] In detail, they summed Q-AIC values for each heatwave definitions from all group-specific mortality in 31 capital cities of China; and the minimal sum of Q-AIC produced the best model fit and the best heatwave definition. Finally, they found that heatwave definition as at least 3 consecutive days with daily maximum temperature ⩾ 92.5th percentile performed the best model fit at the national scale, as the Q-AIC under this definition was much smaller than others (Table 2). Therefore, although different from the global definition, we believe this definition is the best and most appropriate one to estimate the health impact of heatwave at the national level for China.

Table 2. Sum values of Akaike's Information Criteria for quasi-Poisson (Q-AIC) for 31 Chinese cities, using daily maximum temperature in warm season (May-September) for heat wave definitions.

Heatwave	Definition	QAIC
HW01	Daily maximum temperature ≥ 90.0th percentile for two or more consecutive day	1893057
HW02	Daily maximum temperature ≥ 90.0th percentile for three or more consecutive day	1892853
HW03	Daily maximum temperature ≥ 90.0th percentile for four or more consecutive day	1892960
HW04	Daily maximum temperature ≥ 92.5th percentile for two or more consecutive day	1892955
HW05	Daily maximum temperature ≥ 92.5th percentile for three or more consecutive day	**1892623**
HW06	Daily maximum temperature ≥ 92.5th percentile for four or more consecutive day	1893024
HW07	Daily maximum temperature ≥ 95.0th percentile for two or more consecutive day	1893082
HW08	Daily maximum temperature ≥ 95.0th percentile for three or more consecutive day	1893081
HW09	Daily maximum temperature ≥ 95.0th percentile for four or more consecutive day	1893423
HW10	Daily maximum temperature ≥ 97.5th percentile for two or more consecutive day	1893215
HW11	Daily maximum temperature ≥ 97.5th percentile for three or more consecutive day	1893496
HW12	Daily maximum temperature ≥ 97.5th percentile for four or more consecutive day	1893758
HW13	Daily maximum temperature ≥ 99.0th percentile for two or more consecutive day	1893654
HW14	Daily maximum temperature ≥ 99.0th percentile for three or more consecutive day	1893790
HW15	Daily maximum temperature ≥ 99.0th percentile for four or more consecutive day	1893981

(Source: Yang et al. 2019)

Instead of year-round ambient temperature, most of previous studies only included the warm season (e.g., 1 May to 30 September) or summer-time ambient temperature in heatwaves on population health analyses [4,6,7,9]. Only using the warm season data can effectively remove the confounding effect of cold temperature, and also adjust the effect of moderate hot temperature to avoiding the overestimation of the risk of heatwave. Therefore, , we also adopted the warm season ambient temperature in this study.

The gridded 92.5th percentile of daily maximum temperature was calculated for 1986 - 2005 with a resolution of 0.5°. For each year from 2000 to 2019, the number of heatwave events and total days of heatwaves per year was calculated according to the definition.

The vulnerable population was defined as people aged over 65. The heatwave exposure of the vulnerable population was calculated in person-days, i.e. the number of heatwave days multiplied by the exposed population over 65, at each grid. Provincial-level and country level exposure were computed by summing over corresponding cell data. The heatwave exposure from 1986 to 2005 was averaged to be the baseline data. For each year from 2000 to 2019, the change in heatwave exposures relative to the baseline was assessed. It was divided by the elder population to obtain the exposure per elder person.

Due to the increase of frequency/intensity of heatwaves, the per person heatwave exposure is showing an increasing trend. Therefore, the spatial differences in the per person exposure shown in the figure is mainly due to the spatial distribution differences of changes of heatwave days. The total heatwave exposure (mentioned in the text) is also showing an increasing trend. It is not only associated with increase of heatwaves, but also with the aging of population.

Data

1. Climate data was taken from European Centre for Medium-Range Weather Forecasts (ECMWF), ERA5 project.[2]

2. Population data from the hybrid gridded demographic data for the world was taken from Chambers (2020).[3]

Caveats

The definition of vulnerable population (above the age of 65) does not include vulnerable groups that are vulnerable due to limited access to healthcare, poor health status, low socioeconomic status and etc. As described in the global Lancet Countdown 2020 report, there may be some inconsistencies with the population data due to the use of two distinct data sources.

The caveats associated with the definition of a heatwave are described in indicator 1.1.2.

Future Form of Indicator

Future indicator may define vulnerable population based on socioeconomic status, healthcare accessibility (e.g., number of hospital beds per unit number of people), and prevalence of medical conditions that increase risk of morbidity and mortality from heatwaves, such as cardiovascular and respiratory conditions.

Additional Information

The differences of results between the 2020 global report and the China report can be explained. Based on the definition of a heatwave event in the 2020 global report (99th percentile of minimum temperature), the heatwave exposure of 65+ population in 2019 would be 0.5 billion in China. If based on the definition in the China report (92.5th percentile of maximum temperature), then the number of heatwave exposure was 2.82 billion in 2019 (about 5.6 times of that using the old definition). So there was indeed a significant difference between the heatwave event and the heatwave exposure assessed in person days between the global and Chinese report.

Indicator 1.1.2: Heatwave-related mortality

Methods

The heatwave definition and the rationale of choosing this definition has been described in details in indicator 1.1.1 The attributable number (AN) of deaths associated with heatwave is calculated. The method is as follows:

$$\boldsymbol{AN = Pop_{y,p} \times Mort_{y,p} \times HW_{y,p} \times AF_p}$$

Where $Pop_{y,p}$ refers to the grid cell-level population size in a specific year; $\text{Mort}_{y,p}$ is the baseline daily non-accidental mortality rate; the mortality rate from China Statistical Yearbook is divided by 365 as a pre-process

because it is an annual statistic. $HW_{y,p}$ is the heatwave days in a specific year. AF_p is the attributable fraction (AF), which is calculated as:

$$AF = \frac{RR - 1}{RR}$$

Where RR represents the increase in the risk of mortality resulting from heatwave, compared with non-heatwave. The daily death data of urban residents in 31 provincial capital cities of China and the daily weather data is used to calculate the exposure-response relationships, using Poisson generalized linear models. For detailed information, please refer to Yang et al. (2019).[4]

The exposure-response relationship between heatwave and mortality in different provinces (autonomous regions) is represented by the related capital cities in mainland China, and the relationship is assumed to be consistent during the study period. The annual heat-related deaths in a specific province (municipality or autonomous regions) is the sum of the calculated annual heat-related deaths in the grid cells belong to this province (municipality or autonomous regions), and the annual heatwave-related deaths in mainland China is the sum of the deaths of the total grid cells.

Data

1. RR values are derived from Yang et al, (2019).[4]
2. Mortality rates at province levels ($\boldsymbol{Mort_{y,p}}$) are derived from China Statistical Yearbook.
3. Gridded climate data was from the European Centre for Medium-Range Weather Forecasts (ECMWF), ERA5 project.[2]
4. Population data was from the Chambers (2020) hybrid gridded demographic data for the world.[3]
5. Population structure data was from United Nations-World Population Prospects.[10]

Caveats

First, only limited number of exposure-response functions were used for such a big country like China. Second, the exposure-response functions, which can be considered as the effects of high temperature and heatwave on mortality, were assumed to be constant for the past 30 years. This might create an estimation bias. In fact, along with the aging process, people's increasing adaptation ability, the popularity of air conditioning and other potential factors, the exposure-response function might also have changed in the past 30 years. However, due to lack of investigation into this field, for now, we assume the exposure-response functions remains constant.

Future Form of Indicator

One possible improvement of this indicator would be to use further city-level exposure-response function parameters within each province.

Indicator 1.1.3: Change in labour capacity

Methods

Firstly, gridded (0.5°) daily temperature, dew point temperature and relative humidity were used to calculate wet bulb globe temperature (WBGT) in the shade. The calculation method of WBGTmax, WBGTmean and WBGThalf is the same as Watts et al.[1]
Secondly, the fraction of work hours lost (WHL) in each industry was estimated by the loss function from Watts et al.[1]

$$loss\ fraction = \frac{1}{2}\left(1 + \mathrm{ERF}\left(\frac{\mathrm{WBGT} - \mathrm{Prod_{mean}}}{\mathrm{Prod_{sd}}}\right)\right)$$

WBGT refers to the WBGTmax, WBGTmean and WBGThalf estimated in the first step. $\mathrm{Prod_{mean}}$ and $\mathrm{Prod_{sd}}$ refer respectively to the mean and standard deviation of labour loss fraction. They are the fixed parameters for labourers working with different activity levels. Their respective values are displayed in **Table 3**. Labour is commonly divided into engaging in the primary, secondary and tertiary industry in China.[1] The primary industry refers to agricultural, forestry, animal husbandry and fishery, the secondary industry includes manufacturing, construction, mining and utilities, and the tertiary industry includes transport, trade, catering services, finance, real estate and other services.[11] Labour in the primary industry was assumed to work at a metabolic rate of 400w, the secondary industry was at 300w and the tertiary industry was at 200w. After estimating the loss fractions in each industry working at different levels of WBGT, these fractions were clipped at both extremes using the same method as Watts et al.[1]

Table 3: Input values for labour loss fraction[1]

Work level	$\mathbf{Prod_{mean}}$	$\mathbf{Prod_{sd}}$
200w	35.53	3.94
300w	33.49	3.94
400w	32.47	4.16

Note: w is short for watts. Work levels at 200w/300w/400w represent light/medium/heavy physical work respectively.

Thirdly, a labourer was assumed to work 8 hours a day (2 hours at WBGTmean, 2 hours at WBGTmax and 4 hours at WBGThalf), as 8 hours is the legal working time stipulated by the Labour Law of China.[12] Based on the loss function and the above assumption, the daily loss of each person in each industry was estimated. This was then multiplied by the number of people working in each industry to obtain daily losses in each industry. Finally, the total WHL in each industry from 2000-2019 was estimated by summing these daily losses.

Data

1. Gridded climate data was from the European Centre for Medium-Range Weather Forecasts (ECMWF), ERA5 project.[2]
2. Population data was from the hybrid gridded demographic data for the world by Chambers (2020).[3]
3. Data on the percentage of people working in each industry in the previous years was from 2019 Statistical Yearbook of China.[13]

Caveats

The loss function was used to estimate WHL globally.[1] However, whether the function is appropriate for estimating WHL at the provincial level of China is still unknown.

The percentage of workers in the primary, secondary and tertiary industries is only reported at the country level, hence this proportion is distributed evenly to all grid cells, which is not consistent with the actual situation.

The result was different with the global report by Watts as we take different data sources and methodologies. Firstly, gridded population data was from the hybrid gridded demographic data for the world, and the percentage of people working in each industry was from the Chinese Statistical Bureau. Secondly, based on the Chinese national economy classification standard, workers were divided into engaging in the primary, secondary and tertiary industries instead of agriculture, industry and service

sectors in Watts et al.[1] Thirdly, we assumed a labourer works 8 hours a day instead of 12 hours. The daily work time of 8 hours is more realistic in China as this is stipulated by the Chinese labour law. In the sensitivity analysis, the WHL increased by 53%-56% if we assumed a labourer works 12 hours a day as Watts et al.[1]

Future form of indicator

The loss function should be further tested whether it is suitable for the actual situation in China, and the percentage of workers in different industries at the provincial or city level is needed. Also, this indicator will be updated to show WHL in more specific sectors (e.g., manufacturing and construction) in the future. Finally, the percentage of workers in the primary, secondary and tertiary industries is only reported at the country level, hence this proportion is distributed evenly to all grid cells. In the future, the percentage at the provincial or city level is needed.

Additional Information

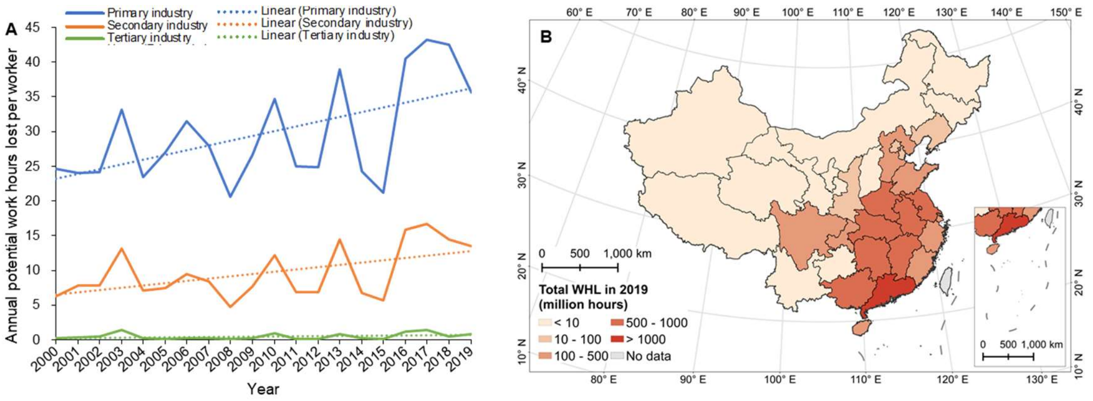

Figure 1: **Heat-related work hours lost in China. (A) Annual potential work hours lost due to heat per person employed in each industry from 2000 to 2019. (B) Total work hours lost in different provinces in 2019.**

Table 4: The total work hours lost (WHL, millions) and average WHL per person working in the primary industry, secondary industry and tertiary industry from 2000 to 2019 in China

Year	Primary industry		Secondary industry		Tertiary industry		All industry	
	Total WHL (millions)	WHL per person	Total WHL (millions)	WHL per person	Total WHL (millions)	WHL per person	Total WHL (millions)	WHL per person
2000	8481.2	24.6	969.1	6.3	33.1	0.2	9483.4	13.8
2001	8360.3	24	1211.2	7.8	60.4	0.3	9631.9	13.9
2002	8468.9	24.2	1163.8	7.8	102.8	0.5	9735.5	13.9
2003	11476.5	33.2	1998.4	13.1	298.1	1.4	13773	19.5
2004	7818	23.5	1140.1	7.1	44.1	0.2	9002.1	12.7
2005	8638.5	27	1267.5	7.5	38.4	0.2	9944.5	13.9
2006	9627.1	31.5	1709.4	9.5	64.6	0.3	11401.1	15.9
2007	8227.3	28	1617.7	8.4	53.4	0.2	9898.4	13.7
2008	5920.5	20.7	936.7	4.8	16.1	0.1	6873.2	9.5
2009	7374.8	26.6	1547.1	7.7	43.3	0.2	8965.2	12.3
2010	9292.1	34.7	2553.2	12.2	230	0.9	12075.3	16.5
2011	6374.7	25	1489.6	6.9	39.6	0.2	7903.8	10.8
2012	6149.4	24.8	1528.9	6.9	19.1	0.1	7697.4	10.5

2013	9040	38.9	3209	14.4	238.5	0.8	12487.4	16.9
2014	5312	24.3	1506.1	6.8	66.4	0.2	6884.5	9.3
2015	4459	21.2	1228.9	5.6	18.4	0.1	5706.3	7.7
2016	8356.7	40.5	3397.8	15.8	368.3	1.1	12122.8	16.3
2017	8663	43.2	3486.6	16.7	473.9	1.4	12623.4	17
2018	8239.5	42.5	2962.3	14.5	156.5	0.5	11358.3	15.3
2019	6905	35.7	2757.9	13.5	275.3	0.8	9938.2	13.4

The reasons why results for indicator 1.1.2 and 1.1.3 have different spatial patterns are at least two-fold:

First, the working hour loss(WHL) is affected by two factor, heat and working-age population, while the vulnerable population of heatwave-related mortality are mainly people older than 65 years. So, the different spatial patterns of working-age people and old people caused the different distribution of figure 3 and 4. For example, Guangdong province is the most concentrated province of manufacturing industry, so there are much more people aged 14-65 than 65+. That's why Guangdong's WHL is high but the heatwave-related mortality is relatively low.

Second, although the temperature is the same, the heat effects are different for WHL and heatwave-related mortality. WHL is calculated using WGBT, while heat-related mortality is calculated using daily maximum temperature. The two are different because WGBT related to air humidity, air movement, radiation temperature and air temperature.

Indicator 1.1.4: Health and exposure to warming

Methods

This indicator remains similar to the methodology described in the 2019 and 2020 global Lancet Countdown reports, with a focus on China. Monthly averaged summer temperature (June, July and August) was obtained from the ERA5 reanalysis data set and population count data from a hybrid gridded demographic data. Both are gridded data with horizontal grid of 0.5°. Population-weighted temperature and area-weighted temperature were calculated every year from 1986 to 2019 for every province and the entire country. Changes in population-weighted and area-weighted temperatures were calculated every year 2000 to 2019 with 1986-2005 as the baseline. Area-weighted temperature was calculated by averaging temperature records at every grid inside a province/for the entire country. Population-weighted temperature was calculated in a similar method with weights proportional to population count.

Data

1. Climate data was taken from European Centre for Medium-Range Weather Forecasts (ECMWF), ERA5 project.[2]

2. Population data is from a hybrid gridded demographic data for the world, created by Chambers (2020).[3]

Caveats

The horizontal resolution of temperature data is too coarse to reflect warming trend at local level. Localized temperature data are preferred.

Future Form of Indicator

Future version may consider using localized reanalysis data set, instead of the global reanalysis data set.

Findings

The country-wide population-weighted temperature rose by 0.64°C in 2019 compared with the 1986-2005 baseline. Province-level changes in annual average population-weighted temperature from 2000 to 2019 are presented **(Figure 2, Figure 3),** relative to the 1986-2005 average. Regions with profound warming are in Southwest China, such as Qinghai-Tibet Plateau and Sichuan Province, where ecosystems are fragile, and Yangtze Delta region, the most economically advanced and populous region in China. Subtropical provinces in South Central China, such as Hainan, Guangdong and Guangxi, witness less population-weighted temperature rise.

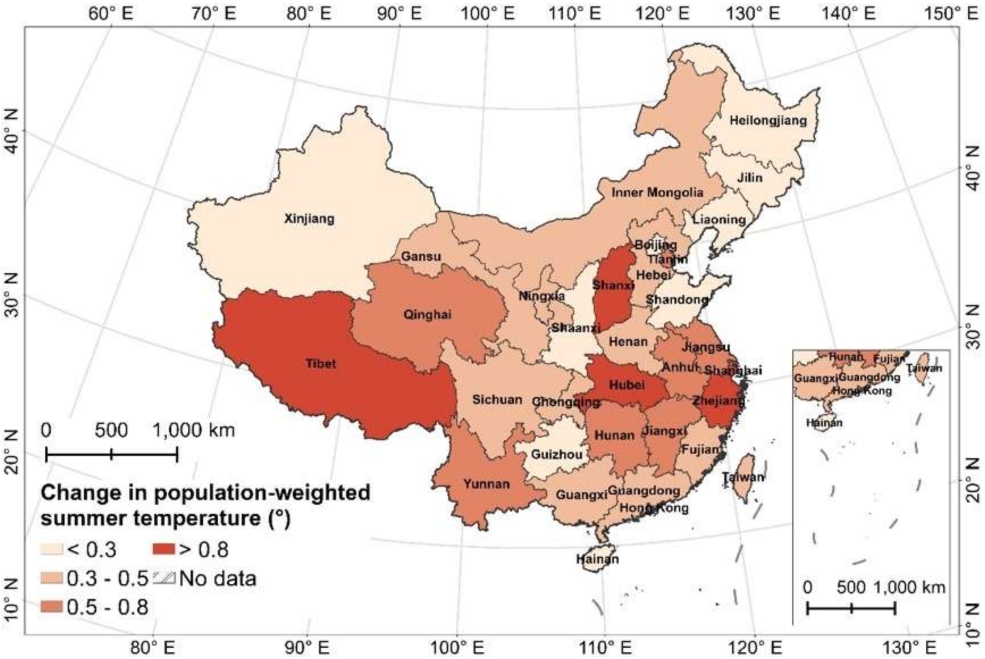

Figure 2: Change in population-weighted summer temperature in 2019, relative to the 1986-2005 average

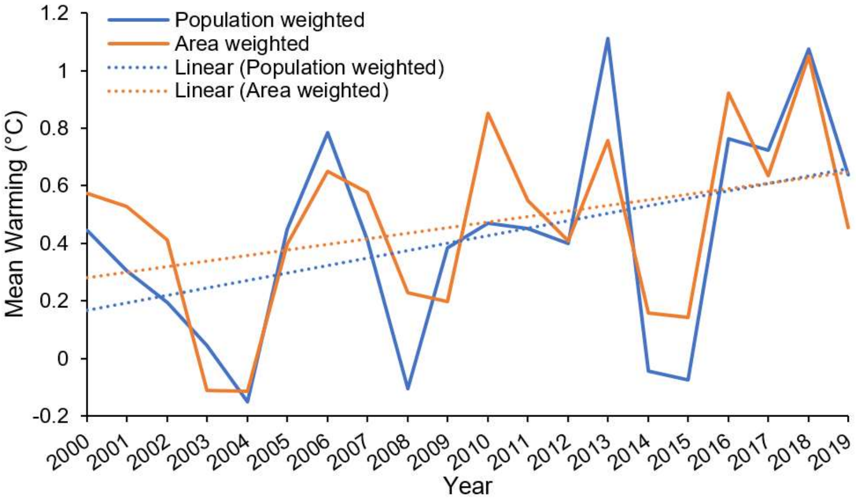

Figure 3: Mean summer warming relative to the 1986–2005 average in China

Indicator 1.1.5: Vulnerability to extremes of heat

Methods

This indicator displays a heat vulnerability index derived from (1) the proportion of the population over 65 years, (2) the prevalence of chronic disease among population over 65 years, (3) the proportion of the population living in urban areas, (4) the number of air conditioners owned per 100 urban households at year-end and (5) the green covered area as % of built-up area, using the equation below:

$$HV_i = \frac{(pop65_i + popurban_i + disease_i - AC_i - green_i)}{5}$$

in which, HV_i refers to the heat vulnerability index in province *i;* $pop65_i$ refers to the proportion of population over 65 years in province *i;* $popurban_i$ refers to the proportion of the population living in urban areas in province *i*; $disease_i$ is the chronic disease prevalence among population over 65 years in province *i*. AC_i is the air conditioner ownership per 100 urban household in province *i*, $green_i$ is the percentage of green covered area in province *i*. Increased urbanization may exacerbate heat island effect and therefore the health effect of heat [14], while expanding green area and installing air conditioner are treated as adaptation measures of this [15,16].

Reasons why five factors are considered in the heat vulnerability index are as follows:

The equation to calculate the heat vulnerability index is referred to sub-indicator 1.1.1 Vulnerability to extremes of heat in the 2019 Lancet Countdown Report [1], indicator 1.1 vulnerability to the heat-related risks of climate change in the 2018 Lancet Countdown report [17] and indicator 1.1 Vulnerability to the heat-related risks of climate change in the 2019 MJA-Lancet Countdown Report [18]. In these three reports, the heat vulnerability index is calculated with the same indicators which include proportion of the population over 65 years, the prevalence of cardiovascular, diabetes and chronic respiratory diseases among population over 65 years and the proportion of the population living in urban (i.e. urbanization rate). As concluded from previously studies, populations aged over 65 years, especially those with underlying disease are more vulnerable to health effect of heat than others [1]. And increased urbanization may exacerbate heat island effect and therefore the health effect of heat [14]. Therefore, these three indicators are taken into account to be a measure of possibly increased heat vulnerability. However, some other studies also show that expanding green area and installing air conditioner are treated as adaptation measures from heat [15 16], which are proved to decrease the heat vulnerability to some extent. In addition, Watts N, et al also mentioned that heat vulnerability in their report did not include prevalence of cooling devices and the prevalence of green areas in cities, which are the caveats of their studies. To make some improvement of this indicator, the number of air conditioners owned per 100 urban households at year-end and the green covered area as % of built-up area are included in the China Lancet Cutdown Report. According to the opposite effect of these indicators on the heat vulnerability, the equation from the global report was revised, using addition when the indicator may increase the vulnerability and subtraction when it may decrease the vulnerability. Although there are still lots of influencing factors of heat vulnerability, for now we only focus on these five most important and relatively convinced factors due to the limited evidence and data availability.

Equal weights are assigned to each indicator without consideration of the relative importance of them. Some researchers applied weights to the indicators based on the strength of statistical relationship with health outcome [19]. However, the indicators used may not be independent of each other due to the complexity of the vulnerability, which may lead to the unbalanced emphasis of the factors when using unequal weights [20]. What's more, the weights of the factors may largely depend on the context, thus the weights may change with the different part of China. Therefore, equal weights are assigned for each indicator in this study to avoid the possible bias from the inadequate understanding of the heat vulnerability.

The number of air conditioners owned per 100 urban households at year-end was normalized to the range from 0 to 1 using Max-Min Method to keep consistent with other sub-indicators before calculation. Then the index was normalized again to provide ranges between 0 and 100. The higher value of the index, the higher the vulnerability to heat exposure is. Due to data limits, the number of air conditioners owned per 100 urban households at year-end was missing for Tianjin, Jilin, Shanghai, Hunan, Yunnan, Tibet, Gansu and Xinjiang in 2013 and 2014, and green covered area as % of built-up area was missing for Beijing in 2010, Tianjin in 2007 and 2008, and Shanghai in 2008 and 2009. To account for the missing values, a linear regression model was used to make the estimations, as the sub-indicator shows a good linear relationship with year. In

addition, the prevalence of chronic disease among the population over 65 years were available for 1998, 2003, 2008 and 2013 at the national level. A linear regression model was used again to estimate the prevalence for other years during 2000-2013. However, the prevalence after 2013 was assumed to be unchanged to avoid further uncertainty. The index displays aggregated trends by regions for the period 2000 to 2018. The Cox-Stuart trend test was used to examine the significance of trend with years.

Data

1. The prevalence of chronic disease among population over 65 years was extracted from National Health Service Survey (NHSS) Report published by Statistical Information Center of National Health Commission. (http://www.nhc.gov.cn/mohwsbwstjxxzx/).

2. The other data related to the heat vulnerability index (proportion of the population over 65 years, proportion of the population living in urban areas, air conditioner owned per 100 urban households at year-end, green covered area as % of built-up area (%)) was extracted from China Statistical Yearbook compiled by National Bureau of Statistics from 2001 to 2019.[13]

Caveats

The caveats of this indicator would mainly be in four aspects.

First, the prevalence of chronic disease among population over 65 years is not available at the provincial level. Second, the index does not include the existence of heat early warning systems. Third, a linear regression model was used to handle with the missing value in some sub-indicators, leading to some bias. Fourth, adjustment of survey method used by National Bureau of Statistics during the study period may cause fluctuation of the data to some extent.

Future Form of Indicator

In the future, we would consider including more sub-indicators to better reflect various aspects of vulnerability to heat.

Findings

Despite the increasing heat and heatwave exposure per capita, the national average vulnerability has increased by 0.92% since 2000. Vulnerability continues to rise in every region of China (**Figure 4**). Northeast China remains the most vulnerable area, followed by Northwest China, Southwest China and North China**.** If adaptation measures such as green area and air conditioner were excluded from the index, the vulnerability to extremes of heat in China would increase by 59% from 2000 to 2018.

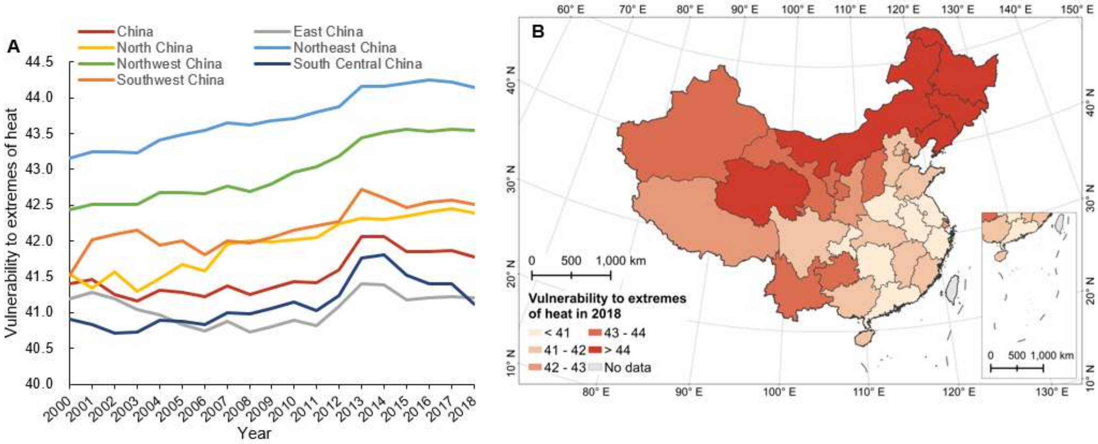

Figure 4: Vulnerability to extremes of heat in China. (A) Trend in different regions from 2000 to 2018. (B) Distribution of vulnerability index in 2018

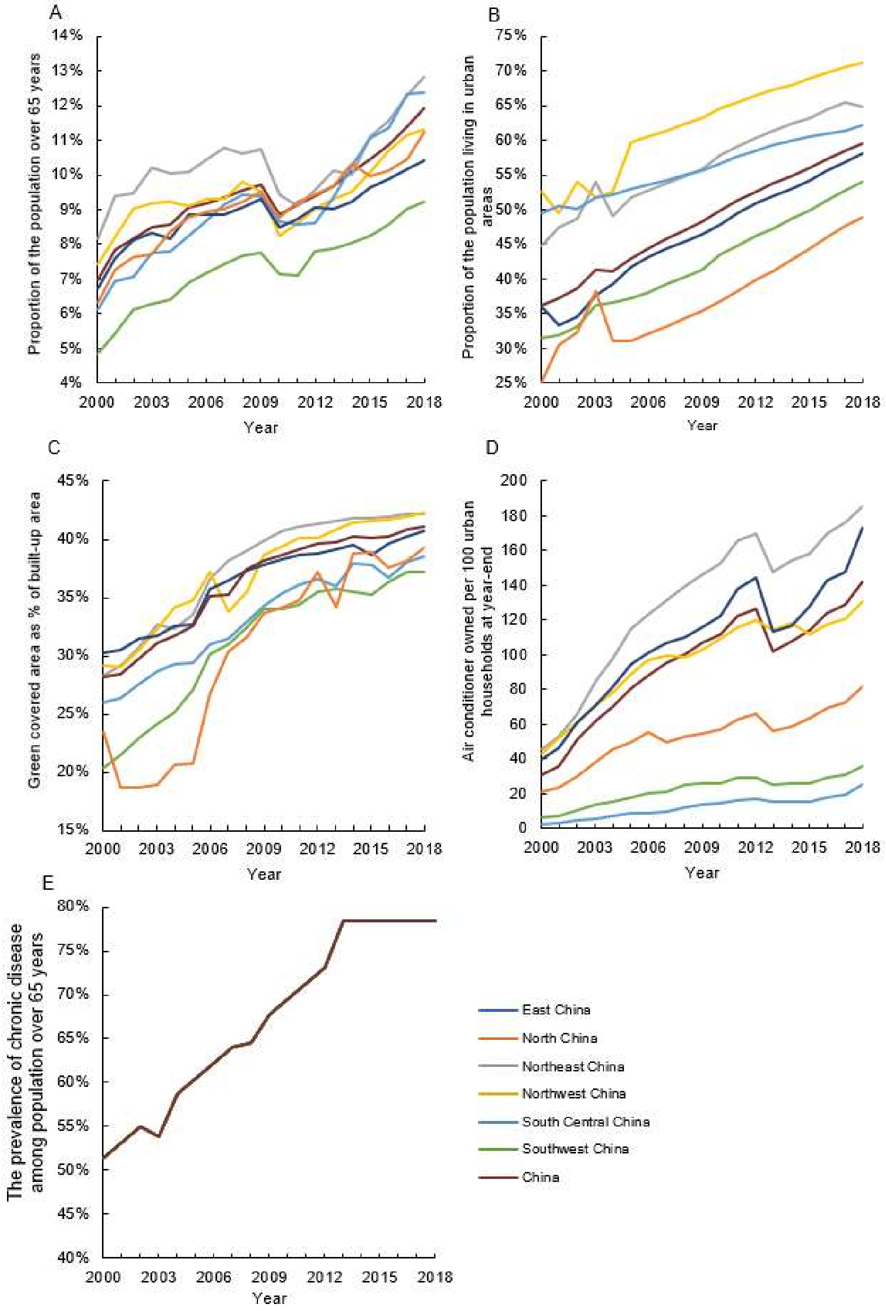

Figure 5: Trend of (A) Proportion of the population over 65 years, (B) Proportion of the population living in urban areas, (C) Green covered area as % of built-up area (%), (D) Air conditioner owned per 100 urban households at year-end in China from 2000 to 2018, (E) The prevalence of chronic disease among population over 65 years.

The sudden decline in proportion of population over 65 years around 2011, air conditioner ownership in China around 2013 was due to the change in statistical approach.

Indicator 1.2: Health and extreme weather events

Indicator 1.2.1: Wildfires

Methods

The methodology for this indicator remains the same as the indicator in the 2020 global Lancet Countdown report. It was calculated by the following equation:

$$PD_y = Pop_y \times \sum FP_{d,pixel}$$

Where $\boldsymbol{PD_y}$ refers to person-days exposed to wildfire in a specified year y, and $\boldsymbol{Pop_y}$ refers to the population count from gridded population data in a specified year y. $\boldsymbol{FP_{d,pixel}}$ refers to a fire point count located within a population data pixel in an unique day $\boldsymbol{d}$ of year $\boldsymbol{y}$. The Collection 6 active fire product[21] is acquired by the Moderate Resolution Imaging Spectroradiometer aboard the NASA Terra and Aqua satellites. Unique acquired date counts of all fire points within a population pixel were calculated, and multiplied by population count per square kilometer, taken from NASA SEDAC GPWv4.35,[21] with urban areas (population density ≥ 400 persons/km^2) removed. Annual exposure days are grouped by into four periods: 2001-2005, 2006-2010, 2011-2015 and 2016-2019. Then mean value of each period was calculated. Finally, the exposure days were allocated to provinces (***Figure** 7* and ***Table** 5*) and 6 regions of China (***Figure** 8*) using zonal statistical methods.

Data

1. Fire point data was downloaded from NASA Near Real-Time and MCD14DL MODIS Active Fire Detections (SHP format).[22]
2. Population data was taken from the Chambers (2020) hybrid gridded demographic data for the world[23] and the Gridded Population of the World Version 4 (GPWv4).[22]

Caveats

The information on confidence field and pixel distance in MODIS database haven't been used in this year's analysis to identify the number of people affected.

This indicator doesn't explicitly describe how the smoke from wildfire would influence human health.[24] The exposure level in wildland-urban interface is underestimated due to the removal of urban area based on the population density.

Future Form of Indicator

In the future, VIIRS data (available since 2012) can be merged to MODIS fire points to calculate the annual indicator result, rather than simply validate the result. The information on fire point confidence and pixel coverage from MODIS database can be used to identify the number of people affected.

Further work will also be undertaken to explore how exposure to wildfire smoke can be captured in this indicator.

Additional Information

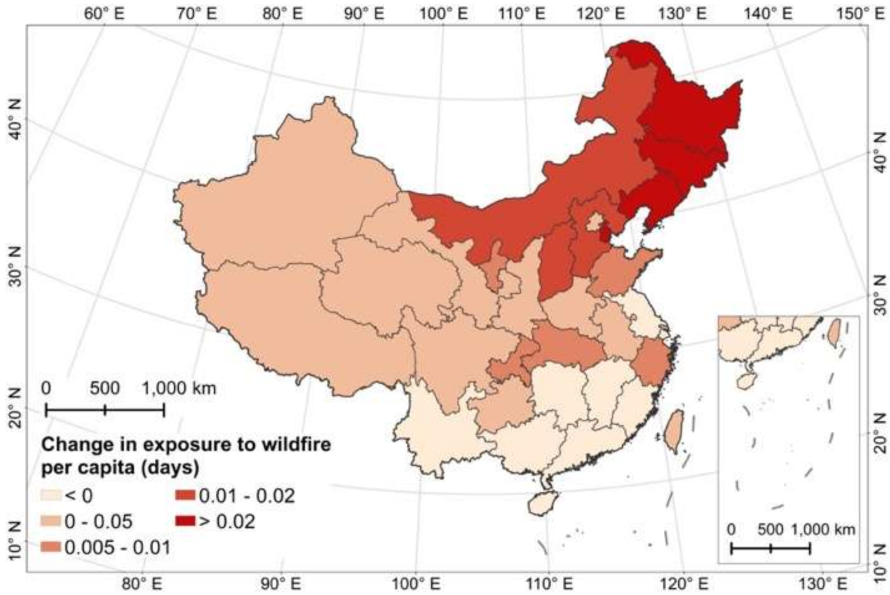

***Figure 6*: Annual average change in exposure to wildfire per capita in different provinces of China during 2016-2019, compared to 2001-2005**

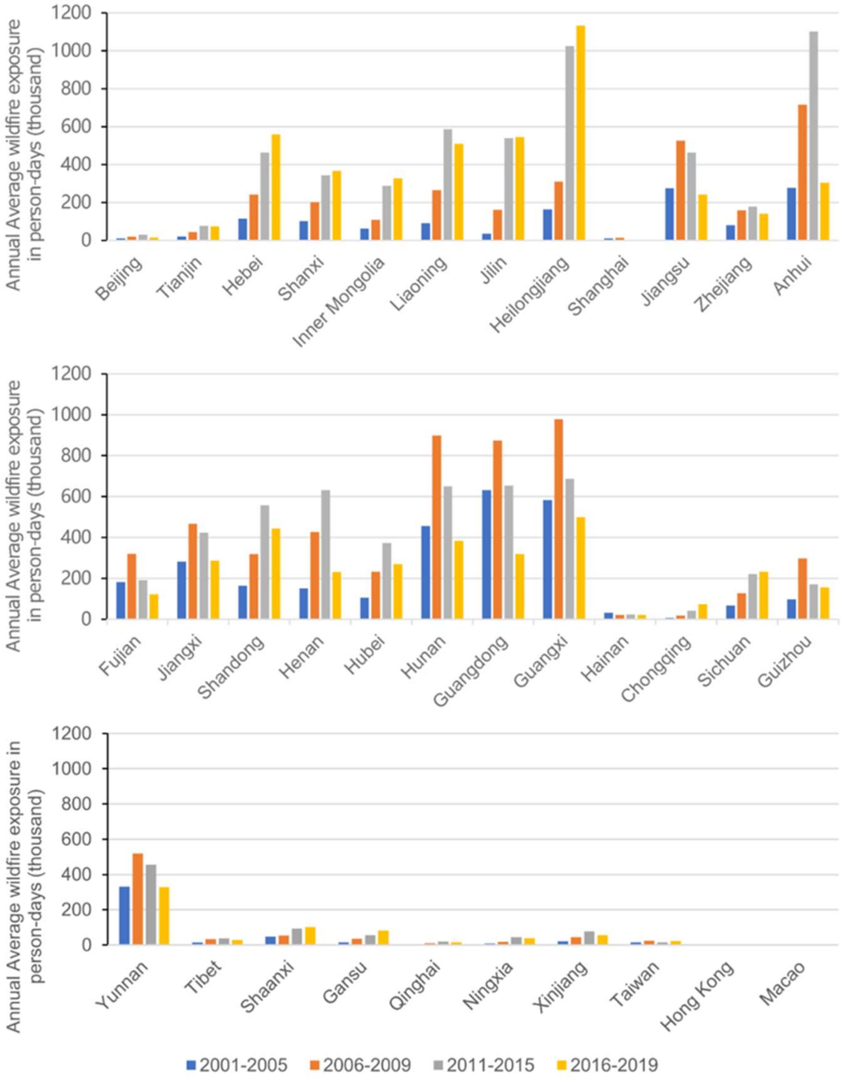

Figure 7: Annual Average Person-days Exposed to Wildfire in Provinces of China, from 2001 to 2019

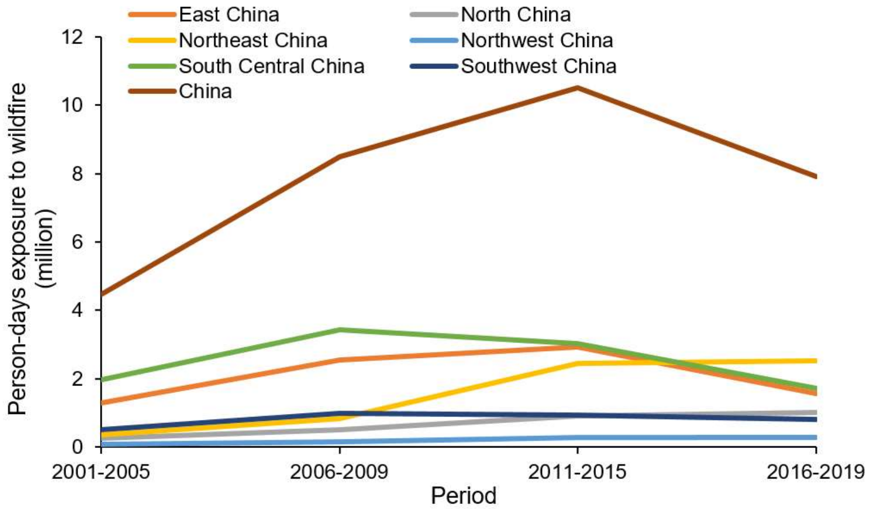

Figure 8: Annual Average Person-days Exposed to Wildfire in six regions of China, from 2001 to 2019

Table 5: Annual Average Person-days Exposed to Wildfires in Provinces of China, from 2001 to 2019

Province	2001-2005	2006-2010	2011-2015	2016-2019
Beijing	9,527	20,460	29,892	15,056
Tianjin	21,134	44,468	76,944	73,957
Hebei	115,508	240,993	463,425	558,113
Shanxi	102,214	201,257	343,560	366,931
Inner Mongolia	62,258	108,625	288,942	326,831
Liaoning	90,067	265,632	586,606	509,408
Jilin	35,975	161,840	539,265	546,148
Heilongjiang	164,006	309,878	1,025,086	1,132,985
Shanghai	9,785	14,098	2,153	620
Jiangsu	275,753	525,651	463,493	240,491
Zhejiang	79,967	158,207	177,609	140,601
Anhui	276,908	716,847	1,101,342	303,894
Fujian	182,076	320,030	190,356	122,355
Jiangxi	282,228	466,663	424,280	287,285
Shandong	164,443	319,377	557,111	444,405
Henan	151,468	426,745	631,763	231,280
Hubei	106,333	232,618	372,435	268,833
Hunan	455,882	898,682	650,163	383,556
Guangdong	631,397	873,869	653,493	318,867
Guangxi	582,128	977,535	687,837	499,334
Hainan	32,626	21,198	24,164	21,681
Chongqing	7,157	18,591	42,229	73,652
Sichuan	67,653	127,245	221,497	232,508
Guizhou	98,069	298,292	171,208	155,944
Yunnan	331,808	518,658	455,622	328,971
Tibet	14,145	32,650	37,689	27,778
Shannxi	47,629	54,016	93,267	100,947
Gansu	15,558	35,436	55,198	82,155
Qinghai	957	9,432	18,803	14,552
Ningxia	7,599	18,142	44,339	38,598
Xinjiang	21,103	44,423	76,160	55,776
Taiwan	15,870	22,881	15,635	22,128
Hong Kong	698	438	407	211
Macao	NaN	NaN	NaN	NaN

Note: NaN means not available due to the area of Macau being too small.

Indicator 1.2.2: Cyclones

Methods

In this indicator, data on exposure and damage levels from cyclones were taken three databases were used to estimate the effects of tropical cyclones in China. Exposure data from 1980 to 2019 are from the China Meteorological Administration (CMA) Tropical Cyclone Database provided by CMA Tropical Cyclone Data Centre.[25] Damage data in mainland China is taken from China Meteorological Disaster Yearbook, which has been published since 2004 by China Meteorological Press. The damage related information in Taiwan Province was downloaded from the Statistical Bureau of Taiwan. The time scale of damage data is from 2004 to 2017.

Based on maximum average wind speeds near the bottom center of tropical cyclones given by the CMA, six different grades could be defined: (1) tropical depression (10.8-17.1m/s); (2) tropical storm (17.2-24.4m/s); (3) severe tropical storm (24.5-32.6m/s);(4) typhoon (32.7-41.4m/s); (5) severe typhoon (41.5-50.9m/s); (6) super typhoon (≥51m/s).

On the exposure dimension, the tropical cyclones are described in frequency, intensity and spatial-temporal distributions at both the country and provincial levels. The student t-test is used to compare the difference between the number of tropical cyclones from 2000 to 2019 and the baseline of the reference period (1980-1999) when the data satisfy the normality test. Otherwise, the Mann-Whitney U test is used. Based on these methods, the cluster of typical vulnerable areas could be found. The intensity changes could also be identified

by comparing the relationships among the tropical cyclone levels on temporal scales.

Based on the hot-spot areas on exposure dimension, the spatial-temporal distribution of population affected and relocated as well as houses and crop fields destroyed are used to evaluate the damages of these disasters on the eco-system. The Mann-Kendall test, a non-parametric test, is used to explore the tendencies of these aspects of damage dimension.[26]

Data

1. Exposure data from 1980 to 2019 are from the CMA Tropical Cyclone Database provided by CMA Tropical Cyclone Data Centre. (http://tcdata.typhoon.org.cn) [25,27]
2. Damage data in mainland China origin from China Meteorological Disaster yearbook published by China Meteorological Press.[27]
3. The damage data in Taiwan Province was downloaded from the Statistical Bureau of Taiwan. [28]

Caveats

The caveats of this indicator would mainly be in three aspects. First, the health effects caused by tropical cyclones on the vulnerable population are not evaluated. The data is provincial-based, so the change of vulnerability cannot be reflected, especially in typical cities that are most affected by tropical cyclones. The changing of adaptation capability at the provincial levels is not evaluated.

Future form of indicator

1. More information on the relationships between tropical cyclones and health outcomes in China will be studied.
2. Data at the typical cities would be used to identify the health vulnerability after the tropical cyclone exposure.

Additional information

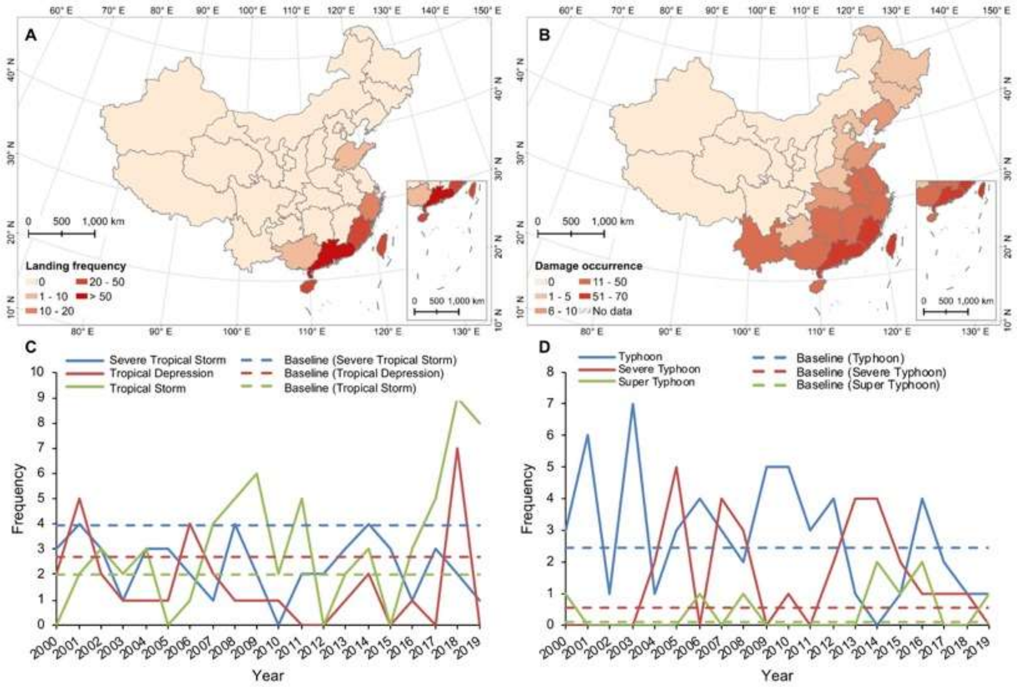

Figure 9: The spatial-temporal distribution of tropical cyclone and its damages nationwide (A) The spatial pattern of cumulative landing locations of tropical cyclones from 2000-2019. (B) Spatial pattern of cumulative damage occurrences of tropical cyclone from 2004-2017, China. (C) The temporal trends of occurrence of tropical cyclones below the typhoon grade. (D) The temporal trends of occurrence of tropical cyclones typhoon grade and above .

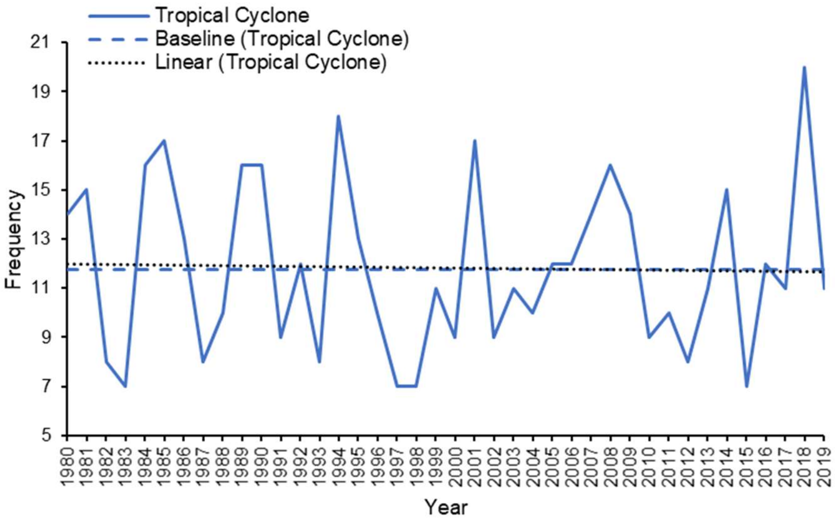

Figure 10: **Frequency of Landing on Tropical Cyclones from 1980 to 2019. There is no statistical difference in distribution between the study period and the reference period of tropical cyclones.**

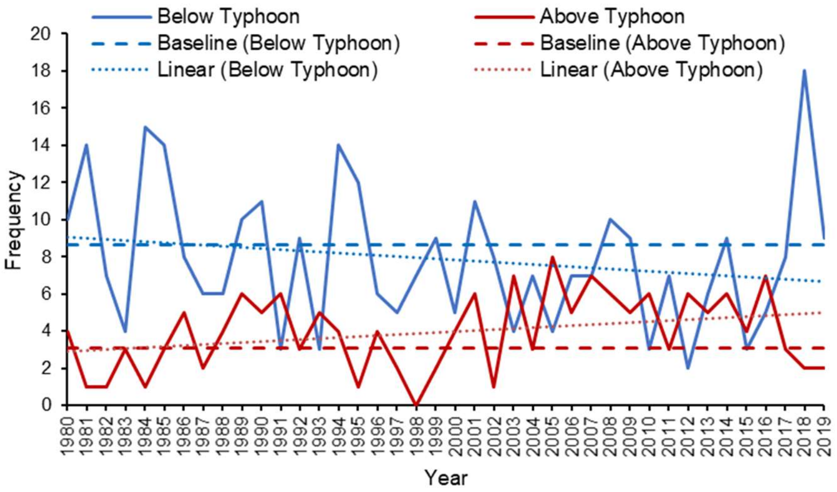

Figure 11: **Frequency of Tropical Cyclones Landfalling on Different Levels from 1980 to 2019.**
The dashed lines are the mean of landfalling frequency at different levels in the reference period. Compared with the reference period, the statistical difference was found at above typhoon level, because of the increasing occurrences of severe and super typhoons.

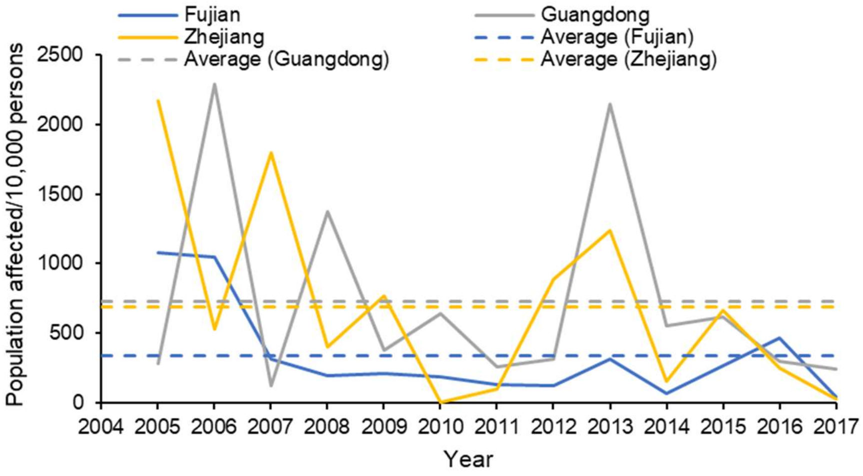

Figure 12: **Population affected by Tropical Cyclones 2004-2017 in typical provinces that are most affected by tropical cyclones. The dashed lines are the mean in the study period. The population affected in Fujian Province are declining significantly.**

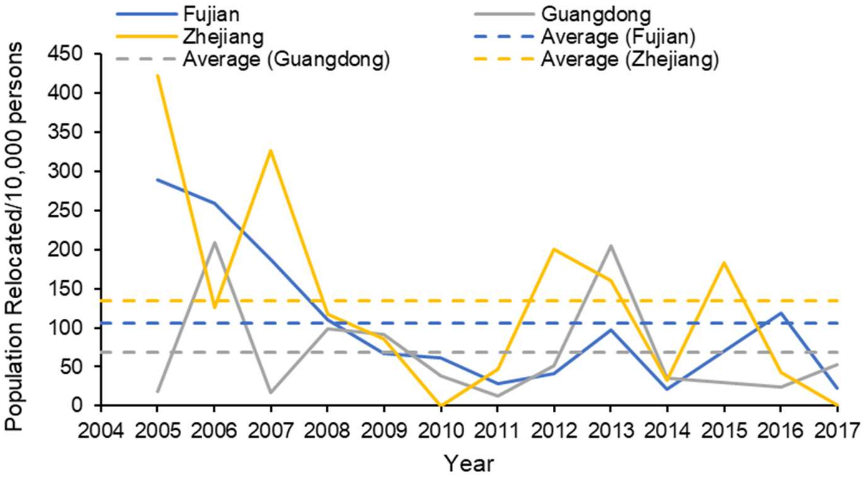

Figure 13: Population Relocated due to Tropical Cyclones in typical provinces that are most affected by tropical cyclones. The dashed lines are the mean in the study period. The relocated population in Zhejiang Province had declined significantly.

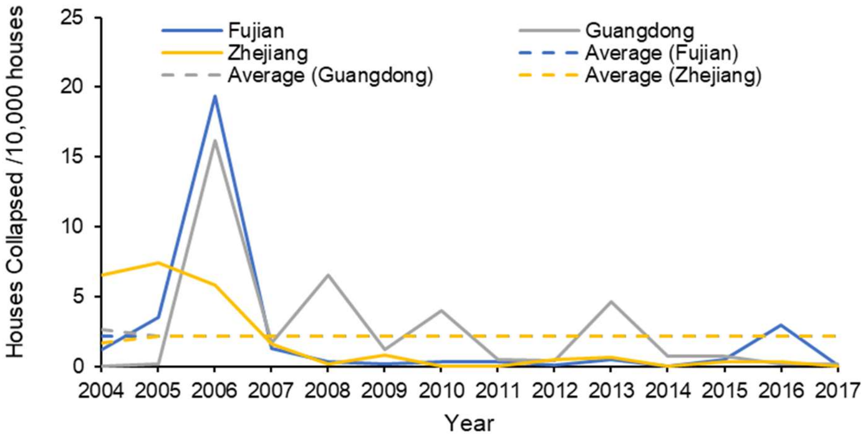

Figure 14: Houses Collapsed caused by Tropical Cyclones on typical provinces that are most affected by tropical cyclones. The dashed lines are the mean in the study period. The houses collapsed in Zhejiang Province had declined significantly.

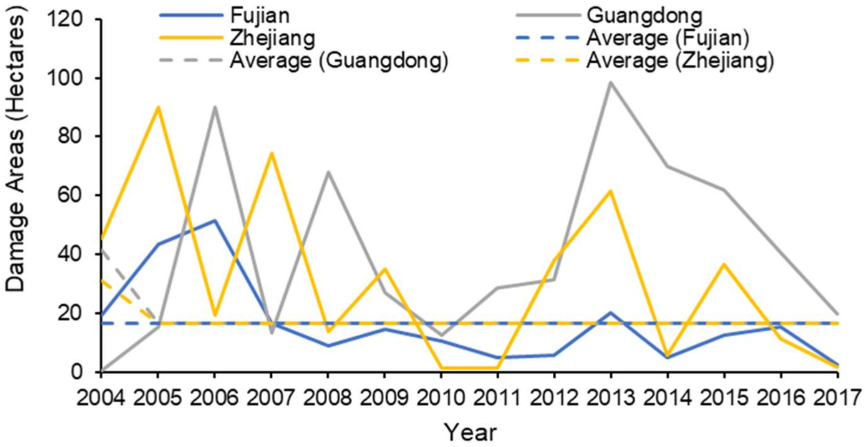

Figure 15: Crop Fields Destroyed caused by Tropical Cyclones on typical provinces that are most affected by tropical cyclones. The dashed lines are the mean in the study period. The crop fields destroyed in Fujian Province had declined significantly.

Table 6: Frequency of Different Grades of Tropical Cyclones on Landing from 1980 to 2019

Year	Tropical Depression	Tropical Storm	Severe Tropical Storm	Typhoon	Severe Typhoon	Super Typhoon
1980	2	5	3	2	2	0
1981	6	3	5	0	1	0
1982	1	3	3	0	1	0
1983	2	0	2	3	0	0
1984	4	1	10	1	0	0
1985	6	1	7	2	1	0
1986	4	3	1	4	1	0
1987	2	0	4	2	0	0
1988	4	0	2	4	0	0
1989	3	2	5	5	0	1
1990	2	4	5	4	1	0
1991	1	0	2	4	2	0
1992	1	3	5	3	0	0
1993	1	1	1	5	0	0
1994	3	4	7	2	2	0
1995	4	3	5	1	0	0
1996	0	3	3	3	1	0
1997	1	1	3	2	0	0
1998	2	2	3	0	0	0
1999	5	1	3	2	0	0
2000	2	0	3	3	0	1
2001	5	2	4	6	0	0
2002	2	3	3	1	0	0
2003	1	2	1	7	0	0
2004	1	3	3	1	2	0

2005	1	0	3	3	5	0
2006	4	1	2	4	0	1
2007	2	4	1	3	4	0
2008	1	5	4	2	3	1
2009	1	6	2	5	0	0
2010	1	2	0	5	1	0
2011	0	5	2	3	0	0
2012	0	0	2	4	2	0
2013	1	2	3	1	4	0
2014	2	3	4	0	4	2
2015	0	0	3	1	2	1
2016	1	3	1	4	1	2
2017	0	5	3	2	1	0
2018	7	9	2	1	1	0
2019	0	8	1	1	0	1

Table 7: Total Frequency of Different Grades of Tropical Cyclones on Landing at Provincial Levels from 1980 to 2019

Landing Provinces	Tropical Depression	Tropical Storm	Severe Tropical Storm	Typhoo n	Severe Typhoon	Super Typhoon
Fujian	7	19	21	21	3	1
Guangdong	30	31	44	32	8	2
Guangxi	6	10	5	0	1	0
Hainan	24	20	17	19	3	1
Jiangsu	1	0	2	1	0	0
Liaoning	3	2	1	0	0	0
Shandong	5	5	2	0	0	0
Shanghai	0	2	2	0	0	0
Taiwan	7	7	19	23	22	4
Hong Kong	2	3	3	0	0	0
Zhejiang	1	4	10	10	5	2

Table 8: Damages Caused by Tropical Cyclones

Year	Population Affected/1,000	Population Relocated/1,000	Houses Collapsed/1,000	Damage Area/Hectares
2004	NA	NA	89.4	1019.22
2005	73679	9399	342	4663
2006	72251.4	8940.1	723	2957.9
2007	42260.6	7273.6	84	2082.03
2008	37915.6	4922.3	127.6	2310.3
2009	19435.6	2784.6	25.3	1114.3
2010	11491.6	1227	49.2	342
2011	18128	2715	25	1548
2012	47637	5696	130	3491
2013	49222	5552	91	2672
2014	26595	1773	52	2483
2015	23756	3595	23	1721
2016	17212	2606	38.2	2024

2017	5859	1091	3.9	394

Table 9: Damages Caused by Tropical Cyclones at Provincial Levels from 2004 to 2017

Affected Province	Population Affected/1,000	Damage Area/Hectares	Population Relocated/1,000	Houses Collapsed/1,000
Anhui	18562.9	133.384	1284.9	132.6
Fujian	44404.3	231.45	13719.3	304.9
Guangdong	95122.7	577.51	8847.5	370.3
Guangxi	53511.9	394.19	3848.9	148.6
Guizhou	235	1.1	15	0
Hainan	38883.1	230.163	3915.8	67
Hebei	5698	8.5	359	24.1
Heilongjiang	3708	136.6	5	1
Henan	2022	19.34	1	12.5
Hubei	4431.2	36.62	180.7	42.1
Hunan	20590.6	98.629	2373.3	223.4
Jiangsu	19162.1	214.813	865.9	23.5
Jiangxi	17417.7	93.723	1271.2	98.8
Jilin	3697	27.3	59	2
Liaoning	6666	62.3	745	31
Shandong	12770.5	121.61	1037	50
Shanghai	2655.1	18.413	1499.7	16
Tianjin	322	6.7	0	2
Yunnan	5650	34.51	79.5	15.1
Zhejiang	89932.7	435.32	17466.9	238.7

Indicator 1.2.3: Flood and drought

Methods

China suffers from frequent floods. More than half of the deaths related to natural disasters were caused by flood in the past two decades.[22] Apart from direct death or physical and mental harms, it also destroys livelihood and shelters disease vectors that threaten human health and well-being.[29] Prolonged drought may impact human health by shortening water supply and farmland production.[30]

In this context, flood is defined as hydrological flood, including riverine flood (the overflow of water from a stream channel onto normally dry land in the floodplain), coastal flood (higher-than-normal levels along the coast and in lakes or reservoirs) and flash flood (pooling of water at or near the point where the rain fell). Drought refers to climatological drought, which is an extended period of unusually low precipitation that produces a shortage of water for people, animals and plants. For a disaster to be entered into this report at least one of the following criteria must be fulfilled: (1) 10 or more people reported killed; (2) 100 or more people reported affected; (3) declaration of a state of emergency; and (4) call for international assistance.[31]

All flood and drought disasters in China (including Taiwan, Hong Kong and Macao) for years 1980-2019 were extracted from the EM-DAT international disaster database, and the number of disasters in China and each province per year was counted according to the location recorded in EM-DAT. The Student's t-test is used to compare the difference between the number of floods and droughts from 2000 to 2019 and the baseline of reference period (1980-1999) when the data meet the normal test. Otherwise, the Mann-Whitney

U test is used. Furthermore, flood is classified into three levels including moderate and less flood, severe flood, and major flood according to the number of deaths and overall economic losses (Table 10).[32]

Table 10: Criteria of catastrophe classes for flood

Flood classes	Overall losses (in million US dollars)				Fatalities
	1980-1989	1990-1999	2000-2009	2010-2019	
Moderate or less	0.63≤US$<29	0.91≤US$<42	1.18≤US$<54	1.33≤US$<61	1-19
Severe	29≤US$<114	42≤US$<164	54≤US$<212	61≤US$<239	20-99
Major	US$≥114	US$≥164	US$≥212	US$≥239	≥100

Note: If only one criterion is met (i.e. either fatalities or overall losses), this is sufficient for classification

The cumulative number of floods and droughts in each province during 2000-2019 was counted and the vulnerable regions are identified.

Data

1. EM-DAT at the Centre for Research on the Epidemiology of Disasters (CRED) at the Université Catholique de Louvain, Belgium[31]

Caveats

It is difficult to estimate the number of people exposed to floods and droughts because the locations of disasters in EM-DAT database cannot always be accurate to city level. Additionally, since there is no direct index of disaster intensity, the death number and total economic loss of each catastrophe are used to classify the disasters, which may lack consistency in different databases and studies.

Data here is taken from the global EM-DAT database. The reasons for not choosing Meteorological Disaster Yearbooks in China as the data sources are three fold: first, the data from the yearbook only covers 2004-2017; second, they have three-year's lag in reporting the data. So the current latest data in the yearbook is in 2017; third, most of the disasters don't have data for occurrences. EM-DAT data is also used because it has detailed data on the occurrences, the province where the disaster occured, the number of people affected and the number of deaths. It is also easier to make comparisons with the global report.

Future Form of Indicator

The EM-DAT database is compiled from various sources including United Nations, governmental and non-governmental agencies, insurance companies, research institutes and press agencies and updated on a daily basis to ensure its credibility and completeness. It will continue to be the main data source for tracking this indicator. However, efforts should be made in the future to obtain the number of people exposed to each disaster through cooperation with Chinese institutions.

Findings

Compared with the reference period (1980-1999), the number of flood disasters, and especially major floods, has increased significantly from 2000 to 2019. It is noted that the number of flood disasters increased significantly from 2013 to 2017, which is probably due to the 2014-2016 strong El Niño.[33] In 2019, there were six floods classified as disasters in China. Most floods occurred in provinces in Southwest and South Central China such as Sichuan, Guizhou, Hunan and Hubei provinces, and their flood occurrences are all significantly higher than that of the reference period.

Compared with 1980-1999, there was no significant change in the number of drought in 2000-2019, with no severe drought between 2018 and 2019. Northern China is drought-prone, with Inner Mongolia suffering the most.

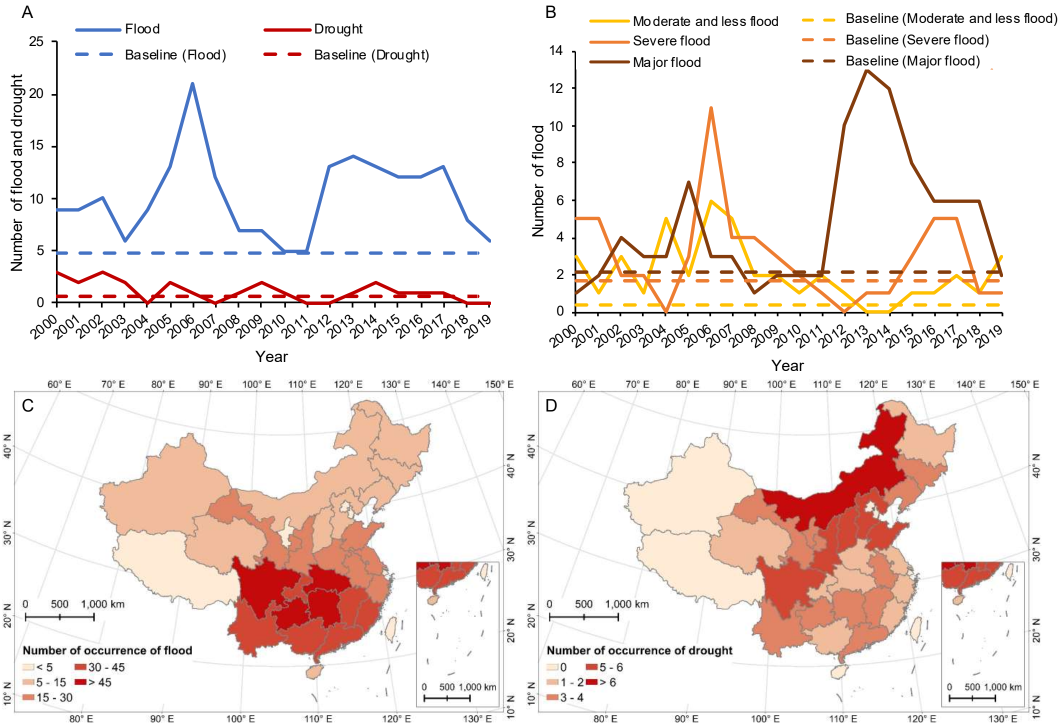

Figure 16: Temporal and spatial distribution of flood and drought: 2000-2019. (A) Changes in the occurrences of flood and drought (horizontal dashed lines represent the baseline levels of reference period. (B) Number of flood with different intensity levels. (C) Cumulative number of flood from 2000 to 2019 by province. (D) Cumulative number of drought from 2000 to 2019 by province.

Table 11: Annual occurrences of flood and drought between 1980-1999 and 2000-2019

Year	Number of flood	Number of drought	Year	Number of flood	Number of drought
1980	4	0	2000	9	3
1981	5	1	2001	9	2
1982	5	0	2002	10	3
1983	2	2	2003	6	2
1984	3	0	2004	9	0
1985	9	1	2005	13	2
1986	2	0	2006	21	1
1987	6	0	2007	12	0
1988	8	1	2008	7	1
1989	2	0	2009	7	2
1990	3	0	2010	5	1
1991	3	1	2011	5	0
1992	7	2	2012	13	0
1993	4	0	2013	14	1
1994	7	2	2014	13	2
1995	3	1	2015	12	1
1996	4	0	2016	12	1
1997	8	1	2017	13	1
1998	6	0	2018	8	0

1999	6	1	2019	6	0
Total	97	13	Total	204	23

Table 12: Cumulative number of floods and droughts in each province from 2000 to 2019

Province	Number of flood	Number of drought	Province	Number of flood	Number of drought
Anhui	24	4	Jiangxi	42	3
Beijing	5	0	Jilin	11	4
Chongqing	40	2	Liaoning	7	4
Fujian	31	2	Macao	0	0
Gansu	25	4	Ningxia	4	3
Guangdong	43	3	Qinghai	7	1
Guangxi	40	2	Shaanxi	28	5
Guizhou	62	3	Shandong	18	5
Hainan	8	0	Shanghai	2	0
Hebei	8	5	Shanxi	8	6
Heilongjiang	12	2	Sichuan	66	5
Henan	21	2	Taiwan	4	0
Hong Kong	1	0	Tianjin	2	0
Hubei	51	2	Tibet	1	0
Hunan	58	4	Xinjiang	9	0
Inner Mongolia	10	8	Yunnan	43	4
Jiangsu	21	2	Zhejiang	22	2

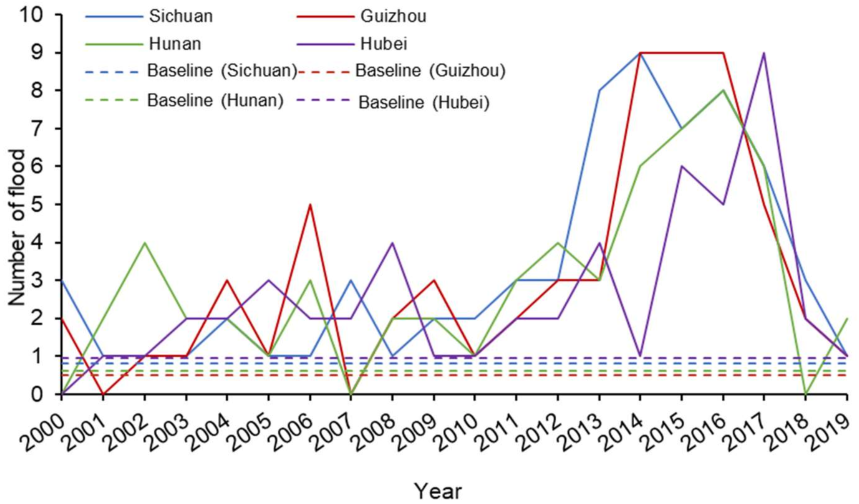

Figure 17: Time trends of flood among four most vulnerable provinces: 2000-2019 (horizontal dashed lines represent the baseline levels of the reference period)

Indicator 1.3: Climate-sensitive infectious diseases

Methods

This indicator focuses on dengue – a notable climate-sensitive vector-borne infectious disease in China. There are three sub-indicators in indicator 1.3 – the climate suitability for *Aedes aegypti (A. aegypti) and Aedes albopictus (A. albopictus),* the vulnerability index to dengue, and the disease burden for dengue in China.

The climate suitability of *A. aegypti and A. albopictus* is represented by Vectorial capacity (VC), which expresses the average daily number of subsequent cases in a susceptible population resulting from one infected case. It is affected by climatic and environmental factors such as land-use type, temperature and rainfall. The VC was calculated according to the method provided by Rocklöv et al.(2019)[34] and Liu-Helmersson et al. (2014)[35]. It takes into account interaction among host, vector and virus. VC is expressed as:

$$VC = ma^2 b_m p^n / - \ln p$$

Where a is the average vector biting rate, b_m is the probability of vector infection and transmission of virus to its saliva, p is the daily survival probability, n is the duration of the extrinsic incubation period-(EIP) , and m is set to 1 assuming female vector and human population as in Watts et al.(2019). [1] Detailed model description and explanation, as well as the relationship between daily temperature with these parameters can be found in Rocklöv et al. (2019).[34] In this study, the time unit is 1 day, and each vector parameter depends on the temperature. The parameter value comes from the literature, usually from experimental data, as described in Liu-Helmersson et al. (2014).[35] The trend of VC time series was analyzed by Mann Kendall trend test. The time unit is 1 season. A two-tailed $p < 0.05$ was considered statistically significant.

The dengue vulnerability index was calculated by dividing VC with average International Health Regulation (IHR) core capacity. The average of IHR core capacity scores is the percentage of attributes of 13 core capacities that have been attained at a specific point in time (presented on an annual basis). It measures the ability to detect, assess, report, inform and deal with public health emergencies. The 13 core capacities of IHR are: (1) National legislation, policy and financing; (2) Coordination and National Focal Point communications; (3) Surveillance; (4) Response; (5) Preparedness; (6) Risk communication; (7) Human resources; (8) Labouratory; (9) Points of entry; (10) Zoonotic events; (11) Food safety; (12) Chemical events; (13) Radionuclear emergencies.

Vulnerability=VC/ average IHR core capacity

National trends for dengue fever are retrieved from the Global Burden of Disease project database over the period 1990-2017 [36] and provincial changes for these diseases between 1990 and 2017.[37] The national trends are presented as incidence rates per 100,000 individuals per year as well as Disability-Adjusted Life Years (DALYs) rates per 100,000 individuals over the period.

Data

1. Monthly average daily temperature (minimums, maximum, and mean) data with the resolution 0.25° from 1961-2018 were from Library for Climate Studies of Chinese Meteorological Administration.[38]
2. The spatio-temporal distributions of *A. aegypti* and *A. albopictus* in 1961-2018 in China were from the China CDC.[39]
3. The IHR core capacity scores from 2010 to 2018 in China were downloaded from WHO website.[40]
4. The national and province-level DALYs rate and incidence rates of dengue over the period use the Global Burden of Disease Study 2017 (GBD 2017) results published for China by the Global Health Data Exchange and provincial-level findings of China published in a systematic analysis in China for GBD 2017.[36,37] The databases were accessed on 29 April 2020.

Additional Information

The average monthly VC for *A. aegypti* and *A. albopictus* in mainland China from 1961 to 2018 are presented in ***Figure 18.*** VC for *A. aegypti* and *A. albopictus* is found to have increased by 37% and 14% respectively when comparing the 2014-2018 average value with the 1961-1965 average value. The overall vulnerability index for two species (*A. aegypti* and *A. albopictus*) had increased slightly by 8% in mainland China over 2010-2018 (***Figure 19***). The vulnerability index for *A. aegypti* is higher than that of *A. albopictus* during the same year.

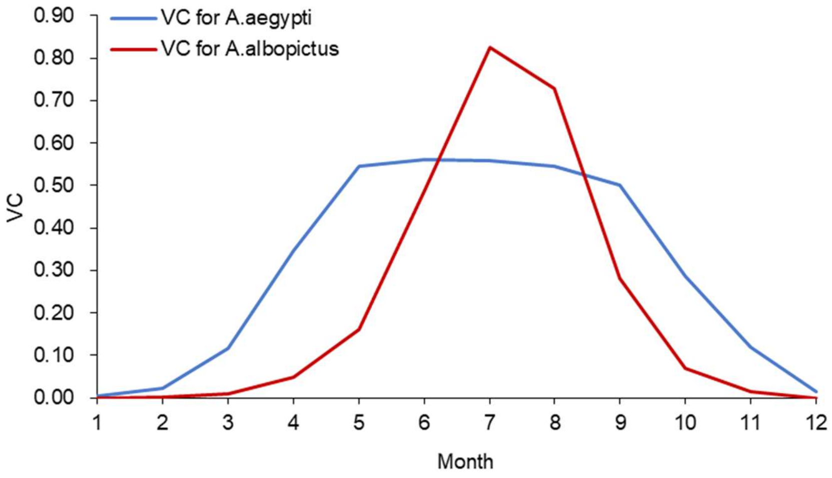

Figure 18: The average monthly VC in mainland China, 1961-2018

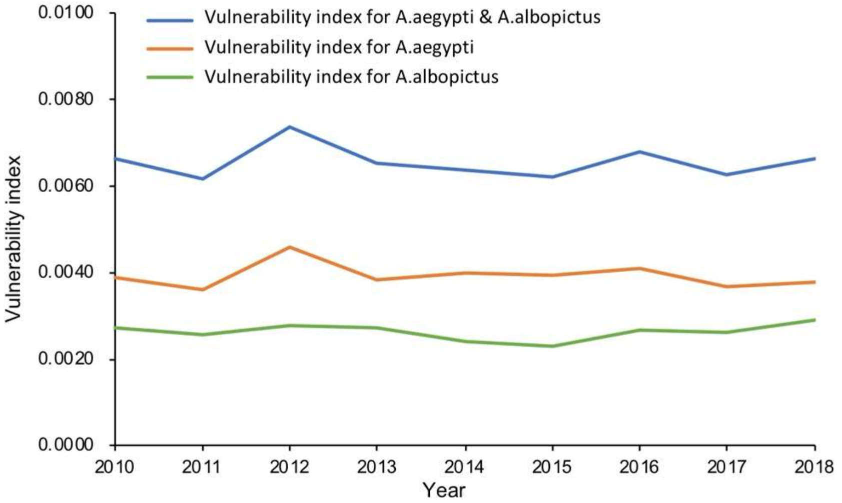

Figure 19: Trends in the annual average vulnerability index for A. aegypti and A. albopictus in mainland China, 2010-2018.

Due to the increased climate suitability, China has witnessed an obvious northward shift of dengue cases (***Figure 20***). The frequency of indigenous dengue outbreak has increased rapidly in China since 2013. In 2019, China experienced an unprecedented multi-sites indigenous outbreak of dengue in 13 provincial-level administrative divisions (PLADs) which significantly higher than the average level (3.67 PLADs per year) from 2013 to 2018.

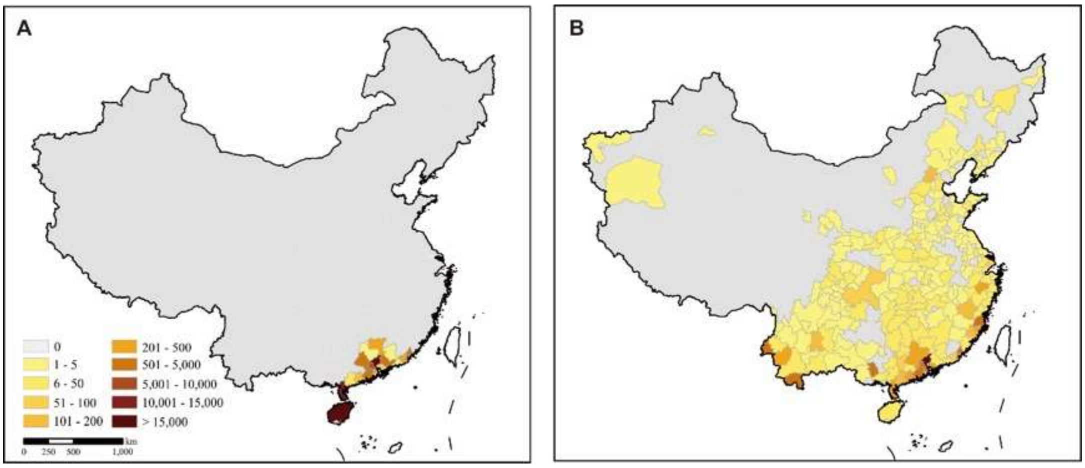

Figure 20: Dengue case numbers distinguished by colour according to the magnitude in each city during 1980-2004 (A) and 2005-2016 (B) (adapted with the permission from authors).[41]

Major caveat of this figure is that it doesn't distinguish local cases with imported cases. But it could still broadly show that dengue has a clear northward trend to the provinces that have no dengue before. The data were unable to be updated till 2019 or 2018 or adapted to have a same baseline as above due to data unavailability.

Table 13: Provincial all-age DALY rate percentage changes between 1990 and 2017 for dengue in China

No.	Location name	All-age DALY rate (per 100 000) percentage change of dengue, 1990-2017*	No.	Location name	All-age DALY rate (per 100 000) percentage change of dengue, 1990-2017*
**1**	China	468.75%	**19**	Jiangxi	NA
2	Anhui	NA	**20**	Jilin	NA
**3**	Beijing	NA	**21**	Liaoning	NA
**4**	Chongqing	NA	**22**	Macau	404.40%
**5**	Fujian	345.53%	**23**	Ningxia	NA
**6**	Gansu	NA	**24**	Qinghai	NA
**7**	Guangdong	359.11%	**25**	Shaanxi	NA
**8**	Guangxi	386.77%	**26**	Shandong	NA
**9**	Guizhou	NA	**27**	Shanghai	NA
**10**	Hainan	339.33%	**28**	Shanxi	NA
**11**	Hebei	NA	**29**	Sichuan	NA
**12**	Heilongjiang	NA	**30**	Taiwan	421.35%
**13**	Henan	NA	**31**	Tianjin	NA
**14**	Hong Kong	382.31%	**32**	Tibet	NA
**15**	Hubei	NA	**33**	Xinjiang	NA
**16**	Hunan	NA	**34**	Yunnan	276.53%
**17**	Inner Mongolia	NA	**35**	Zhejiang	358.97%
**18**	Jiangsu	NA			

*"NA" means that the all-age DALY rate (per 100 000) percentage changes between 1990 and 2017 for dengue can not be calculated in these provinces when the all-age DALY rate (per 100 000) of Dengue in 1990 and 2017 is zero as dengue cases are few.

Caveats

Key caveats and limitations of the VC model and its parameterization are fully described in Liu-Helmersson et al. (2014, 2016)[35,42] and Rocklöv et al., (2019).[34] Overall, the most important limitation is the assumption that the VC is a function of temperature, which should be improved by the more sophisticated model in future. In addition, lacking data concerning IHR core capacities score in each

province of China is another major caveat.

Future Form of Indicator

In future reports, VC can be calculated considering climatic and environmental factors such as factors including temperature and rainfall and local reported dengue cases and monitored vector density in the model synthetically according to different mosquito virus serotypes. New information about data, method and spatial-temporal scale, etc. will be investigated further. In future, the vulnerability index for the provinces with *Ae. aegypti* and *Ae. albopicuts* distribution can be calculated if we can obtain the alternative indicator of IHR at the provincial level.

Section 2: Adaptation, planning, and resilience for health

Indicator 2.1: Adaptation planning and assessment

Indicator 2.1.1: National adaptation plans for health

Methods

A mixed approach, including qualitative analysis of national government documents related to climate change response and a nation-wide China Health and Climate Change Survey for quantitative analysis, was first applied for this indicator. Both documents review and quantitative survey will continue to be conducted annually.

Government documents were searched on the websites of the State Council of PRC, the National Development and Reform Commission, the National Health Commission of PRC *etc.*, and search covered keywords related to climate change, health, adaptation, vulnerabilities, and response *etc.* All of the documents were read through and relevant contents/sections related to climate change and health adaptations were extracted for further analysis. The following national government documents were identified as highly relevant:

- The People's Republic of China. China's National Plan in Response to Climate Change (in Chinese). 2007.
- China's National Development and Reform Commission and eight other ministries. China's National Climate Change Adaptation Strategy (in Chinese). 2013.China's National Development and Reform Commission. China's National Climate Change Planning (2014-2020) (in Chinese). 2014.

The survey questionnaire was designed by the research team from Sun Yat-sen University, adapting the 2018 WHO Health and Climate Change Country Survey. The survey questions related indicator 2.1.1 included "Has your province implemented adaptation plans or strategies to address climate change health risks at the provincial level?", "How is the implementation of relevant policies and strategies?", "What do you think are the main constraints and challenges ahead?", "What do you think should be the priority to implement climate change and health response strategy?". Focus group discussions, key informant consultations were operated at least five times to ensure the validation of the questionnaire. The survey was sent to the provincial Centers for Diseases Control and Prevention in all 31 provinces/regions/municipalities in mainland China in early May 2020, and 17 of them completed the survey.

Data

1. Government documents were retrieved from government websites as described above.
2. Data on provincial adaptation plans or strategies for health was obtained from the nation-wide online voluntary survey conducted by Sun Yat-sen University and China's CDC in early May, 2020.

Caveats

The national online survey related to climate change and health adaptations was conducted in China for the first time in 2020. The survey was not completed by all provinces, regions or municipalities as it was voluntary. It was completed by the provincial Centers for Diseases Control and Prevention in the provinces/regions/municipalities in mainland China, which might only reflect the adaptation plans at local governments' perspectives.

Future Form of Indicator

National reports and documents on climate change and adaptation plans for health will continue to be searched and reviewed annually. The China Health and Climate Change Survey will also be conducted annually and will continue to be the primary source of data to track this indicator 2.1.1. The survey tool could be improved in the future, in terms of the questionnaire validation and response rate.

Additional Information

The Central Government of China issued the National Plan in Response to Climate Change in 2007, which mentions the health impacts of climate change.[43] In 2013, the National Development and Reform Commission (NDRC) and eight other ministries jointly published the National Climate Change Adaptation Strategy, with a section entitled "Human Health", proposing to improve the health and epidemic prevention system, so to provide public weather-health information services.[44] In 2014, the NDRC further implemented the National Climate Change Planning (2014-2020), emphasising the improvement of population adaptability under climate change.[45]

Indicator 2.1.2: National assessments of climate change impacts, vulnerability, and adaptation for health

Methods

A mixed approach, including qualitative analysis of national assessment reports and a nation-wide survey for quantitative analysis, was applied for this indicator.

National reports and documents on assessments of climate change impacts, vulnerability, and adaptation for health released since the year 2000 were systematically searched. The series of reports, "Climate and Environmental Evolution in China", "The National Assessment Report on Climate Change", and "Green Book of Climate Change-Annual Report on Actions to Address Climate Change" were mainly reviewed to qualitatively summarise the national assessment findings.

The provincial-level quantitative data for this indicator is sourced from the China Health and Climate Change Survey as described in indicator 2.1.1.

Data

1. Government documents were retrieved from government websites as described above.
2. Data on provincial assessments of climate change impacts, vulnerability, and adaptation plans for health was obtained from the nation-wide online voluntary survey conducted by Sun Yat-sen University and China's CDC in early May, 2020.

Caveats

The survey was not completed by all provinces as this survey was voluntary; however, the inclusion of 17 provinces in this survey covered most climate zones in China.

Future Form of Indicator

National reports on assessments of climate change impacts, vulnerability, and adaptation for health will continue to be searched and reviewed. The China Health and Climate Change Survey will be conducted annually and will continue to be the primary source of data to track this indicator. The survey tool could be improved in the future, in terms of the questionnaire validation and response rate.

Additional Information

China is facing severe and complex health challenges of climate change. In recent years, the national reports on climate change had involved health as a part of the assessments. Health had been included as a section of a chapter in the report of "Climate and Environmental Evolution in China: 2012"[46] and "The Third National Assessment Report on Climate Change"[47] in 2015.

A scientific assessment of health impacts, vulnerability and adaptation to climate change serves as a baseline analysis of health risks and response measures, which may influence health policymaking and resource allocation.[1] In recent years, the national reports on climate change have involved health as a chapter in the assessment.[46,47] However, the assessment was relatively brief and mainly focused on extreme weather events related to health outcomes and infectious diseases. The projections of health impacts and vulnerability were also included, but the projections were mainly qualitative, and no climate change scenario was used for quantitative estimation.

Of 17 provinces surveyed, only Shanghai reported having completed a province-wide comprehensive assessment of health impact, vulnerability and adaptation to climate change, and five indicated the work had been done in a few cities (*Figure 21).* Heatwave related morbidity or mortality was the most assessed climate-sensitive health outcomes (*Figure 22).* However, only two provinces thought their assessment findings had somewhat influence on the health policy-making, while four indicated the influence was minimal. In terms of the allocation of human and financial resources, only Shanghai indicated the assessment findings had somewhat influence, while others reported minimal or no impact (*Figure 24).*

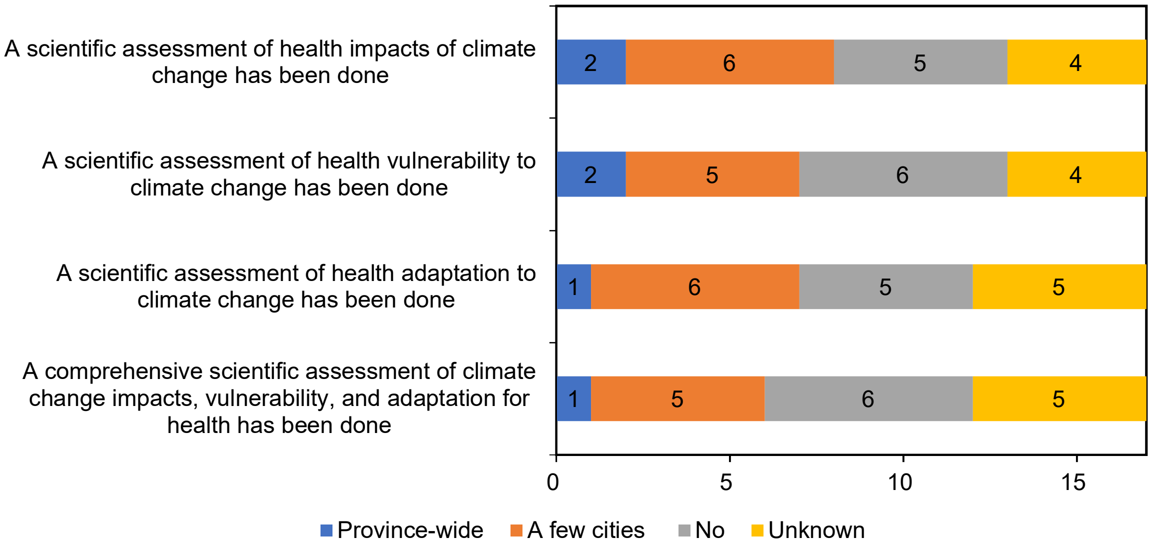

Figure 21: Number of provinces with a scientific assessment of climate change impacts, vulnerability, and adaptation for health

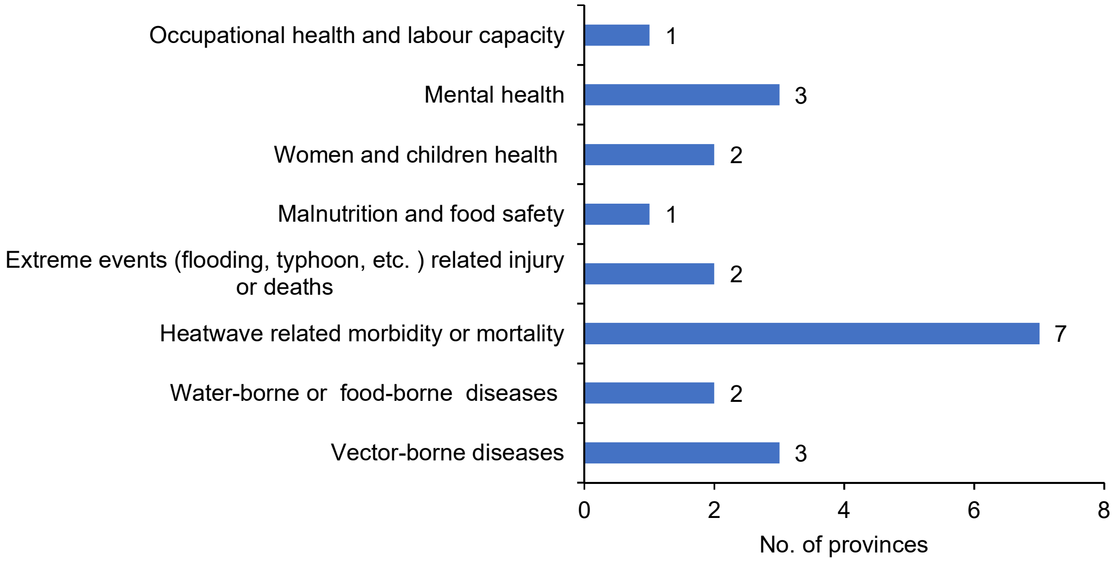

Figure 22: The assessed climate sensitive diseases and health outcomes

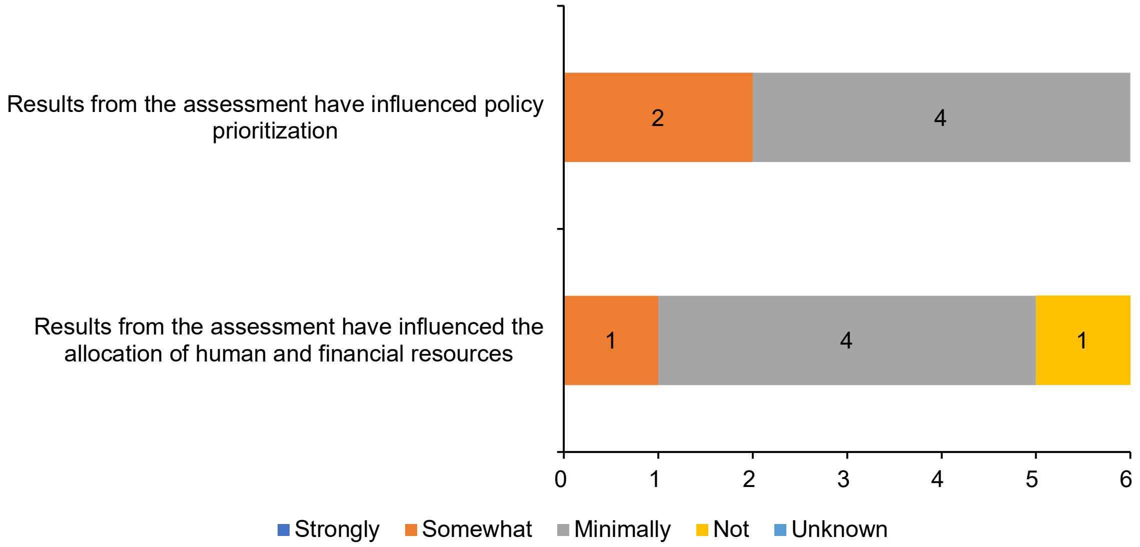

Figure 23: The impacts of assessment findings on the policy prioritisation and the allocation of human and financial resources (Six provinces conducted comprehensive assessment)

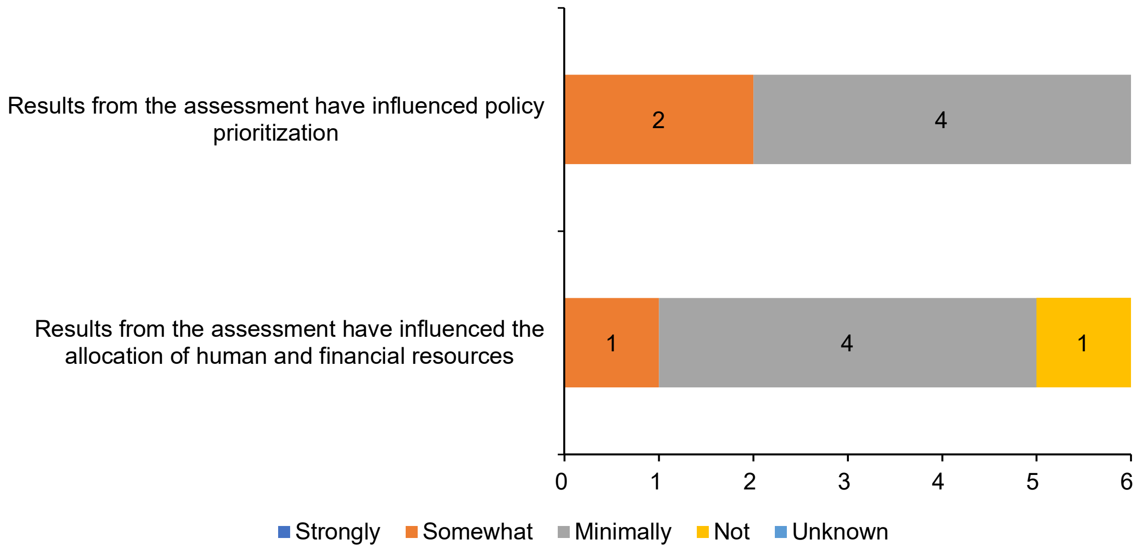

Figure 24: The impacts of assessment findings on the policy prioritisation and the allocation of human and financial resources (Six provinces conducted comprehensive assessment)

Indicator 2.1.3: City-level climate change risk assessments

Methods

Cities have been accommodating an increasing proportion of the total population of China, up to 60 percent by the end of 2019.[48] This indicator is measured with the CDP surveys of global cities in 2017, 2018, and 2019.[49] Fourteen Chinese cities in total joined the annual survey at least once and responded to the related questions about climate change risk or vulnerability assessments and infrastructure affected by climate hazards.

Three sub-indicators were developed based on three questions. The first is the number of the cities that have undertaken a climate change risk or vulnerability assessment, recorded by the 2017, 2018, and 2019 surveys. The second is the number of cities whose climate change risk or vulnerability assessment covering the public health sector, recorded by the 2019 survey. The third is the number of the cities that reported the assets or services related to public health would be affected by climate hazards, recorded in 2018 and 2019 surveys. The methodology follows the 2019 global report of Lancet Countdown but adds a sub-indicator about the inclusion of the public health sector in risk assessments.

Data

1. CDP Annual Cities Survey data

Caveats

Only a small portion of Chinese cities (14 out of more than 600 cities) were involved into the 2017, 2018, and 2019 CDP surveys. The responding cities include Hong Kong, 10 in Taiwan, and 3 in Mainland China. The results lack representativeness.

Future Form of Indicator

The CDP survey is conducted annually. Newly released data will be used with previous data in future reports.

In the future, the 28 pilot cities/counties/districts' climate change risk assessments will be evaluated after the project ends in 2020. The successful cases will be expanded to the whole country. We believe there will be more cities to plan, conduct, and release their own climate change risk assessment. Therefore, a national survey is expected to be designed and conducted to collect responses from city-level governments across China about whether a climate change risk/vulnerability assessment has been undertaken for the city area and whether the health sector has been covered by the assessment.

Findings

Fourteen Chinese cities in total responded to related questions in at least one of the 2017, 2018, and 2019 CDP surveys.

The joint results show 11 (including Hong Kong and 10 in Taiwan) of the 14 cities have undertaken a climate change risk or vulnerability assessment and one (Zhenjiang) in progress, but one (Nanjing) has not undertaken an assessment and one (Shenzhen) has no intention in the future. Concerning the vulnerable sectors, seven cities' completed assessments cover public health. Besides, ten cities in total present that their assets or services related to public health would be affected by climate change.

The great demand of city-level climate change risk assessments for vulnerable areas, groups, and sectors including public health in China has spawned a pilot project from 2017 to 2020 containing 28 cities/counties/districts, which is directed by a national plan jointly issued by Ministry of Housing and Urban-Rural Development (MOHURD) and National Development and Reform Commission (NDRC) in 2016[50, 51].

Table 14: Chinese cities that have undertaken a climate change risk or vulnerability assessment

	2017	2018	2019
Yes (11 cities in total)	Hong Kong, Hsinchu (City)*, Kaohsiung*, New Taipei*, Pingtung*, Taichung*, Taipei*	Hong Kong, Kaohsiung*, New Taipei*, Pingtung*, Taichung*, Taipei*	Hong Kong, Kaohsiung*, Kinmen*, New Taipei*, Pingtung*, Taichung*, Tainan*, Taipei*, Taoyuan*, Yilan*
In progress	Yilan*	Yilan*	Zhenjiang
No	Shenzhen, Taoyuan*	Nanjing	
Not intending to undertake			Shenzhen

*Cities in Taiwan

List of cities whose risk and vulnerability assessment covering the public health sector (seven cities in total)
2019: Hong Kong, Taoyuan*, Taipei*, Tainan*, Taichung*, New Taipei*, Kaohsiung*
(*cities in Taiwan)

List of cities that reported the assets or services related to public health would be affected by climate hazards (10 cities in total)
2018: Hong Kong, Nanjing, New Taipei*, Pingtung*, Taichung*
2019: Hong Kong, Kaohsiung*, New Taipei*, Pingtung*, Taichung*, Tainan*, Taipei*, Taoyuan*, Zhenjiang
(*cities in Taiwan)

List of 28 areas in the pilot project[51] for the *Action Plan for Urban Adaptation to Climate Change*

1. Chaoyang, Liaoning

2. Dalian, Liaoning
3. Hohhot, Inner Mongolia
4. Xifeng District, Qingyang, Gansu
5. Baiyin, Gansu
6. Huangzhong County, Xining, Qinghai
7. Xixian New Area, Shaanxi
8. Shangluo, Shaanxi
9. Baicheng County, Aksu City, Xinjiang
10. Korla, Xinjiang
11. Shihezi, Xinjiang Production and Construction Corps, Xinjiang
12. Huaibei, Anhui
13. Hefei, Anhui
14. Jiujiang, Jiangxi
15. Jinan, Shandong
16. Lishui, Zhejiang
17. Anyang, Henan
18. Wuhan, Hubei
19. Shiyan, Hubei
20. Changde, Hunan
21. Yueyang, Hunan
22. Haikou, Hainan
23. Baise, Guangxi
24. Tongan District, Chongqing
25. Bishan District, Chongqing
26. Hezhang County, Bijie, Guizhou
27. Liupanshui, Guizhou
28. Guangyuan, Sichuan

(Source: http://www.mohurd.gov.cn/wjfb/201702/t20170228_230767.html)

Indicator 2.2: Adaptation delivery and implementation

Indicator 2.2.1: Detection, preparedness, and response to health emergencies

Methods

Because of the consistent structure of the provincial government health emergency management system, the health emergency management and response characteristics are similar among all the provinces of China. However, due to the differences in management efficiency, infrastructure construction and social preparation, different provinces will perform differently. It is very important to analyse these differences, and thus point out the direction of improvement for the government and society. Based on the fundamental of Research and Demonstration of Safety Resilient City Construction and Disaster Prevention Technology (National Key R&D Program of China, Grant No. 2018YFC0809900), from 2019, we started to propose an index system to assess the comprehensive health emergencies management ability of different provinces in China, which suppprted Check-up for China's Cities, an official campaign to promote urban environmental high-quality development launched by Ministry of Housing and Urban-Rural Development of the PRC.. The system includes three dimensions: risk exposure and preparedness, detection and response, resource support and social participation. The index applies to public health emergencies, covering disease outbreaks, mass illness of unknown origin, serious food and occupational poisoning and other emergencies jeopardising public health severely, including the climate-sensitive diseases and mediacal rescue caused by climate-related extreme events. The three dimensions are divided into six second-level indicators and 20 third-level

indicators. The indicators of the index system are listed as follows. The index system could be updated annually by indicators updating and data updating.

Table 15: The indicators of the provincial comprehensive health emergencies management ability index system

First-level Indicators	Second-level Indicators	Third-level Indicators
Risk Exposure and Preparedness(RE&P): the degree of risk faced by the provinces in the health environment and the work done about emergency preparedness.	RE&P 1: Health emergency environmental risks: the health risks due to population mobility and risk management of the provinces.	RE&P 1.1: Proportion of cities identifies as National Health Cities
		RE&P 1.2: Urban population density
		RE&P 1.3: Percentage of migrant population
		RE&P 1.4: Passenger traffic volume
		RE&P 1.5: Number of port entry and exit personnel
	RE&P 2: Health emergency preparedness: the health emergency preparedness of the provinces, in terms of emergency planning, emergency space, and fiscal investment.	RE&P 2.1: Completeness of emergency planning for public health emergencies
		RE&P 2.2: Construction space for emergency facilities
		RE&P 2.3: Percentage of medical and health expenditure out of total government public expenditure
Detection and Response(D&R): the ability for infectious diseases detection and early warming of the provinces, and the health emergency response ability from the perspective of results.	D&R 1: Health emergency detection and early warning: the ability for infectious diseases detection and early warming of the provinces from the perspective of information construction.	D&R 1.1: Construction of Infectious Disease Surveillance Reporting Systems
		D&R 1.2: Availability rate of 4G mobile phone
	D&R 2: Health emergency response: • the management and response to infectious diseases of the provinces.	D&R 2.1: Incidence of category A and B infectious diseases
		D&R 2.2: Death rate of category A and B infectious diseases
Resource Support and Social Participation(RS&SP): the ability to guarantee medical services and the degree of participation of social forces in health care of the provinces.	RS&SP 1: Medical service and resource support: the condition of medical resources and material supplies of the provinces.	RS&SP 1.1: Number of hospitals per 1,000 population
		RS&SP 1.2: Number of primary health care institutions per 1,000 population
		RS&SP 1.3: Number of practicing and assistant doctors per 1,000 population
		RS&SP 1.4: Number of registered nurses per 1,000 population
		RS&SP 1.5: Number of beds in medical and health institutions per 1,000 population
		RS&SP 1.6: Production capacity of pharmaceutical manufacturing industry
	RS&SP 2: Health emergency social participation: the participation of stakeholders in health emergencies.	RS&SP 2.1: Percentage of registered volunteers
		RS&SP 2.2: Number of social organisations in the health sector

The contents and calculation methods of the indicators are described as follows.

- *RE&P 1.1: Proportion of cities identified as National Health Cities*: This indicator is measured by the ratio of the number of National Health Cities in one province to the total number of cities in the province. The National Health City is a national selection carried out every year by Bureau of Disease Control and Prevention, National Health Commission of the PRC. The list of National Health Cities was obtained from the website of National Health Commission of the PRC. The amount of cities was obtained from China Statistical Yearbook.
- *RE&P 1.2: Urban population density*: Urban population density is relevant to the risk of disease spread. It was obtained from China Urban and Rural Construction Statistical Yearbook.

- *RE&P 1.3: Percentage of migrant population*: The percentage of migrant population reflects the risk level of imported infectious diseases and affect community resilience to emergencies. It was obtained from Migrant Population Data Platform, which is an online database provided by Migrant Population Service Center, National Health Commission of the PRC.
- *RE&P 1.4: Passenger traffic volume*: This indicator is measured by the domestic passenger traffic volume per year via one province, including railway, highway and waterway. It's also an indicator reflects the risk level of imported infectious diseases. The data was obtained from China Statistical Yearbook.
- *RE&P 1.5: Number of port entry and exit passengers:* **This indicator is measured by the number of port entry and exit personnel per year via one province, including land ports, waterway ports and air ports. It also reflects the risk level of imported infectious diseases. The data was obtained from China Port Statistical Yearbook.**
- *RE&P 2.1: Completeness of emergency planning for public health emergencies*: This indicator is measured by text analysis to provincial emergency planning for public health emergencies. The results are graded into 0-5 points. The criteria of text analysis include definition of emergencies at different levels, reporting standards, responsibilities and tasks of different departments, mechanisms of emergency response. The text of provincial emergency planning for public health emergencies was obtained from website of general office of provincial government.
- *RE&P 2.2: Construction space for emergency facilities*: The redundancy of construction space for emergency facilities is important when severe epidemic outbreaks. This indicator is measured by the area of urban construction land for municipal utilities per 10,000 population. The data of area of urban construction land for municipal utilities was obtained from China Urban and Rural Construction Statistical Yearbook. The data of population was obtained from China Statistical Yearbook.
- *RE&P 2.3: Percentage of medical and health expenditure out of total government public expenditure*: Fiscal investment is a fundamental work in health emergency preparedness. The data was obtained from China Statistical Yearbook.
- *D&R 1.1: Construction of Infectious Disease Surveillance Reporting System*: Infectious Disease Surveillance Reporting System is a national major project in the field of health emergency response. The system plays an important role in detection, surveillance and rapid reporting to infectious diseases. This indicator is measured by the percentage of counties covered by the system in one province. The data is collected by Chinese Center for Disease Control and Prevention.
- *D&R 1.2: Availability rate of 4G mobile phone*: This indicator is measured by the percentage of population who own a 4G mobile phone. It is a key indicator that reflects the accessibility of warming information. The data was obtained from China Information Almanac.
- *D&R 2.1: Incidence of category A and B infectious diseases*: This indicator is one of the most common used indicator in health emergency response assessment. The infectious diseases are divided into Category A, B and C based on the Law of the People's Republic of China on the Prevention and Treatment of Infectious Diseases[52]. Category A and B infectious diseases are the diseases prevalent and cause casualties easily. The data was obtained from China Health Statistics Yearbook.
- *D&R 2.2: Death rate of category A and B infectious diseases*: This indicator is another one of the most common used indicator in health emergency response assessment. The data was obtained from China Health Statistics Yearbook.
- *RS&SP 1.1: Number of hospitals per 1,000 population*: Hospitals are the major place for health emergency medical treatment. The data was obtained from China Health Statistics Yearbook.
- *RS&SP 1.2: Number of primary health care institutions per 1,000 population*: Primary health care institutions are the major place for early medical treatment and disease prevention. The data was obtained from China Health Statistics Yearbook.
- *RS&SP 1.3: Number of practicing and assistant doctors per 1,000 population*:The number of doctors reflects the ability of treatment for health emergency. The data was obtained from China Health Statistics Yearbook.
- *RS&SP 1.4: Number of registered nurses per 1,000 population*: The number of nurses reflects the ability of nursing for health emergency. The data was obtained from China Health Statistics Yearbook.

- *RS&SP 1.5: Number of beds in medical and health institutions per 1,000 population*: The number of beds in medical and health institutions reflects the admission capacity for health emergency. The data was obtained from China Health Statistics Yearbook.
- *RS&SP 1.6: Production capacity of pharmaceutical manufacturing industry*: The production capacity of pharmaceutical manufacturing industry is important for medical material supplies when severe epidemic outbreaks. This indicator is measured by the annual gross domestic product of pharmaceutical manufacturing industry per 10,000 population. The data of annual gross domestic product of pharmaceutical manufacturing industry was obtained from China Industry Statistics Yearbook.
- *RS&SP 2.1: Percentage of registered volunteers*: Volunteer participation assists the response to health emergency, and it also reflects residents' resilience to health emergency. The data was obtained from the Website of China Volunteer Service, an online platform provided by Ministry of Civil Affairs of the PRC.
- *RS&SP 2.2: Number of social organisations in the health sector*:Social organisations play important roles in the process of heath emergency response. This indicator is measured by the total number of social organisations in the health sector in one province including social groups, foundations and private non-enterprises. These data was obtained from China Civil Affairs' Statistics Yearbook.

To integrate these indictors into an index, we determine weights for all the indicators. We assume the six second-level indicators take equal weights for the index and determine the relative weights of the third-level indicators under the same second-level indicators by Entropy Weigh method (EWM).

The calculation steps of EWM are as follows.

a) Min-Max Normalization.

$$\varphi'_{ij} = \begin{cases} \dfrac{\varphi_{ij} - min\left\{\varphi_{1j}, \varphi_{2j}, \dots, \varphi_{nj}\right\}}{max\left\{\varphi_{1j}, \varphi_{2j}, \dots, \varphi_{nj}\right\} - min\left\{\varphi_{1j}, \varphi_{2j}, \dots, \varphi_{nj}\right\}} & (for\ positive\ indicators) \\ \dfrac{max\left\{\varphi_{1j}, \varphi_{2j}, \dots, \varphi_{nj}\right\} - \varphi_{ij}}{max\left\{\varphi_{1j}, \varphi_{2j}, \dots, \varphi_{nj}\right\} - min\left\{\varphi_{1j}, \varphi_{2j}, \dots, \varphi_{nj}\right\}} & (for\ negative\ indicators) \end{cases}$$

φ_{ij} is the original data of the jth third-level indicator of the ith province, n is the amount of provinces. A positive indicator is an indicator that larger value means better result, while a negative indicator is an indicator that larger value means worse result.

b) Calculate the proportion of normalised sample value.

$$p_{ij} = \frac{\varphi'_{ij}}{\sum_{i=1}^{m} \varphi'_{ij}} \quad (j = 1, 2, \dots m)$$

m is the amount of the third-level indicators under the same second-level indicator.

c) Calculate the entropy of indicators.

$$e_j = -\frac{1}{\ln n} \sum_{i=1}^{n} p_{ij} \ln\left(p_{ij}\right) \quad (j = 1, 2, \dots m)$$

e_j is the entropy of the jth third-level indicator.

d) Calculate the entropy redundancy of indicators.

$$d_j = 1 - e_j$$

d_j is the entropy redundancy of the jth third-level indicator.

e) Determine the relative weights of indicators.

$$w_j = \frac{d_j}{\sum_{j=1}^{m} d_j} \quad (j = 1, 2, \ldots m)$$

The relative weights of third-level indicators are shown below.

Table 16: Relative weights of third-level indicators under the same second-level indicator

First-level Indicators	Second-level Indicators	Third-level Indicators	Relative weights
Risk Exposure and Preparedness(RE&P)	RE&P 1: Health emergency environmental risks	RE&P 1.1: Proportion of the National Health City	0.570
		RE&P 1.2: Urban population density	0.149
		RE&P 1.3: Percentage of migrant population	0.113
		RE&P 1.4: Passenger traffic volume	0.131
		RE&P 1.5: Number of entry and exit passengers	0.037
	RE&P 2: Health emergency preparedness	RE&P 2.1: Completeness of emergency planning for public health emergencies	0.231
		RE&P 2.2: Construction space for emergency facilities	0.563
		RE&P 2.3: Percentage of medical and health expenditure in government public expenditure	0.206
Detection and Response(D&R)	D&R 1: Health emergency detection and early warning	D&R 1.1: Construction of Infectious Disease Surveillance Reporting System	0.280
		D&R 1.2: Availability rate of 4G mobile phone	0.720
	D&R 2: Health emergency response	D&R 2.1: Incidence of category A and B infectious diseases	0.357
		D&R 2.2: Death rate of category A and B infectious diseases	0.643
Resource Support and Social Participation(RS&SP)	RS&SP 1: Medical service and resource support	RS&SP 1.1: Number of hospitals per 1,000 population	0.175
		RS&SP 1.2: Number of primary health care institutions per 1,000 population	0.152
		RS&SP 1.3: Number of practicing and assistant doctors per 1,000 population	0.163
		RS&SP 1.4: Number of registered nurses per 1,000 population	0.080
		RS&SP 1.5: Number of beds in medical and health institutions per 1,000 population	0.130
		RS&SP 1.6: Production capacity of pharmaceutical manufacturing industry	0.299
	RS&SP 2: Health emergency social participation	RS&SP 2.1: Percentage of registered volunteers	0.293
		RS&SP 2.2: Number of social organisations in the health sector	0.707

Data

Unless otherwise specified, the most recent version of data available is used in this study.

1. The list of National Health Cities is obtained from the website of National Health Commission of the PRC (http://www.nhc.gov.cn/). The most recent available version is the list of 2018.
2. Data for total cities, population, passenger traffic volume (including railway, highway and waterway), and percentage of medical and health expenditure in government public expenditure is taken from the China Statistical Yearbook. The most recent available version is China Statistical Yearbook 2019[53], which contains the data of every province in 2018.
3. The data on urban population density and area of urban construction land for municipal utilities is based on China Urban and Rural Construction Statistical Yearbook. The most recent available version is China

Urban and Rural Construction Statistical Yearbook 2018,[54] which contains the data of every province in 2018.

4. The data on the percentage of migrant population is based on the website of Migrant Population Data Platform (http://www.chinaldrk.org.cn/wjw/#/home). The most recent available data is based on the Sixth National Census of China.
5. The data on the number of entry and exit personnel at the port is based on China Port Statistical Yearbook. The most recent available version is China Port Statistical Yearbook 2016,[55] which contains the data of every province in 2016.
6. The text of provincial emergency planning for public health emergencies is taken from the websites of the general office of every provincial government.
7. The percentage of counties covered by Infectious Disease Surveillance Reporting System is collected by Chinese Center for Disease Control and Prevention.
8. The data on the percentage of population available to a 4G mobile phone is based on China Information Almanac. The most recent available version is China Information Almanac 2017,[56] which contains the data of every province in 2016.
9. The data on the incidence of category A and B infectious diseases, the death rate of category A and B infectious diseases, the number of hospitals, the number of primary health care institutions number of practicing and assistant doctors, the number of registered nurses and number of beds in medical and health institutions is based on China Industry Statistics Yearbook. The most recent available version is China Health Statistics Yearbook 2019,[57] which contains the data of every province in 2018.
10. The data on annual gross domestic product of pharmaceutical manufacturing industry is based on China Industry Statistical Yearbook. The most recent available version is China Industry Statistical Yearbook 2017,[58] which contains the data of every province in 2016.
11. The data of percentage of registered volunteers is based on the Website of China Volunteer Service (https://npo.chinavolunteer.cn). The data we use in this study was obtain on 2020-05-05.
12. The data on the number of social organisations in the health sector (including social groups, foundations and private non-enterprises) is taken from the China Civil Affairs' Statistics Yearbook. The most recent available version is China Civil Affairs' Statistics Yearbook 2017,[59] which contains the data of every province in 2016.

Caveats

In this study, the data of most third-level indicators are based on 2018. But limited by the availability of data, the data of some third-level indicators is based on 2016.

Future Form of Indicator

A Time-Series Analysis could be done in the future. And more indicators about comprehensive health emergencies management assessment could be considered. All the data we adopt in this year is collected by official government and could be updated in the next years.

Additional Information

This assessment covers all the other 31 provinces of China except Hong Kong, Macau, Taiwan. The index results and rank of provincial comprehensive health emergencies management ability are listed below. The results present regional differences and take the order of East China, North China, Northeast China, South Central China, Northwest China and Southwest China from higher to lower. The average index result of provinces in East China, North China, Northeast China, South Central China, Northwest China and Southwest China are 55.24, 50.77, 48.29, 45.46, 44.82 and 42.15 respectively.

Table 17: Index results and rank of provincial comprehensive health emergencies management ability

Region	Province	Index result	Rank
North China	Beijing	60.87	3
	Tianjin	42.92	24
	Hebei	51.82	11
	Shanxi	47.49	16
Northeast China	Jilin	51.87	10
	Liaoning	49.46	13
	Heilongjiang	43.31	23
	Inner Mongolia	48.54	14
East China	Shanghai	54.29	6
	Jiangsu	69.72	1
	Zhejiang	54.89	4
	Anhui	52.99	8
	Fujian	45.62	19
	Jiangxi	40.26	28
	Shandong	68.91	2
	Taiwan	——	——
South Central China	Henan	54.35	5
	Hubei	49.72	12
	Hunan	41.93	25
	Guangdong	46.16	18
	Guangxi	35.16	29
	Hainan	45.44	20
	Hong Kong	——	——
	Macau	——	——
Southwest China	Sichuan	48.22	15
	Guizhou	45.23	21
	Yunnan	41.50	27
	Chongqing	43.58	22
	Tibet	32.23	30
Northwest China	Shaanxi	52.26	9
	Gansu	47.30	17
	Qinghai	41.73	26
	Ningxia	53.40	7
	Xinjiang	29.40	31

Indicator 2.2.2: Air conditioning - benefits and harms

Methods

The benefit of air conditioning is associated with the prevention of heatwave-related mortality. The methodology of the prevented fraction calculation is the same as the 2019 global Lancet Countdown report, using the data on the proportion of households with air conditioning in China, as well as a relative risk for heatwave-related mortality of 0.23.

The harms of air conditioning mainly include the increasing energy consumption and CO_2 emissions. The energy consumptions of urban household air conditioning from 2001 to 2015 in China were kindly provided by the Building Energy Conservation Research Center in Tsinghua University, which can represent the intensity of harms due to air conditioning. The CO_2 emission attributable to air conditioning can be calculated by the conversion coefficient between CO_2 emission and electricity consumption (i.e. 0.785kg CO_2 emission per 1kWh electricity consumption of air conditioning).

Data

1. The IEA kindly provided data on the proportion of households with air conditioning in China, which can be used to calculate the prevented fraction of heatwave-related mortality due to air conditioning in China.
2. The energy consumption of urban household air conditioning in China was from the Annual Report on China Building Energy Efficiency by the Building Energy Conservation Research Center in Tsinghua University.

Caveats

Firstly, for the prevented fraction calculation, the value of relative risk (RR) was assumed to be the same as the global scenario. Although the RR due to heatwave in China has been analysed by several Chinese researchers, the results of RR were inconsistent, which varied significantly from 0.91 to 1.34, because of different definitions of heatwave, death categories, genders, age groups and lag periods.[60,61] Furthermore, the application of different values of RR would be inconducive to the international comparison of heatwave-related mortality prevention of air conditioning. For these purposes, the value of RR in this study were still kept the same as that in 2019 global Lancet Countdown report.

Secondly, the prevented fraction of heatwave-related mortality due to air conditioning discussed here was not reconciled with the heat-related mortality in section one, because of different data sources. The former was calculated based on data from the IEA, while the latter used data from other published papers. As research continues, work will be undertaken to make these two indicators consistent in the future.

Thirdly, the calculation of energy consumption of air conditioning did not include commercial buildings and rural areas.

Fourthly, this indicator cannot be broken down to the city level yet due to the inaccessibility of more detailed data.

Future Form of Indicator

This indicator will be improved to reconcile the heatwave-related mortality (indicator 1.1.3) with the prevented fraction discussed here.

Additional Information

In 2015, the total energy consumption of urban household air conditioning in China was 74.5 billion kWh, which accounted for 12% of the total energy consumption of urban residential sector.[62] The large amount of energy consumption and CO_2 emissions due to air conditioning inevitably enhances urban heat island effect and contributes to climate change.

Indicator 2.3: Climate information services for health

Methods

This indicator is measured with web traffic to the website of the National Emergency Early Warning Information Release System (http://www.12379.cn/), [63] established in 2015 that releases meteorological services, specifically, the yearly total numbers of page views for the website in 2018 and 2019. The system releases more than 20 types of meteorological warnings at the national, provincial, city, and county levels. The warnings for some meteorological hazards like cold wave, high temperature, and haze are released with health suggestions for sake of risk prevention.

Data

1. The data of web traffic to the website of the National Emergency Early Warning Information Release System (http://www.12379.cn/), specifically, the yearly total number of page views for the website separately for 2018 and 2019, was provided by the China Meteorological Administration (CMA).

Caveats

This method only captures the public's active use of the website of the National Emergency Early Warning Information Release System. However, the system releases meteorological warnings via multiple channels not limited to this website. Moreover, the proportion of the website usage for health-related warnings is unknown.

Future Form of Indicator

In future reports, this indicator will be improved to better assess the sensitivity and effectiveness of climate information services for health. The presence of provincial level early warning information release systems could be assessed. Warnings for specific climate change and health exposure pathways could be linked with indicators in section 1 of the report to determine the proportion of events identified in section 1 are addressed by the National Emergency Early Warning Information Release System. In the future, the traffic data for different warning release channels adopted by the National Emergency Early Warning Information Release System could also be collected for a more comprehensive delineation of the public use of the system.

Findings

The web traffic data offered by the website operator, the China Meteorological Administration (CMA), was used for this indicator. The meteorological warnings released in 2018[64] and 2019[65] accounted for 96% and 97% of the respective total warnings across the country (270,256 and 261,959, separately) released by the system. The warnings for health-related hazards[66] like high temperature (8.65%), cold wave (3.17%), haze (1.11%), and sandstorm (0.25%) occupied 13% of the total meteorological warnings in 2019.[65] Along with the multi-level warnings, health suggestions are provided.[67]

The total number of page views for the website throughout a year sharply increased from 3.8 million in 2018 to 8.7 million in 2019. The figures could, to some extent, measure the popularity of the national warning release platform for health purposes, although the system releases warnings via multiple channels including short message service, mobile applications and etc.

Table 18: Health suggestions for health-related meteorological hazards

Warning	Health suggestions

**High temperature**	preventing heatstroke, reducing outdoor activities, providing specific suggestions and protections for vulnerable groups
**Cold wave**	keeping warm, taking care of vulnerable groups
**Haze**	wearing masks, people with respiratory diseases reducing outdoor activities
**Sandstorm**	wearing masks, people with respiratory diseases or allergic to sand staying indoor

Note: The National Emergency Early Warning Information Release System releases warnings of meterological hazards apart from these four hazards. . Health suggestions are provided for these four hazards. The health suggestions were summarized based on Trial Procedures on the Release of Early Warning Signals for Unexpected Meteorological Disasters promulgated by the China Meteorological Administration in 2004.

Section 3: Mitigation actions and health co-benefits

Carbon intensity of the economic system

Methods

This indicator contains two components:

1. Carbon intensity (CI) of the economic system, both at national (2000-2019) and regional (six regions) (2000-2017) scales, in kgCO_2/US$; and
2. National CO_2 emissions from energy combustion by fuel and industrial process (mainly cement), in MtCO_2 (2000-2019).

The technical definition of CI is the kilograms (kg) of CO_2 emitted for each unit (US$) of GDP. The rationale for the indicator choice is that carbon intensity of the economic system will provide information on the level of fossil fuel use, which has associated air pollution impacts. Higher intensity values indicate a more fossil dominated economic system, and one that is likely to have a higher coal share. As countries pursue climate mitigation goals, the carbon intensity is likely to reduce with benefits for air pollution. The indicator is calculated based on total CO_2 emissions from fossil fuel divided by Gross Domestic Product (GDP)). GDP reflects the economic development status in an area/country.

CO_2 emissions of China and 30 provinces (excluding Tibet) from 2000 to 2017 are calculated by sectoral approach and reference approach individually, while in year 2018 and 2019, CO_2 emissions of China are calculated by reference approach. Below is the equation for CI:

$$CI_{st} = CO2_{st}/GDP_{st}$$

Where s denotes region; t represents year; $CO2_{st}$ denotes CO_2 emission in s in t; GDP_{st} represents the GDP in s in t which is collected from China Statistical Yearbook [68] .

CO_2 emissions data by sectoral/reference approach during 2000 to 2017 is collected from China Emission Accounts and Datasets (CEADs, www.ceads.net);[69,70] CO_2 emissions data of China from 2018 to 2019 is calculated by reference approach and for details of this approach see Shan et al. (2018).[69] The daily CO_2 emissions of China in 2019 and 2020 is taken from https://arxiv.org/abs/2004.13614.

Data

1. Energy balance tables are taken from China Energy Statistical Yearbook 2001-2018;[68]
2. CO_2 emissions are taken from China Emission Accounts and Datasets (CEADs, www.ceads.net).[69,70]
3. The daily CO_2 emissions of China in 2019 and 2020 is taken from https://arxiv.org/abs/2004.13614.

Caveats

CO_2 emissions of China in 2018 and 2019 are estimated by reference approach and they may be overestimated. Compared sectoral emissions, reference emissions were 1-7% higher for three key reasons. Firstly, the energy

loss during energy transformation process is not excluded from the reference energy consumption.[69] Secondly, only transport loss and nonenergy usage of primary energy sources are excluded from the total consumption in the reference approach, without the removeal of secondary energy sources. Finally, there is roughly a 1.2% difference between the energy production and consumption data in China's energy balance table. According to the data from 2000 to 2017 provided by CEADs (www.ceads.net), the CO_2 emissions of China by reference approach is an average of 3.4% higher emissions estimated by sectoral approach.

Future Form of Indicator

This indicator for provinces will need to be updated to provide the data for the most recent years.

Additional Information

CI of China from 2000 to 2019 is generally decreasing (***Figure 25***). The reason behind this is that the average annual increase rate of CO_2 emissions of China (7.3%) is lower than that of GDP (8%). CO_2 emissions from fossil fuel combustion in China rose rapidly between 2000 and 2012, then fluctuated between 2013-2017 (by sectoral approach), and increased by 2.7 % from 2018 to 2019 (by reference approach) ***(Figure 25)***.[69,70] However, CO_2 emissions from natural gas combustion has increased by 1.6 times from 2000 to 2019 by reference approach. [69,71]

The CI of six regions is also generally decreasing. However, the CI in Northwest and Northeast China has increased by 4.1% and 0.2% respectively in 2017 compared to 2016, with large fluctuations in CI seen in Northwest China from 2003 to 2017. The CI of Northwest China because Ningxia province lacked the CO_2 emissions data from 2000 to 2002. At 1.2kg/US$1 in 2017, East China had the lowest CI, while Northwest China had the highest CI at 4 kg/US$.

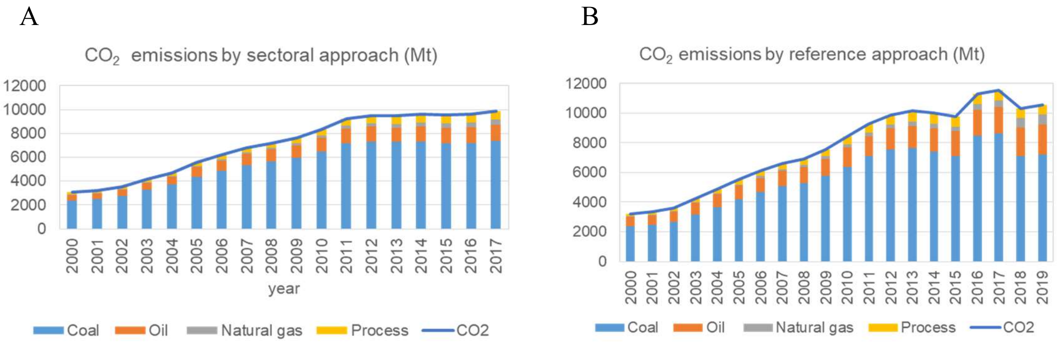

Figure 25. CO_2 emissions of China calculated by (A) sectoral approach (2000-2017) and (B) reference approach (2000-2019)

Indicator 3.1: The energy system and health

Indicator 3.1.1: Coal phase-out

Methods

Two indicator componentsare used here:
1. Total primary supply of coal in China and by province (in EJ units); and
2. Share of coal in total primary energy supply.

The indicator on primary energy supply of coal is an aggregation of all coal types used across all sectors, with data taken from annual editions of Energy Statistical Yearbook of China. The share of coal in total

energy supply is estimated by dividing primary energy supply of coal across all sectors by total primary energy supply.

The data for both indicator components is available for the period 2000-2018 at the national level, and for the period 2000-2017 for each province.

Data

1. The data for this indicator is taken from annual edition of Energy Statistical Yearbook of China.[72]

Caveats

These indicators provide a proxy for air quality emissions associated with the combustion of coal. Further work is required to convert coal use by sector and type into emissions of different air quality pollutants.

Future Form of Indicator

In the future, this indicator set could be developed to also estimate the actual air pollutant emissions associated with coal use. This could be estimated using the GAINS model, with inputs of sectoral use, coal type (both of which are available) and appropriate emission factors.

Additional Information

Due to the energy use from all sources, the overall coal share in China's TPES continued to decline, from 72% in 2005 (the highest share during 2000-2018) to 66% in 2014, and further to 59% (***Figure 26***) in 2018, reduced by 14% during 2000-2018. From regional perspectives, Beijing-Tianjin-Hebei area and East China provinces along the coastline have cut their coal use substantially in recent years, while a continuous increase was observed in Northwestern provinces, (***Figure 26***). As a result of the COVID-19 pandemic, demand for coal fell by 6.8% in the first quarter of 2020 in China,[73] and is projected to decline by 5% throughout the year.[74]

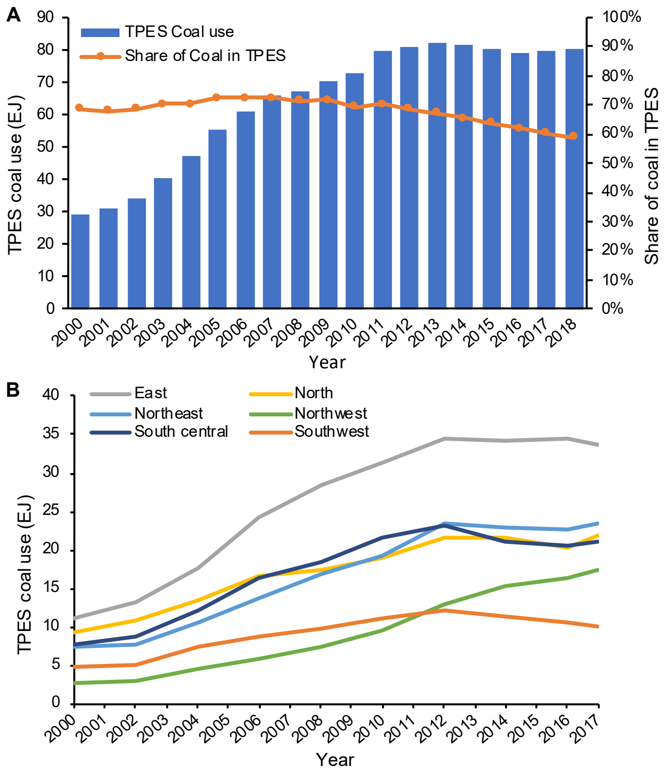

Figure 26: National and regional Total Primary Energy Supply (TPES) from coal (2000-2018). (A) TPES from coal and the proportion in TPES in China; (B) TPES from coal in six regions.

Table 19 Coal consumption by province, 2010-2017, Unit: PJ

Province	2010	2011	2012	2013	2014	2015	2016	2017
Beijing	551.5	495.3	475.2	422.7	363.5	243.9	177.4	102.7
Tianjin	1006.2	1101.5	1109.0	1105.0	1052.4	950.1	885.5	811.3
Hebei	5749.3	6445.8	6564.5	6628.2	6203.7	6058.7	5883.4	5739.3
Shanxi	6251.7	7008.3	7232.7	7669.2	7868.3	7769.4	7456.6	8989.2
Inner Mongolia	5652.8	7260.5	7665.8	7309.0	7633.5	7640.6	7677.3	8079.3
Liaoning	3539.5	3779.3	3813.8	3795.8	3768.5	3629.1	3546.9	3681.6
Jilin	2006.0	2310.0	2320.0	2179.9	2172.7	2052.6	1971.3	1958.2
Heilongjiang	2557.9	2763.2	2923.3	2777.2	2846.0	2811.9	2937.9	3028.8
Shanghai	1229.9	1285.7	1193.8	1189.3	1024.8	989.8	968.3	958.3
Jiangsu	4835.7	5728.2	5811.5	5850.0	5633.7	5695.8	5871.4	5572.4
Zhejiang	2920.2	3093.1	3008.9	2964.4	2893.9	2894.2	2919.9	2985.5
Anhui	2800.0	3043.3	3078.0	3279.2	3304.7	3280.5	3292.5	3367.0
Fujian	1470.8	1824.1	1776.2	1691.1	1716.2	1603.5	1429.0	1579.0
Jiangxi	1307.5	1462.8	1423.9	1518.6	1565.2	1611.5	1594.6	1624.7
Shandong	7814.0	8147.4	8422.1	7888.4	8281.6	8567.3	8569.9	7989.1
Henan	5453.1	5939.6	5283.6	5245.5	5076.3	4965.4	4862.1	4745.4
Hubei	2819.7	3308.5	3307.2	2546.9	2488.5	2463.0	2446.2	2465.3
Hunan	2370.3	2722.6	2529.6	2349.5	2281.6	2332.4	2395.5	2596.7

Guangdong	3345.9	3859.9	3691.4	3581.0	3561.5	3472.3	3377.6	3594.7
Guangxi	1299.3	1472.2	1520.6	1537.4	1422.7	1265.8	1364.4	1384.4
Hainan	135.5	170.6	194.9	211.2	213.2	224.4	212.5	230.1
Chongqing	1339.1	1504.9	1413.0	1213.0	1276.0	1265.9	1187.8	1182.0
Sichuan	2411.6	2397.7	2485.2	2444.7	2312.2	1944.5	1856.7	1644.5
Guizhou	2283.4	2529.8	2790.0	2857.5	2745.9	2686.5	2855.9	2807.1
Yunnan	1957.1	2023.0	2061.9	2047.9	1815.9	1614.6	1561.9	1509.6
Shaanxi	2436.3	2787.9	3302.0	3610.6	3846.6	3846.2	4117.7	4201.2
Gansu	1128.2	1319.4	1372.8	1369.3	1405.9	1372.6	1335.0	1331.5
Qinghai	266.0	315.7	389.1	434.0	380.3	315.7	410.8	365.7
Ningxia	1206.8	1663.6	1686.2	1786.3	1854.1	1864.6	1813.9	2314.8
Xinjiang	1696.9	2039.9	2517.9	2973.7	3367.8	3633.9	3974.2	4264.1

Note: (1) data for Tibet is not available. (2) Due to statistical difference, provincial sum does not equal to national total.

Indicator 3.1.2: Low-carbon emission electricity

Methods

Two indicators are used here, and presented in two ways:

1. Total low-carbon electricity generation (including solar, wind, hydropower and nuclear), in absolute terms (TWh) and as a % share of total electricity generated; and
2. Total renewable generation (excluding hydro), in TWh, and as a % share of total electricity generated.

The increase in the use of low carbon and renewable energy for electricity generation will push other fossil fuels, such as coal, out of the mix over time, resulting in an improvement in air quality, with benefits to health. The renewables (excluding hydro) indicator has been used to allow for the racking of rapidly emergent renewable technologies. For both indicators, generation, rather than capacity, has been chosen as a metric as the electricity generated from these technologies is what actually displaces fossil-based generation.

Due to lack of electricity generation data at the provincial level, six provinces with the highest GDP in 2019[75] we selected to represent their region (Beijing for North; Liaoning for Northeast; Jiangsu for East; Guangdong for South central; Sichuan for Southwest; Shaanxi for Northwest). The data is taken from the China energy balance datasets.[76] The absolute level indicators are the total gross electricity generated, aggregating the relevant technology types. The share indicators are estimated as the low carbon or renewable generation as a % of total generation.

Data

1. The data is taken from the China energy balance datasets. [76]

Caveats

1. Solar, wind and nuclear generation data from the National Bureau Statistics of China is only available from 2015.
2. This indicator set does not provide information on the air pollutant emissions displaced due to the

increasing share of renewable generation.

Future Form of Indicator

Detailed data of provinces should be updated to get the accurate regional results.

Additional Information

Low-carbon electricity in China and six regions has been generally increasing from 2015 to 2019.From 2000 to 2014, the only low-carbon electricity reported in China was hydropower, which accounts for a larger share of low-carbon electricity than renewable energy (57.1% ~74.09% of total low-carbon electricity) from 2015 to 2019, but its share has declined during this period. The national share of renewable energy increased annually. In 2019, low-carbon electricity nationally accounted for 31.13% of total China electricity generation). From 2018 to 2019, renewable energy increased by 16.77% while hydropower increased by 5.67%.

As costs continue to fall, solar generation continues to grow at remarkable rates of around 26.51 % but still only accounts for 3.06 % of total generation. Among 30 provinces of China. At 11.2 TWh in 2019, Qinghai produces the most solar power.[76] Northwest China is the area that provides the most solar power in China due to appropriate natural environment for solar generation. Southwest and South Central regions generate more hydropower than northern provinces, mainly because that there are more rivers, a more favorable terrain and a more humid climate in the southern area of China than the northern area. However, it should be noted that in the Northeast China also has abundant hydropower.

The shares of low-carbon electricity in Northeast and South Central China were higher than national average, but they had different shares of types of renewable energy in their mix mainly due to their resource endowment and socioeconomic development level. In Liaoning, nuclear, wind and solar generation made up 66.15%, 30.91% and 2.88% of low-carbon electricity, respectively; while nuclear, wind and solar generation were 92.17% , 5.76% and 2.07% of low-carbon electricity in Guangdong.

The detailed data of different sources of electricity generation in China and other six regions see Table 20 to Table 26

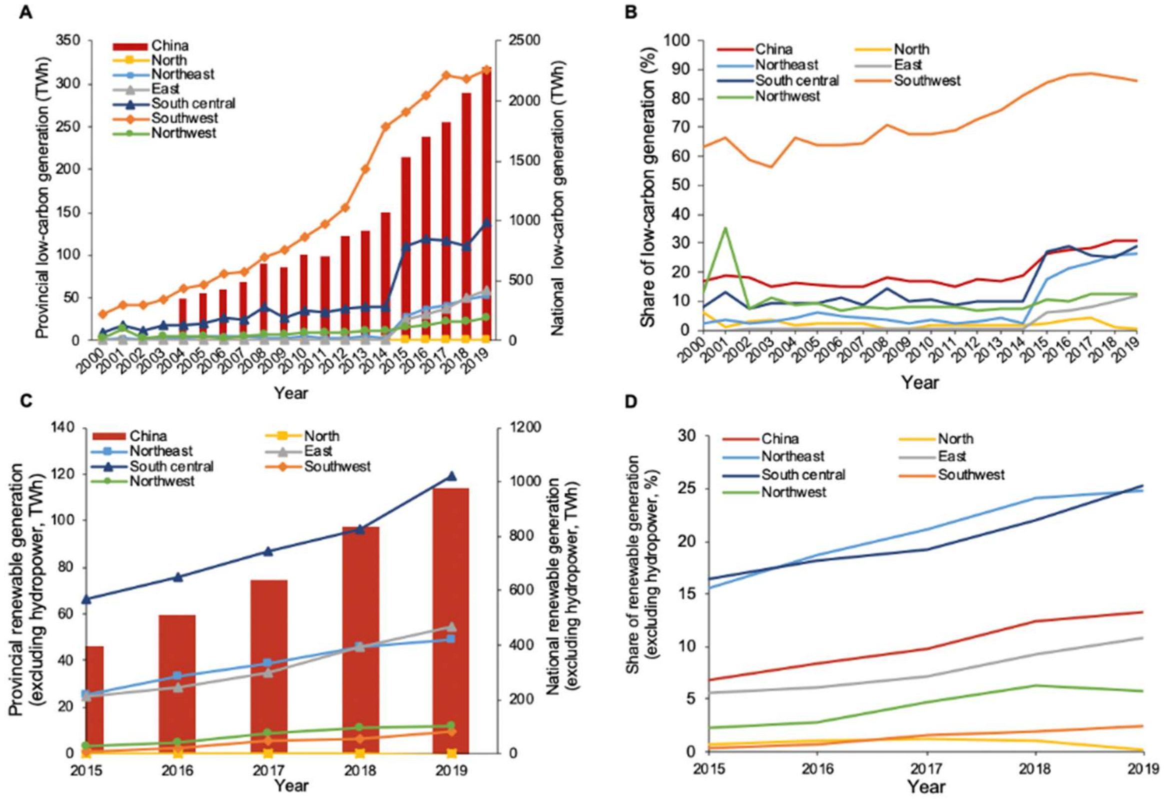

Figure 27: Renewable and low-carbon emission electricity generation
(A) Electricity generated from low-carbon sources. (B) Share of electricity generated from low-carbon sources. (C) Electricity generated from renewable sources (excluding hydropower). (D) Share of electricity generated from renewable sources (excluding hydropower). TWh=terawatt hours.

Table 20: Different sources of electricity generation in China (TWh)

Year	Hydropower	Nuclear	Wind	Solar	Thermal power	Low carbon generation	Renewable generation	Total generation
2000	222.4	0.0	0.0	0.0	1088.5	222.4	0.0	1328.7
2001	277.4	0.0	0.0	0.0	1176.8	277.4	0.0	1480.8
2002	288.0	0.0	0.0	0.0	1328.8	288.0	0.0	1602.4
2003	283.7	0.0	0.0	0.0	1580.4	283.7	0.0	1910.6
2004	353.5	0.0	0.0	0.0	1795.6	353.5	0.0	2203.3
2005	397.0	0.0	0.0	0.0	2047.3	397.0	0.0	2500.1
2006	435.8	0.0	0.0	0.0	2369.6	435.8	0.0	2865.9
2007	485.3	0.0	0.0	0.0	2722.9	485.3	0.0	3281.6
2008	637.0	0.0	0.0	0.0	2707.2	637.0	0.0	3495.8
2009	615.6	0.0	0.0	0.0	2982.8	615.6	0.0	3714.7
2010	722.2	0.0	0.0	0.0	3331.9	722.2	0.0	4207.0
2011	698.9	0.0	0.0	0.0	3833.7	698.9	0.0	4712.9
2012	872.1	0.0	0.0	0.0	3892.8	872.1	0.0	4987.7
2013	920.3	0.0	0.0	0.0	4247.0	920.3	0.0	5431.6
2014	1072.9	0.0	0.0	0.0	4400.1	1072.9	0.0	5794.3
2015	1130.3	170.8	185.8	38.8	4284.2	1525.6	395.3	5814.9
2016	1184.0	213.3	237.1	61.6	4437.1	1696.0	511.9	6133.0
2017	1189.8	248.1	295.0	96.7	4662.7	1829.6	639.8	6451.1

2018	1232.1	295.0	365.8	176.9	4924.9	2069.8	837.7	6769.2
2019	1301.9	348.7	405.7	223.8	5045.0	2280.1	978.2	7325.3

Table 21: Different sources of electricity generation in Beijing (representing North China) (TWh)

Year	Hydropower	Nuclear	Wind	Solar	Thermal power	Low carbon generation	Renewable generation	Total generation
2000	0.9	0.0	0.0	0.0	13.7	0.9	0.0	14.5
2001	0.2	0.0	0.0	0.0	13.0	0.2	0.0	13.3
2002	0.4	0.0	0.0	0.0	13.6	0.4	0.0	14.2
2003	0.7	0.0	0.0	0.0	18.6	0.7	0.0	19.2
2004	0.4	0.0	0.0	0.0	19.8	0.4	0.0	20.4
2005	0.5	0.0	0.0	0.0	21.0	0.5	0.0	21.3
2006	0.5	0.0	0.0	0.0	20.7	0.5	0.0	21.5
2007	0.5	0.0	0.0	0.0	22.3	0.5	0.0	22.8
2008	0.0	0.0	0.0	0.0	24.3	0.0	0.0	24.3
2009	0.0	0.0	0.0	0.0	24.1	0.0	0.0	24.3
2010	0.4	0.0	0.0	0.0	26.2	0.4	0.0	26.9
2011	0.4	0.0	0.0	0.0	25.6	0.4	0.0	26.3
2012	0.4	0.0	0.0	0.0	28.3	0.4	0.0	29.1
2013	0.5	0.0	0.0	0.0	32.8	0.5	0.0	33.6
2014	0.7	0.0	0.0	0.0	35.9	0.7	0.0	36.9
2015	0.7	0.0	0.3	0.1	41.1	1.0	0.3	42.1
2016	1.2	0.0	0.3	0.1	41.8	1.7	0.4	43.4
2017	1.1	0.0	0.3	0.1	37.2	1.6	0.5	38.8
2018	1.0	0.0	0.4	0.1	42.3	1.4	0.4	43.7
2019	1.0	0.0	0.0	0.1	42.1	1.1	0.1	43.1

Table 22: Different sources of electricity generation in Liaoning (representing Northeast China) (TWh)

Year	Hydropower	Nuclear	Wind	Solar	Thermal power	Low carbon generation	Renewable generation	Total generation
2000	1.5	0.0	0.0	0.0	62.8	1.5	0.0	64.6
2001	2.3	0.0	0.0	0.0	63.9	2.3	0.0	66.2
2002	1.4	0.0	0.0	0.0	70.9	1.4	0.0	72.5
2003	2.3	0.0	0.0	0.0	81.2	2.3	0.0	83.7
2004	3.9	0.0	0.0	0.0	83.4	3.9	0.0	87.5
2005	5.7	0.0	0.0	0.0	84.5	5.7	0.0	90.4
2006	4.7	0.0	0.0	0.0	96.3	4.7	0.0	101.5
2007	4.4	0.0	0.0	0.0	106.5	4.4	0.0	111.5
2008	3.9	0.0	0.0	0.0	108.5	3.9	0.0	113.8
2009	2.9	0.0	0.0	0.0	111.7	2.9	0.0	116.3
2010	4.4	0.0	0.0	0.0	120.4	4.4	0.0	129.5
2011	3.2	0.0	0.0	0.0	126.0	3.2	0.0	137.0
2012	3.8	0.0	0.0	0.0	130.4	3.8	0.0	144.1
2013	6.1	0.0	0.0	0.0	133.4	6.1	0.0	155.4
2014	4.2	0.0	0.0	0.0	137.0	4.2	0.0	165.6
2015	3.2	14.5	11.2	0.1	135.8	29.0	25.8	166.5
2016	4.7	20.0	12.9	0.3	140.0	37.9	33.2	177.9
2017	3.5	23.6	14.4	0.6	140.9	42.0	38.6	182.9
2018	2.8	28.4	16.5	1.0	141.1	48.7	45.9	189.8
2019	2.8	32.7	15.3	1.4	147.4	52.2	49.4	199.6

Table 23: Different sources of electricity generation in Jiangsu (representing East China) (TWh)

Year	Hydropower	Nuclear	Wind	Solar	Thermal power	Low carbon generation	Renewable generation	Total generation

2000	0.0	0.0	0.0	0.0	91.0	0.0	0.0	91.0
2001	0.0	0.0	0.0	0.0	98.6	0.0	0.0	98.7
2002	0.1	0.0	0.0	0.0	111.6	0.1	0.0	111.7
2003	0.4	0.0	0.0	0.0	133.3	0.4	0.0	133.7
2004	0.3	0.0	0.0	0.0	155.1	0.3	0.0	155.5
2005	0.3	0.0	0.0	0.0	211.4	0.3	0.0	212.0
2006	0.3	0.0	0.0	0.0	251.3	0.3	0.0	253.6
2007	0.3	0.0	0.0	0.0	257.7	0.3	0.0	267.5
2008	0.7	0.0	0.0	0.0	263.1	0.7	0.0	281.5
2009	0.2	0.0	0.0	0.0	276.2	0.2	0.0	292.8
2010	0.3	0.0	0.0	0.0	316.6	0.3	0.0	335.9
2011	0.2	0.0	0.0	0.0	356.3	0.2	0.0	376.3
2012	1.1	0.0	0.0	0.0	377.9	1.1	0.0	400.1
2013	1.1	0.0	0.0	0.0	409.9	1.1	0.0	432.1
2014	1.2	0.0	0.0	0.0	409.4	1.2	0.0	434.6
2015	1.2	16.6	5.9	1.9	410.4	25.6	24.5	436.1
2016	1.7	15.4	9.4	4.1	440.3	30.6	28.9	470.9
2017	2.9	17.3	11.7	6.2	453.0	38.0	35.1	491.5
2018	3.3	24.2	17.3	4.6	447.7	49.4	46.1	493.4
2019	3.3	32.9	15.9	5.9	443.9	57.9	54.7	501.5

Table 24: Different sources of electricity generation in Guangdong (representing South Central China) (TWh)

Year	Hydropower	Nuclear	Wind	Solar	Thermal power	Low carbon generation	Renewable generation	Total generation
2000	10.6	0.0	0.0	0.0	103.9	10.6	0.0	129.3
2001	19.0	0.0	0.0	0.0	107.5	19.0	0.0	141.8
2002	10.9	0.0	0.0	0.0	121.0	10.9	0.0	152.6
2003	18.0	0.0	0.0	0.0	139.9	18.0	0.0	188.3
2004	19.2	0.0	0.0	0.0	166.1	19.2	0.0	214.1
2005	20.8	0.0	0.0	0.0	176.5	20.8	0.0	227.9
2006	26.8	0.0	0.0	0.0	188.4	26.8	0.0	246.6
2007	24.1	0.0	0.0	0.0	218.7	24.1	0.0	273.2
2008	38.8	0.0	0.0	0.0	196.9	38.8	0.0	271.6
2009	26.9	0.0	0.0	0.0	215.8	26.9	0.0	275.8
2010	34.9	0.0	0.0	0.0	248.8	34.9	0.0	323.7
2011	33.1	0.0	0.0	0.0	301.8	33.1	0.0	380.2
2012	36.7	0.0	0.0	0.0	288.1	36.7	0.0	376.4
2013	38.9	0.0	0.0	0.0	297.3	38.9	0.0	387.5
2014	40.7	0.0	0.0	0.0	301.9	40.7	0.0	401.3
2015	43.7	60.6	5.5	0.2	293.4	110.0	66.4	403.5
2016	44.3	70.3	4.7	0.4	297.2	119.9	75.5	417.0
2017	30.8	80.0	5.5	1.1	332.9	117.3	86.5	450.3
2018	14.7	89.2	6.3	0.8	326.0	111.0	96.3	437.0
2019	18.5	110.2	6.9	2.5	334.6	138.0	119.5	472.6

Table 25: Different sources of electricity generation in Sichuan (representing Southwest China) (TWh)

Year	Hydropower	Nuclear	Wind	Solar	Thermal power	Low carbon generation	Renewable generation	Total generation
2000	31.5	0.0	0.0	0.0	18.5	31.5	0.0	50.0

2001	42.2	0.0	0.0	0.0	20.9	42.2	0.0	63.3
2002	41.0	0.0	0.0	0.0	28.6	41.0	0.0	69.6
2003	48.0	0.0	0.0	0.0	36.8	48.0	0.0	84.9
2004	62.1	0.0	0.0	0.0	31.4	62.1	0.0	93.4
2005	65.3	0.0	0.0	0.0	36.5	65.3	0.0	101.9
2006	78.5	0.0	0.0	0.0	44.2	78.5	0.0	122.7
2007	81.4	0.0	0.0	0.0	44.9	81.4	0.0	126.3
2008	98.1	0.0	0.0	0.0	40.1	98.1	0.0	138.3
2009	106.5	0.0	0.0	0.0	51.3	106.5	0.0	157.9
2010	121.3	0.0	0.0	0.0	57.0	121.3	0.0	179.5
2011	136.4	0.0	0.0	0.0	60.9	136.4	0.0	198.1
2012	156.2	0.0	0.0	0.0	58.8	156.2	0.0	215.1
2013	200.2	0.0	0.0	0.0	62.8	200.2	0.0	263.1
2014	250.1	0.0	0.0	0.0	59.0	250.1	0.0	309.5
2015	266.8	0.0	1.0	0.1	45.0	267.9	1.1	313.0
2016	285.2	0.0	1.8	0.6	39.8	287.6	2.4	327.4
2017	304.1	0.0	3.8	1.7	38.4	309.6	5.5	348.0
2018	298.2	0.0	5.5	1.4	44.8	305.1	6.9	349.9
2019	307.6	0.0	7.3	2.0	50.3	316.8	9.3	367.1

Table 26: Different sources of electricity generation in Shaanxi (representing Northwest China) (TWh)

Year	Hydropower	Nuclear	Wind	Solar	Thermal power	Low carbon generation	Renewable generation	Total generation
2000	3.5	0.0	0.0	0.0	23.7	3.5	0.0	27.2
2001	14.9	0.0	0.0	0.0	27.5	14.9	0.0	42.4
2002	2.6	0.0	0.0	0.0	31.8	2.6	0.0	34.4
2003	4.7	0.0	0.0	0.0	37.3	4.7	0.0	41.9
2004	4.2	0.0	0.0	0.0	45.6	4.2	0.0	49.8
2005	5.1	0.0	0.0	0.0	49.6	5.1	0.0	54.9
2006	4.0	0.0	0.0	0.0	54.5	4.0	0.0	58.5
2007	5.5	0.0	0.0	0.0	65.1	5.5	0.0	70.7
2008	6.4	0.0	0.0	0.0	78.7	6.4	0.0	85.3
2009	7.5	0.0	0.0	0.0	83.4	7.5	0.0	90.9
2010	8.7	0.0	0.0	0.0	102.5	8.7	0.0	111.2
2011	10.0	0.0	0.0	0.0	112.2	10.0	0.0	122.2
2012	8.9	0.0	0.0	0.0	125.2	8.9	0.0	134.2
2013	11.1	0.0	0.0	0.0	139.2	11.1	0.0	151.2
2014	11.7	0.0	0.0	0.0	149.1	11.7	0.0	163.0
2015	13.4	0.0	2.8	0.8	145.2	17.0	3.6	162.3
2016	12.5	0.0	3.7	1.3	158.1	17.6	5.1	175.7
2017	14.2	0.0	5.1	3.4	158.6	22.7	8.5	181.4
2018	11.5	0.0	7.2	4.0	156.7	22.7	11.2	178.2
2019	13.7	0.0	7.1	5.2	185.9	26.0	12.2	211.9

Indicator 3.2: Clean household energy

Methods

This indicator is modelled with household investigation data collected by Building Energy Conservation Research Center, Tsinghua University and per capita household energy consumption data compiled by National Bureau of Statistics.
The definition of clean energy differs from the global Lancet Countdown. Here, nuclear and renewable energy (solar energy, hydroenergy, wind energy, biomass energy, etc.) are defined as clean energy in China and also electricity is not tracked.
The energy consumption data of the different types of energy consumption were converted through the average energy efficiency in 2015.
Since energy structure adjustment is related to infrastructure, fossil fuel utilization status such as urban natural gas penetration rate was investigated. Meanwhile, renewable energy sources including solar energy and geothermal energy are mostly used in domestic hot water, so that the equipment was investigated as well.
The data provided in the 2020 report focuses on household energy consumption and energy structure, particularly cooking and domestic hot water.

Data

1. The per capita household energy consumption data is taken from the National Bureau of Statistics.
2. The data on household cooking energy sources and domestic hot water equipment is taken from the Building Energy Conservation Research Center, Tsinghua University.
3. The data on rural households is taken from CRECS 2013.[62]

Caveats

The caveats of this indicator would mainly be in three aspects. First, the sample size of cooking and domestic hot water investigation is limited to urban households in 2015 while data on rural households are from an investigation in 2013. More recent and adequate data may reveal different results, as more policies have been introduced since China joined the Paris Climate Agreement in 2015 to promote clean energy. Annually data collection should be organized or initiated by the government.Second, the impact of markets and policies on the promotion of clean energy was not included.

Additional Information

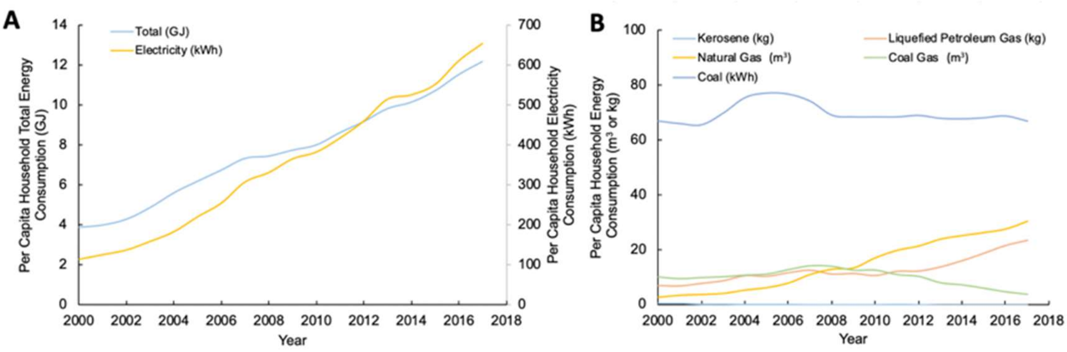

Figure 28: **Household Energy Consumption in China. (A) Household energy consumption per capita from 2000 to 2017, total (left axis) vs electricity (right axis). (B) Per capita household energy consumption by fuel type from 2000 to 2017.**

Indicator 3.3: Air pollution, energy, and transport

Indicator 3.3.1: Exposure to air pollution in cities

Methods

This indicator reports the trends of annual air pollutant concentrations in China's cities based on monitoring data of air pollutants. The distribution of cities' annual average $PM_{2.5}$ ae well as the developing trends are analyzed with statistical description method (Minimum, Lower quartile, Median, Upper quartile and Maximum) based on the monitoring data involve 367 cities during 2015-2019 in China.
Population of the Beijing-Tianjin-Hebei region (so-called "2+26" cities), Fenhe and Weihe plain, and Northwest China exposed to the most serious $PM_{2.5}$ pollution. "2+26" cities are considered an important atmospheric pollution transit corridor which connects Jing-Jin-Ji district with the surrounding area while Fenhe and Weihe plain is another corridor linking Fenhe plain, Weihe plain and surrounding cities.

Data

Data of daily 24-hour average $PM_{2.5}$ concentrations in cities are downloaded from the Data Center of Ministry of Ecology and Environment of China. According to 'Technical Regulation for Ambient Air Quality Assessment' (HJ 633-2013) published by the Ministry of Environmental Protection of China, the city-specific annual average $PM_{2.5}$ concentration is calculated by arithmetic mean of daily 24-hour average $PM_{2.5}$ concentrations. [77]

Caveats

The indicator relies on the accuracy and timeliness of the air pollution monitoring.

Future Form of Indicator

The combination of monitoring data, atmospheric transport and chemistry model (e.g., GEOS-Chem), and air pollutant emission projection model (e.g., GAINS) has a practical urgency for pollutant exposure analysis of higher resolution[78,79].

Additional Information

Benefiting from the toughest ever clean air action plan launched in 2013 and subsequent effective measures (such as strengthening industrial emission standards, upgrades on industrial boilers, phasing out outdated industrial capacities and promoting clean fuels in the residential sector), remarkable air quality improvements have been made in China[80].

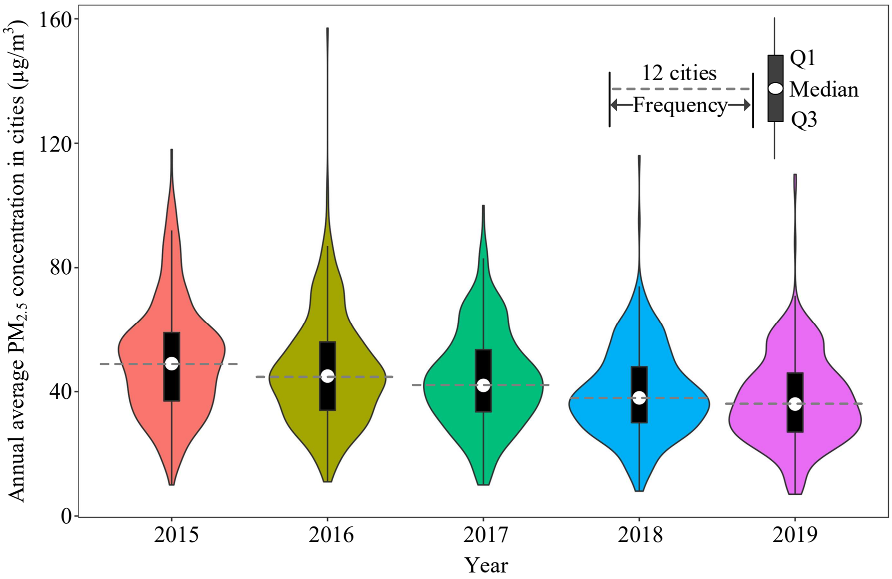

Figure 29: **Distribution of annual average $PM_{2.5}$ concentrations of China's 367 cities. The white dot represents the median Q2; The black rectangle is the range from the lower quartile Q1 to the upper quartile Q3; The external shape of the black rectangle is kernel density estimation, of which the vertical length represents dispersion degree and the horizontal width denotes city frequency in a certain pollutant concentration.**

Table 27: Statistics for annual average $PM_{2.5}$ concentrations of China's cities (Unit: $\mu g/m^3$).

Year	Minimum	Lower quartile	Median	Upper quartile	Maximum
2015	10	37	49	59	118
2016	11	34	45	56	157
2017	10	34	42	53	100
2018	8	30	38	48	116
2019	7	27	36	46	110

Table 28: List of "2+26" cities and the Fenhe and Weihe Plain.

**"2+26" cities**		**Fenhe and Weihe Plain**	
Province/Municipality	City	Province	City
Beijing	Beijing	Shanxi	Jinzhong
Tianjin	Tianjin		Yuncheng
Hebei	Shijiazhuang		Linfen
	Baoding		Lvliang
	Langfang	Henan	Luoyang
	Cangzhou		Sanmenxia
	Hengshui	Shaanxi	Xian
	Xingtai		Tongchuan
	Tangshan		Baoji
	Handan		Xianyang
Shanxi	Taiyuan		Weinan
	Yangquan		Yangling
	Changzhi		
	Jincheng		
Shandong	Jinan		
	Zibo		
	Jining		
	Dezhou		

	Binzhou	
	Liaocheng	
	Heze	
Henan	Zhengzhou	
	Kaifeng	
	Anyang	
	Hebi	
	Xinxiang	
	Jiaozuo	
	Puyang	

Indicator 3.3.2: Premature mortality from ambient air pollution by sector

Methods

This indicator quantifies the number of premature deaths attributable to long-term ambient fine particulate matter ($PM_{2\cdot5}$) exposure by sectorial sources for each province in China. The greenhouse gas-air pollution interactions and synergies (GAINS) model is used to quantify the sectorial contribution to ambient $PM_{2\cdot5}$.[81] Data from the International Energy Agency (IEA) World Energy Outlook 2019 and the data of Chinese statistical yearbook in 2018 is integrated into GAINS to develop the provincial air pollution emission inventory by fuels and sectors.

Atmospheric chemistry and dispersion coefficients with the European Monitoring and Evaluation Programme (EMEP) Chemistry Transport Model are used to simulate the changes in ambient $PM_{2\cdot5}$ with varying emissions.[82] Validation of the performance of the ambient annual $PM_{2\cdot5}$ concentration of the GAINS against results from the observed are presented in ***Figure 30***. Premature deaths from total ambient $PM_{2\cdot5}$ by provinces and sectors in China are calculated using the integrated exposure-response functions (IERs) employed by the WHO (2016) assessment on the disease burden from long-term exposure to ambient air pollution,[83] which relies on cause-specific mortality relative risk (RR) functions and requires the application to a higher range of annual average concentrations in the study area.[84]
The concentration-response (C-R) functions and relative risks [Eq. (1)] were based on the IERs from the GBD 2013,[83] across the full range of $PM_{2\cdot5}$ concentrations. $RR_{IER}(z)$ represents the relative risks in the $PM_{2\cdot5}$ exposure concentration of C (in micrograms per meter cubed); C_0 represents the counterfactual concentration below which it is assumed there is no additional risk. For very large C, $RR_{IER}(z)$ approximates $1+\alpha$. A power of $PM_{2\cdot5}$, δ, was included here to predict risk over a very large range of concentrations.

$$RR_{IER}(Z)=\begin{cases} 1, \text{ for } C < C_0 \\ 1+\alpha\{1-\exp[-\gamma(C-C_0)^{\delta}]\}, \text{ for } C \geq C_0 \end{cases} \quad (1)$$

We adopted a calculation approach [Eq. (2)] developed for the GBD 2013 to estimate $PM_{2.5}$-related premature mortality in each province, and the following five endpoints are included in our estimation: ischemic heart disease (IHD), chronic obstructive pulmonary disease (COPD), lung cancer (LC), and stroke in adults, and acute lower respiratory infections (ALRI) in children less than 5 years old. For IHD and stroke, the RR is different between age strata, and for COPD and LC, the RR in the same exposure concentration is the same for the entire group of adults (aged 25 or more). We estimated the premature mortality $M_{i,j}$ of each province (and of each age stratum for IHD and stroke) and disease endpoint j attributable to ambient $PM_{2\cdot5}$ for Province i.

$$M_{i,j}=P_i \times \hat{I}_j \times (RR_j(C_i)-1), \text{ where } \hat{I}_j=\frac{I_j}{RR_j} \quad (2)$$

$\hat{I}_j$ represents the hypothetical "underlying incidence" (i.e., cause-specific mortality rate) that would remain if $PM_{2\cdot5}$ concentrations were reduced to the theoretical minimum risk concentration. Here, P_i is the population of province i, I_j is the reported regional average annual disease incidence (mortality) rate for endpoint j, C_i represents the annual-average $PM_{2\cdot5}$ concentration in county i, RR_j (C_i) is the relative risk for end point j at

concentration C_i, and RR_j represents the average population-weighted relative risk for end point j.

Data

1. Emissions data was taken from the IEA World Energy Outlook 2019 and the Chinese statistical yearbook in 2018
2. Provincial air pollution emission inventory by fuels and sectors was from GAINS model;
3. Provincial demographic and mortality data was from Chinese statistical yearbook in 2015;
4. Baseline mortality data was obtained from Zhou et al.[85] and the results of GBD 2013 studies;[83]
5. The RR value and estimated parameters were from GBD 2013.[83]

Caveats

There are three key caveats of this indicator. Firstly, the indicator relies on model calculations which are currently available for a limited set of regions (Europe, South Asia, East Asia). Uncertainty in the shape of integrated exposure-response relationships in different provinces make the quantification of health burden inherently uncertain. Secondly, estimated annual mean $PM_{2.5}$ concentration for each province was calculated from GAINS model, the health effects related to air pollution are calculated based on provincial concentration rather than grid data, this part will be improved in the future. Thirdly, $PM_{2.5}$ from various sources used the same C-R function and RR, so the estimated results may deviate from the actual situation to some extent.

Additional information

The three provinces with the highest number of $PM_{2.5}$-related deaths are Shandong, Henan, and Hebei Province. Compared to 2015, the number of premature deaths in 2018 dropped by roughly 17,000, 15,000, and 11,000 for these three provinces, respectively. The contribution from households dropped sharply between 2015 and 2018 due to restrictions on raw coal consumption, although households continue to account for 18% of total premature deaths in North China, where residential heating is required. Households and transport play a key role in megacities (i.e. Beijing and Chongqing), while industry and power have a dominant contribution in less developed provinces, for example Anhui, Hebei, Henan, Shandong, and Sichuan provinces.

Table 29: Premature deaths due to long-exposure to $PM_{2.5}$ from different sectors in each province in 2015 (Unit: person).

Province	Power plants	Industry	Transport	Households	Waste	Agriculture	Other sectors	Natural	Total
**Anhui**	4293	11340	4380	6763	3206	15946	2986	2174	51087
**Beijing**	731	2851	1345	3650	1351	3400	1730	1180	16240
**Chongqing**	1243	4631	3196	7689	1244	5768	1092	261	25124
**Fujian**	1288	2796	2503	1455	1369	3765	961	1507	15644
**Gansu**	589	1394	503	1912	638	1471	284	4123	10913
**Guangdong**	3830	8229	7451	4112	5464	9296	2991	2882	44255
**Guangxi**	1748	5636	2627	3050	1562	4549	801	3254	23226
**Guizhou**	1382	3239	1181	5311	1005	3410	431	2558	18517
**Hainan**	186	395	356	200	164	334	99	477	2211
**Hebei**	4408	19003	4224	12944	4261	18501	6424	5846	75612
**Heilongjiang**	1577	3322	2716	4522	1587	4692	918	2232	21566
**Henan**	7357	22243	6438	12681	5963	28194	5432	2814	91121

**Hubei**	3093	11136	3831	7934	2851	12091	1991	1082	44009
**Hunan**	2918	11271	3876	7496	2803	10914	1721	2287	43285
**Inner Mongolia**	822	1476	954	5233	605	1516	352	5484	16443
**Jiangsu**	5054	13052	6808	5341	3263	15827	4376	2971	56692
**Jiangxi**	1712	5173	1960	2244	1462	5716	1011	1297	20574
**Jilin**	1413	3388	1773	4135	1341	4450	726	1069	18295
**Liaoning**	3224	10289	4476	7123	2801	9738	2229	2596	42476
**Ningxia**	225	329	111	293	107	295	50	1367	2778
**Qinghai**	98	295	197	417	175	260	66	625	2131
**Shaanxi**	1769	4165	1960	6048	2402	5767	1269	2664	26045
**Shandong**	8430	22821	8461	12400	4443	29039	5693	3608	94895
**Shanghai**	1581	3721	1946	861	1018	3067	1144	0	13338
**Shanxi**	1897	5559	1209	5068	1355	5573	1069	3132	24863
**Sichuan**	3375	14144	6759	14190	4798	15698	2613	1070	62647
**Tianjin**	879	2599	1291	1752	558	2824	1004	727	11634
**Tibet**	0	1	1	2	0	1	0	9	14
**Xinjiang**	533	1114	1732	2530	195	1505	598	5908	14117
**Yunnan**	517	2085	975	1813	224	1962	222	4153	11951
**Zhejiang**	2627	4859	3344	1704	1016	6746	1989	1558	23843

Table 30: Premature deaths due to long-exposure to $PM_{2.5}$ from different sectors in each province in 2018 (Unit: person).

Province	Power plants	Industry	Transport	Households	Waste	Agriculture	Other sectors	Natural	Province total
Anhui	3806	10650	4295	1673	2592	17366	3854	2589	46825
Beijing	695	2626	1212	940	750	3659	2291	1457	13631
Chongqing	1106	4785	3141	1590	1085	6360	1653	346	20065
Fujian	1003	2647	2418	435	803	3900	1212	1734	14152
Gansu	523	1350	472	965	403	1433	399	4787	10333
Guangdong	3257	8340	7015	2376	2670	9838	3901	3470	40867
Guangxi	1355	5723	2343	1178	919	4575	1029	3730	20852
Guizhou	1089	2992	1013	5287	652	3175	555	2838	17601
Hainan	149	376	325	85	91	316	117	509	1969
Hebei	4134	17808	3861	3565	3122	20173	8406	7078	68146
Heilongjiang	1347	2740	2054	5270	726	4298	1047	2308	19790
Henan	6644	19873	5906	3423	4669	30865	7089	3389	81858
Hubei	2606	10106	3603	3936	2089	13099	2591	1291	39321
Hunan	2140	10952	3600	5405	2043	11064	2165	2626	39996
Inner_Mongolia	940	1506	899	1758	238	1624	518	6983	14467
Jiangsu	4346	11830	6494	1250	2221	16677	5349	3367	51534
Jiangxi	1387	4791	1811	938	1054	5779	1283	1495	18538
Jilin	1319	3058	1512	2225	683	4583	914	1226	15519
Liaoning	3097	9354	3952	2852	1428	10183	2836	3067	36769
Ningxia	276	351	91	135	60	284	62	1516	2776
Qinghai	103	302	187	383	90	257	94	747	2163
Shaanxi	1749	4359	1848	2656	1557	6451	1821	3398	23839
Shandong	7810	20524	7621	3568	3486	31142	7215	4253	85619
Shanghai	1184	3277	1863	309	794	3046	1370	0	11842
Shanxi	1918	4758	1053	2909	959	5893	1406	3777	22673
Sichuan	2167	15336	6803	2517	3553	16695	3769	1359	52198
Tianjin	774	2569	1083	461	427	2935	1264	852	10365
Tibet	0	1	1	2	0	1	0	8	12
Xinjiang	984	1083	1309	3314	71	1309	736	6405	15211
Yunnan	290	1801	813	1151	161	1747	304	4367	10632
Zhejiang	1915	4664	3205	770	896	6816	2419	1758	22442

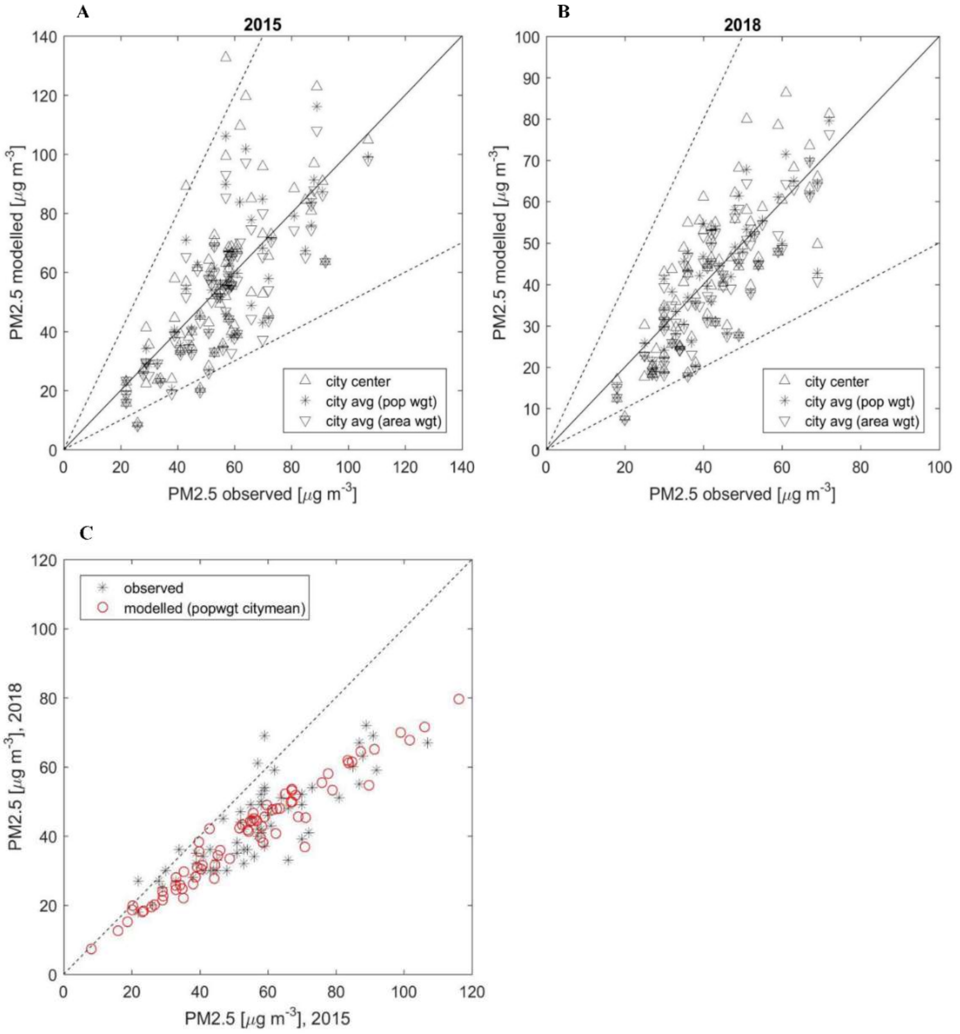

Figure 30: Comparison of concentration of ambient fine particulate matter ($PM_{2.5}$) of the GAINS and the results of observed in 2015-2018

Indicator 3.4: Sustainable and healthy transport

Methods:

This indicator shows the changes in emission intensity of road transport, as well as the average emission per vehicle, of 4 major pollutants (CO, HC, NOx, PM_{10}), from 2000 to 2018 for China, and total emission intensity of road transport for all provinces from 2010 to 2018, where data is available.

Emissions intensity is calculated through the ratio of vehicular emissions to vehicle ownership.

Data

1. Emission data is from China Vehicle Environmental Management Annual Reports (2009-2019)[86]
2. Vehicle ownership data is from National Bureau of Statistics of China (2000-2018)[87]

Caveats

There are several caveats for this first version of this indicator. Firstly, data used did not include low-speed vehicle or motorcycles, so it does not consider all emissions from vehicles. Secondly, this indicator does not currently measure greenhouse gas emissions intensity, so whilst it is an indicator of the health co-benefits of mitigation (through the reduction in road transport-related air pollutants), it does not currently measure mitigation directly. Thirdly, this indicator does not consider other health co-benefits of mitigation in the transport sector, such as the health benefits resulting from increased physical activity related to walking and cycling. Finally, provincial emission data is not available before 2010.

Future Form of Indicator

This indicator can be improved when reliable data of new energy vehicle ownership for each province becomes available, which can be used as supporting data for additional analysis. In the future, carbon intensity of vehicles will also be analysed for this indicator. Additionally, the Chinese government has recently released an Action Plan for Green Travel (2019-2022) in May, 2019. This will mean that data on the percentage of the population walking, cycling, and using public transport provincially and nationally should be soon available and will be introduced as a new indicator in future reports.

Additional Information

The emission of four major pollutants, hydrocarbon (HC), carbon monoxide (CO), nitrogen oxide (NO_x) and particulate matter (PM_{10}), threatens public health, especially when people are close to the mobile sources in densely populated areas of many provinces in China. China has made great effort for the past decades to lower mobile source pollution, including the implementation of increasingly stringent standards for vehicle emission and fuel quality, and the push for cleaner fuel through modal shift and new energy vehicles. [86] Also, new energy vehicle ownership reached 1.46% of the total vehicle ownership in China in 2019, which has increased rapidly from 220,000 in 2014 to 3,810,000 in 2019.[88]

Table 31: The emission intensity of road transport in China from 2000 to 2018 for CO, HC, NOx and PM_{10} (Unit: Tons of Emission/Vehicle)

Years	CO	HC	NOx	PM_{10}
2000	1.524635	0.16968	0.238049	0.03145
2001	1.434485	0.165368	0.228075	0.029189
2002	1.332086	0.152447	0.217225	0.026642
2003	1.163693	0.136806	0.188843	0.022661
2004	1.071385	0.123621	0.174109	0.020566
2005	0.925416	0.108556	0.152865	0.017597
2006	0.801114	0.093851	0.129823	0.015038
2007	0.683973	0.080076	0.113345	0.012872
2008	0.592006	0.069221	0.098243	0.010903
2009	0.486577	0.056205	0.084864	0.009012
2010	0.406905	0.046143	0.068804	0.007242
2011	0.298835	0.039065	0.061605	0.006306
2012	0.262094	0.031025	0.053315	0.005415
2013	0.22984	0.027245	0.046464	0.004475
2014	0.201581	0.023907	0.039656	0.003768
2015	0.184784	0.021603	0.033105	0.003291
2016	0.161431	0.019295	0.028781	0.002756
2017	0.139683	0.01698	0.025485	0.002334
2018	0.12308	0.01473	0.022465	0.001817

Table 32: The total emission intensity of road transport from 2010 to 2018 for provinces in China where data is available (Unit: Tons of Emission/Vehicle)

Provinces	2010	2011	2012	2013	2014	2015	2016	2017	2018

Anhui	0.517	0.392	0.325	0.274	0.252	0.225	0.190	0.198	0.174
Beijing	0.230	0.192	0.160	0.144	0.127	0.116	0.098	0.156	0.134
Chongqing	0.689	0.505	0.425	0.365	0.319	0.271	0.236	0.196	0.166
Fujian	0.405	0.298	0.248	0.196	0.172	0.145	0.126	0.184	0.152
Gansu	1.318	0.972	0.823	0.725	0.652	0.525	0.459	0.197	0.182
Guangdong	0.510	0.379	0.334	0.291	0.243	0.220	0.189	0.169	0.147
Guangxi	0.674	0.507	0.439	0.355	0.295	0.255	0.212	0.266	0.211
Guizhou	0.530	0.413	0.365	0.326	0.296	0.256	0.222	0.206	0.168
Hainan	0.487	0.386	0.432	0.320	0.275	0.222	0.155	0.232	0.160
Hebei	0.636	0.473	0.401	0.353	0.310	0.274	0.236	0.200	0.172
Heilongjiang	0.918	0.701	0.650	0.587	0.529	0.484	0.428	0.209	0.177
Henan	0.627	0.470	0.416	0.357	0.266	0.275	0.238	0.189	0.165
Hubei	0.582	0.438	0.385	0.339	0.288	0.244	0.199	0.198	0.158
Hunan	0.456	0.351	0.318	0.282	0.249	0.221	0.187	0.204	0.162
Inner Mongolia	0.782	0.580	0.537	0.483	0.448	0.424	0.378	0.185	0.166
Jiangsu	0.409	0.310	0.250	0.216	0.186	0.163	0.143	0.162	0.145
Jiangxi	0.799	0.593	0.484	0.396	0.347	0.294	0.250	0.211	0.174
Jilin	0.843	0.633	0.565	0.497	0.434	0.396	0.344	0.155	0.154
Liaoning	0.519	0.388	0.341	0.309	0.271	0.246	0.220	0.199	0.184
Ningxia	0.842	0.654	0.566	0.500	0.476	0.370	0.326	0.227	0.186
Qinghai	0.734	0.589	0.576	0.501	0.483	0.386	0.356	0.233	0.160
Shaanxi	0.618	0.466	0.414	0.341	0.299	0.268	0.243	0.183	0.156
Shandong	0.412	0.309	0.257	0.217	0.183	0.165	0.149	0.196	0.161
Shanghai	0.334	0.263	0.234	0.223	0.197	0.168	0.138	0.212	0.186
Shanxi	0.657	0.505	0.457	0.412	0.376	0.348	0.316	0.184	0.172
Sichuan	0.377	0.294	0.262	0.225	0.190	0.170	0.150	0.184	0.164
Tianjin	0.304	0.234	0.211	0.187	0.186	0.174	0.157	0.163	0.143
Tibet	1.831	1.336	1.327	1.199	1.086	0.842	0.742	0.393	0.234
Xinjiang	1.260	0.917	0.771	0.687	0.614	0.587	0.519	0.172	0.175
Yunnan	0.620	0.485	0.438	0.404	0.372	0.351	0.308	0.107	0.179
Zhejiang	0.255	0.192	0.167	0.149	0.127	0.115	0.099	0.137	0.126

Indicator 3.5: Food, agriculture, and health

Methods

At the national level, emissions from livestock and crop production in China (area code 351) were obtained from the FAOSTAT from 2000-2017. At provincial level, emissions of 30 provinces in China mainland in 2017 were calculated, taking data from the China Statistical Yearbook. For livestock, methane emissions from enteric fermentation and manure management are calculated by multiplying livestock numbers and emission factors per head, nitrous oxide emissions from manure management and manure left on pasture are calculated by multiplying manure excreta and emission factors per kilogram of manure nitrogen. The following livestock are included: ruminant including buffaloes, camels, cattle (dairy), cattle (non-dairy), goats, and sheep, and non-ruminant including chicken (broilers), chicken (layers), ducks, swine (market), swine (breeding), asses, horses, mules, and turkeys.

For crops, methane emission from rice cultivation are calculated by multiplying rice area and emission factors per hectare. Nitrous oxide emissions from fertilizer (synthetic fertilizer and manure) and crop residues applied to soil are calculated by multiplying the nitrogen content of fertilizer or crop residue returning to field and emission factors per kilogram of nitrogen. Emissions from crop residue burning are calculated by multiplying the dry biomass of crop residue for burning and emissions factors per kilogram of dry biomass.

Data

1. At provincial level, crop production, sown area of rice and synthetic fertilizer use for crop emission calculation, and livestock number for livestock emission calculation were obtained from China Statistical Yearbook 2018.
2. Emission factors for crop residue, enteric fermentation, manure management, and manure left on pasture were obtained FAOSTAT (calculated data).
3. Crop residue biomass was calculated by multiplying grain production and a straw/grain ratio obtained from Gu et al. (2015).[89]
4. Emission factors for straw burning were derived from Zhang et al. (2017)[90] and Zhang et al. (2008)[91]. Animal excreta were calculated according to the Technical Guidelines for Compiling the Inventory of Atmospheric Ammonia Emission.[92]
5. The percentage of manure applied to soil was derived from Ma et al. (2012).[93]

Caveats

The sum of provincial emissions calculated differ from national emission obtained from FAOSTAT, because some parameters are missing in FAOSTAT for the calculation of provincial emissions, or China specific data derived from literature. The sum of provincial emissions from livestock was 12% lower than livestock emissions for China obtained from FAOSTAT, mainly due to a relatively underestimation of emissions from manure left on pasture. In calculating manure nitrogen left on pastures by grazing livestock, China's five main pastoral areas: Inner Mongolia, Gansu, Qinghai, Tibet, and Xinjiang were classified as grazing systems according to Bai et al. (2013),[94] while other provinces were assumed to have no grazing systems due to data availability. This classification leads to a relatively underestimation of livestock manure left on pasture. The sum of provincial emissions from crop production was 16% higher than crop emissions for China obtained from FAOSTAT, mainly due to a relatively overestimation of emissions from burning of crop residues. In calculating emissions from burning of crop residues, we used emission factors derived from Zhang et al. (2008),[91] who simulated the open burning of crop residues in China by a custom-designed combustion and test device. The emission of CO_2e per kilogram dry matter of burned crop residues was 17-28 times higher than that from FAOSTAT calculated data using IPCC Tier 1 method.

Findings

Overall CO_2e emissions from Chinese livestock have decreased by 7% since 2000 to approximately 0.29 Gt in 2017 (***Figure 31***). Ruminants contribute to 77% of total livestock emissions (0.22 $GtCO_2e$ per year). This is split between non-dairy cattle (34-46%), followed by goats and sheep (17-20%), buffalo (12-13%) and dairy cattle (3-8%). Emissions from non-ruminants are divided between pigs (13-16%), poultry (5-7%) and others (2-4%). A decrease in stock of non-dairy cattle by 28% is the main reason for livestock emission reduction from 2000 to 2017, while the stock of dairy cattle, goats and sheep, and poultry increased by 147%, 8%, and 35% respectively (***Figure 32)***.

Emissions of CO_2e from crop production have increased by 21% since 2000, to around 0.39 Gt in 2017 (***Figure 31***). The majority of the increase in emissions is attributed to synthetic fertilizer (45-52% of total crop emmissions in 2017) and crop residues (8-10%) which contributed 74% and 16% to the total increase in crop emissions over this period. The other activites contributing to crop emissions include

rice cultivation (29-35%), manure applied to soil (9-10%) and crop residues burning (1%).
China is the largest consumer of synthetic fertilizer worldwide. Synthetic fertilizer has played an indispensable role in ensuring food security in China during the past three decades, but also is a major source of pollution for freshwater and coastal ecosystems and greenhouse gas emissions.[95,96] From 2000 to 2017, China's chemical fertilizer use increased by 41%. However, in 2015, China made a plan of Zero Growth in Chemical Fertilizer Use by 2020[97] and since 2015, China's chemical fertilizer use has begun to decline (***Figure 33)***.

At the provincial level, for livestock, Inner Mongolia, Sichuan, Yunnan and Xinjiang have the largest emissions (31% national wide) due to large numbers of ruminants (***Figure 34***). For crop production, Henan has the largest emission due to the consumption of synthetic fertilizer, followed by Heilongjiang, Hunan, and Anhui that have large area of rice cultivation. The four provinces together contribute to 27% of total national emissions from crop production.

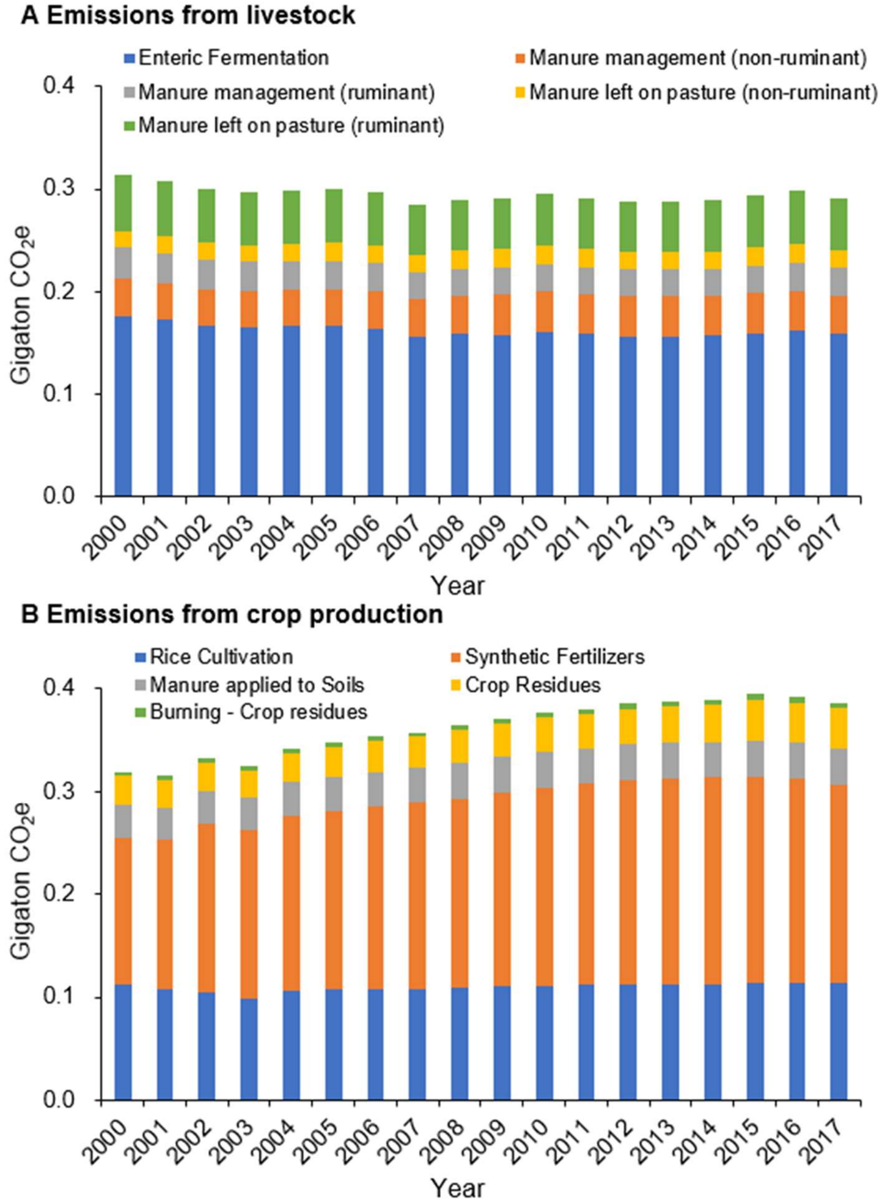

Figure 31: Gigaton CO_2e emissions from 2000 to 2017. (A) CO_2e emissions from livestock. (B) CO_2e emissions from crop production. CO_2e=carbon dioxide equivalent.

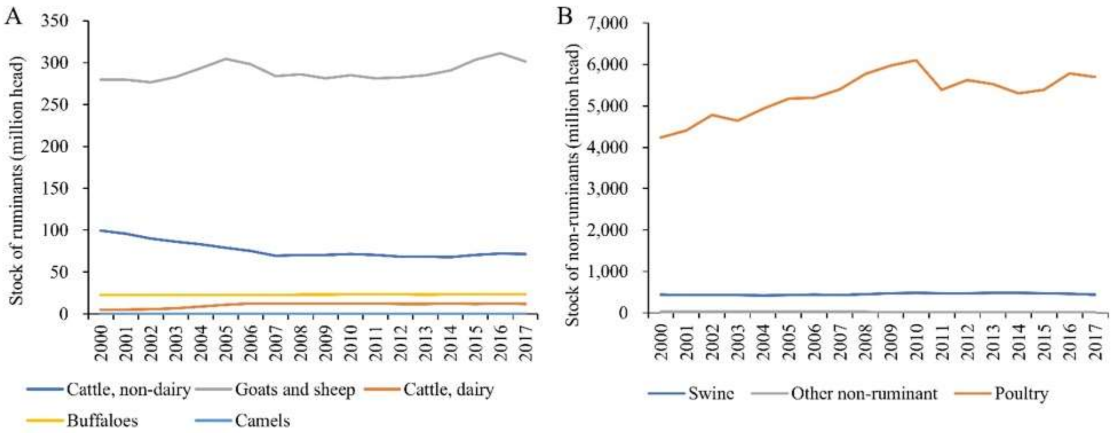

Figure 32: China's livestock number during 2000 to 2017
(A) Stock of ruminant animals. (B) Stock of non-ruminant animals.

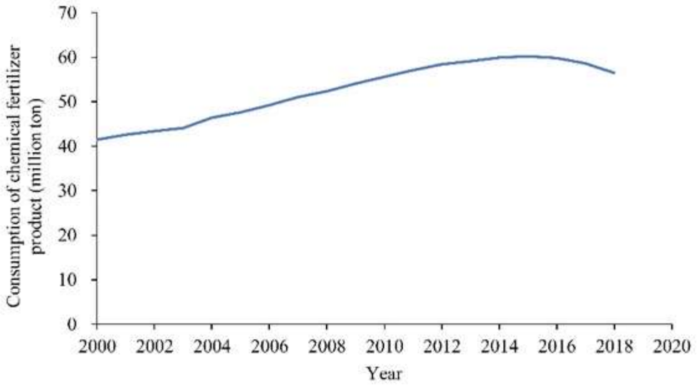

Figure 33: China's consumption of chemical fertilizer products during 2000 to 2018

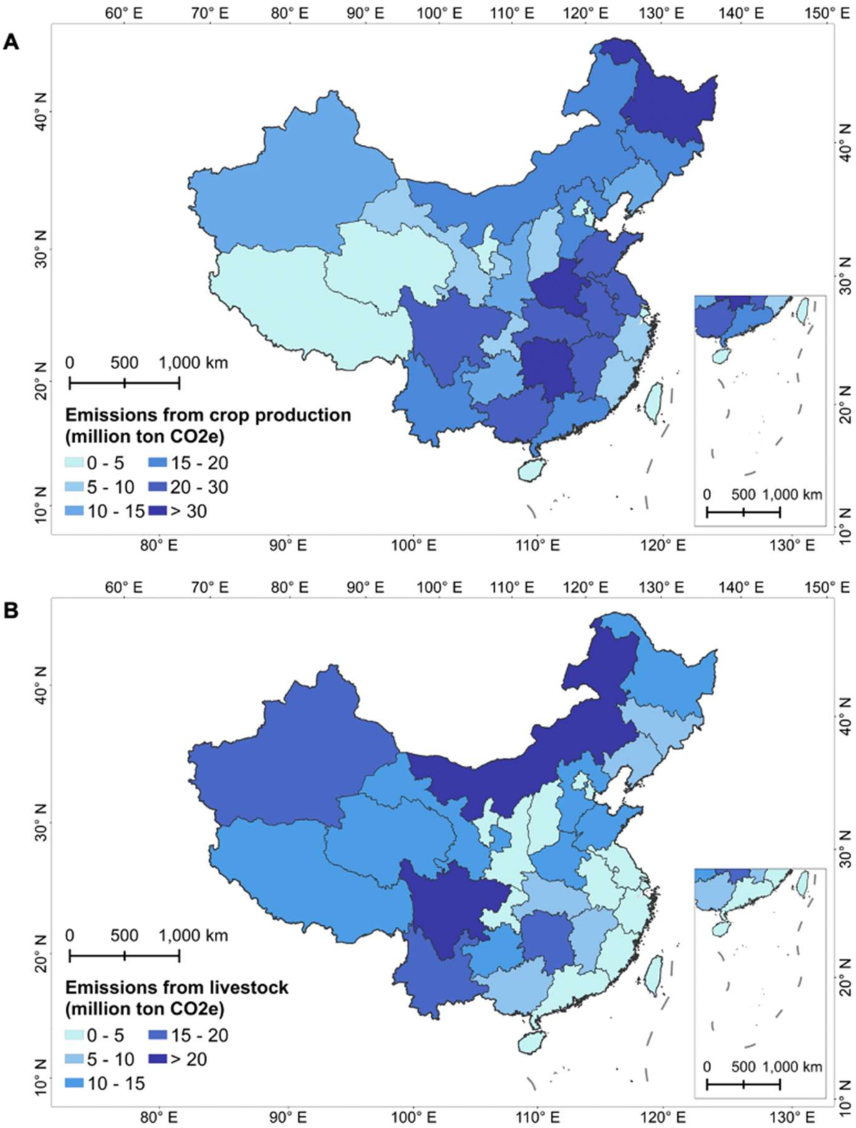

Figure 34: Provincial CO_2e emissions in 2017. (A) CO_2e emissions from livestock. (B) CO_2e emissions from crop production. CO_2e=carbon dioxide equivalent.

Section 4: Economics and finance

Indicator 4.1: Health and Economic Costs of Climate Change and Benefits from Mitigation

Indicator 4.1.1: Costs of heat-related mortality

Methods

This indicator is based on the value of statistical life (VSL) to monetize the heatwave-related mortality, assuming a fixed VSL for each area in China across time. 31 provinces in China were included in the estimation.
Heatwave-related mortality data is provided by WG1.

This indicator assumes a fixed VSL for each area in China across time. The mean value of VSLs in constant 2007 price (about 1.62 million RMB)[98] estimated in many studies. This was translated to the value in constant 2015 US$ price. The monetized value of mortality is equivalent to mortality multiped by the fixed VSL. The total values are also calculated as a proportion of GDP for China and for each province.

Data

1. Heatwave-related mortality data as described in indicator 1.1.2;
2. GRP, GRP index, GDP, GDP index and exchange rate data from National Bureau of Statistics of China.[99]

Caveats

The caveats of this indicator would mainly be in two aspects.Firstly, the VSL method has the disadvantage of relying on the estimates of what people say they would be willing pay to reduce mortality risks, collected in surveys. The results of studies highly depend on the survey design and characteristics of populations questioned, leading to doubts on the gap between surveyed results and actual VSL.Secondly, different areas have different VSLs, therefore using a fixed VSL in all regions may lead to some uncertainty.

Future Form of Indicator

In the future, this indicator would explore other methods to estimate economic costs of heatwave-related mortality, considering methods to estimate VSLs from wage-risk studies which measured revealed rather than hypothetical willingness to pay; or try to include value of Years of life lost (YLL) into consideration so that it could capture the age distribution of lives lost from heat stress.

Indicator 4.1.2: Economic cost of heat-related labour productivity loss

Methods

This indicator measures the total economic costs on industrial output resulting from potential heat-related labour productivity losses reported in indicator 1.1.3. It sees how the heat-related labour productivity loss in each industry influences the output of other industries through inter-industrial dependencies. Its calculation is based on the Ghosh model under the Input-Output (IO) analytical framework (see Xia, Li [100] for a full description of the model). The approach considers loss in productive hours as an indicator that brings about reduction in industrial value added, which in turn affects the output of all sectors adversely through the production supply chains. Therefore, it distinguishes between direct and indirect economic costs due to heat-related labour productivity loss. The direct cost results from the initial decrease in value added, and the indirect cost comes from inter-industrial dependencies. The analysis is also extended to a multi-regional scale to incorporate the spill-over effect of indirect economic cost through the inter-provincial trade links.

The main procedures are as below:

1. The calculations are first performed on the national scale using the Chinese IO tables available for five years between 2007-2017 (2007, 2010, 2012, 2015 and 2017), and then on the provincial scale using the Chinese Multiregional IO table in 2015. All the IO tables used in this analysis are converted from current LCU prices into constant US$ in 2015.
2. The reductions in industrial working hours are compared with the normal industrial working hours without heat-related labour productivity loss. This elicits the percentage losses of labourers' working hours caused by heat-related labour productivity loss each year, which constitutes a key input variable of the Ghosh model. It is assumed that there is no heat-related capital loss in this section.
3. The calculated percentage reductions in industrial working hours are expected to cause the same percentage reductions in industrial value added, as labour is a major component of the industrial value added.
4. The initial loss of a sector's value added, which constitutes the direct economic cost, will have knock-on effect that reduces other sectors' output through the production and supply network depicted by the

IO matrix, and the aggregate of output reductions of all sectors is counted as the total economic cost caused by heat-related labour productivity loss. The increase from the direct to the total cost indicates the indirect cost resulting from inter-industrial dependencies.

5. The industries are divided into primary, secondary and tertiary industries, following the same rules as Indicator 1.1.3.

Data

1. Data on heat-related labour productivity loss is provided by WG1 responsible for Indicator 1.1.3.
2. The Chinese IO tables between 2007 and 2017 are obtained from the website of the National Bureau of Statistics of China.[101]
3. The Chinese multi-regional IO table for 2015 is obtained from the CEADs dataset.[102]

Caveats

See Indicator 1.1.3, for caveats related to the calculation of heat-related labour productivity loss.
The current report employs a supply-driven IO model that fixes the input proportions between different kinds of productive factors. This means that producers do not seek for substitutive factor inputs when labourers become less productive due to heat-related labour productivity loss. The model also excludes the possibility of price adjustment, such as rising wages, to encourage labourer's production enthusiasm. Such rigidity decides that the model is better suitable for a short-term analysis. Therefore, the indirect economic cost is estimated during a single year with heat stress.

Due to data availability, the analysis is only performed at specific years with accessible IO tables. Because of different IO tables used, the sum of provincial costs due to heat-related labour productivity loss may be slightly different from the national costs.

Future Form of Indicator

In the future, this indicator will be developed to cover consecutive years with well-established Chinese IO tables both on the national and multi-provincial scales. Additionally, the industries will be disaggregated into more sub-industries when deeper investigations are conducted on industrial labourers.

Additional Information

Although the tertiary industry suffered much smaller direct costs from heat-related labour productivity loss than other industries, it still made up a considerable proportion of indirect costs (15.3% in 2015) due to inter-industrial dependencies.

Table 33: Chinese direct and indirect economic costs, in billions of US$ in 2015, from heat-related labour productivity loss by industry and year.

Years	Direct losses (billion US$)			Indirect losses (billion US$)		
	Primary industry	Secondary industry	Tertiary industry	Primary industry	Secondary industry	Tertiary industry
2007	5.70	6.35	0.32	1.64	15.82	2.84
2010	8.90	10.90	1.50	2.70	33.12	5.88
2012	7.73	7.26	0.14	2.25	22.82	4.18
2015	6.68	6.84	0.11	2.17	26.46	5.17
2017	13.03	25.58	2.78	4.31	65.38	15.23

Table 34: Chinese economic costs, in terms of shares in regional GDP, from heat-related labour productivity loss by province in 2015.

Provinces	Direct costs (% of regional GDP)	Indirect costs (% of regional GDP)
Beijing	0.01%	0.15%
Tianjin	0.05%	0.16%
Hebei	0.07%	0.17%
Shanxi	0.00%	0.14%
Inner Mongolia	0.00%	0.11%
Liaoning	0.00%	0.12%
Jilin	0.00%	0.07%
Heilongjiang	0.00%	0.09%
Shanghai	0.38%	0.23%
Jiangsu	0.26%	0.41%
Zhejiang	0.16%	0.36%
Anhui	0.20%	0.45%
Fujian	0.16%	0.31%
Jiangxi	0.31%	0.50%
Shandong	0.09%	0.30%
Henan	0.14%	0.40%
Hubei	0.21%	0.35%
Hunan	0.19%	0.28%
Guangdong	0.79%	0.85%
Guangxi	0.58%	0.64%
Hainan	0.98%	0.43%
Chongqing	0.08%	0.25%
Sichuan	0.05%	0.16%
Guizhou	0.00%	0.12%
Yunnan	0.00%	0.16%
Tibet	0.00%	0.05%
Shaanxi	0.02%	0.13%
Gansu	0.00%	0.15%
Qinghai	0.00%	0.09%
Ningxia	0.00%	0.11%
Xinjiang	0.00%	0.07%

Indicator 4.1.3: Economic costs of air pollution-related premature deaths

Methods

The methodology for this indicator differs from its counterpart in the global Lancet Countdown report, which refers to the monetary values of lost years of life due to ambient $PM_{2.5}$ pollution, as well as the methods used in Indicator 4.1.1 of this report. Here, using methodology similar to that of Indicator 4.1.2, this indicator measures the reductions in industrial output resulting from $PM_{2.5}$-related premature deaths of labourers. The calculation is based on the Ghosh model under the Input-Output (IO) analytical framework.[103,104] The approach considers the $PM_{2.5}$-related mortality of labourers as a form of labour productivity loss, and sees how the loss in each industry influences the output of other industries through inter-industrial dependencies. The model assumes that the percentage losses of labour productivity bring about the same percentage

reductions in industrial value added, which in turn affects the output of all industries adversely through the production supply chains. Therefore, it distinguishes between direct and indirect economic costs due to $PM_{2.5}$ pollution. The direct cost results from the initial decrease in value added, and the indirect cost comes from inter-industrial dependencies. The analysis is also extended to a multi-regional scale to incorporate the spill-over effect of indirect economic cost through the inter-provincial trade links.

The main procedures are as follows:

1. The calculations are first performed on the national scale using the Chinese IO tables for two years 2015 and 2018, and then on the multi-provincial scale using the Chinese Multiregional IO table in 2015. The multi-provincial analysis is performed for the single year of 2015, as it is the only year with the available Multiregional IO table. All the IO tables used in this analysis are changed from current LCU prices into constant US$ in 2015.
2. The total numbers of $PM_{2.5}$-related deaths are multiplied by the "labour force mortality rates" to calculate the absolute losses of labourers. Labour force mortality rates in this context refer to the proportions of deaths at the working age (i.e., 15-65) among deaths of all age groups. As $PM_{2.5}$-related labour force mortality rates are not available at present, the all-cause labour force mortality rates are used as reference. Different provinces have different labour force mortality rates.
3. The absolute losses of labourers are disaggregated into the primary, secondary and tertiary industries according to the sectoral results of $PM_{2.5}$-related deaths, and then divided by the sizes of industrial labour force in provinces (or the national labour force in the analysis on the national scale) to obtain the relative losses of labourers.
4. This entails the percentage losses of industrial labour productivity, which constitutes a key input variable of the Ghosh model. It is assumed that there is no capital loss caused by $PM_{2.5}$ pollution and labourers are fully employed in the economy.
5. The calculated percentage reductions in industrial labour productivity are expected to cause the same percentage reductions in industrial value added, as labour is a major component of the industrial value added. This assumption is drawn from the principle of the IO framework, which defines that proportional increase in industrial output can only be achieved by simultaneous increases in both capital and labour.[105] In other words, the shortage of any input can directly constrain the industrial output capacity, with full employment of input factors.
6. The initial loss of an industry's value added, which constitutes the direct economic cost, will have knock-on effect that reduces other industries' output through the production and supply network depicted by the IO matrix, and the aggregate of output reductions in all industries is counted as the total economic costs of premature deaths due to $PM_{2.5}$ pollution. The increase from the direct to the total costs indicates the indirect costs resulting from inter-industrial dependencies.
7. The primary, secondary and tertiary industries are identified following the same rules as Indicator 1.1.3.

Data

1. Data on premature mortality from ambient $PM_{2.5}$ pollution is provided by WG3 responsible for Indicator 3.3.2.
2. The Chinese IO tables in 2015 and 2018 are obtained from the website of the National Bureau of Statistics of China.[101]
3. The Chinese multi-regional IO table in 2015 is obtained from the CEADs dataset.[102]
4. The provincial labour force by industry is sorted from Chinese provincial statistical yearbooks.
5. The all-cause mortalities by province and age group are collected from the sixth national population census of China.[106]

Caveats

See Indicator 3.3.2, for caveats related to the calculation of premature mortality due to ambient air pollution. The morbidity rates of $PM_{2.5}$ pollution, which could entail larger economic costs, are not incorporated in this analysis due to the short time limits of the project. However, this is made up by comparing the results with previous work that considers both $PM_{2.5}$-related mortality and morbidity rates.[103,104] The comparison would deliver more comprehensive information on the economic costs of $PM_{2.5}$ pollution. The industrial labour

losses are not derived directly from the sectoral results of $PM_{2.5}$-related deaths, as deaths attributed to a certain sector (e.g., the transport sector), do not necessarily mean deaths taking place within that sector. The breakdown of labour losses into the three industries is weighted-proportional to the regional employment in the three industries. For example, it is assumed that most $PM_{2.5}$-related labour deaths attributed to the agricultural sector fall into the primary industry, while those attributed to the transport sector belong mainly to the secondary and tertiary industries. Therefore, the primary industry is given more weight when proportionally disaggregating the labour deaths with agricultural causes into the three industries, while the secondary and tertiary industries are given more weights for those attributed to the transport sector. The report employs a supply-driven IO model that fixes the input proportions between different kinds of productive factors. This means that producers do not seek for substitutive factor inputs when labour employment decreases due to $PM_{2.5}$-related mortality. The model also excludes the possibility of market-based price adjustment, such as rising wages, to encourage the working enthusiasm of the remaining labourers. Such rigidity decides that the $PM_{2.5}$-related economic cost is estimated on the short-term scale with constant economic conditions. In 2015, due to different IO tables used, the sum of provincial costs connected to $PM_{2.5}$ pollution in the multi-regional analysis may be slightly deviated from the national costs in the single-region analysis. Finally, this indicator considers the economic costs of mortality related to people's ability to work, however it does not consider the monetary value people place on life (i.e., VSL).

Future Form of Indicator

An ideal form of this indicator would reflect economic costs resulting from both mortality and morbidity rates of $PM_{2.5}$ pollution. This can be developed in future iterations of this indicator.

Additional Information

Table 35: Chinese direct and indirect economic costs, in billions of US$ in 2015, from premature mortality of PM2.5 pollution by industry and year.

Years	Direct losses (billion US$)			Indirect losses (billion US$)		
	Primary industry	Secondary industry	Tertiary industry	Primary industry	Secondary industry	Tertiary industry
2015	0.33	1.49	1.69	0.23	5.52	1.55
2018	0.35	1.62	1.90	0.22	4.89	1.71

Table 36: Chinese economic costs, in terms of shares in regional GDP, from premature mortality of ambient air pollution by province in 2015.

Provinces	Direct costs (% of regional GDP)	Indirect costs (% of regional GDP)
Beijing	0.04%	0.05%
Tianjin	0.05%	0.07%
Hebei	0.06%	0.09%
Shanxi	0.04%	0.06%
Inner Mongolia	0.05%	0.05%
Liaoning	0.06%	0.09%
Jilin	0.05%	0.08%
Heilongjiang	0.04%	0.05%
Shanghai	0.03%	0.05%
Jiangsu	0.03%	0.07%
Zhejiang	0.02%	0.06%
Anhui	0.03%	0.10%
Fujian	0.02%	0.04%

Jiangxi	0.02%	0.05%
Shandong	0.05%	0.10%
Henan	0.05%	0.10%
Hubei	0.04%	0.06%
Hunan	0.04%	0.05%
Guangdong	0.03%	0.05%
Guangxi	0.03%	0.05%
Hainan	0.01%	0.04%
Chongqing	0.05%	0.08%
Sichuan	0.05%	0.08%
Guizhou	0.04%	0.05%
Yunnan	0.02%	0.04%
Tibet	0.00%	0.01%
Shaanxi	0.05%	0.06%
Gansu	0.03%	0.05%
Qinghai	0.03%	0.04%
Ningxia	0.03%	0.05%
Xinjiang	0.05%	0.06%

Indicator 4.1.4: Economic losses due to climate-related extreme events

Methods

The methodology of this indicator is different from its counterpart in the global report, as it includes both direct and indirect economic losses of climate-related extreme events. Direct losses are the physical or tangible damage due to these events, which is reported in the global report. Indirect losses refer to the subsequent losses, including business interruption losses of affected economic sectors, and the spread of losses towards other initially non-affected economic sectors, and the costs of recovery processes.[107]

In this report, the indirect losses are calculated as the reductions of industrial Gross Value Added (GVA), drawing on the Flood Footprint model which highlights the significance of inter-industrial dependencies and post-disaster recovery costs in the economic impact assessment of disaster risks.[108-110] Loss values and all other economic data used in this analysis are converted into US$ 2015 terms.

The main procedures are as below:

1. Five years 2007, 2010, 2012, 2015 and 2017 are selected to perform the calculation of economic losses due to climate-related extreme events on the national scale. These are the years when Chinese Input-Output (IO) tables are available. The national IO tables used in this analysis are converted from current LCU prices into constant US$ in 2015, and then divided by the number of months per year as the calculation is performed on monthly basis.
2. Annual direct damage due to climate-related extreme events is obtained on the national scale, and is further broken down into three industrial sectors and a residential sector, according to the proportions based on empirical evidence of Chinese flooding events between 1961-1990.
3. The annual damage values are then split into five months (from May to September), as the summer seasons are considered as highly risky with climate-related extreme events. Therefore, the modelling follows a "consecutive multiple events" strategy that is originally introduced by Zeng

and Guan [108].

4. The absolute direct damage (in US$ 2015 terms) is divided by the amount of industrial capital stock to get the percentage losses of capital productivity. The Chinese capital stock by industry is calculated using the perpetual inventory method, which is a common practice in the field of capital stock estimation.[111-113]
5. The percentage losses of industrial capital productivity are delivered into the Flood Footprint model to calculate the indirect losses due to climate-related extreme events. The capital productivity loss of a certain industry initially reduces its own output capacity, and then spreads to other industries through inter-industrial dependencies, which are depicted by the IO matrix of that year. The capital loss in the residential sector does not affect industrial output capacity directly, as this sector does not participate in production activities. However, the reconstruction requirements in the residential sector competes for resources with the industrial sectors, which in turn slows down the recovery of the industrial output capacity. The reconstruction demands in both the industrial and residential sectors are satisfied through the proportional rationing scheme among all categories of final uses.
6. The indirect losses are calculated as the accumulative losses of industrial GVA until the economy is recovered to its pre-event levels.
7. The three major industrial sectors are identified following the same rules as Indicator 1.1.3. The first or primary industry refers to agricultural, forestry and fishing activities, the secondary industry includes mining, manufacturing, utilities and construction, and the third or tertiary industry includes transport, trade, catering services, finance, real estate and other services.

Data

1. Data on direct damage is sourced from the NatCatSERVICE of Munich Re.[114]
2. The Chinese IO tables between 2007 and 2017 are obtained from the website of the National Bureau of Statistics of China.[101]
3. Chinese GDP are from the World Bank Development Indicator Database.[115]
4. Data on Chinese industrial capital stock is derived from the IMF's Investment and Capital Stock Dataset (ICSD),[116] and Statistical Yearbooks of the Chinese Investment in Fixed Assets.[117]

Caveats

The model has assumed no market-based price adjustment and substitution of suppliers, encountering event-induced shortage of intermediate supplies. Categories of input factors are employed in fixed proportions, which are defined by the IO matrix of the year, during the production processes. This means that the economic losses of climate-related extreme events are calculated on the short term, generally no more than two years, until the economy is recovered to the pre-disaster level.In addition, the model does not consider productivity losses of labourers, another key productive factor, resulting from climate-related extreme events, as such data is not available at present. However, empirical evidence shows that compared to the percentage losses of capital, the relative losses of labour are usually much lower, so that they have little effect on the modelling results.

Future Form of Indicator

In the future, this indicator will be developed to present Chinese multi-provincial economic losses due to climate-related extreme events, using the Multi-regional Disaster Footprint model, when data on physical damage is improved on the provincial scale.

Findings

The economic losses in China, relative to GDP, due to climate-related extreme events, were declining after peaking in 2010, the year most severely hit by extreme events. However, recent losses in 2017 still reached nearly US$20 billion (0.18% of GDP) (Table 35). This is consistent with the pattern of physical damage

caused by these events estimated by Munich Re over the years.[114] For all years, the total losses reached on average 134% of direct losses. This result is slightly lower than 139% in Hallegatte [118] who estimated the economic losses of Katrina in Louisiana. A major part (43%-51%) of indirect losses were found in the primary industry, although its direct losses stayed lower than other industries, both in relative and absolute terms. This is caused by the low capital intensity (capital per output) in this industry, which makes smaller physical losses bring about greater reduction in its capital productivity, and thus higher indirect losses.

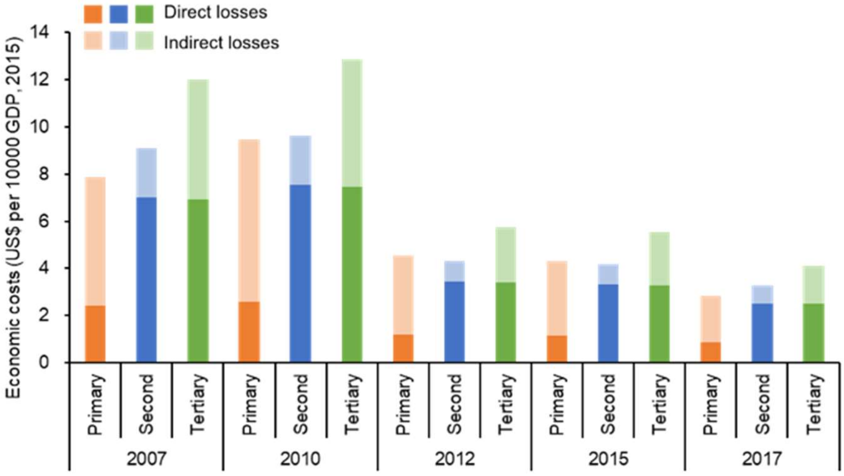

Figure 35: Economic losses from climate-related extreme events relative to GDP (dark color indicates direct costs and light one indicates indirect costs)
GDP = gross domestic product. US$ 2015 = based on the value of the US dollar in 2015.

Table 37: Chinese direct and indirect losses, relative to GDP, from climate-related extreme events by industry and year.

Years	Direct losses/US$10000 GDP			Indirect losses/US$10000 GDP		
	Primary industry	Secondary industry	Tertiary industry	Primary industry	Secondary industry	Tertiary industry
2007	2.42	6.99	6.92	5.44	2.09	5.07
2010	2.61	7.52	7.45	6.85	2.12	5.39
2012	1.19	3.43	3.40	3.37	0.89	2.32
2015	1.16	3.33	3.30	3.14	0.86	2.23
2017	0.87	2.52	2.49	1.94	0.75	1.60

Indicator 4.2: The Economics of the Transition to Zero-Carbon Economies

Indicator 4.2.1: Healthy energy investments

Methods

The methodology for this indicator remains the same as described in the 2018 global Lancet Countdown report appendix, however the 2019 global Lancet Countdown report used the updated changed definition of investment given by the IEA. The revised approach from IEA considered ‘ongoing’ capital spending, with investment in a new plant spread evenly from the year new construction begins, to the year it becomes operational. In this Chinese Lancet Countdown report and previous global reports, data was presented as

‘overnight’ investment, in which all capital spending on a new plant is assigned to the year in which the plant became operational.

The data for this indicator is from the Wind Economic database.[119] Wind is a comprehensive and paid database which massively combines macro and sectoral data. It is commonly used for financial and macro analysis. Four categories of energy investment are defined:

• Fossil-fired power – investment in fixed capital information and constructing power generation facilities of coal-, gas-, and oil-fired electricity.
• Nuclear –investment in fixed capital information and constructing power generation facilities of nuclear electricity.
• Hydro power – investment in fixed capital information and constructing power generation facilities of hydroelectricity.
• Wind power – investment in fixed capital information and constructing power generation facilities of wind electricity.
• Solar PV – investment in fixed capital information and constructing power generation facilities of solar electricity.
• Biomass – investment in fixed capital information and constructing power generation facilities of biomass electricity.
• Grid – investment in fixed capital information of constructing overall power grid.

There are two types of investments for each kind of energy in the power sector. One is the investment in fixed capital formation, which is a general term for the workload of constructing and purchasing fixed assets and the expenses related to it in a certain period (type 1). The other is investment in constructing power generation facilities including construction of electricity grid and power networks (type 2, Table 38). Considering the data continuity, especially the availability of data in 2018-2019, only the latter type of investment was analyzed in this indicator.

Data

1. Energy investment data, listed by wind, hydro, nuclear, coal and overall power grid, is taken from the Wind Economic database.[119]
2. The National Power Industry Statistics Data contributes the results of Solar PV and it originates from National Energy Administration of PRC.[120]
3. Biomass data is taken from the China National Renewable Energy Center (CNREC) Renewable Energy Outlook 2019.[121]
4. Values presented are in US$ 2019 billion, based on the value of RMB in 2015 and the exchange rate according to National Bureau of Statistics of China. [122]

Caveats

Renewable energy investment here mainly includes centralized project but excludes investment in decentralized facilities. In the original dataset, there are two types of investment dataset. Type 1 is investment in fixed capital formation, and Type 2 is investment in constructing power generation facilities. Type 1 doesn’t include solar PV and is only available up to 2017. Type 2 is more comprehensive and includes data until 2019, however, it doesn’t include other facilities related to renewable energy power generation and distribution. It is worth noting that type 2 is part of type 1 and investment in constructing power generation facilities could partially represents the future use of this energy. Furthermore, we also used data from China’s Renewable Energy Outlook 2019[121] for biomass.

Data on the recent investments in energy efficiency improvement is not available. Meanwhile, low-carbon energy may also include sectors other than power generation, although data is not available. This indicator for provincial level should be updated for the most recent years. This indicator should be updated with the data before 2010.

Future Form of Indicator

This indicator for provincial level should be updated for the most recent years. This indicator should be updated with the data before 2010. Further datasets containing data on investments in energy efficiency and low-carbon enery in sectors other than power generation will be explored.

Additional Information

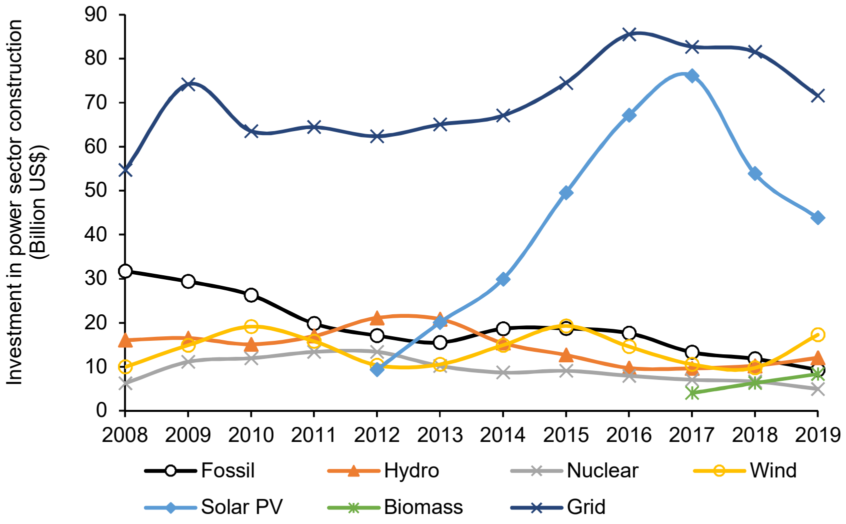

Figure 36: **Investment in power sector construction from 2008 to 2019**

Table 38: Investment in power sector construction from 2008 to 2019 (US$ billion)

	2008	2009	2010	2011	2012	2013	2014	2015	2016	2017	2018	2019
Fossil	31.72	29.38	26.28	19.81	17.08	15.50	18.64	18.67	17.62	13.29	11.79	9.29
Hydro	16.04	16.50	15.09	16.97	21.11	20.81	15.35	12.67	9.71	9.64	10.23	12.00
Nuclear	6.23	11.12	11.93	13.35	13.37	10.17	8.67	9.07	7.93	7.03	6.63	4.94
Wind	9.97	14.88	19.12	15.78	10.35	10.54	14.90	19.27	14.59	10.55	9.74	17.27
Solar PV					9.34	20.06	29.89	49.52	67.15	76.12	53.92	43.84
Biomass										4.00	6.29	8.34
Grid	54.71	74.19	63.53	64.44	62.37	65.04	67.06	74.50	85.50	82.72	81.53	71.61
Non-fossil	32.24	42.50	46.14	46.10	54.16	61.58	68.81	90.52	99.39	107.34	86.80	86.39
Total [Generation]	63.96	71.88	72.42	65.91	71.24	77.08	87.45	109.19	117.01	120.64	98.59	95.68
Total	118.67	146.07	135.95	130.35	133.62	142.12	154.52	183.69	202.50	203.35	180.13	167.29

[Notes: originally derived from Wind Economic database and China Renewable Energy Outlook 2019[121]]

Indicator 4.2.2: Employment in low-carbon and high-carbon industries

Methods

This indicator presents China's direct employment in fossil fuel extraction industries, including coal mining,

oil and gas exploration and extraction, as well as direct and indirect employment in renewable energy.

The data for this indicator is sourced from REN21 Global Status Report 2019 [123] (renewables) and CEIC Data (2012-2019)[124] (fossil fuel extraction), National Bureau of Statistics of China[122].

Renewable industries included are:

- Hydropower
- Solar energy
- Wind energy;
- Bioenergy;
- Other technologies.

Bioenergy includes liquid biofuels, soil biomass and biogas. Solar energy includes solar heating/cooling; solar photovoltaic and concentrated solar power, 'Other technologies' includes geothermal energy, ground-based heat pumps, municipal and industrial waste, and ocean energy. Fossil fuel extraction includes coal mining, oil and gas exploration and production.Fossil fuel extraction values include direct employment, whereas renewable energy jobs include direct and indirect employment (e.g. equipment manufacturing), except for large hydropower (direct employment only).

Due to an improvement in data collection and estimation methodology, employment values reported for other technologies are unavailable in some years.

Data

1. Data on renewable energy employment is sourced from REN21 Global Status Report 2019.[123]
2. Data on employment in fossil fuel extraction is from CEIC Data (2012-2019)[124], National Bureau of Statistics of China[122]

Caveats

The caveats of this indicator can be described in three aspects. Provincial level data is not available for most recent years and employment in low-carbon industries data is only available from 2012. Both direct and indirect employment in renewable industries are counted, whereas only direct employment in fossil fuel extraction is considered for employment in fossil fuel industries.

.

Future Form of Indicator

An ideal future form of this indicator would track both direct and indirect employment from the renewables and fossil fuel extraction industries, along with the provincial level distribution in their change over time.

Additional Information

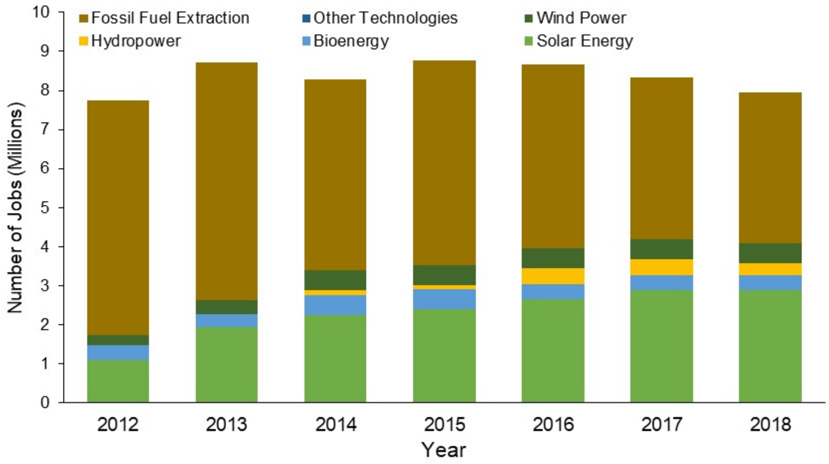

***Figure 37*: Employment in renewable energy and fossil-fuel extraction sectors**

Table 39: China employment in renewable energy and fossil-fuel extraction sectors (Million Jobs)

	2012	2013	2014	2015	2016	2017	2018
Solar Energy	1.1	1.93	2.241	2.395	2.663	2.897	2.875
Bioenergy	0.38	0.354	0.521	0.521	0.376	0.376	0.382
Hydropower	0	0	0.126	0.1	0.407	0.407	0.308
Wind Power	0.267	0.356	0.502	0.507	0.509	0.51	0.51
Other Technologies	0	0	0	0	0	0.002	0.003
Fossil Fuel Extraction	5.996	6.072	4.884	5.238	4.72	4.13	3.881

Indicator 4.2.3: Fossil fuel subsidies

Methods

The methodology for this indicator is the same as described in the 2019 global Lancet Countdown report appendix.[1] The data for this indicator is taken from the IEA,[125] which is calculated based on the price-gap approach. As the most commonly applied methodology for quantifying consumption subsidies, the price-gap approach compares average end-user price paid by consumers with reference prices that reflect full cost of supply. Therefore, the price gap equals to the amount by which an end-use price falls short of the reference price, indicating the presence of a subsidy. Prices are presented in US$2018. The data required for the price-gap calculations are extensive. Original data and a detailed description of the calculation methodology can be obtained from the IEA (2019).[126]

Data

1. IEA, Fossil-fuel consumption subsidies by country.[125]

Caveats

Coal consumption were not available for any of the years reported, and gas consumption subsidies were unavailable for some years, due to the lack of consistent data. Moreover, these values do not include the economic value of the unpriced negative externalities.

Future Form of Indicator

Future versions of this indicator will aim to have the consistent inclusion of production and consumption subsidies for all fuels, especially coal, available on an annual basis. Similar to the global Lancet Countdown report, a 'net carbon price' indicator will be developed, including data from this indicator, along with that of the carbon pricing (Indicator 4.3.1).

Additional Information

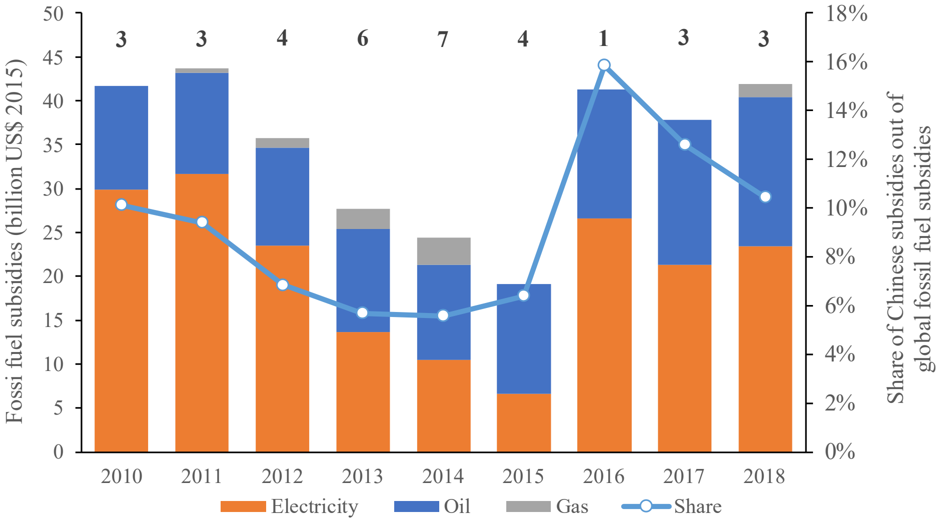

Figure 38: **Fossil fuel and electricity consumption subsidies in China, 2010-2018**
Note: The number on top of each bar represents the rank of fossil fuel subsidy of China in the world on the corresponding year.

Table 40: Fossil fuel consumption subsidies in China, 2010-2018 (million 2015 US$)

Year	Oil	Electricity	Gas	Coal	Total
2010	11885.9	29827.3	-	-	41713.1
2011	11450.1	31697.7	530.4	-	43678.2
2012	11086.9	23531.6	1065.6	-	35684.0
2013	11728.9	13634.7	2358.3	-	27721.9
2014	10840.4	10520.0	3024.9	-	24385.3
2015	12457.5	6661.9	-	-	19119.4
2016	14658.8	26599.9	-	-	41258.8
2017	16437.6	21343.0	-	-	37780.7
2018	16953.9	23450.3	1520.4	-	41924.5

Indicator 4.2.4: Coverage and strength of carbon pricing

Methods

The methodology for this indicator remains the same as described in the appendix of the 2019 global Lancet Countdown report. Data for this indicator, including general information and daily real-time prices, are from the World Bank Carbon Pricing Dashboard and the websites of carbon pilot markets in China. Price data

period is from Jan. 2014 to April 2020 for eight pilot markets, including Beijing, Shanghai, Guangdong, Tianjin, Hubei, Chongqing, Fujian, and Shenzhen. Annual weighted average prices are calculated from daily price data for these eight pilot markets. GHG coverage data is presented as the proportions of 2012 global (53,937 MtCO2e), national and jurisdiction's anthropogenic GHG emissions are based on emission data from EDGAR (Emissions Database for Global Atmospheric Research) as well as the coverage calculations from World Bank Carbon Pricing Dashboard. Here the "proportion of jurisdiction's GHG emissions covered" is calculated by dividing the covered quantity of emissions of carbon markets by the total GHG emissions in the corresponding administrative region. For example, when calculating the emission coverage of regional pilots, then the "jurisdiction's emission" is the sum of all GHG emissions in these pilot regions. Here data is presented for 2019.

Data

1. Data on carbon prices is taken from the World Bank Carbon Pricing Dashboard;[127]
2. GHG emissions data is taken from EDGAR.[128]

Caveats

The instruments experience some overlap in emission coverage with China's national ETS (Table 41). The to-be-implemented China national carbon market in 2021 and the overlapping of regional pivots with it are also considered. Here the "proportion of jurisdiction's GHG emissions covered" is calculated by dividing the covered quantity of emissions of carbon markets by the total GHG emissions in the corresponding administrative region. The time series plot shows the annual average prices of carbon in eight Chinese pilot carbon markets (Table 42). All these markets open in the year 2013 or later, and the prices are somewhat fluctuant. Generally, the prices are relatively low, especially compared to the prices of carbon pricing initiatives in other countries, which are typically above US$15 and could also be as high as more than US$100 (Sweden carbon tax). Currently, the prices of these pilots are probably unable to support the climate target "well below 2°C" as literatures show that the required carbon price might be US$40-80 by 2020.

Additional information

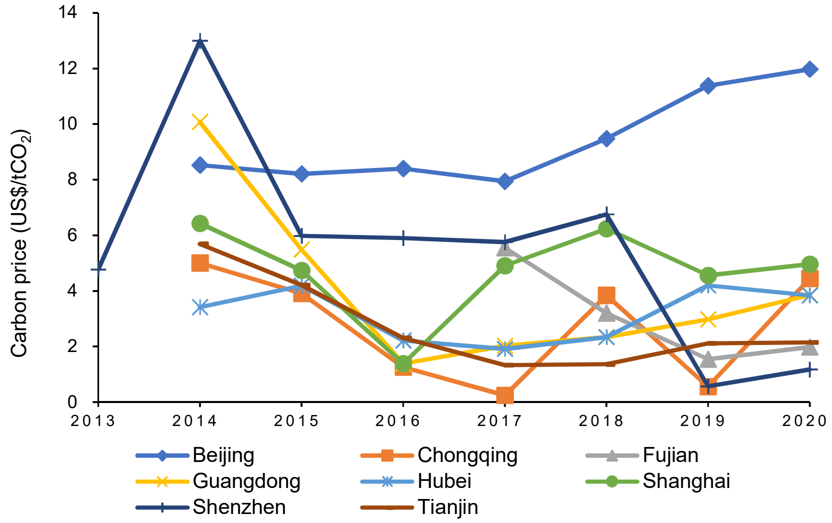

Figure 39: **Carbon prices in eight pilot markets in China**

Table 41: Coverage of carbon pricing in China

Carbon pricing coverages	GHG emissions	Proportion of global	Proportion of	Proportion of

	covered [MtCO2e]	GHG emissions covered	China's GHG emissions covered	jurisdiction's GHG emissions covered
Total, regional pilots, including overlapping#	1330.03	2%	11%	53%
Total, regional pivots, excluding overlapping	864.52	1%	7%	35%
Total, China + regional, including overlapping	4561.93	8%	37%	31%
Total, China + regional, excluding overlapping	4121.14	8%	33%	28%

Here the “overlapping” means the overlapping of regional carbon market with China’s national carbon trade program which is scheduled to be implemented in 2021

Table 42: Carbon prices in eight pilot markets in China, US$ /tCO_2

Name of the initiative	2013	2014	2015	2016	2017	2018	2019	2020
Beijing pilot ETS		8.52	8.21	8.40	7.94	9.48	11.38	11.97
Chongqing pilot ETS		5.00	3.91	1.26	0.24	3.84	0.56	4.44
Fujian pilot ETS					5.56	3.20	1.546	1.98
Guangdong pilot ETS		10.08	5.49	1.39	2.02	2.33	2.97	3.85
Hubei pilot ETS		3.41	4.18	2.21	1.91	2.33	4.20	3.84
Shanghai pilot ETS		6.42	4.73	1.38	4.90	6.23	4.56	4.96
Shenzhen pilot ETS	4.77	12.99	5.98	5.90	5.76	6.76	0.56	1.17
Tianjin pilot ETS		5.69	4.21	2.30	1.32	1.36	2.11	2.15

Section 5: Public engagement

Indicator 5.1: Media coverage of health and climate change

Methods

Although around 1.4 billion newspapers are sold per month in China, these numbers have been declining since 2013.[129] On the other hand, social media platform Weibo has been increasing its users in recent years, with around 400 million active users in 2020 with 130 million words and 1.5 million videos published on this platform every day and 64,000 average posts per minute.[130] For this indicator, three types of media accounts on Weibo to study coverage of climate change and health in social media, including official media (@People’s Daily), commercial media (@The Beijing News and @Caixin), and professional media (@Health News and @China Science Daily), were selected. Weibo posts from January 2010 to December 2019 were searched and collected using a python-based crawler. Due to the word limit of Weibo posts, posts are generally very short, so when searching for related posts, as long as a relevant keyword appears, it is regarded as a qualified post. First, posts containing climate change-related keywords. The choice of keywords in accordance to previous research of media coverage of health and climate change for People’s Daily in China in the global Lancet Countdown reports (**Table 43**).

All health and climate change-related posts were manually screened to avoid false-positive results. Posts containing keywords but irrelevant to health and climate change are excluded from the search result.

Table 43: Chinese keywords for the search in Weibo

Climate change-related keywords		Health-related keywords	
Chinese	English	Chinese	English
气候变化	Climate change	疟疾	Malaria
全球变暖	Global warming	腹泻	Diarrhea/ Scour
温室	Greenhouse	感染	Infected
极端天气	Extreme weather	肺炎	Pneumonia
全球环境变化	Global environment change	流行病	Epidemic
低碳	Low carbon	公共卫生	Public health
可再生能源	Renewable energy	卫生	Hygiene

碳排放	Carbon Production	发病	Disease outbreak
二氧化碳排放	Carbon dioxide emissions	营养	Nutrition
气候污染	Air pollution	精神障碍	Mental disorders
气候	Climate	发育	Puberty growth
全球升温	Global warming	传染	Infection
再生能源	Renewable energy	疾患	Disease
CO2 排放	CO2 emissions	症	Symptom
温室气体	Greenhouse gas	瘟疫	Epidemic
极端气候	Extreme weather	流感	Flu
高温	High temperature	流行感冒	Influenza
变暖	Warming	治疗	Treatment
排放	Emission	保健	Health care
环境变化	Environmental change	健康	Health
升温	Warming	死亡	Death
全球温升	Global warming	精神疾病	Mental disease
热浪	Heat wave	精神病	Mental illness
暴雨	Rainstorm	登革热	Dengue
气温	Temperature	饥饿	Hunger/ Famine/ Starvation
洪水	Flooding	粮食	Food
洪灾	Inundation	有害	Harmful
气候反常	Abnormal climate	皮肤病	Dermatosis
野火	Wildfire	风湿	Rheumatism
山火	Forest fire	呼吸系统疾病	Respiratory diseases
雪灾	Snowstorm	人类健康	Human health
低温	Low temperature	人体健康	Physical health
年代际	Interdecadal	身体健康	Body health
冰雪	Ice and snow	心脏病	Heart disease
可持续发展	Sustainable development	糖尿病	Diabetes
海洋酸化	Ocean acidification	疾病	Illnesses
静稳	Stagnant	热死	Heat death
		口罩	Face mask
		防护	Protection

Data

1. Posts published by Weibo accounts @People's Daily, @The Beijing News, @Caixin, @Health News, @China Science Daily were collected from January, 2010 to December, 2019;
2. Choice of keywords in accordance to previous research of media coverage of health and climate change for People's Daily in China in the global Lancet Countdown report.[1]

Future Form of Indicator

1. The number of social media accounts can be expanded in the future.
2. The keywords used in this research are obtained from the study of media coverage of health and climate change for People's Daily in China after manual screen, which is a traditional media. Therefore, the keywords should be edited to be more in line with the characteristics of social media in the future research.

Additional Information

Figure 40 and ***Figure 41*** illustrate the number of climate change-related and health and climate change-

related posts, respectively. The official media People's Daily published the highest number of posts on health and climate change (296) over the whole 2010-2019 period while commercial media Caixin had the highest number of posts on climate change (2266). Health News posted lowest on both topics. However, the Health News had the highest ratio of health-related issues to climate change-related posts and Caixin had the lowest. Therefore, although official media and commercial media cared about health problems and climate change-related topics, they did not connect them together while professional media had more consideration on their connection.

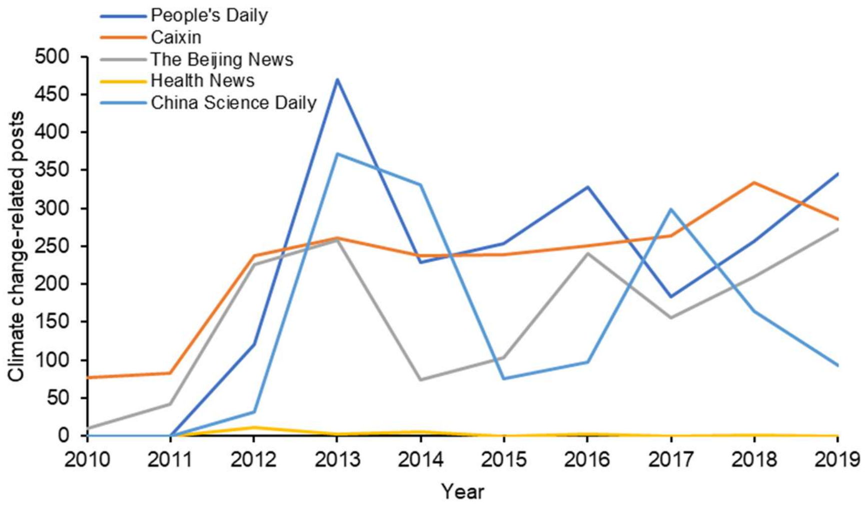

Figure 40: Coverage of climate change on five media accounts on Weibo

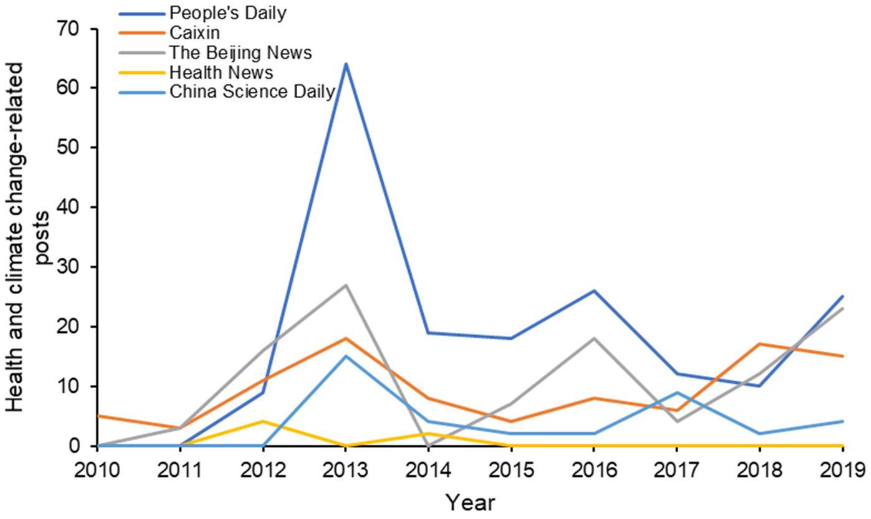

Figure 41: Coverage of climate change and health on five media accounts on Weibo after manual screening

Indicator 5.2: Individual engagement in health and climate change

Methods

This indicator analyses individual engagement in health and climate change through search queries related

to health and/or climate change, through the search engine, Baidu, the most widely used search engine in China, with more than 1.1 billion users which covers most of the population in China. [131] Baidu always takes more than 66% of the market share of the search engine in China over the past decades. [132] The keywords, developed by this team of researchers, were designed to capture search queries related to health and climate change. The used keywords are mainly based on the health and climate change search terms for indicator of media coverage of health and climate change in the global Lancet Countdown 2019 report,[1] but also include more search terms to improve the coverage in Chinese. All the queries in Baidu™ search engine in which contain a minimum of one health keyword (**Table 44**) were identified as health queries. Similarly, queries with at least one climate change keyword (**Table 45**) were identified as climate change queries. The queries in which appeared keywords from both (I) health, and (II) climate change were identified as health&climate change co-queries.

The false-positive ratio for such keyword matching method for search queries is low. At first, the length of the queries is short. According to our statistics, the average Chinese character length of climate change queries, health queries and health&climate change co-queries in our data is 15.87, 12.80 and 20.73, respectively. Given such short query length windows, the queries containing the corresponding keywords have a high probability to be related to the topic of the keyword. Furthermore, we also manually examined 1000 matched queries for each type of keywords. The false-positive ratios for climate change queries, health queries and health&climate change co-queries are 2.0%, 6.1% and 5.1%, respectively.

Table 44: Health-related keywords

**Health-related keywords in Chinese**	**Health-related keywords in English**
健康	Healthy
疾病	Disease
养生	Health preservation
保健	Healthcare
公共卫生	Public health
疟疾	Malaria
死亡率	Mortality
营养	Nutrition
营养不良	Malnutrition
脱水	dehydration
发病	Morbidity
发病率	Morbidity
发育迟缓	Stunting
传染病	Communicable disease
慢性病	Chronic disease
高血压	Hypertension
肿瘤	Tumour
中风	Apoplexy
心脏病	Heart disease
肺炎	Pneumonia
癌症	Cancer
肺癌	Lung cancer
肝癌	Liver cancer
糖尿病	diabetes
肥胖	Obesity
身体超重	Overweight

非传染性疾病	Non-communicable diseases
流行病	Epidemic
流行病学	Epidemiology
腹泻	Diarrhoea
SARS	SARS
非典型肺炎	Atypical pneumonia
严重急性呼吸综合征	Severe acute respiratory syndrome (SARS)
重症急性呼吸综合症	Severe acute respiratory syndrome (SARS)
麻疹	Measles
早产	Premature
流产	Abortion
抑郁障碍	Depressive disorder
抑郁症	Depression
心理障碍	Psychological disorders
心理问题	Psychological problems
心理疾病	Mental illness
精神障碍	Mental disorders
精神病	Mental disease
精神疾病	Mental illness
精神健康	Mental health

Table 45: Climate change-related keywords

Climate change-related keywords in Chinese	Climate change-related keywords in English
气候变化	Climate change
气候变暖	Climate warming
全球变暖	Global warming
全球暖化	Global warming
全球温度升高	Global temperature rise
全球气温升高	Global temperature rise
地球温度升高	The rise of the earth's temperature
海平面上升	Sea level rise
冰川融化	Glacial melting
温室效应	Greenhouse effect
温室气体排放	Greenhouse gas emissions
碳排放	Carbon emission
二氧化碳排放	CO2 emission
碳减排	Carbon emission reduction
二氧化碳减排	Carbon dioxide reduction
温室气体减排	Greenhouse gas emission reduction
极端天气	Extreme weather
全球环境变化	Global environmental change
气候变异	Climate variability

The climate change query proportion was calculated by using the number of identified climate change queries to divide the total number of queries in the same fixed time interval. The formula of query proportion can be formulated as:

$$\textbf{climate change query proportion} = \frac{\textbf{number of identified climate change queries}}{\textbf{number of total queries}}$$

$$\frac{\textbf{Health \& climate change co} - \textbf{queries}}{\textbf{Climate change queries}} = \frac{\textbf{number of identified health\&climate change co} - \textbf{queries}}{\textbf{number of iddentified climate change queries}}$$

$$\frac{\textbf{Health \& climate change co} - \textbf{queries}}{\textbf{Health queries}} = \frac{\textbf{number of identified health\&climate change co} - \textbf{queries}}{\textbf{number of identified health queries}}$$

In order to visualise the geographical distribution of query proportion in China, the indicator was also calculated in provincial level. In order to calculate such query proportion distribution, all the queries were searched within the recent year (from 1st Jan. 2019 to 31th Dec. 2019). For each province in China, the climate change query proportion and health query proportion were calculated with the number of identified health or climate change queries in this province as numerator, and with the number of total queries in this province as denominator.

None of the queries of this study can be associated with a particular individual. Each query record only contained the query, the submission time and the submission city, without of information about the identity of any user. Furthermore, any of original search logs are being processed and used with respect to Baidu's privacy policy (https://www.baidu.com/duty/yinsiquan.html). In this study, we used the search queries all over China from Jan 2017 to Dec. 2019.

Data

1. The search query data were based on search query logs from search engine provided by Baidu Inc. All the analytics of this indicator are conducted on Baidu' servers by researchers from Baidu.

Caveats

First of all, though Baidu is the largest search engine in China, it does not cover all population groups in China. Particularly, some groups of the population, such as the elderly, children, the poor and less educated people, have few chances to use the search engine. Therefore, this indicator only reflected the public attention that is biased towards attention from typical internet users. Extending the analyses to other groups based on other complementary methods, such as survey-based method, will help to reduce this bias, and uncover the engagement heterogeneity in different population groups.

Second, this indicator uses search behavior in the search engine as a proxy for engagement phenomenon. There are other activities to engage in climate change, like reading books or attending social events. Some survey-based methods might provide some calibration for this indicator in the future.

Though the above caveats can be overcome by survey-based methods, it is not easy to calculate the indicator with as large-scale samples as the indicator based on the queries from the search engine. The high cost of the survey-based method is the main obstacle to implementing the survey-based indicator, especially considering that the respondents of the survey disperse in diverse regions over China.

In this study, since the queries were identified by keywords, the coverage and the types of keywords have an influence on the final results. Though the keywords have been enumerated with the best effort, it is possible to miss some keywords to identify the related queries.

Future Form of Indicator

In this future, this indicator will pay more attention to recall more queries related to health and climate change topic. In this study, the analysis here is based on a narrow range of keywords. Many queries which are direct or indirectly related with health and climate change may be excluded. Future work in this area will consider to cover more engagement reflected in the queries. In order to recall more queries about climate change and health, future efforts of this indicator will be made to improve the coverage of identified queries by a knowledge graph. In future, a knowledge graph about climate change will be constructed based on the scientific journal and reports. The existing medical knowledge graph can also be utilized. Then the climate change and/or health queries can be identified if the queries contain any entity in the climate change knowledge graph and/or medical knowledge graph. Based on knowledge graph, we can also provide additional forms of analysis.

Additional Information

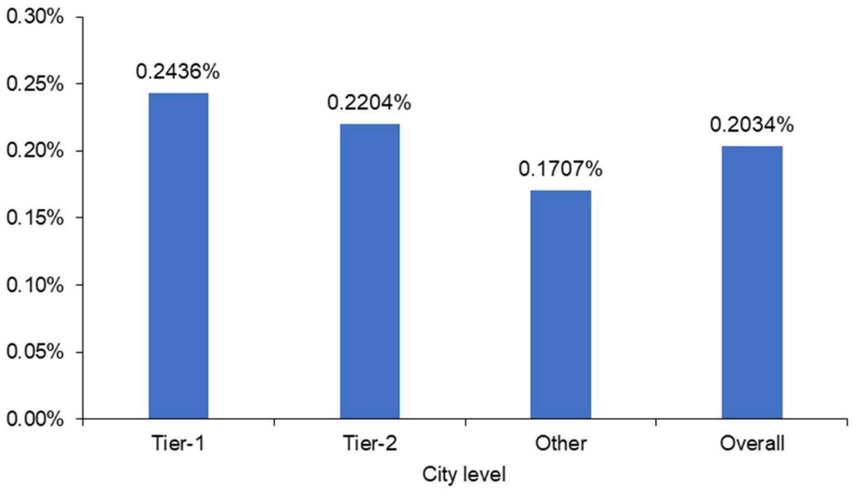

Figure 42: **Proportion of health and climate change co-queries out of total climate change queries in tier-1, tier-2 and other cities**

While the proportion of queries related to climate change continued to increase in the past three years, the queries for health and climate change are seldom co-searched by users (***Table 46***).
Further analysis also showed that the proportion of health&climate change co-query over climate change queries in developed cities is higher than in other cities in China. Here the cities are divided into three levels according to the Chinese city tier system which is a hierarchical classification of Chinese cities. The consensus in China is that four cities belong to tier-1, which are Beijing, Shanghai, Guangzhou, and Shenzhen. The tier-2 cities in this study consist of the capital city of each province plus other four vice provincial cities (Dalian, Qingdao, Ningbo and Xiamen). *Figure 43* shows the proportion of health&climate change co-queries over climate change queries of tier-1 cities was 19.74% higher than the one of overall cities, and one of tier-2 cities was 8.33% higher than the one of overall cities. Tier-1 and Tier-2 cities represent the most developed areas of China. To sum up, the proportion of the health&climate change co-queries over climate change queries in developed cities (tie-1 and tie-2 cities) is 11.53% more than the overall cities. This indicates that, with the economic development of cities, more people will engage in the impact of climate change on health.

Table 46 Queries **(per hundred thousand)** related to health and climate change in 2017-2019

Year	2017	2018	2019

$\frac{number\ of\ identified\ climate\ change\ queries}{number\ of\ total\ queries}$	0.793	1.018	0.921
$\frac{Health\ \&\ climate\ change\ co-queries}{Climate\ change\ queries}$	204.8	146.3	203.4
$\frac{Health\ \&\ climate\ change\ co-queries}{Health\ queries}$	0.396	0.359	0.467

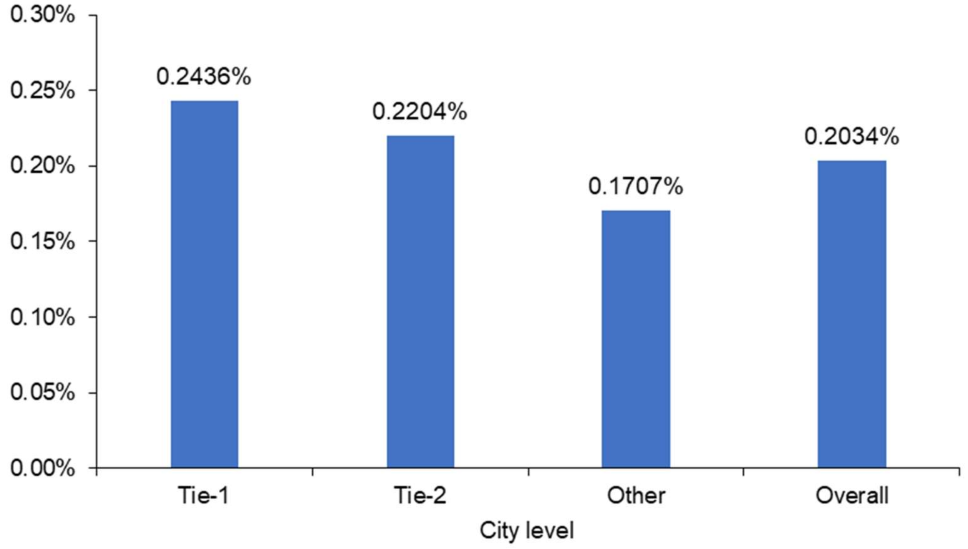

Figure 43: Share of health&climate change co-queries out of total climate change queries in tie-1, tie-2 and other cities

Figure 44 illustrates that the arid areas in Northern and Western China, and hot areas in summer, such as Chongqing, have a more substantial proportion of climate change queries, indicating more people engaging in the climate change concern than other areas in China. Meanwhile, there are also more people in Tibet engaging in the climate change since the climate change affects the world's third pole at a greater margin than nearly any other areas in China.

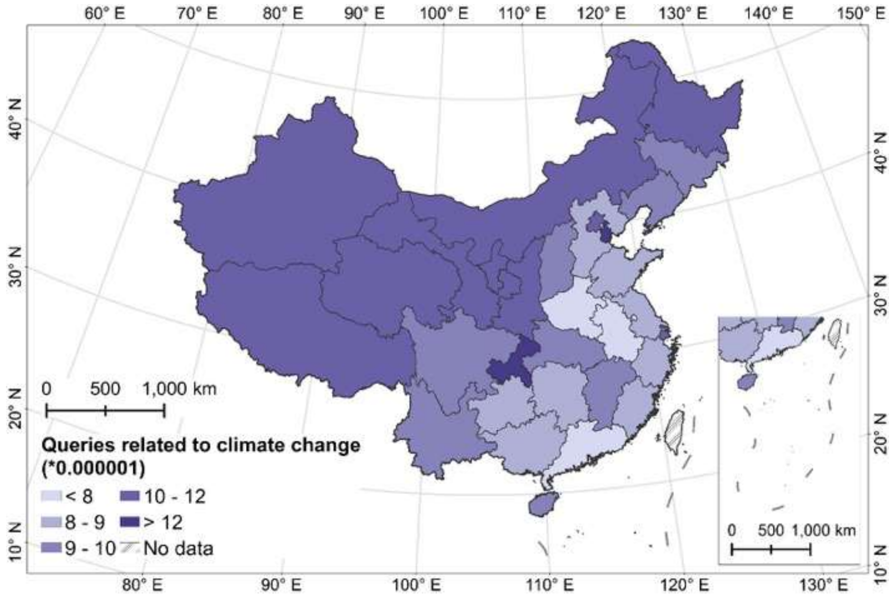

Figure 44: The distribution of the proportion of the queries related to climate change in different provinces in China in 2019.

We also observe that the COVID-19 pandemic has triggered more individual engagement in climate change, especially at the beginning of the COVID-19 pandemic in China. More analytics about COVID-19 and climate change will be provided in the follow-up report next year.

Indicator 5.3: Coverage of health and climate change in scientific journals

Indicator 5.3.1 Coverage of health and climate change in CNKI

Methods

Keywords for the topics of (a) Climate Change, and (b) Health were selected in accordance with the new climate change keywords used in the MJA-Lancet Countdown 2019 report [133] were identified as shown in ***Table 47***. Firstly, number of search result of journal articleswith the climate change-related keywords (***Table 47***) were counted from 2008 to 2019 on CNKI, a national research database, led by Tsinghua University. Secondly, the number of search results of climate change-related and health-related keywords were counted from 2008 to 2019 on CNKI. Thirdly, manually screening was used to filter articles. If the manual screening confirmed that the topic is Health and Climate Change, it is retained.

Table 47: Chinese keywords for the search in scientific journals

**Climate change-related keywords**		**Health-related keywords**	
Chinese	English	Chinese	English
气候变化	Climate change	健康	Health
全球变暖	Global warming	疾病	Disease
温室效应	Greenhouse effect	非传染性疾病<br>传染病	Non-Communicable,<br>NCD, Communicable
温室气体排放<br>温室气体减排<br>二氧化碳（碳）减排	Greenhouse gas emission,<br>Carbon emissions	流行病学	Epidemiology
干旱	Drought	生活方式	Lifestyle
野火	Bushfire	死亡	Mortality
热带气旋	Tropical cyclone	营养	Nutrition
热浪	Heatwave	营养不良	Malnutrition
		脱水	Dehydration
		发病	Morbidity
		移民	Migration
		精神疾病	Mental disorders
		协同效益（应)	Co-Benefits

Data

1. Scientific journal articles on health and climate change were searched in the national database, CNKI (https://www.cnki.net/).

Caveats

The list of keywords is very limited and it may not contain all climate change- and health-relevant keywords.

Future Form of Indicator

The source of Chinese papers and list of keywords can be further expanded in the future. Coverage in English scientific journals would be explored in next year’s research.

Additional Information

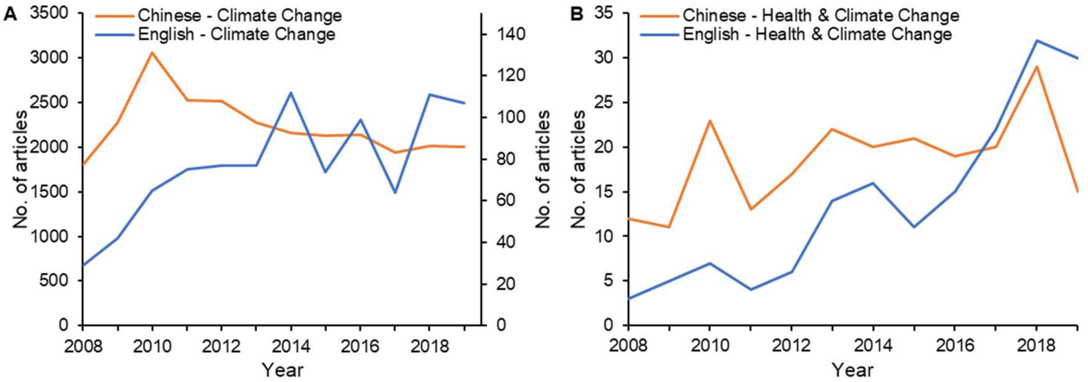

***Figure 45*: Annual coverage of climate change- and health and climate change- related journals in Chinese and English between 2008 and 2019. (A) Climate change only. (B) Health and climate change.**

Chinese scholars have been studying the impact of climate change on human infectious diseases in recent years.[134,135]

Indicator 5.3.2 Coverage of health and climate change in Ovid Embase and Ovid Medline databases

Methods

The inclusion of climate-related terms and their co-occurrence with health terms in scientific publications was tracked using a bibliometric search in both Ovid Medline (including Medline In-Process & Other Non-Indexed Citations for those citations not indexed) and Ovid Embase databases as described in the global Lancet Countdown report. Following a search unrestricted by geographical location, results for China were specifically filtered through Endnote.

The Ovid Embase and Ovid Medline databases were selected due to their coverage of health, medical and biomedical sciences, with content that is predominantly journal articles. Ovid Medline contains 25 million citations from 5600 journals, while Ovid Embase is bigger with 32 million citations from 8,500 journals. Where Medline is predominantly health and biomedicine, Embase has a greater pharmaceutical focus, all of which are relevant to health and climate change. Both databases are updated online daily and can thus provide the annual data (with a 31 December cut-off each year) needed for the indicator. These databases also function through the sophisticated Ovid interface and allow access to the comprehensive indexing systems and thesaurus of Medical Subject Headings (MeSH) for Medline and Emtree for Embase.

Also considered for use were Science Direct and the Web of Science suite of databases, but, with broad subject coverage, these would not enable the necessary search precision.

By screening the retrieved articles between 2007 and 2019, those articles that contained both health and climate change terms in their title or abstract, but do not make any meaningful link between them, were excluded. A meaningful link here means some association between climate change and an aspect of health. This link may be the focus of the article or tangential to it. As an example, climate change may be mentioned at the end of an abstract, where it is noted the health topic that is the focus of the article (e.g. dengue fever distribution) is expected to worsen or change under climate change scenarios.

Data were extracted using search filters that function via Boolean operators (AND, OR, NOT) (see below for final search strategies). For purposes of consistency and efficiency of analysis, the majority of each search

filter is designed to produce results with the search terms in either the title or abstract. Indeed, indexing is also likely to be poorly assigned or inconsistently assigned to references. The search filter is designed to retrieve all relevant results (high sensitivity) while keeping irrelevant results, and therefore effort on the part of the researchers, to a minimum (high precision).

To identify articles where associations are made between climate change and health, the filter was split into two facets, one for climate change and one for health. Terms that made up the filter were derived using both subjective and objective methods. Subjective methods included utilising terms already known by the research team, as well as those appearing in previous iterations of the Lancet Countdown. Objective methods included the use of online word frequency software (Writewords). Articles looking at health and climate change were run through this software, which organises the words or phrases in order of frequency, allowing relevant terms to be extracted.

Though this process was iterative, the climate change facet was undertaken first, as this was considered to likely consist of fewer terms and be comparatively less complex. All terms were tested independently and alongside other terms: that is, each was input into the OVID databases, from which samples of 100 were drawn and screened for relevance. Terms with high relevance were either piloted or adapted, to be tested alongside other terms and to restrict inclusion to records referring to human health. With different indexing systems, these were then translated between the databases. In addition, terms to ascertain results for editorials, comment sections, and letters were used to compare the volume of these against journal articles.

Estimates of sensitivity for the strategies were established by running the climate change facet through the Ovid interface alone, without the health facet. Samples of 1000 were then extracted and screened for relevant articles. The number of relevant articles found that were also found by the whole search strategy were divided by the number of total relevant articles found, giving an estimate of sensitivity in percentage form. For this indicator, the 90% sensitivity threshold required for systematic reviews was used (Beynon, 2013).

With an acceptable estimate of sensitivity (>90%), results of the search strategies were downloaded into Endnote and into two separate libraries: one for Medline (and Medline In-Process & Other Non-Indexed Citations), the other for Embase. Duplicates were removed from the individual libraries, before the libraries were merged and duplicates, shared across the library, were removed. The remaining records were screened for inclusion based on the inclusion and exclusion criteria outlined above for articles making a meaningful link between health and climate change. Results were screened twice by the same researcher. In addition, another researcher screened a 10% sample to ensure the criteria were met. The step-wise process of the selection of articles can be seen in ***Figure 47***.

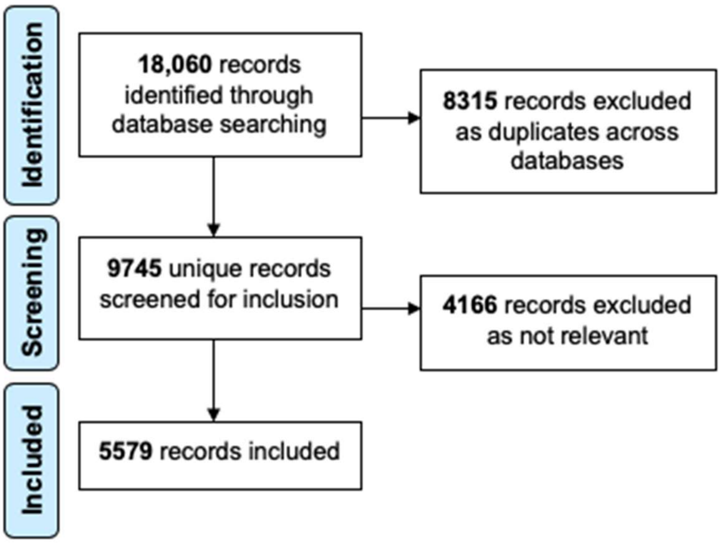

Figure 46: PRISMA flow diagram showing steps of selection process.

Numbers indicate the article count retained at each step of the process. With the applied search terms more than 18,000 scientific articles on health and climate change were identified for the period of 2007-2019. After the screening process, only 30.8% (n=5579) were retained and found to be relevant.

Following screening, precision was established by calculating the number of relevant records retrieved, divided by the total number of records retrieved. The development of the search strategy was repeated, and all of the necessary stages leading up to this point, until precision was established at over 50% for each database.

With an acceptable level of precision established for each database, the data were coded and organised in Endnote. Results for China were filtered based on the institutional location of the first author.

<u>Search terms</u>

**Medline**		**Medline (In-Process & Other Non-Indexed Citations)**		**Embase**	
**1**	carbon footprint*.ti,ab.	**1**	(climat* adj3 chang*).ti,ab.	**1**	(climat* adj3 chang*).ti,ab.
**2**	carbon footprint/	**2**	climate variability.ti,ab.	**2**	Climate Change/
**3**	(climat* adj3 chang*).ti,ab..	**3**	(climat* adj3 warming).ti,ab.	**3**	Greenhouse Effect/
**4**	climat* cris?s.ti,ab.	**4**	global warming.ti,ab.	**4**	greenhouse gas*.ti,ab.
**5**	climat* variability.ti,ab.	**5**	greenhouse effect*.ti,ab.	**5**	global warming.ti,ab.
**6**	climat* warming.ti,ab.	**6**	green house effect*.ti,ab.	**6**	Carbon Footprint/
**7**	exp Climate Change/	**7**	greenhouse gas*.ti,ab.	**7**	Greenhouse Gas/
**8**	GHG*.ti,ab.	**8**	(greenhouse adj2 emission*).ti,ab.	**8**	(greenhouse adj2 emission*).ti,ab.
**9**	global warming.ti,ab.	**9**	climat* model*.ti,ab.	**9**	(climat* adj3 warming).ti,ab.
**10**	greenhouse effect*.ti,ab.	**10**	climat* scenario*.ti,ab.	**10**	GHG*.ti,ab.
**11**	greenhouse effect/	**11**	green house emission*.ti,ab.	**11**	climat* model*.ti,ab.
**12**	greenhouse emission*.ti,ab.	**12**	GHG*.ti,ab.	**12**	climat* variability.ti,ab.
**13**	greenhouse gas*.ti,ab.	**13**	carbon footprint*.ti,ab.	**13**	carbon footprint*.ti,ab.
**14**	Greenhouse Gases/	**14**	climate induced.ti,ab.	**14**	climat* scenario*.ti,ab.
**15**	climate induced.ti,ab.	**15**	climat* cris?s.ti,ab.	**15**	greenhouse effect*.ti,ab.
**16**	climat* scenario*.ti,ab.	**16**	health.ti.	**16**	climate induced.ti,ab.
**17**	climat* model*.ti,ab.	**17**	disease*.ti.	**17**	climat* cris?s.ti,ab.
**18**	exp Health/	**18**	infectious.ti.	**18**	Ep.fs.
**19**	Global Health/	**19**	mortality.ti.	**19**	exp Malignant neoplasm/
**20**	health status/	**20**	healthy.ti.	**20**	exp skin disease/

**21**	health status disparities/	**21**	mental.ti.	**21**	exp lung disease/
**22**	exp disease/	**22**	malaria.ti.	**22**	diabetes mellitus/
**23**	exp virus diseases/	**23**	dengue.ti.	**23**	Disease association/
**24**	exp viruses/ and human*.ab.	**24**	respiratory.ti.	**24**	Western blotting/
**25**	exp Communicable Diseases/	**25**	infection*.ti.	**25**	etiology/
**26**	Infection/	**26**	wellbeing.ti.	**26**	immunology/
**27**	aedes/	**27**	well being.ti.	**27**	Infection/
**28**	water/ps	**28**	outbreak*.ti.	**28**	Death/
**29**	allergens/	**29**	zika.ti.	**29**	Cardiovascular disease/
**30**	exp Disease Outbreaks/	**30**	undernutrition.ti.	**30**	Fever/
**31**	exp Mortality/	**31**	influenza.ti.	**31**	health/
**32**	mo.fs.	**32**	hospitali?ation*.ti.	**32**	Mental disease/
**33**	exp Malaria/	**33**	epidemic.ti.	**33**	Epidemiology/
**34**	exp disease transmission, infectious/	**34**	ecohealth.ti.	**34**	Cerebrovascular accident/
**35**	exp Neoplasms/	**35**	ebola.ti.	**35**	hospital admission/
**36**	exp Heat Stress Disorders/	**36**	death.ti.	**36**	anemia/
**37**	exp Fever/	**37**	kills.ti.	**37**	Chronic disease/
**38**	exp Metabolic Diseases/	**38**	cholera.ti.	**38**	public health/
**39**	exp Death/	**39**	foodborne.ti.	**39**	cancer risk/
**40**	exp Skin/re	**40**	epidemics.ti.	**40**	Virus infection/
**41**	exp Environmental Illness/	**41**	endemic.ti.	**41**	kidney failure/
**42**	Community-Acquired Infections/	**42**	pandemic.ti.	**42**	Mental health/
**43**	exp Mental Disorders/	**43**	syndrome.ti.	**43**	Neurologic disease/
**44**	Environmental Exposure/ae	**44**	asthma.ti.	**44**	Health status/
**45**	nutrition disorders/	**45**	illness*.ti.	**45**	exp Birth weight/
**46**	child nutrition disorders/	**46**	morbidity.ti.	**46**	Human immunodeficiency virus/
**47**	exp Rickettsiaceae/	**47**	cancer.ti.	**47**	exp zoonosis/
**48**	exp infant nutrition disorders/	**48**	malnutrition.ti.	**48**	prophylaxis/
**49**	exp malnutrition/	**49**	mental health.ti.	**49**	Disease transmission/
**50**	exp wasting syndrome/	**50**	mental disorder*.ti.	**50**	Gastrointestinal disease/

**51**	exp encephalitis/	**51**	(global adj2 nutrition*).ti.	**51**	Infection risk/
**52**	salmonella infections/	**52**	(population adj2 nutrition*).ti.	**52**	Mental stress/
**53**	Helminthiasis/	**53**	(security adj2 nutrition*).ti.	**53**	antivirus agent/
**54**	food contamination/	**54**	(insecurity adj2 nutrition*).ti.	**54**	exp allergen/
**55**	zoonoses/	**55**	(global adj2 food adj2 (supply or production)).ti.	**55**	Childhood disease/
**56**	Noncommunicable Diseases/	**56**	(security adj2 food).ti.	**56**	immunogenicity/
**57**	health.ti.	**57**	(insecurity adj2 food).ti.	**57**	malnutrition/
**58**	disease*.ti.	**58**	lyme disease.ti.	**58**	Pregnancy outcome/
**59**	infectious.ti.	**59**	Chikungunya.ti.	**59**	exp *malaria/
**60**	mortality.ti.	**60**	Hantavirus.ti.	**60**	Health hazard/
**61**	healthy.ti.	**61**	West Nile disease.ti.	**61**	Life expectancy/
**62**	mental.ti.	**62**	west nile fever.ti.	**62**	Child development/
**63**	mental.ti.	**63**	global disease*.ab.	**63**	dermatology/
**64**	malaria.ti.	**64**	global health.ab.	**64**	hygiene/
**65**	malaria.ti.	**65**	well being.ab.	**65**	virus detection/
**66**	dengue.ti.	**66**	wellbeing.ab.	**66**	genotoxicity/
**67**	respiratory.ti.	**67**	human health.ab.	**67**	Allergic rhinitis/
**68**	infection*.ti.	**68**	vector borne disease*.ab.	**68**	women's health/
**69**	wellbeing.ti.	**69**	health implication*.ab.	**69**	exp leishmania/
**70**	well being.ti.	**70**	public health.ab.	**70**	encephalitis/
**71**	outbreak*.ti.	**71**	health consequence*.ab.	**71**	Child health/
**72**	zika.ti.	**72**	mental health.ab.	**72**	Communicable disease/
**73**	undernutrition.ti.	**73**	reproductive health.ab.	**73**	virus vector/
**74**	influenza.ti.	**74**	health adaptation.ab.	**74**	infant mortality/
**75**	hospitali?ation.ti.	**75**	(mortality adj2 morbidity).ab.	**75**	Health disparity/
**76**	epidemic.ti.	**76**	infectious disease*.ab.	**76**	Psychological well being/
**77**	ecohealth.ti.	**77**	health outcomes.ab.	**77**	Reproductive health/
**78**	ebola.ti.	**78**	health vulnerability.ab.	**78**	Tropical medicine/
**79**	death.ti.	**79**	(health adj2 impact*).ab.	**79**	Vulnerable population/
**80**	kills.ti.	**80**	(health adj2 threat*).ab.	**80**	Allergic disease/
**81**	cholera.ti.	**81**	(burden adj2 disease*).ab.	**81**	Maternal welfare/
**82**	foodborne.ti.	**82**	(population adj2 health).ab.	**82**	Toxoplasma gondii/
**83**	epidemics.ti.	**83**	(health adj2 effect*).ab.	**83**	Disease burden/
**84**	endemic.ti.	**84**	(health adj2 risk*).ab.	**84**	Childhood mortality/
**85**	pandemic.ti.	**85**	(health adj2 benefit*).ab.	**85**	Dengue virus/
**86**	syndrome.ti.	**86**	(health adj2 co-benefit*).ab.	**86**	Infectious agent/
**87**	asthma.ti.	**87**	mental disorder*.ab.	**87**	respiratory tract allergy/
**88**	illness*.ti.	**88**	Noncommunicable Disease*.ab.	**88**	enterovirus/
**89**	morbidity.ti.	**89**	malaria.ab.	**89**	anopheles/

**90**	cancer.ti.	**90**	syndrome.ab.	**90**	pollen allergy/
**91**	malnutrition.ti.	**91**	(tree or trees or soil).ti.	**91**	campylobacter/
**92**	mental health*.ti.	**92**	(people or human* or public health or men or women or children or patients or students).af.	**92**	exp Heat injury/
**93**	(global adj2 nutrition*).ti.	**93**	(editorial or letter or comment).pt.	**93**	Global health/
**94**	(population adj2 nutrition*).ti.	**94**	or/1-15	**94**	Non communicable disease/
**95**	(security adj2 nutrition*).ti.	**95**	or/16-90	**95**	norovirus/
**96**	(insecurity adj2 nutrition*).ti.	**96**	94 and 95	**96**	Ebola hemorrhagic/
**97**	(global adj2 food adj2 (supply or production)).ti.	**97**	96 not 91	**97**	Health impact assessment/
**98**	(security adj2 food).ti.	**98**	97 and 92	**98**	Yellow fever/
**99**	(insecurity adj2 food).ti.	**99**	limit 98 to yr="2007 -2019"	**99**	leptospira/
**100**	Chikungunya.ti.	**100**	limit 99 to abstracts	**100**	chikungunya/
**101**	Hantavirus.ti.	**101**	100 not 93	**101**	Arbovirus/
**102**	West Nile virus.ti.			**102**	tick-borne disease/
**103**	west nile fever.ti.			**103**	Food insecurity/
**104**	global disease*.ab.			**104**	Premature mortality/
**105**	global health.ab.			**105**	Trihalomethanes/
**106**	well being.ab.			**106**	population health/
**107**	wellbeing.ab.			**107**	Japanese encephalitis/
**108**	human health.ab.			**108**	Crimean-Congo hemorrhagic fever/
**109**	vector borne disease*.ab.			**109**	urban health/
**110**	health implication*.ab.			**110**	disease*.ti.
**111**	public health.ab.			**111**	cancer.ti.
**112**	health consequence*.ab.			**112**	health.ti.
**113**	mental health.ab.			**113**	infection*.ti.
**114**	reproductive health.ab.			**114**	mortality.ti.
**115**	health adaptation.ab.			**115**	respiratory.ti.
**116**	(mortality adj2 morbidity).ab.			**116**	death.ti.
**117**	infectious disease*.ab.			**117**	healthy.ti.

**118**	syndrome.ab.			**118**	mental.ti.
**119**	health outcomes.ab.			**119**	asthma.ti.
**120**	health vulnerability.ab.			**120**	influenza.ti.
**121**	(health adj2 impact*).ab.			**121**	illness*.ti.
**122**	(health adj2 threat*).ab.			**122**	malaria.ti.
**123**	(burden adj2 disease*).ab.			**123**	infectious.ti.
**124**	(population adj2 health).ab.			**124**	outbreak*.ti.
**125**	(health adj2 effect*).ab.			**125**	hospitali?ation*.ti.
**126**	(health adj2 risk*).ab.			**126**	epidemic.ti.
**127**	(health adj2 benefit).ab.			**127**	dengue.ti.
**128**	(health adj2 co-benefit*).ab.			**128**	endemic.ti.
**129**	mental disorder*.ab.			**129**	well being.ti.
**130**	Noncommunicable Disease*.ab.			**130**	pandemic.ti.
**131**	malaria.ab.			**131**	cholera.ti.
**132**	mycotoxins/ not food contamination/			**132**	ebola.ti.
**133**	respiratory tract diseases/			**133**	zika.ti.
**134**	Aspergillus/			**134**	west nile virus.ti.
**135**	Candida/			**135**	epidemics.ti.
**136**	exp candida/			**136**	wellbeing.ti.
**137**	exp aspergillus/			**137**	Hantavirus.ti.
**138**	Disease Susceptibility/			**138**	(insecurity adj2 food).ti.
**139**	encephalitis/			**139**	kills.ti.
**140**	HIV infections/			**140**	(global adj2 food adj2 (supply or production)).ti.
**141**	bacterial infection/			**141**	flavivirus.ti.
**142**	or/1-17			**142**	(global adj2 nutrition*).ti.
**143**	or/18-131			**143**	(security adj2 nutrition*).ti.
**144**	or/18-141			**144**	ecohealth.ti.

**145**	(tree or trees).ti.			**145**	(security adj2 food).ti.
**146**	soil.ti.			**146**	(mortality adj2 morbidity).ab.
**147**	exp animals/ not humans.sh.			**147**	public health.ab.
**148**	142 and 143			**148**	mental health.ab.
**149**	142 and 144			**149**	infectious disease*.ab.
**150**	148 not 145			**150**	well being.ab.
**151**	150 not 146			**151**	malaria.ab.
**152**	151 not 147			**152**	health outcomes.ab.
**153**	149 not 145			**153**	(health adj2 effect*).ab.
**154**	153 not 146			**154**	human health.ab.
**155**	154 not 147			**155**	mental disorder*.ab.
**156**	155 NOT 152			**156**	(burden adj2 disease*).ab.
**157**	limit 152 to yr="2007 - Current"			**157**	(health adj2 impact*).ab.
**158**	limit 155 to yr="2007 - Current"			**158**	wellbeing.ab.
**159**	(editorial or letter or comment).pt.			**159**	global health.ab.
**160**	157 not 159			**160**	gastroenteritis.ab.
**161**	158 not 159			**161**	(population adj2 health).ab.
				**162**	reproductive health.ab.
				**163**	(health adj2 threat*).ab.
				**164**	health consequence*.ab.
				**165**	health implication*.ab.
				**166**	flavivirus.ab.
				**167**	aeroallergens.ab.
				**168**	vector borne disease*.ab.
				**169**	(health adj2 co-benefit*).ab.
				**170**	health adaptation.ab.
				**171**	or/1-17
				**172**	or/18-170
				**173**	(tree or trees).ti.
				**174**	soil.ti.
				**175**	(exp animal/ or nonhuman/) not exp human/
				**176**	or/172-174

				**177**	171 and 172
				**178**	177 not 176
				**179**	limit 178 to yr="2007 - 2019"
				**180**	limit 179 to abstracts

Data

1. Articles in scientific journals were searched in the Ovid Embase and Ovid Medline databases. The bibliometric search worked with specific inclusion and exclusion criteria that were applied to capture only the most relevant literature. This includes peer-reviewed scientific articles on health and climate change in English, with no direct restriction to country or population applied. All peer-reviewed articles, originating from Chinese institutions, and reporting the findings of original qualitative and quantitative studies will be included, together with reviews, editorials, viewpoints, letters or comments.

Caveats

The methodology provided here enables a quantitative appraisal of the research question. The quality of the data and the specifics of its content are not assessed by the indicator team. However, with the outputs all published in peer-reviewed journals, there is a de facto check on quality. For this reason, the indicator does not cover grey literature.

Future Form of Indicator

There is scope to formulate add-ons to the indicator, for example focusing on trends in scientific coverage of particular climate-sensitive health outcomes.

Additional information to Figure 3 in the maintext

Figure 3 in the maintext displayed the key rising health risks from climate change in each province in China when only considering indicators shown in the maintext. As the findings of more indicators are displayed in the appendix, the following ***Figure 47*** gives a more comprehensive picture on the key risking health risks from climate change in each province.

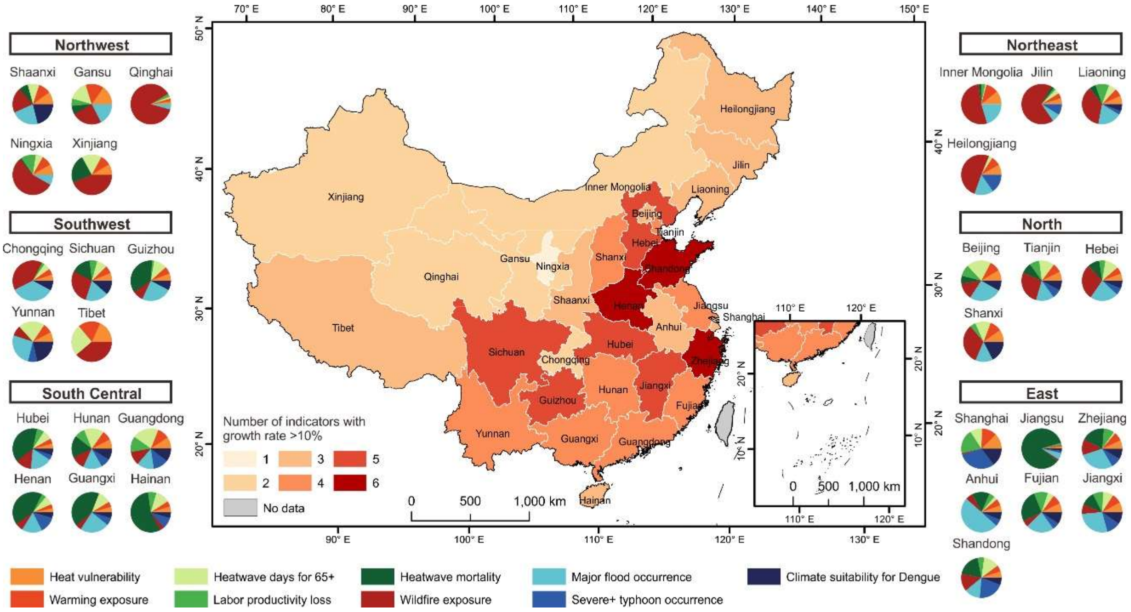

Figure 47. The key rising health risks from climate change in each province in China.
Each province is colored by the number of indicators with growth rate larger than 10% between 2000 and 2019. Each color in the pie chart represents one aspect of health risk, such as heat vulnerability, wildfire exposure and etc. Slice area is proportional to the changing rates of different health risks. The provinces are grouped by geographic regions. This figure indicates that no province is immune to the health impact of climate change, but different provinces have their unique health threats. In Shandong province, the top three rapidly increasing health risks are respectively occurrences of severe and super typhoon, heatwave-related mortality and wildfire exposure. Although Henan is next to Shandong, the top three rapidly increasing health risks in Henan are different.

Additional information to Figure 12 in the maintext

Figure 12 in the maintext only displays the the trend of selected indicators. For some indicators, we have displayed the results of several sub-indicators in figure 12. For example, indicator 3.1 contains reduction of carbon intensity, coal phase-out and low-carbon electricity, which have all been shown in figure 12. ***Table 48*** explains the detailed description of each indicator behind the figure 12 in the maintext.

Table 48: Description of of each indicator presented in indicator trend overview of 2020 China Lancet Countdown assessment report

		Name of indicator	Description of of each indicator
Impact	1	Heatwave days in 65+	Change in heatwave exposure days per person aged 65+ in China, compared to 1985-2005
	2	Heatwave-related mortality	The number of heatwave-related mortality in China
	3	Labour productivity loss	The average heat-related work hours loss per worker in China
	4	Severe+ typhoon occurrence	The occurences of cyclones larger than typhoon (i.e. severe typhoon and super typhoon)
	5	Climate suitability for dengue	The vectorial capacity of Aedes
	6	Costs of heatwave-related mortality	Annual national monetized value of heatwave-related mortality US$Billion
Response	1	Reduction of carbon intensity	Reduction of carbon intensity of the energy system, compared with year 2011 (sectoral approach)
	2	Coal phase-out	Share of Coal in TPES
	3	Low-carbon electricity	share of low-carbon electricity generation in total electricity generation

	4	Clean household energy use	Share of electricity in total household energy consumption
	5	Reduction of urban air pollution	Reduction of annual average PM2.5 concentrations of China's cities compared with year 2014
	6	Reduction of fossil fuel subsidies	Reduction of fossil fuel subsidies US$ billion, compared with year 2010

References

1. Watts N, Amann M, Arnell N, et al. The 2019 report of The Lancet Countdown on health and climate change: ensuring that the health of a child born today is not defined by a changing climate. *Lancet* 2019; **394**(10211): 1836-78.
2. Service(C3S) CCC. ERA5: Fifth generation of ECMWF atmospheric reanalyses of the global climate. Copernicus Climate Change Service Climate Data Store (CDS).
3. Chambers J. Hybrid gridded demographic data for the world, 1950-2020. 1.0 ed. Zenodo; 2020.
4. Xu Z, FitzGerald G, Guo Y, Jalaludin B, Tong S. Impact of heatwave on mortality under different heatwave definitions: A systematic review and meta-analysis. *Environ Int* 2016; **89-90**: 193-203.
5. Tian Z, Li S, Zhang J, Guo Y. The characteristic of heat wave effects on coronary heart disease mortality in Beijing, China: a time series study. *PLoS One* 2013; **8**(9): e77321.
6. Chen K, Bi J, Chen J, Chen X, Huang L, Zhou L. Influence of heat wave definitions to the added effect of heat waves on daily mortality in Nanjing, China. *Sci Total Environ* 2015; **506-507**: 18-25.
7. Oudin Astrom D, Schifano P, Asta F, et al. The effect of heat waves on mortality in susceptible groups: a cohort study of a mediterranean and a northern European City. *Environ Health* 2015; **14**: 30.
8. Yang J, Yin P, Sun J, et al. Heatwave and mortality in 31 major Chinese cities: Definition, vulnerability and implications. *Sci Total Environ* 2019; **649**: 695-702.
9. Kim YO, Lee W, Kim H, Cho Y. Social isolation and vulnerability to heatwave-related mortality in the urban elderly population: A time-series multi-community study in Korea. *Environ Int* 2020; **142**: 105868.
10. WPP U. United Nations-World Population Prospects. 2020 (accessed May 23th 2020).
11. United Nations Statistical Division (UNSD). International standard industrial classification of all economic activities, revision 4 (ISIC Rev.4). New York, United Nations: United Nations Publications; 2008.
12. Kjellstrom T, Freyberg C, Lemke B, Otto M, Briggs D. Estimating population heat exposure and impacts on working people in conjunction with climate change. *Int J Biometeorol* 2018; **62**(3): 291-306.
13. NBS. China Statistical Yearbook. 2020 2020. http://www.stats.gov.cn/tjsj/ndsj/ (accessed May 23th 2020).
14. Cao Q, Yu D, Georgescu M, Wu J, Wang W. Impacts of future urban expansion on summer climate and heat-related human health in eastern China. *Environ Int* 2018; **112**: 134-46.
15. Dang TN, Van DQ, Kusaka H, Seposo XT, Honda Y. Green Space and Deaths Attributable to the Urban Heat Island Effect in Ho Chi Minh City. *Am J Public Health* 2018; **108**(S2): S137-s43.
16. Zhang W, Zheng C, Chen F. Mapping heat-related health risks of elderly citizens in mountainous area: A case study of Chongqing, China. *Sci Total Environ* 2019; **663**: 852-66.
17. Watts N, Amann M, Arnell N, et al. The 2018 report of the Lancet Countdown on health and climate change: shaping the health of nations for centuries to come. *Lancet* 2018; **392**(10163): 2479-514.
18. Beggs PJ, Zhang Y, Bambrick H, et al. The 2019 report of the MJA-Lancet Countdown on health and climate change: a turbulent year with mixed progress. *The Medical journal of Australia* 2019; **211**(11): 490-1.e21.

19. He C, Ma L, Zhou L, et al. Exploring the mechanisms of heat wave vulnerability at the urban scale based on the application of big data and artificial societies. *Environ Int* 2019; **127**: 573-83.
20. Brooks N, Neil Adger W, Mick Kelly P. The determinants of vulnerability and adaptive capacity at the national level and the implications for adaptation. *Global Environmental Change* 2005; **15**(2): 151-63.
21. NASA EarthData Active Fire Data. 2020. https://earthdata.nasa.gov/earth-observation-data/near-real-time/firms/active-fire-data (accessed 20200425 2020).
22. NASA. Near Real-Time and MCD14DL MODIS Active Fire Detections. 2020. https://earthdata.nasa.gov/active-fire-data (accessed May 23th 2020).
23. Chambers J. Hybrid gridded demographic data for the world, 1950-2020. April 27, 2020 ed; 2020.
24. Black C, Tesfaigzi Y, Bassein JA, Miller LA. Wildfire smoke exposure and human health: Significant gaps in research for a growing public health issue. *Environ Toxicol Pharmacol* 2017; **55**: 186-95.
25. Ying M, Zhang W, Yu H, et al. An Overview of the China Meteorological Administration Tropical Cyclone Database. *Journal of Atmospheric & Oceanic Technology* 2014; **31**(2): 287-301.
26. Mann HB. Non-Parametric Test Against Trend. *Econometrica* 1945; **13**(3): 245-59.
27. Tropical cyclone data center of China Meteorological Administration: Path of tropical cyclone. 2020. http://tcdata.typhoon.org.cn/ (accessed May 23 2020).
28. China(Taiwan) NSRo. *https://engstatgovtw/mpasp?mp=5*; **Accessed: July 5, 2020**.
29. Paterson DL, Wright H, Harris PNA. Health Risks of Flood Disasters. *Clin Infect Dis* 2018; **67**(9): 1450-4.
30. Ault TR. On the essentials of drought in a changing climate. *Science* 2020; **368**(6488): 256-60.
31. Centre for Research on the Epidemiology of Disasters. EM-DAT The International Disaster Database. 2020. https://emdat.be/ (accessed June 2 2020).
32. Wirtz A, Kron W, Löw P, Steuer M. The need for data: natural disasters and the challenges of database management. *Natural Hazards* 2014; **70**(1): 135-57.
33. Ma F, Ye A, You J, Duan Q. 2015-16 floods and droughts in China, and its response to the strong El Niño. *The Science of the total environment* 2018; **627**: 1473-84.
34. Rocklöv J, Tozan Y. Climate change and the rising infectiousness of dengue. *Emerging Topics in Life Sciences* 2019; **3**(2).
35. Jing L-H, Hans S, Annelies W-S, Joacim R. Vectorial capacity of Aedes aegypti: effects of temperature and implications for global dengue epidemic potential. *PloS one* 2014; **9**(3).
36. Network. GBoDC. Global Burden of Disease Study 2017 (GBD 2017) Results. Seattle, United States: Institute for Health Metrics and Evaluation (IHME); 2018.
37. Zhou M, Wang H, Zeng XY, et al. Mortality, morbidity, and risk factors in China and its provinces, 1990-2017: a systematic analysis for the Global Burden of Disease Study 2017. *The Lancet* 2019; **394**(10204): 1145-58.
38. Wu J GX. A set of daily observations of Chinese grid data and comparison with other data [J]. *Earth and Planetary Physics* 2013; **56**(04): 1102-11.
39. JC F. Impact of climate change on dengue fever and its adaptability. *Chinese Center for Disease Control and Prevention* 2013.
40. WHO. The IHR core capacity scores 2019. https://www.who.int/data/gho/indicator-metadata-registry/imr-details/4672 (accessed May 23 2020).
41. Li R, Xu L, Bjørnstad ON, et al. Climate-driven variation in mosquito density predicts the spatiotemporal dynamics of dengue. 2019; **116**(9): 3624-9.
42. Liu-Helmersson J, Quam M, Wilder-Smith A, et al. Climate Change and Aedes Vectors: 21st Century Projections for Dengue Transmission in Europe. *EBioMedicine* 2016; **7**.

43. People’s Republic of China. China’s National Plan in Response to Climate Change (in Chinese). 2007.

44. China’s National Development and Reform Commission and 8 other ministries. China’s National Climate Change Adaptation Strategy (in Chinese). 2013.

45. China’s National Development and Reform Commission. China’s National Climate Change Planning (2014-2020) (in Chinese). 2014.

46. Qin D. Climate and Environmental Evolution in China: 2012: China Meteorological Press; 2012.

47. Writing Committee of The Third National Assessment Report on Climate Change. The Third National Assessment Report on Climate Change: Science Press; 2015.

48. China NBoSo. Statistical Communiqué of the People's Republic of China on the 2019 National Economic and Social Development. Beijing, China: National Bureau of Statistics of China; 2020.

49. CDP. Data cities. London, UK: CDP; 2020.

50. MOHURD, NDRC. Action Plan for Urban Adaptation to Climate Change. Beijing, China: Ministry of Housing and Urban-Rural Development of the People’s Republic of China, National Development and Reform Commission of the People’s Republic of China; 2016.

51. MOHURD, NDRC. Notice of the MOHURD and NDRC on Issuing the Constrcution of the Pilot Climate-adaptive Cities. Beijing, China: Ministry of Housing and Urban-Rural Development of the People’s Republic of China, National Development and Reform Commission of the People’s Republic of China; 2017.

52. Congress SCotNPs. Law of the People's Republic of China on the Prevention and Treatment of Infectious Diseases. Beijing, China; 2013.

53. China NBoSo. China Statistical Yearbook 2019. Beijing: China Statistics Press; 2019.

54. China MoHaU-RDoPsRo. China Urban and Rural Construction Statistical Yearbook 2018. Beijing: China Statistics Press; 2018.

55. Port-of-entry CAo. China Port Statistical Yearbook 2016. Beijing: China Customs Press; 2016.

56. State Information Center of China CIIA. China Information Almanac 2017. Beijing: China Information Almanac Periodical Press; 2017.

57. China NHCoPsRo. China Health Statistics Yearbook 2019. Beijing: China Union Medical University Press; 2019.

58. Department of Industry Statistics NBoSoC. China Industry Statistical Yearbook 2017. Beijing: China Statistics Press; 2017.

59. China MoCAoPsRo. China Civil Affairs’ Statistics Yearbook 2017. Beijing: China Statistics Press; 2017.

60. Zheng J, Han W, Jiang B, Ma W, Zhang Y. Infectious Diseases and Tropical Cyclones in Southeast China. *International journal of environmental research and public health* 2017; **14**(5).

61. Jiao K, Hu W, Ren C, Xu Z, Ma W. Impacts of tropical cyclones and accompanying precipitation and wind velocity on childhood hand, foot and mouth disease in Guangdong Province, China. *Environmental Research* 2019; **173**(JUN.): 262-9.

62. Wu SM, Zheng XY, Wei C. Measurement of inequality using household energy consumption data in rural China. *Nature Energy* 2017; **2**(10).

63. National Emergency Early Warning Information Release System. *http://www12379cn/* 2015; **accessed 20 May 2020**.

64. Center NEW. Early Warning Report 2018, 2019.

65. Center NEW. Early Warning Report 2019, 2020.

66. Patz JA, Campbell-Lendrum D, Holloway T, Foley JA. Impact of regional climate change on human health. *Nature* 2005; **438**(7066): 310-7.

67. Administration CM. Measures for the Release and Dissemination of Early Warning Signals for

Meteorological Disasters. Beijing, China: China Meteorological Administration 2007.
68. NBSC. China Statistical Yearbook Beijing: China Statistics Press; 2001-2019.
69. Shan Y, Guan D, Zheng H, et al. China CO2 emission accounts 1997-2015. *Sci Data* 2018; **5**: 170201.
70. Shan Y, Liu J, Liu Z, et al. New provincial CO2 emission inventories in China based on apparent energy consumption data and updated emission factors. *Applied Energy* 2016; **184**: 742-50.
71. Shan Y, Huang Q, Guan D, Hubacek K. China CO2 emission accounts 2016–2017. *Scientific Data* 2020; **7**(1): 1-9.
72. National Data. National Bureau of Statistics of China. 2019. http://data.stats.gov.cn/ (accessed May 5, 2020).
73. Association CNC. Coal Industry Annual Development Report. 2019. http://www.coalchina.org.cn/index.php?m=content&c=index&a=show&catid=9&id=118819 (accessed June 1, 2020).
74. IEA. Global Energy Review 2020: The impacts of the Covid-19 crisis on global energy demand and CO2 emissions., 2020.
75. NBSC. China Statistical Yearbook 2018. Beijing: China Statistics Press; 2019.
76. NBSC. China Energy Statistical Yearbook Beijing: China Statistics Press; 2001-2018.
77. (MEPC) Ministry of Environmental Protection of China. Technical regulation for selection of ambient air quality monitoring stations (on trial). http://www.cnemc.cn/publish/totalWebSite/news/news_38841.html; 2013.
78. Zhao H, Geng G, Zhang Q, et al. Inequality of household consumption and air pollution-related deaths in China. *Nat Commun* 2019; **10**(1): 4337.
79. Xie Y, Dai H, Zhang Y, Wu Y, Hanaoka T, Masui T. Comparison of health and economic impacts of PM2.5 and ozone pollution in China. *Environ Int* 2019; **130**: 104881.
80. Zhang Q, Zheng YX, Tong D, et al. Drivers of improved PM2.5 air quality in China from 2013 to 2017. *Proceedings of the National Academy of Sciences of the United States of America* 2019; **116**(49): 24463-9.
81. Amann M, Bertok I, Borken-Kleefeld J, et al. Cost-effective control of air quality and greenhouse gases in Europe: Modeling and policy applications. *Environ Model Softw* 2011; **26**(12): 1489–501.
82. Simpson D, Benedictow A, Berge H, et al. The EMEP MSC-W chemical transport model – Technical description. *ATMOSPHERIC CHEMISTRY AND PHYSICS* 2012; **12**(16): 7825-65.
83. World Health Organisation. Ambient Air Pollution: A Global Assessment of Exposure and Burden of Disease. Geneva, Switzerland: World Health Organisation, 2016.
84. Burnett RT, Pope CA, 3rd, Ezzati M, et al. An integrated risk function for estimating the global burden of disease attributable to ambient fine particulate matter exposure. *Environmental health perspectives* 2014; **122**(4): 397-403.
85. Zhou M, Wang H, Zhu J, et al. Cause-specific mortality for 240 causes in China during 1990-2013: a systematic subnational analysis for the Global Burden of Disease Study 2013. *Lancet* 2016; **387**(10015): 251-72.
86. China MoEPotPsRo. China Vehicle Environmental Management Annual Report. In: China MoEPotPsRo, editor.; 2009-2019.
87. China NBoSo. National data. 2020.
88. National Private Vehicle Ownership *The Ministry of Public Security of the People's Republic of China* 2020.
89. Gu B, Ju X, Chang J, Ge Y, Vitousek PM. Integrated reactive nitrogen budgets and future trends in China. *Proceedings of the National Academy of Sciences* 2015; **112**(28): 8792.
90. Zhang D, Shen J, Zhang F, Li Ye, Zhang W. Carbon footprint of grain production in China. *Scientific*

Reports 2017; **7**(1): 4126.
91. Zhang H, Ye X, Cheng T, et al. A laboratory study of agricultural crop residue combustion in China: Emission factors and emission inventory. *Atmospheric Environment* 2008; **42**(36): 8432-41.
92. Ministry of Ecology and Environment of the People's Republic of China. Technical guidelines for compiling the inventory of atmospheric ammonia emission (for Trial Implementation). 2014.
93. Ma L, Velthof GL, Wang FH, et al. Nitrogen and phosphorus use efficiencies and losses in the food chain in China at regional scales in 1980 and 2005. *Science of The Total Environment* 2012; **434**: 51-61.
94. Bai ZH, Ma L, Oenema O, Chen Q, Zhang FS. Nitrogen and Phosphorus Use Efficiencies in Dairy Production in China. *Journal of Environmental Quality* 2013; **42**(4): 990-1001.
95. Chen X, Cui Z, Fan M, et al. Producing more grain with lower environmental costs. *Nature* 2014; **514**(7523): 486-9.
96. West PC, Gerber JS, Engstrom PM, et al. Leverage points for improving global food security and the environment. *Science* 2014; **345**(6194): 325.
97. Ministry of Agriculture of the People's Republic of China. The Plan of Zero Growth in Chemical Fertilizer and Pesticide Use by 2020. 2015. http://jiuban.moa.gov.cn/zwllm/tzgg/tz/201503/t20150318_4444765.htm.
98. Partridge I, Gamkhar S. A methodology for estimating health benefits of electricity generation using renewable technologies. *Environment International* 2012; **39**(1): 103-10.
99. National Data. National Bureau of Statistics of China. http://data.stats.gov.cn/ (accessed May 2 2020).
100. Xia Y, Li Y, Guan D, et al. Assessment of the economic impacts of heat waves: A case study of Nanjing, China. *Journal of Cleaner Production* 2018; **171**: 811-9.
101. National Bureau of Statistics of China. Input-Output Tables. 2020. http://data.stats.gov.cn/files/html/quickSearch/trcc/trcc07.html (accessed 5 May 2020).
102. Heran Z, Zengkai Z, Wendong W, et al. Regional determinants of China’s consumption-based emissions in the economic transition. *Environmental Research Letters* 2020.
103. Xia Y, Guan D, Jiang X, Peng L, Schroeder H, Zhang Q. Assessment of socioeconomic costs to China’s air pollution. *Atmospheric Environment* 2016; **139**: 147-56.
104. Xia Y, Guan D, Meng J, Li Y, Shan Y. Assessment of the pollution-health-economics nexus in China. *Atmospheric Chemistry and Physics* 2018; **18**(19): 14433-43.
105. Miller RE, Blair PD. Input-output analysis: foundations and extensions: Cambridge university press; 2009.
106. National Bureau of Statistics of China. Tabulation on the 2010 population census of the People's Republic of China. Beijing, China: China Statistics Press; 2010.
107. Koks EE, Bockarjova M, De Moel H, Aerts JCJH. Integrated direct and indirect flood risk modeling: development and sensitivity analysis. *Risk Anal* 2015; **35**(5): 882-900.
108. Zeng Z, Guan D. Methodology and application of flood footprint accounting in a hypothetical multiple two-flood event. *Philosophical Transactions of the Royal Society A: Mathematical, Physical and Engineering Sciences* 2020; **378**(2168): 20190209.
109. Zeng Z, Guan D, Steenge AE, Xia Y, Mendoza-Tinoco D. Flood footprint assessment: a new approach for flood-induced indirect economic impact measurement and post-flood recovery. *Journal of Hydrology* 2019; **579**: 124204.
110. Mendoza-Tinoco D, Hu Y, Zeng Z, et al. Flood Footprint Assessment: A Multiregional Case of 2009 Central European Floods. *Risk Anal* 2020.
111. Chow GC. Capital formation and economic growth in China. *The Quarterly Journal of Economics* 1993; **108**(3): 809-42.

112. Holz CA. New capital estimates for China. *China Economic Review* 2006; **17**(2): 142-85.

113. OECD. Measuring capital -- OECD manual on the measurement of capital stocks, consumption of fixed capital and capital services; 2001.

114. Munich RE. NatCatSERVICE. Munich, Germany: Munich RE; 2020.

115. WBG. World development indicators. Washington, DC, USA: World Bank Group; 2020.

116. International Monetary Fund (IMF). Investment and Capital Stock Dataset (ICSD). 2020. https://data-imf-org.uea.idm.oclc.org/?sk=1CE8A55F-CFA7-4BC0-BCE2-256EE65AC0E4 (accessed 11 May 2020).

117. National Bureau of Statistics of China. Statistical Yearbook of the Chinese Investment in Fixed Assets. Beijing, China: China Statistics Press; 2018.

118. Hallegatte S. An adaptive regional input-output model and its application to the assessment of the economic cost of Katrina. *Risk Anal* 2008; **28**(3): 779-99.

119. Wind Economic Database 2020. In: Wind Information Co. Lhwwcceeh, editor. Shanghai; 2020.

120. NEA. The National Energy Administration of PRC. *2019 National Power Industry Statistics* 2020; **NEA: Beijing, China,** : http://www.nea.gov.cn/2020-01/20/c_138720881.htm.

121. China National Renewable Energy Center (CNREC). China Renewable Energy Outlook 2019. Beijing, 2020.

122. National Data. *National Bureau of Statistics of China* 2020; **http://data.stats.gov.cn/english/**(Accessed: May 5, 2020).

123. Murdock HE, Gibb D, André T, et al. Renewables 2019 Global Status Report. *https://wwwren21net/gsr-2019/* 2019.

124. CEIC. China Employment in Fossil Fuel Extraction *CEIC Global Economic Data, Indicators, Charts & Forecasts [Online]* 2012-2019; **Available at: https://www.ceicdata.com/zh-hans/china/no-of-employee-by-industry-monthly/no-of-employee-petroleum-coking--nuclear-fuel; https://www.ceicdata.com/zh-hans/china/no-of-employee-by-industry-monthly/no-of-employee-coal-mining--dressing**.

125. IEA. Fossil-fuel consumption subsidies by country. 2018. https://www.iea.org/data-and-statistics/charts/fossil-fuel-consumption-subsidies-by-country-2018 (accessed May 5, 2020).

126. IEA. Energy Subsidies. Paris, France: International Energy Agency, 2019.

127. WBG. Carbon Pricing Dashboard. In: Group WB, editor. Washington, DC, USA; 2019.

128. JRC. GHG (CO2, CH_4, N_2O, F-gases) emission time series 1990-2012 by region/country. 2016.

129. Statista. Monthly sales volume of newspapers in China 2011-2020. 2020. https://www.statista.com/statistics/277979/amount-of-newspapers-sold-in-china-by-month/ (accessed July 2020).

130. Statista. Number of Sina Weibo users in China 2017-2021. 2019. https://www.statista.com/statistics/941456/china-number-of-sina-weibo-users/ (accessed July 2020).

131. Baidu Announces First Quarter 2019 Results. *http://irbaiducom/news-releases/news-release-details/baidu-announces-first-quarter-2019-results* 2020.

132. Search Engine Market Share China June 2019 - June 2020. *https://gsstatcountercom/search-engine-market-share/all/china*.

133. Zhang Y, Beggs PJ, Bambrick H, et al. The MJA–Lancet Countdown on health and climate change: Australian policy inaction threatens lives. *Medical Journal of Australia* 2018; **209**(11): 474-.

134. Li GD ZJ, Jiao GJ, Zhao ZS. Research progress on the impact of climate change on the outbreak of infectious diseases [J]. *Acta Ecologica Sinica* 2013; **33**(21): 6762-73.

135. Wu XX TH, Zhou S, Chen LF, Xun B. The impact of global change on the occurrence and spread of human infectious diseases [J] *SCIENCE CHINA Earth Sciences* 2013; **43**(11): 1743-59.
